# Effect of Spinal Manipulative and Mobilization Therapies in Young Adults With Mild to Moderate Chronic Low Back Pain: A Randomized Clinical Trial

**DOI:** 10.1001/jamanetworkopen.2020.12589

**Published:** 2020-08-05

**Authors:** James S. Thomas, Brian C. Clark, David W. Russ, Christopher R. France, Robert Ploutz-Snyder, Daniel M. Corcos

**Affiliations:** 1 Department of Physical Therapy, Virginia Commonwealth University, Richmond; 2 Department of Physical Medicine and Rehabilitation, Virginia Commonwealth University, Richmond; 3 Division of Physical Therapy, Ohio University School of Rehabilitation and Communication Sciences, Athens; 4 Ohio Musculoskeletal and Neurological Institute, Ohio University, Athens; 5 Department of Biomedical Sciences, Ohio University, Athens; 6 Morsani College of Medicine, University of South Florida School of Physical Therapy and Rehabilitation Sciences, Tampa; 7 Department of Psychology, Ohio University, Athens; 8 Applied Biostatistics Laboratory, University of Michigan School of Nursing, Ann Arbor; 9 Feinberg School of Medicine, Department of Physical Therapy and Human Movement Sciences, Northwestern University, Chicago, Illinois

## Abstract

**Importance:**

Low back pain (LBP) is one of the most common reasons for seeking medical care. Manual therapy is a common treatment of LBP, yet few studies have directly compared the effectiveness of thrust (spinal manipulation) vs nonthrust (spinal mobilization) techniques.

**Objective:**

To evaluate the comparative effectiveness of spinal manipulation and spinal mobilization at reducing pain and disability compared with a placebo control group (sham cold laser) in a cohort of young adults with chronic LBP.

**Design, Setting, and Participants:**

This single-blinded (investigator-blinded), placebo-controlled randomized clinical trial with 3 treatment groups was conducted at the Ohio Musculoskeletal and Neurological Institute at Ohio University from June 1, 2013, to August 31, 2017. Of 4903 adult patients assessed for eligibility, 4741 did not meet inclusion criteria, and 162 patients with chronic LBP qualified for randomization to 1 of 3 treatment groups. Recruitment began on June 1, 2013, and the primary completion date was August 31, 2017. Data were analyzed from September 1, 2017, to January 20, 2020.

**Interventions:**

Participants received 6 treatment sessions of (1) spinal manipulation, (2) spinal mobilization, or (3) sham cold laser therapy (placebo) during a 3-week period.

**Main Outcomes and Measures:**

Coprimary outcome measures were the change from baseline in Numerical Pain Rating Scale (NPRS) score over the last 7 days and the change in disability assessed with the Roland-Morris Disability Questionnaire (scores range from 0 to 24, with higher scores indicating greater disability) 48 to 72 hours after completion of the 6 treatments.

**Results:**

A total of 162 participants (mean [SD] age, 25.0 [6.2] years; 92 women [57%]) with chronic LBP (mean [SD] NPRS score, 4.3 [2.6] on a 1-10 scale, with higher scores indicating greater pain) were randomized. Fifty-four participants were randomized to the spinal manipulation group, 54 to the spinal mobilization group, and 54 to the placebo group. There were no significant group differences for sex, age, body mass index, duration of LBP symptoms, depression, fear avoidance, current pain, average pain over the last 7 days, and self-reported disability. At the primary end point, there was no significant difference in change in pain scores between spinal manipulation and spinal mobilization (0.24 [95% CI, −0.38 to 0.86]; *P* = .45), spinal manipulation and placebo (−0.03 [95% CI, −0.65 to 0.59]; *P* = .92), or spinal mobilization and placebo (−0.26 [95% CI, −0.38 to 0.85]; *P* = .39). There was no significant difference in change in self-reported disability scores between spinal manipulation and spinal mobilization (−1.00 [95% CI, −2.27 to 0.36]; *P* = .14), spinal manipulation and placebo (−0.07 [95% CI, −1.43 to 1.29]; *P* = .92) or spinal mobilization and placebo (0.93 [95% CI, −0.41 to 2.29]; *P* = .17).

**Conclusions and Relevance:**

In this randomized clinical trial, neither spinal manipulation nor spinal mobilization appeared to be effective treatments for mild to moderate chronic LBP.

**Trial Registration:**

ClinicalTrials.gov Identifier: NCT01854892

## Key Points

**Question:**

What is the comparative effectiveness of spinal manipulation compared with spinal mobilization relative to a placebo control in reducing pain and disability in chronic low back pain?

**Findings:**

In this randomized clinical trial that included 162 young adults, there was no difference in reduction of pain and disability when comparing spinal manipulation to spinal mobilization relative to the placebo control treatment.

**Meaning:**

Neither spinal manipulation nor mobilization appeared to be an effective intervention for young adults with mild to moderate chronic low back pain.

## Teaser

This randomized clinical trial evaluates the effectiveness of spinal manipulation and spinal mobilization compared with a placebo at reducing pain and disability among young adults with mild to moderate chronic low back pain.

## Introduction

Low back pain is one of the most common reasons for seeking care from a physician, with total costs exceeding $100 billion annually in the US alone.^1,2,3^ These costs are driven primarily by 7% to 10% of patients who develop chronic low back pain. Manipulative therapies are a common conservative treatment approach for low back pain, with more than 18 million American adults receiving manipulative therapies at a total annual out-of-pocket cost of $3.9 billion.^4^

Spinal manipulative treatments can be broadly classified as manipulation-based or mobilization-based techniques. Manipulation-based techniques apply a high-velocity, low-amplitude force to the spine and are often accompanied by an audible sound from 1 or more joints.^5^ In contrast, mobilization-based techniques use a low-velocity, low-force approach that generally does not produce audible joint sounds. Although the clinical effectiveness of manipulative therapy techniques for acute and subacute low back pain has been reported,^6,7,8^ the evidence on the effectiveness for reducing pain in patients with chronic low back pain is mixed.^9,10,11^ Reviews have generally found that manipulation is more effective compared with standard of care,^6^ but few studies have compared the clinical effectiveness of these 2 manipulative therapies,^12,13,14,15,16,17^ and existing studies have yielded discrepant results. Some studies indicate that manipulation-based techniques are more effective for low back pain compared with nonthrust techniques^13,14,16^; however, other studies report no significant difference in clinical outcomes between spinal manipulation and mobilization.^12,15,17^ Finally, most studies on manipulative therapies have used standard of care or waiting list controls as the comparison group.^6^ The current trial sought to use stronger control given the prevalence of both techniques in clinical practice and that understanding their relative effectiveness is an important and unresolved issue.

Accordingly, the RELIEF (Researching the Effectiveness of Lumbar Interventions for Enhancing Function) Study was conducted as a phase II randomized clinical trial to compare the effectiveness of spinal manipulation, spinal mobilization, and placebo (sham laser) in the treatment of pain and disability among young adults with chronic low back pain.

## Methods

### Study Design

**Figure 1.**
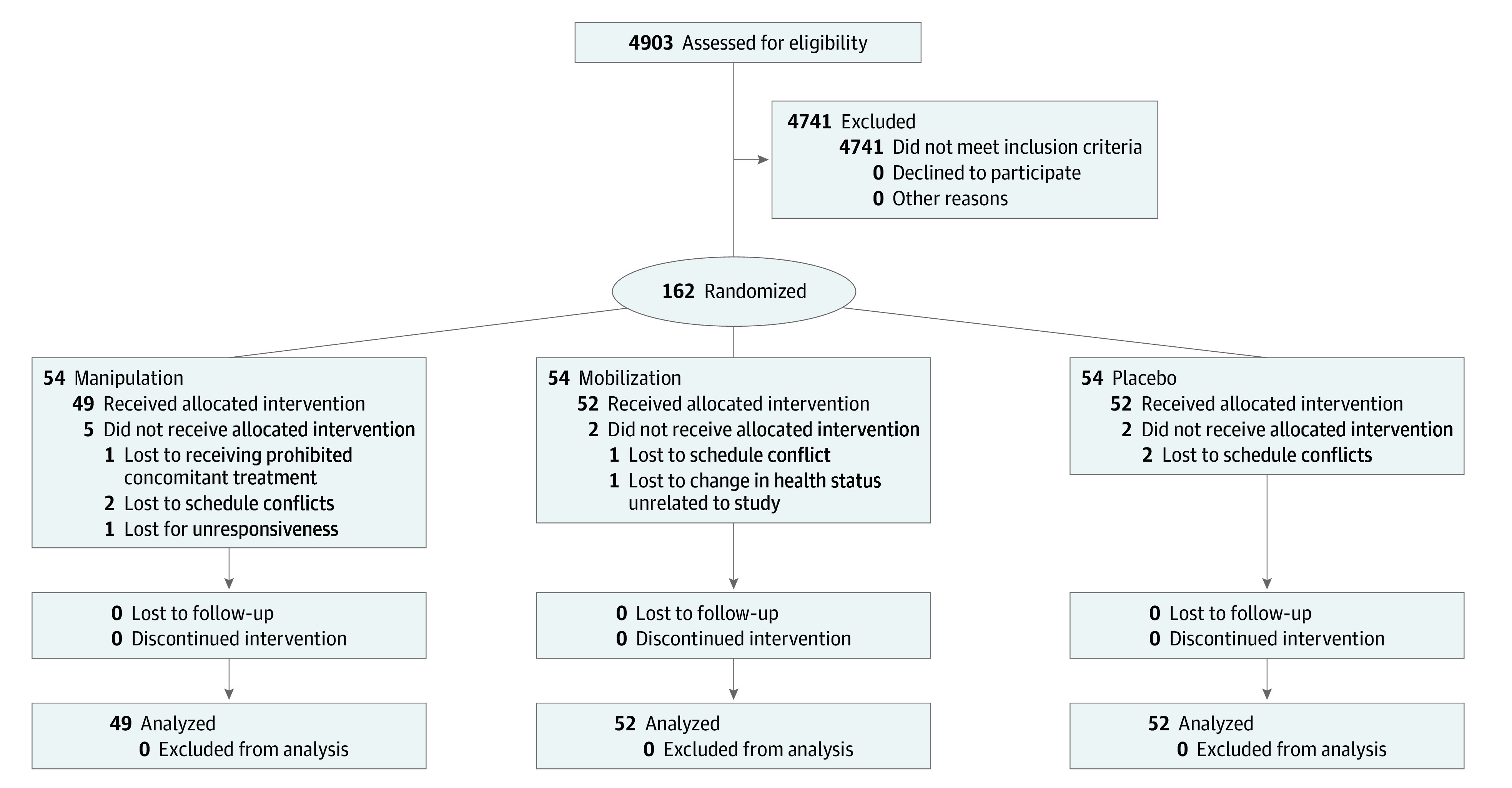
CONSORT Diagram

This randomized clinical trial was a single-blinded (investigator-blinded), placebo-controlled, 3-arm, parallel-groups trial with repeated measurements. The Ohio University institutional review board approved this study, and written informed consent was obtained from all participants. The detailed study protocol (Supplement 1) is published^18^ and registered on ClinicalTrials.gov (NCT01854892). This study was conducted according to the Consolidated Standards of Reporting Trials (CONSORT) reporting guideline. Recruitment began on June 1, 2013, and the primary completion date was August 31, 2017. Participants were randomized in a 1:1:1 ratio into 1 of 3 groups (Figure 1).

Group 1 received spinal manipulation treatment 2 times per week for 3 weeks (spinal manipulation). Group 2 received spinal mobilization treatment 2 times per week for 3 weeks. The placebo group received sham cold laser treatment 2 times per week for 3 weeks. Randomization was stratified by sex, using a permuted block design, for the purpose of maintaining balance across treatment groups. This-computer generated randomization table was maintained by the study coordinator. All outcome measures were obtained by study staff blinded to the assignment of treatment group.

### Study Participants

Patients with chronic low back pain (n = 162) were randomly assigned to the following treatment groups (54 patients per group): (1) spinal manipulation, (2) spinal mobilization, or (3) placebo. The Box lists the inclusion and exclusion criteria for the study. In brief, key inclusion criteria were: being 18 to 45 years of age, reporting low back pain constantly or on most days for the last 3 months and low back pain that has caused them to seek care or consultation from a health care practitioner, and average pain of at least 2 on a scale of 0 to 10 (with higher scores indicating greater pain) and a Roland-Morris Disability Questionnaire score of at least 4 (scores range from 0 to 24, with higher values indicating greater disability). To further reduce heterogeneity associated with low back pain, all potential participants completed a physical exam to determine whether they had at least 3 of 4 clinical characteristics that have been associated with positive response to spinal manipulation.^19^ In the original clinical prediction rule, an individual had to meet 4 of 5 characteristics (ie, symptoms for <16 days, no symptoms distal to the knee, low fear avoidance behavior, at least 1 hypomobile vertebral segment, and at least 1 hip with internal range of motion greater than 35 degrees).^19^ As this trial sought to examine spinal manipulative therapies in a cohort with chronic back pain, none of the participants had symptom duration of less than 16 days. Interventions allowed during the trial were over-the-counter pain relief medications (eg, ibuprofen, acetaminophen) and application of heat or ice packs. However, narcotics, muscle relaxants, manipulation, mobilization, acupuncture, or massage treatments were prohibited.

Box. **Inclusion and Exclusion Criteria for Study Participation**

**Inclusion**

- Between 18 and 45 years of age
- Answer yes to the following questions:
  - Have you had low back pain constantly or on most days for the last 3 months?
  - Has your back pain caused you to seek care or consultation from a health care provider?
- Average pain intensity assessed using the Numerical Pain Rating Scale over the past week ≥2 on a 0-10 numerical pain scale
- Roland-Morris Disability Questionnaire score ≥4
- Exhibit at least 3 of the following findings reported in the clinical prediction rules for spinal manipulation:
  - No symptoms distal to the knee
  - Fear Avoidance Beliefs Questionnaire work subscale score <19
  - At least 1 hypomobile lumbar spinal segment
  - At least 1 hip with >35 degrees internal rotation range of motion

**Exclusion**

- Have a personal history of the following neurological disorders: Alzheimer disease, amyotrophic lateral sclerosis, multiple sclerosis, Parkinson disease, or stroke
- Have a history of the congestive heart failure or heart attack in past 24 months
- Have a body mass index greater than 35 kg/m^2^
- Have used narcotics or muscle relaxants within 30 days prior to study enrollment
- Have clinical depression (ie, score 24 or higher on the Center for Epidemiology Depression Scale)
- Report having pending litigation related to an episode of low back pain or are receiving any type of disability services related to low back pain
- Have active cancer
- Are blind
- Report being pregnant, lactating, or that they anticipate becoming pregnant in the next 3 months
- Current drug or alcohol use or dependence that, in the opinion of the primary investigators, would interfere with adherence to study requirements.

### Participant Recruitment

We used print and media advertisements, including mass mailing campaigns, to recruit a broad set of individuals with low back pain. This recruitment resulted in 4903 people completing a brief online survey to determine initial eligibility. In general, the vast majority of these potential participants were excluded owing to (1) insufficient pain levels, (2) insufficient duration of symptoms, (3) receiving prohibited interventions, or (4) age outside of the criterion. Individuals who met initial study criteria (n = 182) were then scheduled for an in-person physical screening prior to assignment to a treatment group. Our inclusion and exclusion criteria were designed to optimize the potential for observing an effect of manipulative intervention. Accordingly, a relatively homogenous group of individuals with chronic low back pain who were young and without obesity was enrolled (individuals with above class 1 [low-risk] obesity, defined by a body mass index [calculated as weight in kilograms divided by height in meters squared] of 30 to 34.9, were excluded). We postulate that these criteria minimized the confounding effects of aging and obesity and likely the comorbidities associated with these factors. Careful investigator bias control measures included the following: (1) study personnel conducting the outcome assessments were blind to group assignment throughout the length of the study, (2) clinicians were not aware that the cold laser was a sham intervention, and (3) the statistician and principal investigators remained blind to group assignment until data collection and analyses were completed. While participants could not be blinded to the assigned treatment group, they were unaware that the cold laser was inert (ie, placebo).

### Study Timeline

Eligible participants were enrolled into the study and randomized to an intervention group, baseline clinical measures were obtained, a treatment intervention was performed, and then the clinical measures were repeated (ie, immediately after the first treatment). Baseline measures to characterize the sample are provided in the Box. Participants then received 5 more treatments over the next 3 weeks. Prior to the second treatment intervention, participant expectations for treatment success were assessed using a credibility and expectancy questionnaire.^20^ The credibility and expectancy instrument consists of 7 questions with a 9-point Likert scale to assess how confident the participant is that the treatment they have received has the potential to reduce their symptoms. It is often used to assess the strength of the placebo treatment group in randomized trials. Three (±1) days after the final treatment, participants reported back to the laboratory for a follow-up assessment of all clinical measures (ie, primary end point). Participants returned approximately 4 weeks after their last treatment for a follow-up assessment of the clinical outcome measures.

### Study Interventions

#### Spinal Manipulation Treatment

The participant was placed in a side-lying position facing the clinician with the more painful side facing upward. The clinician passively flexed the participant’s hips and knees to induce lumbar spine flexion until they felt the spinous process of the affected lumbar vertebrae begin to move. Next, the clinician passively rotated the participant’s torso opposite to the side they were lying on until they felt rotation in the vertebra above the suspected lesion. The clinician applied a rapid thrust to the shoulder (anterior to posterior force) and pelvis (posterior to anterior force) resulting in a rotation force couple on the hypomobile segment. If a cavitation (ie, an audible pop) occurred, the treatment was considered complete. If no cavitation was produced, the participant was repositioned and the manipulation was attempted again. A maximum of 2 attempts per side was permitted. If no cavitation was produced after the 4 attempts (ie, 2 per side), the treatment was considered complete.

#### Spinal Mobilization Treatment

The participant was positioned using the same technique as described above. The clinician then placed 1 hand on the anterior shoulder and 1 hand on the posterior pelvis. The participant was asked to try to gently push the shoulder and pelvis into the clinician’s hands. Thus, they performed an isometric rotation of the torso resulting in a rotation force couple on the hypomobile segment. The clinician had the participant hold the contraction for a minimum of 7 seconds and a maximum of 60 seconds if the patient could comfortably tolerate the stretch. If a cavitation occurred or the clinician assessed a release in tissue tension, the treatment was considered complete. Otherwise, the clinician repositioned the patient as needed and repeated the treatment for up to 10 minutes.

#### Placebo (Sham Laser) Treatment

The laser manufacturer (MedX Health Corp) provided a MedX 1100 system that did not deliver any significant amount of light energy or heat but otherwise appeared operational to participants and clinicians. The sham laser was delivered over the painful region with the study participant positioned as described for spinal manipulation and spinal mobilization treatments.

#### Practitioners, Adherence, and Safety Assessments

Licensed clinicians (either a doctor of osteopathic medicine or physical therapist), with at least 3 years of clinical experience using manipulative therapies provided all treatments. A yearly workshop organized by the study team harmonized treatment interventions to ensure fidelity to the interventions described above. Successful adherence was defined as a study participant receiving at least 5 of the 6 treatments (83%). At each visit, all negative changes in health status were recorded as adverse events with attribution and grading determined based on the National Cancer Institute’s Common Terminology Criteria for Adverse Events.

### Statistical Analysis

Sample size calculations for this study have been previously described.^18^ In brief, based on extant studies of spinal manipulation, we used a Cohen *d* effect size of 0.5 for simulation studies that indicated 54 subjects per treatment group would be sufficient to achieve power of 0.8 to detect a clinically meaningful treatment effect for coprimary outcomes (ie, change in Numerical Pain Rating Scale [NPRS] score >2 and disability >3) at our primary end point. Sample size calculations also assumed a 10% attrition rate.

The primary hypotheses were that (1) the spinal manipulation and the spinal mobilization groups would display a significantly larger reduction in NPRS level from baseline than the placebo at the primary end point and (2) the spinal manipulation and the spinal mobilization groups would display a significantly larger reduction in Roland-Morris Disability Questionnaire score than the placebo at the primary end point. One-tailed tests were used to assess these directional hypotheses. A 2-point reduction in NPRS score^21^ and a 3-point reduction in Roland-Morris Disability Questionnaire score^22^ were considered clinically meaningful important differences. *P* < .05 was used to indicate statistical significance.

Data were examined to check for the following key assumptions: (1) normal distribution of random intercepts and slopes as well as residuals and (2) homogeneity of residual variances across the 3 treatment groups. Linear mixed-effects regression analyses served as the main analytic framework. This regression technique incorporates miscellaneous factors (eg, blocks and practitioners) as random effects so that the variances due to those factors can be partitioned. Furthermore, the maximum-likelihood estimation allows participants with missing data to be included in the model if the observations were missing completely at random or missing at random.

All regression models incorporated random intercept terms to accommodate the within-subject variance associated with longitudinal experimental designs and fixed-factor parameters for the main effects of group (sham-laser control as reference), main effect for time (baseline as reference), and the simple interaction terms of group by time (relative to control and baseline). Biological sex was included in each of the models. Additional a priori contrasts comparing changes from baseline between the 2 intervention groups (ie, simple interaction contrasts) were also performed, with Holm alpha adjustments to preserve the familywise error rates to 0.05.

#### Intention-to-Treat Analysis

All randomized study participants with at least a baseline assessment were included in the intention-to-treat (ITT) analysis. This trial enrolled and randomized participants following an in-person physical examination. Baseline measures were then completed in a follow-up visit within 7 days of the physical screening. Four of the randomized participants dropped out before baseline measures. Accordingly, our primary analysis does not meet the strict definition for ITT analysis. Thus, we used a near ITT analysis as the primary method for assessing outcomes, and maximum-likelihood estimation was used to estimate any missing observations.

#### Per-Protocol Analysis

For the per-protocol analysis (PPA), we excluded study participants who (1) attended fewer than 5 treatment sessions and (2) received prohibited concomitant interventions or (3) developed an exclusionary medical condition while on the study protocol. The outcomes from the ITT analysis and PPA were nearly identical (ie, differences in estimated marginal means were generally less than 0.5, with no differences in significance). Accordingly, only the results of the ITT analysis are presented.

## Results

A total of 162 participants (92 women [57%] and 70 men [43%]) with a mean (SD) age of 25.0 (6.2) years (range, 18.6-45.9 years) were enrolled in this trial. At baseline, this cohort reported a mean (SD) pain score over the last 7 days of 4.3 (2.5) on the NPRS (range, 0-10), a mean (SD) score of 9.9 (4.5) on the Roland-Morris Disability Questionnaire (range, 0-24), and mean (SD) length of chronic pain of 6.2 (5.1) years, indicating mild to moderate back pain of long duration. Fifty-four participants were randomized to the spinal manipulation group (31 women [57%] and 23 men [43%]), 54 participants to the spinal mobilization group (31 women [57%] and 23 men [43%]), and 54 participants to the placebo group (30 women [56%] and 24 men [44%]). Four participants were withdrawn from the study after the randomization process owing to scheduling issues (2 from the spinal manipulation group, 1 from spinal mobilization, and 1 from placebo) and never completed baseline measures or any part of the treatments and follow-up assessments. An additional 5 individuals did not complete the allocated intervention and follow-up visits. Participant characteristics as a function of treatment group are reported in Table 1.

**Table 1.** Baseline Participant Characteristics

Characteristic	Treatment group, mean (SD)^a^		
	Spinal manipulation	Spinal mobilization	Placebo (sham cold laser)
Sex, No. (%)			
Female	30 (59)	30 (58)	30 (58)
Male	21 (41)	22 (42)	22 (42)
Age, mean (SD), y	26.8 (7.2)	24.3 (5.3)	24.4 (5.9)
Race/ethnicity, No. (%)			
Hispanic/Latino	3 (5.5)	1 (1.8)	2 (3.7)
African American	2 (3.7)	5 (9.3)	6 (11.1)
Asian	2 (3.7)	4 (7.4)	1 (1.8)
White	46 (85.2)	41 (76.0)	44 (81.5)
>1 Race/ethnicity	2 (3.7)	2 (3.7)	3 (5.6)
Unreported	1 (1.8)	2 (3.7)	0
Body mass index, mean (SD)^b^	24.9 (4.35)	23.8 (3.28)	24.2 (4.1)
LBP duration, mean (SD), y	6.7 (5.6)	6.5 (5.6)	5.3 (4.1)
Depression, mean (SD) score^c^	17.1 (10.0)	19.0 (10.1)	15.9 (9.1)
Kinesiophobia, mean (SD) score^d^	37.9 (6.9)	34.6 (4.1)	36.7 (7.3)
Pain during last 7 d, mean (SD) score^e^	4.5 (2.7)	4.2 (2.4)	4.1 (2.4)
Disability, mean (SD) [range] score^f^	9.7 (4.5) [8.1-11.4]	10.1 (4.5) [8.5-11.7]	10.0 (4.5) [8.2-11.8]

Abbreviation: LBP, low back pain.

^a^ Four participants withdrew after randomization owing to scheduling issues (2 from the spinal manipulation group, 1 from spinal mobilization, and 1 from placebo) and did not complete baseline measures or any part of the treatments or follow-up assessments.

^b^ Calculated as weight in kilograms divided by height in meters squared.

^c^ Calculated using the Center for Epidemiologic Studies Depression Scale (scores range from 0 to 60, with higher scores indicating increased levels of depression).

^d^ Calculated using the Tampa Scale for Kinesiophobia (scores range from 17 to 68, with higher scores indicating greater levels of movement).

^e^ Calculated using the Numerical Pain Rating Scale (scores range from 0 to 10, with higher scores indicating greater pain).

^f^ Calculated using the Roland-Morris Disability Questionnaire (scores range from 0 to 24, with higher scores indicating greater disability).

### Primary Outcomes

**Figure 2.**
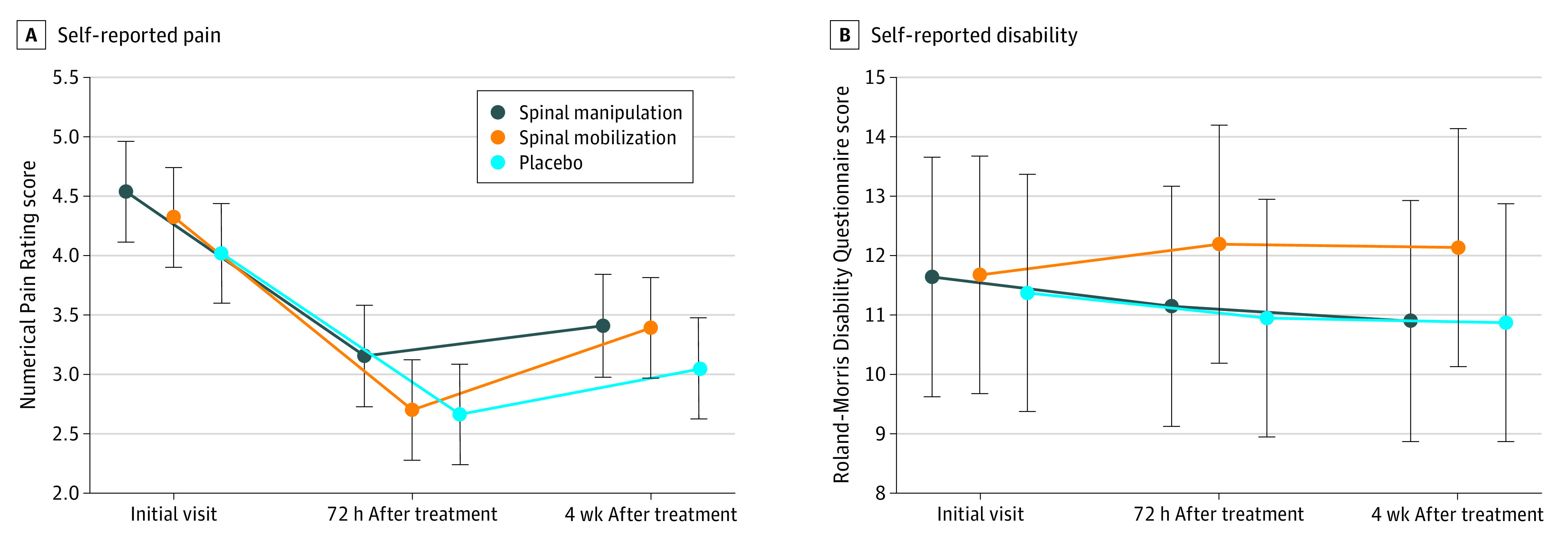
Change in Self-reported Pain and Disability Scores A, Self-reported assessment of average pain during the last 7 days (Numerical Pain Rating Scale score; range, 0-10, with higher scores indicating greater pain). B, Self-reported disability (Roland-Morris Disability Questionnaire; range, 0-24, with higher scores indicating greater disability). Means and 95% CIs (error bars) are plotted for ratings collected at the initial visit, 72 hours after completing 3 weeks of treatment (ie, primary end point), and 4 weeks after treatment completion (ie, follow-up). The plots are offset along the time axis for visual clarity.

At the primary end point, there was no significant difference in the change in pain scores between spinal manipulation and spinal mobilization (0.24; 95% CI, −0.38 to 0.86; *P* = .45), spinal manipulation and placebo (−0.03; 95% CI, −0.65 to 0.59; *P* = .92), or spinal mobilization and placebo (−0.26; 95% CI, −0.38 to 0.85; *P* = .39) (Figure 2A). There was no significant difference in change in self-reported disability scores between spinal manipulation and spinal mobilization (−1.00; 95% CI, −2.37 to 0.36; *P* = .14), spinal manipulation and placebo (−0.07; 95% CI, −1.43 to 1.29; *P* = .92), or spinal mobilization and placebo (0.93; 95% CI, −0.41 to 2.29; *P* = .17) (Figure 2B). There was no significant interaction of sex on pain or disability measures.

### Treatment Expectancy

A comparison of treatment credibility and expectancy ratings across groups was not statistically significant (*F*_2,151_ = 1.70, *P* = .19), indicating that, on average, participants in each group had similar expectations regarding the likely benefit of their assigned treatment. Next, we examined the relationship between these individual treatment expectancy ratings (Table 2), obtained before the second treatment session, and changes in current NPRS ratings (ie, 72 hours posttreatment − baseline). For the sample as a whole, treatment expectancy scores were inversely correlated with the change in pain ratings (*r* = −0.396; *P* < .01), indicating that those who had higher expectations of treatment success reported larger decreases in pain with treatment. When these expectancy effects were examined within each treatment group, significant inverse correlations were observed for placebo (*r* = −0.569; *P* < .001) and spinal manipulation (*r* = −0.423; *P* = .002) but not spinal mobilization (*r* = −0.188; *P* = .18). The changes in Roland-Morris Disability Questionnaire ratings (ie, session 9 − session 2) were not significantly related to treatment expectancy.

**Table 2.** Relationship Between Treatment Expectancy and Observed Pain Changes by Study Group

Treatment expectancy total score	Change from baseline to 72 h after treatment		Change from baseline to 4 wk after treatment	
	Pain right now, *r*	*P* value	Pain right now, *r*	*P* value
Spinal manipulation	−0.423	.002	−0.103	.49
Spinal mobilization	−0.188	.18	−0.112	.43
Placebo (sham cold laser)	−0.569	<.001	−0.471	<.001

## Discussion

The RELIEF Study is one of the few randomized clinical trials to date to compare the effectiveness of 2 common manipulative therapy techniques to a robust placebo for treatment of chronic low back pain. We found no effects of treatment group on the change in pain and disability immediately following a 3-week course of treatment or at the follow-up 4 weeks after treatment completion. Although we hypothesized that spinal manipulation and spinal mobilization would be more effective in reducing pain and disability compared with the placebo (ie, sham cold laser), this hypothesis was not supported by the data.

The clinical effectiveness of manipulative therapy techniques for acute and subacute low back pain has been reported^6,7,8^; however, the evidence on the effectiveness for reducing pain in chronic low back pain sufferers is mixed.^9,10,11^ We developed a modified version of a clinical prediction rule to identify individuals with chronic low back pain most likely to respond to a manipulative therapy intervention. Specifically, we modified the clinical prediction rule by Childs and colleagues^19^ by removing 1 of the 5 criteria (ie, symptoms <16 days) for this study. It should be noted that there were no data to indicate that acuteness of symptom onset was more heavily weighted than the 4 other criteria (ie, no symptoms distal to the knee, low fear avoidance behavior, at least 1 hypomobile vertebral segment, and at least 1 hip with internal range of motion greater than 35 degrees). Even with a modified clinical prediction rule to identify a cohort of individuals with chronic back pain most likely to respond to manipulative therapies, our data indicate that spinal manipulation and spinal mobilization were not very effective in reducing pain and disability in this cohort. This finding is consistent with those of Dougherty and colleagues,^23^ who also found that a modified version of this rule was not effective in discriminating which patients with chronic low back pain are most likely to respond to spinal manipulation. It is important to note that findings were reported by Dougherty and colleagues more than a year after subject recruitment began for the RELIEF Study.

There is also the possibility that manipulative therapy techniques are not as effective for treating mild to moderate pain conditions. Across treatment groups, participants in this study reported a mean pain score of 4.2 (on a 0-10 scale) when asked to rate their pain over the last 7 days. While our data show that manipulative therapy techniques were no more effective than an effective placebo treatment for mild to moderate chronic low back pain, it is possible that these techniques would be more effective for individuals with more severe pain. Alternatively, the finding that manipulative therapies were not very effective in a young cohort of patients with mild to moderate low back pain with few confounding factors may provide additional evidence that this approach is not ideal for chronic back pain. That possibility would be consistent with the literature that indicates manipulation is a reasonable, effective intervention for those with acute low back pain^6^ whereas the results are more mixed in chronic low back pain.^9,10,11^ Although the young age cohort in our study, which likely arose from our primary recruitment strategy being direct emails and online advertisements, is a limitation with respect to generalizability, a recent paper by Manogharan and colleagues^24^ suggests there are relatively small differences in baseline characteristics and prognosis among young, middle-aged, and older cohorts of patients with low back pain. In sum, these findings provide additional evidence about the limits of manipulative therapies in chronic low back pain.

Another consideration, not only in the RELIEF trial but in all studies on manipulative therapy, is the effectiveness of the placebo to create a true control condition. Whereas an effective placebo is critical to maintain blinding and reduce the risk of bias, poor placebos are quite common in low back pain studies.^25^ A review of low back pain trials by Machado and colleagues^25^ reported that 20% of trials had a placebo that was a potentially genuine treatment, 13% maintained adequacy of blinding, and most that assessed treatment expectations found that participants had lower expectations of placebo as compared to the active treatment groups.^25^ Another review reported that the quality of placebos typically used in studies on manipulative therapy is inadequate and that few studies controlled for or assessed treatment expectations.^26^ We used a validated treatment expectancy measure,^20^ and, consistent with Puhl et al,^26^ our data clearly showed that expectancy of treatment success was related to reductions in pain but not disability. Although there was no effect of treatment group on participant treatment expectancy ratings, the sham cold laser treatment group showed the strongest relationship between expectancy and pain reduction. Taken in total, these data suggest that sham cold laser is an effective placebo for spinal manipulation studies and that the power of participant expectations can be quite large. These findings reinforce the conclusions from Puhl et al^26^ that future trials should carefully select the control treatment, and the effectiveness of the control must be evaluated to provide confidence for assessing the effectiveness of manipulative therapy.

### Limitations

This study has limitations that should be noted. First, our participants were relatively young adults with only mild to moderate chronic low back pain with low levels of self-reported disability. Although this age cohort may not seek medical care for low back pain as often as middle-aged and older adults, it is worth noting the average duration of symptoms for this cohort was greater than 6 years. Thus, this is potentially an understudied cohort, and data on treatment effectiveness of manual therapies is needed. Second, the trial used a single-blinded design. However, this design is consistent with all studies of spinal manual therapies, as it is impossible to have a true double-blind design because the clinician will obviously know what treatment they are providing. We attempted to minimize any bias by having the data collection team assessing clinical outcomes blinded to treatment assignment. Another potential limitation is that the interventions were restricted to the isolated application of manipulation, mobilization, or placebo (ie, inert cold laser) using a classic randomized trial design. This approach was designed to reduce potential confounds by the application of concomitant interventions, such as stretching and strengthening exercises. Thus, a future study that uses a pragmatic clinical trial design approach could provide further understanding of the potential additive benefits of these manual therapies.

## Conclusions

Our findings indicated that spinal manipulation and spinal mobilization were no more effective than a well-chosen placebo in reducing pain and disability in patients with chronic low back pain. We conclude that these manipulative therapy techniques do not appear to be effective for chronic low back pain, at least among relatively young individuals with mild to moderate back pain.

## Notes

Supplement 1. **Trial Protocol**

Supplement 2. **Data Sharing Statement**
