# Supplementary material for: Effect of Spinal Manipulative and Mobilization Therapies in Young Adults With Mild to Moderate Chronic Low Back Pain: A Randomized Clinical Trial
Source: JAMA Netw Open. 2020 Aug 5;3(8):e2012589. doi: 10.1001/jamanetworkopen.2020.12589 (PMC7407093; doi:10.1001/jamanetworkopen.2020.12589)
Supplement: Supplement 1. — Trial Protocol [file jamanetwopen-3-e2012589-s001.pdf]

From: compliance@ohio.edu
To: Batey, Rebecca
Subject: LEO: IRB AMENDMENT 12-F-16 WAS APPROVED
Date: Friday, August 5, 2016 4:09:03 PM

Project Number	12-F-16
Project Status	APPROVED
Committee:	Biomedical IRB
Compliance Contact:	Robin Stack (stack@ohio.edu)
Primary Investigator:	James Thomas
Project Title:	The RELIEF Study - Researching the Effectiveness of Lumbar Interventions to Enhance Function
Level of Review:	FULL

The Biomedical IRB reviewed and approved your amendment of the above referenced research.

IRB Approved:	08/05/2016 4:09:01 PM
Expiration:	12/01/2016

Waivers: N/A

If applicable, informed consent (and HIPAA research authorization) must be obtained from subjects or their legally authorized representatives and documented prior to research involvement. In addition, FERPA, PPRA, and other authorizations must be obtained, if needed. The IRB-approved consent form and process must be used. Any changes in the research (e.g., recruitment procedures, advertisements, enrollment numbers, etc.) or informed consent process must be approved by the IRB before they are implemented (except where necessary to eliminate apparent immediate hazards to subjects).

The approval will no longer be in effect on the date listed above as the IRB expiration date. A Periodic Review application must be approved within this interval to avoid expiration of the IRB approval and cessation of all research activities. All records relating to the research (including signed consent forms) must be retained and available for audit for at least three (3) years after the research has ended.

It is the responsibility of all investigators and research staff to promptly report to the Office of Research Compliance / IRB any serious, unexpected and related adverse and potential unanticipated problems involving risks to subjects or others.

This approval is issued under the Ohio University OHRP Federalwide Assurance #00000095. Please feel free to contact the Office of Research Compliance staff contact listed above with any questions or concerns.

Research Compliance
117 Research and Technology Center 740.593.0664
compliance@ohio.edu

From: compliance@ohio.edu
To: Batey, Rebecca
Subject: LEO: IRB AMENDMENT 12-F-16 WAS APPROVED
Date: Wednesday, August 17, 2016 3:33:30 PM

LEO V3

Project Number	12-F-16
Project Status	APPROVED
Committee:	Biomedical IRB
Compliance Contact:	Robin Stack (stack@ohio.edu)
Primary Investigator:	James Thomas
Project Title:	The RELIEF Study - Researching the Effectiveness of Lumbar Interventions to Enhance Function
Level of Review:	FULL

The Biomedical IRB reviewed and approved your amendment of the above referenced research.

IRB Approved:	08/17/2016 3:33:27 PM
Expiration:	12/01/2016

Waivers: N/A

If applicable, informed consent (and HIPAA research authorization) must be obtained from subjects or their legally authorized representatives and documented prior to research involvement. In addition, FERPA, PPRA, and other authorizations must be obtained, if needed. The IRB-approved consent form and process must be used. Any changes in the research (e.g., recruitment procedures, advertisements, enrollment numbers, etc.) or informed consent process must be approved by the IRB before they are implemented (except where necessary to eliminate apparent immediate hazards to subjects).

The approval will no longer be in effect on the date listed above as the IRB expiration date. A Periodic Review application must be approved within this interval to avoid expiration of the IRB approval and cessation of all research activities. All records relating to the research (including signed consent forms) must be retained and available for audit for at least three (3) years after the research has ended.

It is the responsibility of all investigators and research staff to promptly report to the Office of Research Compliance / IRB any serious, unexpected and related adverse and potential unanticipated problems involving risks to subjects or others.

This approval is issued under the Ohio University OHRP Federalwide Assurance #00000095. Please feel free to contact the Office of Research Compliance staff contact listed above with any questions or concerns.

From: compliance@ohio.edu
To: Batey, Rebecca
Subject: LEO: IRB AMENDMENT 12-F-16 WAS APPROVED
Date: Friday, August 19, 2016 4:50:23 PM

LEO V3

Project Number	12-F-16
Project Status	APPROVED
Committee:	Biomedical IRB
Compliance Contact:	Robin Stack (stack@ohio.edu)
Primary Investigator:	James Thomas
Project Title:	The RELIEF Study - Researching the Effectiveness of Lumbar Interventions to Enhance Function
Level of Review:	FULL

The Biomedical IRB reviewed and approved your amendment of the above referenced research.

IRB Approved:	08/19/2016 4:50:21 PM
Expiration:	12/01/2016

Waivers: N/A

If applicable, informed consent (and HIPAA research authorization) must be obtained from subjects or their legally authorized representatives and documented prior to research involvement. In addition, FERPA, PPRA, and other authorizations must be obtained, if needed. The IRB-approved consent form and process must be used. Any changes in the research (e.g., recruitment procedures, advertisements, enrollment numbers, etc.) or informed consent process must be approved by the IRB before they are implemented (except where necessary to eliminate apparent immediate hazards to subjects).

The approval will no longer be in effect on the date listed above as the IRB expiration date. A Periodic Review application must be approved within this interval to avoid expiration of the IRB approval and cessation of all research activities. All records relating to the research (including signed consent forms) must be retained and available for audit for at least three (3) years after the research has ended.

It is the responsibility of all investigators and research staff to promptly report to the Office of Research Compliance / IRB any serious, unexpected and related adverse and potential unanticipated problems involving risks to subjects or others.

This approval is issued under the Ohio University OHRP Federalwide Assurance #00000095. Please feel free to contact the Office of Research Compliance staff contact listed above with any questions or concerns.

Research Compliance
117 Research and Technology Center 740.593.0664
compliance@ohio.edu

From: compliance@ohio.edu
To: Batey, Rebecca
Subject: LEO: IRB PERIODIC REVIEW 12-F-16 WAS APPROVED
Date: Monday, November 21, 2016 7:25:47 AM

LEO V3

Project Number	12-F-16
Project Status	APPROVED
Committee:	Biomedical IRB
Compliance Contact:	Robin Stack (stack@ohio.edu)
Primary Investigator:	James Thomas
Project Title:	The RELIEF Study - Researching the Effectiveness of Lumbar Interventions to Enhance Function
Level of Review:	FULL

The Biomedical IRB reviewed and approved your periodic review of the above referenced research.

IRB Approved:	11/21/2016 07:25:45 AM
Expiration:	11/03/2017

Waivers: N/A

If applicable, informed consent (and HIPAA research authorization) must be obtained from subjects or their legally authorized representatives and documented prior to research involvement. In addition, FERPA, PPRA, and other authorizations must be obtained, if needed. The IRB-approved consent form and process must be used. Any changes in the research (e.g., recruitment procedures, advertisements, enrollment numbers, etc.) or informed consent process must be approved by the IRB before they are implemented (except where necessary to eliminate apparent immediate hazards to subjects).

The approval will no longer be in effect on the date listed above as the IRB expiration date. A Periodic Review application must be approved within this interval to avoid expiration of the IRB approval and cessation of all research activities. All records relating to the research (including signed consent forms) must be retained and available for audit for at least three (3) years after the research has ended.

It is the responsibility of all investigators and research staff to promptly report to the Office of Research Compliance / IRB any serious, unexpected and related adverse and potential unanticipated problems involving risks to subjects or others.

This approval is issued under the Ohio University OHRP Federalwide Assurance #00000095. Please feel free to contact the Office of Research Compliance staff contact listed above with any questions or concerns.

Research Compliance
117 Research and Technology Center 740.593.0664
compliance@ohio.edu

Project Number	12-F-16
Project Status	APPROVED
Committee:	Biomedical IRB
Compliance Contact:	Robin Stack (stack@ohio.edu)
Primary Investigator:	James Thomas
Project Title:	The RELIEF Study - Researching the Effectiveness of Lumbar Interventions to Enhance Function
Level of Review:	FULL

The Biomedical IRB reviewed and approved the above referenced research.

IRB Approved:	02/14/2017 2:51:00 PM
Expiration:	11/03/2017

Waivers: None

If applicable, informed consent (and HIPAA research authorization) must be obtained from subjects or their legally authorized representatives and documented prior to research involvement. In addition, FERPA, PPRA, and other authorizations must be obtained, if needed. The IRB-approved consent form and process must be used. Any changes in the research (e.g., recruitment procedures, advertisements, enrollment numbers, etc.) or informed consent process must be approved by the IRB before they are implemented (except where necessary to eliminate apparent immediate hazards to subjects).

The approval will no longer be in effect on the date listed above as the IRB expiration date. A Periodic Review application must be approved within this interval to avoid expiration of the IRB approval and cessation of all research activities. All records relating to the research (including signed consent forms) must be retained and available for audit for at least three (3) years after the research has ended.

It is the responsibility of all investigators and research staff to promptly report to the Office of Research Compliance / IRB any serious, unexpected and related adverse and potential unanticipated problems involving risks to subjects or others.

This approval is issued under the Ohio University OHRP Federalwide Assurance #00000095. Please feel free to contact the Office of Research Compliance staff contact listed above with any questions or concerns.

From: [Thomas, James]
To: [Batey, Rebecca]
Subject: FW: NCCIH approval of revised protocol (version 6.0) - RELIEF Gender Supplemental - revised protocol (Thomas/Clark R01AT006978)
Date: Tuesday, July 5, 2016 6:58:28 AM
Attachments: image003.png
image004.png

From: Khalsa, Partap (NIH/NCCIH) [E] [mailto:khalsap@mail.nih.gov]
Sent: Thursday, June 30, 2016 5:32 PM
To: Thomas, James; Clark, Brian
Cc: NCCIH OCRA; Young, Cornice (NIH/NCCIH) [E]
Subject: NCCIH approval of revised protocol (version 6.0) - RELIEF Gender Supplemental - revised protocol (Thomas/Clark R01AT006978)

Dear Drs. Thomas and Clark,

Based on the clarification provided by Dr. Clark (see below), NCCIH is approving the revised protocol (version 6.0) for the parent study and ancillary study (funded by the ORWH supplement). Please keep a copy of this communique for the study's regulatory binder. Once the study has received IRB approval for the revised protocol, then please so inform NCCIH of said approval.

Regards,

Partap

Partap S. Khalsa, DC, PhD, DABCO
Deputy Director, Division of Extramural Research
National Center for Complementary and Integrative Health (NCCIH)
National Institutes of Health (NIH)
6707 Democracy Boulevard, Suite 401
Bethesda, MD 20892-5475
Office: (301) 594-3462; Fax: (301) 480-1587
Mobile: (301) 768-5173
Web: nccih.nih.gov
NIH National Center for Complementary and Integrative Health

From: Thomas, James [mailto:thomasj5@ohio.edu]
Sent: Thursday, June 30, 2016 7:41 AM
To: Khalsa, Partap (NIH/NCCIH) [E] <khalsap@mail.nih.gov>
Subject: RE: RELIEF Gender Supplemental - revised protocol [version 6.0] (Thomas/Clark R01AT006978) - UPDATE

Partap,
Dynamic trunk stiffness can be determined from the data collected on all participants in Group 2 (Spinal Effects) as the data necessary for these calculations were collected as part of the approved protocol, but this analysis **was not** explicitly proposed in the original DSP. The Co-I on gender supplemental, Dr. Peter Pidcoe, is the bioengineer who designed the puller apparatus used to deliver the trunk perturbations. He will now assist in developing the software necessary to derive dynamic trunk stiffness from the data already collected.

Please let me know if you need any additional information or clarification on this issue.

Regards,

Jim

UPDATE: Had an error in the first response ("was" should have been "was not"). Sent the first reply too early in the morning.

From: Khalsa, Partap (NIH/NCCIH) [E] [mailto:khalsap@mail.nih.gov]
Sent: Wednesday, June 29, 2016 4:57 PM
To: Thomas, James
Subject: FW: RELIEF Gender Supplemental - revised protocol [version 6.0] (Thomas/Clark R01AT006978)

Dear Jim,

As part of NCCIH's review of the revised study protocol (including the new supplement), would you please clarify for NCCIH the answer to the following question:

It appears that a new measure (i.e., Dynamic Trunk Stiffness) has been added to the protocol.

6.2.4 Follow-up Treatment Interventions and Assessments

Treatment Intervention 1 (visit 2)

- NPR (Assessed following treatment)
- Treatment Administration Form

The following physiological outcomes will be obtained immediately following the first treatment intervention based on the experimental group to which the participant was assigned to (i.e., muscular, spinal, cortical)

- Group 1 (Muscular Effects):
    - oSkeletal muscle proton transverse relaxation time (T2) of the lumbar muscles.
- Group 2 (Spinal Effects)
    - oShort-latency stretch reflex excitability.
    - oTrunk muscle onset latencies in response to postural perturbations.

oLumbar motion during volitional movement tasks

oDynamic trunk stiffness

As the study is almost complete for the LBP participants, how will Dynamic Trunk Stiffness be measured in the LBP participants in Group 2? Was it previously assessed (e.g., as part of Trunk muscle onset latencies in response to postural perturbations) but not included explicitly within the protocol, or will the study only obtain it on LBP participants from this point forward, and if so, how many LBP participants will this include?

Regards,

Partap

Partap S. Khalsa, DC, PhD, DABCO
Deputy Director, Division of Extramural Research
National Center for Complementary and Integrative Health (NCCIH)
National Institutes of Health (NIH)
6707 Democracy Boulevard, Suite 401
Bethesda, MD 20892-5475
Office: (301) 594-3462; Fax: (301) 480-1587
Mobile: (301) 768-5173
Web: nccih.nih.gov
NIH National Center for Complementary and Integrative Health

From: Thomas, James [mailto:thomasj5@ohio.edu]
Sent: Wednesday, June 22, 2016 4:31 PM
To: Khalsa, Partap (NIH/NCCIH) [E] <khalsap@mail.nih.gov>
Cc: Batey, Rebecca <batey@ohio.edu>
Subject: RELIEF Gender Supplemental

Dear Partap,
Per our conversation last week, we have modified the DSP, e-crfs, ICF, and advertising flier to incorporate the changes associated with the funded gender supplemental. We took a mixed approach to update the DSP. That is we incorporated changes within the document where necessary, but also put a significant portion of the proposed new experiments in an appendix. The changes to the main document are highlighted in blue to ease the burden on the reviewers. Our intent is submit these changes to OU IRB as soon as we have approval from NCCIH. Please let me know if you require any additional information on this document.

Regards,

Jim Thomas

++

James S. Thomas, PT, PhD
Professor & Director of Research
Division of Physical Therapy
Ohio University
W-281 Grover Center
Athens, Ohio 45701
Office Phone: 740-593-4178
Fax: 740-593-0293
www.ohio.edu/motorcontrol
www.ohio.edu/RELIEF
www.ohio.edu/backpain

++

**From:**	Khalsa, Partap (NIH/NCCIH) [E]
**To:**	Thomas, James
**Cc:**	Batey, Rebecca; Clark, Brian
**Subject:**	RE: RELIEF Gender Supplemental, revised protocol [version 6.0] (Thomas/Clark R01AT006978)
**Date:**	Wednesday, June 22, 2016 5:03:18 PM
**Attachments:**	image002.png

Dear Jim,

This is to acknowledge receipt of the revised study protocol (version 6.0) that incorporates changes related to the ORWH administrative supplement. I have initiated the NCCIH review of the revised protocol and anticipate responding to you within 3 – 4 weeks from now.

Regards,

Partap

Partap S. Khalsa, DC, PhD, DABCO
Deputy Director, Division of Extramural Research
National Center for Complementary and Integrative Health (NCCIH)
National Institutes of Health (NIH)
6707 Democracy Boulevard, Suite 401
Bethesda, MD 20892-5475
Office: (301) 594-3462; Fax: (301) 480-1587
Mobile: (301) 768-5173
Web: nccih.nih.gov
NIH National Center for Complementary and Integrative Health

From: Thomas, James [mailto:thomasj5@ohio.edu]
Sent: Wednesday, June 22, 2016 4:31 PM
To: Khalsa, Partap (NIH/NCCIH) [E] <khalsap@mail.nih.gov>
Cc: Batey, Rebecca <batey@ohio.edu>
Subject: RELIEF Gender Supplemental

Dear Partap,
Per our conversation last week, we have modified the DSP, e-crfs, ICF, and advertising flier to incorporate the changes associated with the funded gender supplemental. We took a mixed approach to update the DSP. That is we incorporated changes within the document where necessary, but also put a significant portion of the proposed new experiments in an appendix. The changes to the main document are highlighted in blue to ease the burden on the reviewers. Our intent is submit these changes to OU IRB as soon as we have approval from NCCIH. Please let me know if you require any additional information on this document.

Regards,

Jim Thomas

+++

James S. Thomas, PT, PhD
Professor & Director of Research
Division of Physical Therapy
Ohio University
W-281 Grover Center
Athens, Ohio 45701
Office Phone: 740-593-4178
Fax: 740-593-0293
www.ohio.edu/motorcontrol
www.ohio.edu/RELIEF
www.ohio.edu/backpain

+++

THE RELIEF STUDY

RESEARCHING THE EFFECTIVENESS OF LUMBAR INTERVENTIONS TO ENHANCE FUNCTION

DETAILED STUDY PROTOCOL

Final Version 6.0 dated 19 August 2016 4:50:21 PM

PRINCIPAL INVESTIGATORS:

James S. Thomas, P.T., Ph.D.
Professor, School of Rehabilitation and Communication Sciences and the Ohio Musculoskeletal and Neurological Institute, Ohio University
740-593-4178 (W); 740-591-1563 (C); thomasj5@ohio.edu

Brian C. Clark, Ph.D.
Professor, Department of Biomedical Sciences and the Ohio Musculoskeletal and Neurological Institute, Ohio University
740-593-2354 (W); 315-751-8732 (C); clarkb2@ohio.edu

SUPPORTED BY:
The National Center of Complementary and Integrative Health

NIH R01AT006978-01

CONFIDENTIAL
The information in The RELIEF Study Case Report Forms document is intended for official use The RELIEF Study team members and The RELIEF Study funding agencies and monitoring boards. Information in this document is confidential and should not be distributed to unauthorized individuals.

Protocol Revision History

Version Number: DRAFT v01.1
Version Date: August 1, 2012
IRB Approval: June 8, 2012
Summary of Revisions Made:

1. Initial protocol developed.

Version Number: DRAFT 0sign1.4
Version Date: 25 November 2012
IRB Approval: 5 December 2012
Summary of Revisions Made:

1. Provided methodological and administrative details associated with the mechanistic component of the study.
2. Defined the populations to be used in the intent to treat and per protocol analyses, and refined the planned statistical analyses.
3. Added Fisher's LSD to RCT statistical analyses
4. Made numerous minor changes to study surveys and questionnaires. These changes were made to ensure that all data is obtained in a streamlined and efficient manner. No substantial changes to the study design or outcome measures were made.
5. Altered the randomization protocol to assign study participants to one of three treatment groups with the assignment to the physiological groups based on subject scheduling and laboratory availability.

Version Number: FINAL 1.0
Version Date: 18 March 2013 (initial approval),
9 September 2013 (Amendment 3)
18 March 2014 (periodic review renewal)
IRB Approval: 8 May 2013 (initial approval)
16 September 2013 (amendment 3)
3 April 2014 (periodic review renewal)
Summary of Revisions Made:

1. Administrative changes: Minor changes involving grammar, wordsmithing, punctuation, and other editorial changes have been made throughout the document. All are clearly identified in the track-changes version of the amendment.
2. Based on conversation with NIH program staff, the randomization plan proposed in version 01.4 has been revised. Specifically, we now propose that study participants will be recruited and enrolled into one of three separate physiologic experiments (i.e., three separate studies that will assess the 1) muscular, 2) spinal, and 3) cortical effects of the respective interventions). Then, within each of these experiments, study participants will be randomly assigned to one of three treatment arms (i.e., spinal manipulation, spinal mobilization, or sham laser therapy).
3. We updated the detailed study protocol to reflect the inclusion of a 'Delegation of Authority Log' that will be used to denote specific individuals who are authorized by the PI's to participate in selected aspects of the study. We also updated section 10.1 (data management) to reflect a more specific data management plan that involves a combination of maintaining written records as well as an electronic database.
4. Additional details were added describing how we will allocate individuals to a given experiment in instances where multiple experiments are actively enrolling study participants and a participant meets eligibility requirements for more than one experiment.

5. Adverse Event Reporting – Sections 7.1-7.4: Overall changes to align the protocol with the Data and Safety Monitoring Plan as officially requested in the “action item” section of the NCCIH monitor’s site initiation visit report. Revised reporting times to align with Data and Safety Monitoring Plan and to meet specific requirements of various overseeing organizations. Added CTCAE attribution and severity scales as recommended by the IMC. Clarified procedure for reassessing health status at each visit.
6. Quality Assurance – Section 10.1: Additional clarification regarding collection forms, their handling, and quality assurance checks. Section 10.3.3: A new section added regarding protocol compliance and monitoring.
7. Updated Subject Flow Chart to reflect changes in experiments and allocation.
8. Addition of Co-Medical Director Dr. Timothy Law, D.O., per request of IRB.

Version Number: FINAL 2.0
Version Date: 5 June 2014 (initial approval)
6 March 2015 (periodic review renewal)
IRB Approval: 10 June 2014 (initial approval)
12 March 2015 (periodic review renewal)
Summary of Revisions Made

1. Administrative changes: Minor changes involving grammar, wordsmithing, punctuation, and other editorial changes have been made throughout the document. All are clearly identified in the track-changes version of the amendment.
2. Changed “treatment” to “intervention” in the study flow chart to comply with IRB requests.
3. Added Research Assistant to the list of study staff performing data entry in Section 10.1
4. Revised inclusion/exclusion “spine or pathologic fractures” to “pathologic fractures of the spine”
5. Clarified the exclusion criteria language “Report having received any manual therapy intervention in the past 1-month” to exclude only those therapies applied to the back and include reference to the first study intervention and to state the timeframe in more measurable terms (30 days).
6. Changed migraine headache TMS lab testing exclusion to stipulate 6-months prior to V2 lab testing.
7. Changed time between V7 (final intervention) and V8 from “48-hours” or “2 days” to “3-days +/-1-day” and time between V7 and V9 (physiologic testing) from 4-weeks to “28-days +/- 4-days” to allow for more flexibility in scheduling.
8. Added end of study notification to informed consent protocol.
9. Changed the date of randomization to visit 2 to ensure nobody is randomized to a treatment arm who is unable to participate in active protocol. For example, a participant may pass Visit 1 screening and therefore be enrolled, but realize they are unable to attend all of the appointments.
10. Added procedures for re-screening.
11. Changed the medical director to Dr. Tim Law, D.O.. Dr. Law was also added to the treatment provider team, and replaced Dr. Walkowski on all applicable committees.
12. Removed Dr. Soroka, treatment provider, from the Roster.
13. Specifications of Safety Parameters (7.1) was updated to reflect the revisions of the Health Status Reassessment forms.
14. Changed placement of Expectancy & Credibility Questionnaire to following treatment 1 instead of treatment 2
15. Added The ID Migraine™ self-administered screener to protocol for determining migraine headache, a contraindication for TMS testing.

Version Number: FINAL 3.0
Version Date: 20 April 2015
IRB Approval: 12 March 2015
Summary of Revisions Made

1. Administrative changes: Minor changes involving grammar, wordsmithing, punctuation, and other editorial changes have been made throughout the document. All are clearly identified in the track-changes version of the amendment.
2. Transition from paper-based case report forms to electronic/online forms.

Version Number: FINAL 4.0
Version Date: 28 August 2015
IRB Approval: 28 Septemeber 2015 (Periodic Review and Amendment)
4 November 2015 (Amendment)
2 December 2015 (Periodic Review)
Summary of Revisions Made

1. Administrative changes: Minor changes involving grammar, wordsmithing, punctuation, and other editorial changes have been made throughout the document. All are clearly identified in the track-changes version of the amendment.
2. Exclusion criteria added – positive pregnancy test excludes participants from MRI
3. Incidental findings procedures added to MRI testing
4. The “National Center for Complementary and Alternative Medicine” (NCCAM) has been updated to reflect the name change to “National Center for Complementary and Integrative Health” (NCCIH)

Version Number: FINAL 5.0
Version Date: 21 April 2016
IRB Approval: 15 June 2016 2:51:09 PM
Summary of Revisions Made

1. Administrative changes: Minor changes involving grammar, wordsmithing, punctuation, and other editorial changes have been made throughout the document. All are clearly identified in the track-changes version of the amendment.
2. Additional questions added to prescreen questionnaire. Some lab experiment groups are closing due to reaching goal capacity. Certain health history factors will exclude some participants from specific lab groups.
3. Addition of ResearchMatch.org as a recruitment tool.
4. Addition of “Back Pain?” advertising tool.
5. Addition of study staff signature line to informed consent form.

Version Number: FINAL 6.0
Version Date: 22 June 2016
NIH Approval: 30 June 2016
IRB Approval: 19 August 2016
Summary of Revisions Made

1. Administrative changes: Minor changes involving grammar, wordsmithing, punctuation, and other editorial changes have been made throughout the document. All are clearly identified in the track-changes version of the amendment.
2. Addition of supplemental research.

Table of Contents

Protocol Revision History 1

STUDY TEAM ROSTER 7

PARTICIPATING STUDY SITES 7

PRÉCIS 8

1. STUDY OBJECTIVES 11

1.1 Mechanistic Component 11

1.2 RCT Component 12

2. BACKGROUND AND RATIONALE 13

2.1 Background on Low Back Pain and Manual Therapy Treatments 13

2.2 Study Rationale 13

3. STUDY DESIGN 16

4. SELECTION AND ENROLLMENT OF PARTICIPANTS 21

4.1 Inclusion Criteria 21

4.2 Exclusion Criteria 21

4.3 Study Enrollment Procedures 22

5. STUDY INTERVENTIONS 24

5.1 Interventions and Administration 24

5.2 Handling of Study Interventions 25

5.3 Concomitant Interventions 25

5.3.1 Allowed Interventions 25

5.3.2 Required Interventions 25

5.3.3 Prohibited Interventions 25

5.4 Adherence Assessment 25

6. STUDY PROCEDURES 27

6.1 Schedule of Evaluations. 27

6.2 Description of Evaluations 28

6.2.1 Screening Evaluation 28

6.2.2 Enrollment, Randomization and Baseline 29

6.2.3 Blinding 33

6.2.4 Follow-up Treatment Interventions and Assessments 34

7. SAFETY ASSESSMENTS **35**
7.1 Specification of Safety Parameters 35
7.2 Assessing, Recording, and Analyzing Safety Parameters 36
7.3 Adverse Events and Serious Adverse Events 36
7.4 Reporting Procedures 38
7.5 Follow-up for Adverse Events 38
7.6 Safety Monitoring 38
8. INTERVENTION DISCONTINUATION **39**
9. STATISTICAL CONSIDERATIONS **40**
10. DATA COLLECTION AND QUALITY ASSURANCE **49**
10.1 Data Collection Forms 49
10.2 Data Management 49
10.3 Quality Assurance 49
10.3.1 Training 49
10.3.2 Quality Control Committee 49
10.3.3 Protocol Compliance 49
10.3.3 Metrics 50
10.3.4 Protocol Deviations 50
10.3.5 Monitoring 50
11. PARTICIPANT RIGHTS AND CONFIDENTIALITY **51**
11.1 Institutional Review Board (IRB) Review 51
11.2 Informed Consent Forms 51
11.3 Participant Confidentiality 51
11.4 Study Discontinuation 51
12. COMMITTEES **51**
13. PUBLICATION OF RESEARCH FINDINGS **53**
14. REFERENCES **54**
Appendix: Supplemental **57**
Specific Aims **57**
Specific Aim 1. 57
Specific Aim 2. 57
Specific Aim 3 57

Specific Aim 4. 57

Research Strategy 57

SIGNIFICANCE 58

INNOVATION 59

APPROACH 59

1. Rationale 59

2. Assessing Trunk Stiffness 60

NUMBER OF SUBJECTS AND POWER 62

SUBJECTS AND RECRUITMENT 62

Inclusion criteria 63

Exclusion criteria 63

Hypothesis Testing: Aim 1 64

Hypothesis Testing: Aim 2 64

Caveats and Considerations for Aims 1 & 2 64

Specific Aim 3. 64

Hypothesis 3.1 64

Hypothesis 3.2 64

Specific Aim 4 64

Hypothesis 4.1. 64

Timeline 65

Summary 65

References 66

STUDY TEAM ROSTER

Principal Investigators:

- James S. Thomas, P.T., Ph.D.
  - W277 Grover Center; 740-593-4178 (T); 740-593-0292 (F); thomasj5@ohio.edu
- Brian C. Clark, Ph.D.
  - 236 Irvine Hall; 740-593-2354 (T); 740-597-2778 (F) clarkb2@ohio.edu

The RELIEF Study Medical Director and Co-Medical Director:

- Timothy Law, D.O. – Medical Director
  - 244 Irvine Hall; 740-593-2233 (T); lawt@ohio.edu
- Stevan Walkowski, D.O. – Co-Medical Director
  - 248 Grosvenor Hall; 740-593-2231 (T); walkowsk@ohio.edu

The RELIEF Study Treatment Provider Team:

- Timothy Law, D.O.
  - 244 Irvine Hall; 740-593-2233 (T); Timothy.Law@empireblue.com
- James Odenthal, P.T.
  - W290 Grover Center; 740-593-4722 (T); odenthaj@ohio.edu
- David Russ, P.T., Ph.D.
  - W279 Grover Center; 740-566-0022 (T); russd@ohio.edu
- Betty Sindelar, P.T., Ph.D.
  - W295 Grover Center; 740-597-1883 (T); sindelar@ohio.edu
- Stevan Walkowski, D.O.
  - 248 Grosvenor Hall; 740-593-2231 (T); walkowsk@ohio.edu
- Timothy Law, D.O.
  - 244 Irvine Hall; 740-593-2233 (T); lawt@ohio.edu

The RELIEF Study Coordination and Data Collection Team

- An unblinded study coordinator and blinded research assistants assigned to the data collection team. Specific individuals and their respective roles are identified and tracked in The RELIEF Study 'Delegation of Authority Log' (see appendix).

Other Key Members of The RELIEF Study Team Roster

- Christopher France, Ph.D. (Psychologist & Clinical Outcomes Expert)
  - 251 Porter Hall; 740-593-1079 (T); france@ohio.edu
- Masato Nakazawa, Ph.D. (Biostatistician)
  - 236 Grosvenor Hall; 740-593-2336; nakazawa@ohio.edu
- Daniel Corcos, Ph.D. (Clinical Trial Design Expert)
  - University Illinois-Chicago, 312-355-1708 (T), dcorcos@uic.edu
- Guang Yue, Ph.D. (Magnetic Resonance Imaging Expert)
  - Kessler Foundation; 973-324-8362 (T); GYue@kesslerfoundation.org
- Peter Pidcoe, Ph.D. (Bioengineer)
  - Virginia Commonwealth University, 804-628-3655 (T); pepidcoe@vcu.edu

PARTICIPATING STUDY SITES

This is a single site study at Ohio University.

PRÉCIS

Study Title

The RELIEF Study: Researching the Effectiveness of Lumbar Interventions for Enhancing Function Study

Objectives

There are two major goals of this study. One is to determine the biological mechanisms of two manual therapy interventions commonly used in the treatment of chronic low back pain (hence forward referred to as the 'Mechanistic Component'). The second is to conduct a randomized clinical trial (RCT) to determine the effectiveness of these two manual therapies at reducing pain and disability in patients with chronic low back pain compared to each other as well as a sham control group (hence forward referred to as the 'RCT Component').

The supplemental study seeks to examine the effects of sex on 1) trunk stiffness in response to multi-directional seated perturbations in participants with chronic LBP compared to matched healthy controls and 2) changes in trunk stiffness in response to multi-directional seated perturbations in participants with chronic LBP following an initial spinal manipulative treatment and following a 3-week course of treatment.

Design and Outcomes

A single-blinded (investigator-blinded) sham-controlled study to test the mechanisms and effectiveness of two manual therapy techniques applied to individuals (18-45 years of age) with chronic low back pain. Specifically, the clinical outcome data from the mechanistic component will be pooled across experiments to permit an exploratory Phase II RCT investigating the effectiveness of manual therapies in treating chronic low back pain.

Participants with chronic low back pain will be recruited and enrolled into one of three separate experiments that constitute the mechanistic component to determine the muscular, spinal, and cortical effects of manual therapies. For this objective we seek to determine the acute effects of manual therapies as well as the effects following a course of treatment. Within each of these experiments study participants will be randomly assigned to one of the three treatment arms: 1) spinal manipulation, 2) spinal mobilization, or 3) sham Laser therapy. Treatments will be delivered twice per week for 3-weeks. The primary outcome variables for both components are provided below.

Participants with no low back pain will be recruited and enrolled into a modified spinal experiment group to determine sex differences in dynamic trunk stiffness in both a healthy and patient population.

<u>Mechanistic Component</u>

- Experiment 1 (Muscular Effects):
    - Skeletal muscle proton transverse relaxation time (T2) of the lumbar muscles.
- Experiment 2 (Spinal Effects):
    - Short-latency stretch reflex excitability.
    - Trunk muscle onset latencies in response to postural perturbations.
    - Lumbar motion during volitional movement tasks.

- Dynamic trunk stiffness
- Experiment 3 (Cortical Effects):
  - Motor evoked potential amplitude.
  - Intracortical facilitation.
  - Short-interval intracortical inhibition.

RCT Component

Co-Primary Outcomes:

- Change in Numerical Pain Rating (NPR) score for average over the last 7-days
- Change in Roland Morris Disability Questionnaire (RMDQ)

Secondary Outcomes:

- Change in PROMIS Pain Behavior
- Change in PROMIS Pain Interference
- Change in PROMIS Pain Intensity
- Change in PROMIS Physical Function
- Change in NPR scores for current pain, and worst and best pain in the last 7-days

Interventions and Duration

Three interventions will be compared: 1) spinal manipulation, 2) spinal mobilization, and 3) sham Laser therapy. The total length of time each participant will be on study (intervention period + additional follow-up off intervention) is 7 weeks (3 weeks of the intervention + 4 weeks of follow-up). The primary endpoint for the RCT Component will be following the 3-week course of treatment (visit 8). A detailed study diagram is provided in Section 3.

Duration for the supplemental research will be 3-hours including screening, baseline assessments, and lab testing.

Sample Size and Population

Target population: Forty-two patients with chronic low back pain will be enrolled into experiment 1 (Muscular Effects) and randomly assigned to the following 3 treatment arms (14 patients per arm): 1) spinal manipulation, 2) spinal mobilization, or 3) sham Laser. Sixty patients with chronic low back pain will be enrolled into experiment 2 (Spinal Effects) and randomly assigned to the same 3 treatment arms (20 patients per arm). Sixty patients with chronic low back pain will be enrolled into experiment 3 (Cortical Effects) and randomly assigned to the 3 treatment arms. The clinical outcome data from the individuals will be pooled across the three physiological experiments for the RCT component. This pooling will result in a total of 54 patients being assigned to one of the three treatment arms (total n=162 patients).

The supplemental research will recruit an additional 60 healthy participants with no history of LBP and who are matched on sex, age (± 3 years), height (± 2 inches), and weight (± 10 lbs) to the participants

recruited into Experiment 2 (Spinal Effects) (total n=222).

1. STUDY OBJECTIVES

There are two major goals of this study. One is to determine the biological mechanisms of two manual therapy interventions commonly used in the treatment of chronic low back pain (i.e., 'Mechanistic Component'). The second is to conduct a randomized clinical trial (RCT) to determine the effectiveness of these two manual therapies at reducing pain and disability in patients with chronic low back pain compared to each other as well as to a sham control group (i.e., 'RCT Component'). Accordingly, this study consists of three mechanistic experiments across which the clinical outcome variables will be pooled for an exploratory Phase II RCT investigating the effectiveness of manual therapies in treating chronic LBP.

1.1 Mechanistic Component

The primary objective of the mechanistic component is to identify the physiological and biomechanical bases for changes in pain and disability associated with the respective interventions. As stated previously, participants will be recruited and enrolled into three separate experiments and then randomly assigned to one of three treatment arms. Additionally, for this objective we seek to determine the 1) acute effects of manual therapies, 2) the effects following a 3-week course of treatment, and 3) the sustained effects assessed approximately 1 month after completing the course of treatment. Thus, for this objective we will include 4 assessment points (baseline plus the 3 time points described above) for our physiological outcome measures. Below we outline the primary physiological outcomes for each group.

- Group 1 (Muscular Effects): Subjects assigned to this group will undergo a magnetic resonance imaging examination to quantify the skeletal muscle proton transverse relaxation time (T2) of the lumbar muscles.

- Group 2 (Spinal Effects): Subjects assigned to this group will undergo neurophysiological and biomechanical examinations to quantify short-latency stretch reflex excitability, trunk muscle onset latencies in response to postural perturbations, and lumbar motion during volitional movement tasks.

- Group 3 (Cortical Effects): Subjects assigned to this experiment will undergo a transcranial magnetic stimulation protocol to quantify motor evoked potential amplitude, intracortical facilitation, and short-interval intracortical inhibition.

Collectively, data from these physiological outcome groups will test the central hypothesis that manual therapies—particularly spinal manipulation techniques— reduce spinal reflex gain (reflected by decreased short-latency reflex response), which in turn decreases reflexive muscle activity (i.e., spasm; reflected by diminished resting MRI T2 signal and surface EMG). The decreased muscle spasm leads to reduced pain, which should remove or reduce inhibitory effects arising from nociceptive afferents projecting to the motor cortex. This leads to an increase in motor cortex excitability (reflected by an increase in intracortical facilitation and/or decrease in intracortical inhibition). Ultimately, when pain and inhibitory input to the motor cortex are reduced, pain and disability decrease and volitional movement is improved. Additionally, the supplemental research data will test the hypothesis that male participants with chronic LBP will present with increased stiffness compared to female participants with chronic LBP, that participants with chronic LBP will present with increased stiffness compared to healthy controls, that there are no net sex effects of spinal manipulation on trunk stiffness, and both manipulative interventions will decrease passive trunk stiffness as evidenced by the trunk's initial mechanical response to multi-directional trunk perturbations.

1.2 RCT Component

The primary objective of this component is to determine whether individuals with chronic low back pain have greater reductions in disability and pain following one of the following interventions delivered for 3-weeks: 1) spinal manipulation, 2) spinal mobilization, or 3) sham Laser. Hypothesis: The manual therapy interventions will result in a 2 point greater reduction in pain and a 3 point greater reduction in disability when compared to the sham Laser intervention, with the spinal manipulation intervention demonstrating the greatest reduction in pain and disability of all three treatment interventions.

2. BACKGROUND AND RATIONALE

2.1 Background on Low Back Pain and Manual Therapy Treatments

Low back pain is one of the most common reasons for seeking medical care and accounts for over 3.7 million physician visits/year in the U.S. alone. Ninety percent of adults will experience low back pain in their lifetime, 50% will experience recurrent LBP, and 10% will develop chronic pain and related disability.[1-4] According to a recent national survey, more than 18 million Americans over the age of 18 years received manipulative therapies in 2007 at a total annual out of pocket cost of $3.9 billion.[5] Spinal manipulative treatments can be broadly classified as manipulation-based or mobilization-based techniques. Manipulation-based techniques (e.g., translatory thrust) apply a high-velocity, low-amplitude force to the spine and are often accompanied by an audible sound from one or more joints. In contrast, mobilization-based techniques (e.g., muscle energy) use a low-velocity, low-force approach that generally does not produce audible joint sounds. Recent evidence suggests that there are differences in the short-term clinical effects of manipulation-based versus mobilization-based spinal techniques.[6] Specifically, manipulation-based techniques result in greater reduction in pain and disability compared to mobilization-based techniques. While there is growing evidence for the clinical effectiveness of manipulative therapies to treat low back pain, little is known on the neurophysiologic consequences and effects of either manipulation-based or mobilization-based treatments.[7-11] Further, addition data is needed to understand how these different manual therapy techniques effect clinical changes in pain and disability. The lack of empirical data hinders acceptance by the wider scientific and health-care communities, and it also limits the development of rational strategies for using manipulative therapies.

2.2 Study Rationale

Background & Significance: Over the past decade there has been growing scientific evidence supporting the clinical effectiveness of manual therapy treatments for low back pain.[10-17] However, little scientific evidence has been offered to explain the effects and mechanisms underlying these treatments. Many scientists and clinicians have long postulated that spinal manipulation exerts its biologic effects on segmental components of the central nervous system.[18-33] It has been proposed that manual therapies act through a cascade of neurophysiologic responses [26, 34] to reduce muscle spasm and subsequent pain. Two general models of low back pain that have received the most attention (i.e., pain adaptation and pain-spasm-pain models) have been used to support these propositions.

The pain adaptation model posits a decrease in trunk agonist muscle activity and an increase in antagonist muscle activity that serves a protective function.[35] This model also predicts no difference in resting activity of the trunk muscles. In contrast, the pain-spasm-pain model of LBP (Figure 1) suggests that pain leads to muscular hyperactivity (spasm), which in turn causes pain.[36] While two distinct neural pathways have been proposed to support this model, the common theme is that increased excitatory input to the alpha-motorneuron pool leads to more sustained and intense muscle activity in LBP patients (for review see Van Dien et al., 2003) [37]. Spinal manipulation has been proposed to act *via* the pain-spasm-pain model, yet this has not been clearly demonstrated.

We recently reported that a single session of spinal manual therapy treatment—involving a combination of manipulation and mobilization techniques—reduces activation of trunk muscles, as determined by muscle functional magnetic resonance imaging [18]. Consistent with these findings, a few studies [38-41] and anecdotal reports [26, 42] have noted reduced paraspinal muscle activity, as determined by surface electromyography, following spinal manipulation on individuals with low back pain. Further, our preliminary data also indicate that spinal manipulation treatments reduce the amplitude of short-latency stretch reflexes, but only if an audible joint sound is produced [43]. While manipulative treatments could act *via* the pain-spasm-pain model by reducing the underlying nociceptive stimulus (hypomobile segment),[40, 42, 44-48] there are three primary limitations to these studies that make such conclusions tenuous. Specifically, these studies have:

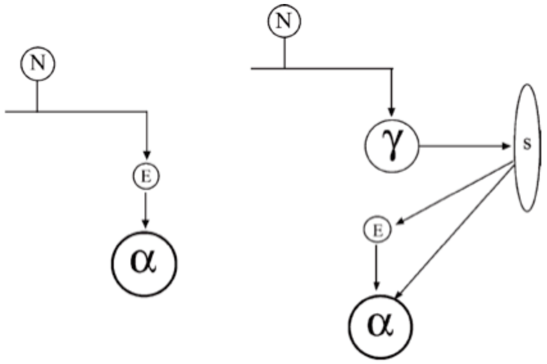

Figure 1. Two neural pathways suggested to form the basis of a pain-spasm-pain cycle. Left side: Nociceptive afferents (N) transmit feedback via excitatory interneurons (E) to the alpha motor neurons (α) that cause increased muscle activation (spasm). Right side: Nociceptive afferents (N) provide excitatory input on the gamma motor neurons (γ) that increase the sensitivity of the muscle spindles (s), which activate alpha motoneurons via excitatory interneurons (E) further increasing muscle activation and pain.
From Van Dien et al., J Electromyography Kinesiol, 2003.

- Not taken a comprehensive approach to determine the effects of manipulation on the various segmental components of the central nervous system (cortical and spinal) that could explain the reported reductions in muscle activity. Thus, the neurophysiologic mechanisms underlying spinal manipulative treatments remain largely unknown.

- Primarily focused on manipulation-based treatments and disregarded mobilization-based treatments. Thus, the physiologic effects of manipulation-based versus mobilization-based treatments are unknown.

- Not examined both the immediate physiologic effects, those following a course of treatment, as well the sustained effects (i.e., changes lasting after treatment has concluded).

The RELIEF Study will address all of these shortcomings. The Mechanistic Component will assess treatment-induced changes at the muscular, spinal, and cortical levels. Importantly, the RCT Component of the RELIEF Study will provide critical data on treatment effectiveness, which will also allow the examination of the relationship between the changes in the physiological outcomes and clinical outcomes on symptom resolution following treatment.

<u>Known and Potential Risks of the Treatment Interventions:</u> Spinal manual therapies are some of the most common treatments for low back pain, and the risk of serious injury from the lumbar spinal manual therapy techniques is reported to be extremely small [49]. Gouveia recently conducted a systematic review to examine the adverse reactions associated with manual therapies. Their search identified 46 articles that included data concerning adverse events. With regards to lumbar (i.e., not cervical) manipulation, most of the adverse events reported were benign and transitory [49]. They did however report some cases of serious adverse events, such as disc herniation and progression to cauda equina syndrome [49]. However, others have estimated of the incidence of serious adverse events from published case reports and case series to be extremely rare (i.e., about 1 case event per 1 to 2 million treatments) [50]. A more common risk is soreness, which generally dissipates within hours to days.

Rationale regarding the supplemental research is included in the Appendix.

3. STUDY DESIGN

The RELIEF Study is a single-blinded (investigator-blinded) sham-controlled study to test the mechanisms and effectiveness of two manual therapy techniques applied to individuals (18-45 years of age) with chronic low back pain. There are two major goals of this study. One is to determine the biological mechanisms of two manual therapy interventions commonly used in the treatment of chronic low back pain (i.e., 'Mechanistic Component'). The second is to conduct a randomized clinical trial (RCT) to determine the effectiveness of these two manual therapies at reducing pain and disability in patients with chronic low back pain compared to each other as well as to a sham control group (i.e., 'RCT Component'). Accordingly, this study consists of three mechanistic experiments across which the clinical outcome variables will be pooled for an exploratory Phase II RCT investigating the effectiveness of manual therapies in treating chronic LBP.

A total of 162 individuals with chronic low back pain and 60 participants without low back pain will participate in the RELIEF Study. Forty-two individuals will be recruited and enrolled into Experiment 1 (Muscular Effects), sixty individuals with low back pain and 60 with no low back pain will be recruited and enrolled into Experiment 2 (Spinal Effects), and sixty individuals will be recruited and enrolled into Experiment 3 (Cortical Effects). Within each of these separate experiments, participants with chronic low back pain will be randomly assigned to one of the three treatment arms: 1) spinal manipulation, 2) spinal mobilization, or 3) sham Laser therapy. The clinical outcome data will be pooled across the three physiological experiments resulting in 54 subjects/treatment arm. Treatments will be delivered twice per week for 3-weeks. The primary outcomes for the Mechanistic and RCT components of the RELIEF Study are provided below:

<u>Mechanistic Component</u>

- Group 1 (Muscular Effects):
  - Skeletal muscle proton transverse relaxation time (T2) of the lumbar muscles.
- Group 2 (Spinal Effects):
  - Short-latency stretch reflex excitability.
  - Trunk muscle onset latencies in response to postural perturbations.
  - Lumbar motion during volitional movement tasks.
  - Dynamic trunk stiffness
- Group 3 (Cortical Effects):
  - Motor evoked potential amplitude.
  - Intracortical facilitation.
  - Short-interval intracortical inhibition.

<u>RCT Component</u>

Co-Primary Outcomes:

- Change in Numerical Pain Rating (NPR) score over the last 7-days
- Change in Roland Morris Disability Questionnaire (RMDQ)

Secondary Outcomes:

- Change in PROMIS Pain Behavior
- Change in PROMIS Pain Interference

- Change in PROMIS Pain Intensity
- Change in PROMIS Physical Function
- Change in NPR scores for current pain, and worst and best pain in the last 7-days

More information regarding the supplemental research study design is included in the appendix.

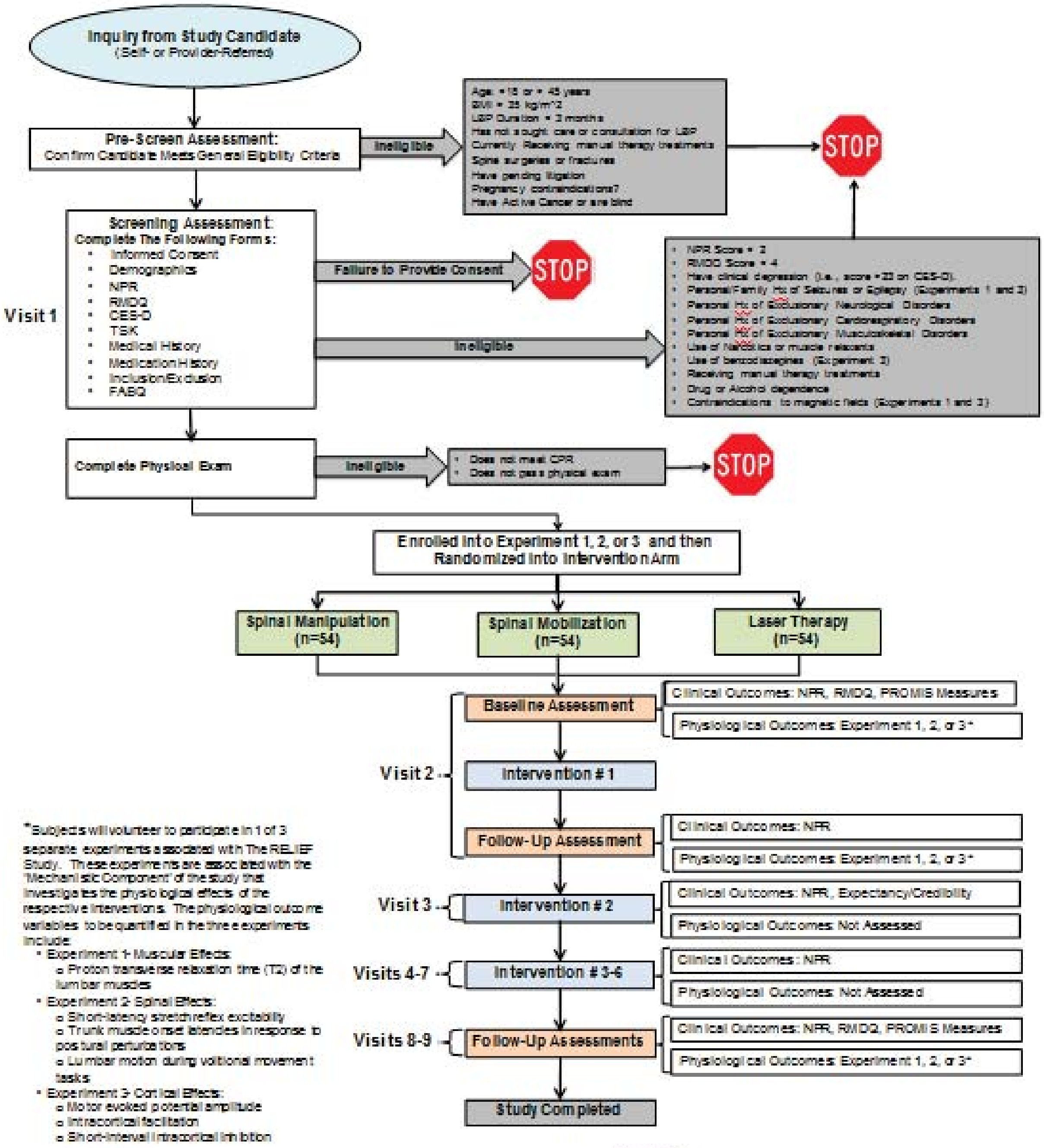
Inquiry from Study Candidate
(Self- or Provider-Referred)
Pre-Screen Assessment:
Confirm Candidate Meets General Eligibility Criteria
Ineligible
Age • 18 or • 45 years
BMI • 35 kg/m^2
LBP Duration • 3 months
Has not sought care or consultation for LBP
Currently Receiving manual therapy treatments
Spine surgeries or fractures
Have pending litigation
Pregnancy contraindications?
Have Active Cancer or are blind
STOP
Visit 1
Screening Assessment:
Complete The Following Forms:
Informed Consent
Demographics
NPR
RMDQ
CES-D
TSK
Medical History
Medication History
Inclusion/Exclusion
FABQ
Failure to Provide Consent
STOP
Ineligible
NPR Score • 2
RMDQ Score • 4
Have clinical depression (i.e., score •23 on CES-D).
Personal/Family Hx of Seizures or Epilepsy (Experiments 1 and 3)
Personal Hx of Exclusionary Neurological Disorders
Personal Hx of Exclusionary Cardiorespiratory Disorders
Personal Hx of Exclusionary Musculoskeletal Disorders
Use of Narcotics or muscle relaxants
Use of benzodiazepines (Experiment 3)
Receiving manual therapy treatments
Drug or Alcohol dependence
Contraindications to magnetic fields (Experiments 1 and 3)
Complete Physical Exam
Ineligible
Does not meet CPR
Does not pass physical exam
STOP
Enrolled into Experiment 1, 2, or 3 and then Randomized into Intervention Arm
Spinal Manipulation (n=54)
Spinal Mobilization (n=54)
Laser Therapy (n=54)
Visit 2
Baseline Assessment
Clinical Outcomes: NPR, RMDQ, PROMIS Measures
Physiological Outcomes: Experiment 1, 2, or 3*
Intervention #1
Follow-Up Assessment
Clinical Outcomes: NPR
Physiological Outcomes: Experiment 1, 2, or 3*
Visit 3
Intervention #2
Clinical Outcomes: NPR, Expectancy/Credibility
Physiological Outcomes: Not Assessed
Visits 4-7
Intervention #3-6
Clinical Outcomes: NPR
Physiological Outcomes: Not Assessed
Visits 8-9
Follow-Up Assessments
Clinical Outcomes: NPR, RMDQ, PROMIS Measures
Physiological Outcomes: Experiment 1, 2, or 3*
Study Completed
*Subjects will volunteer to participate in 1 of 3 separate experiments associated with The RELIEF Study. These experiments are associated with the "Mechanistic Component" of the study that investigates the physiological effects of the respective interventions. The physiological outcome variables to be quantified in the three experiments include:
Experiment 1- Muscular Effects:
Proton transverse relaxation time (T2) of the lumbar muscles
Experiment 2- Spinal Effects:
Short-latency stretch reflex excitability
Trunk muscle onset latencies in response to postural perturbations
Lumbar motion during volitional movement tasks
Experiment 3- Cortical Effects:
Motor evoked potential amplitude
Intracortical facilitation
Short-interval intracortical inhibition

<u>Target Populations and Study Groups</u>:

Forty-two patients with chronic low back pain will be enrolled into experiment 1 (Muscular Effects) and randomly assigned to the following 3 treatment arms (14 patients per arm): 1) spinal manipulation, 2) spinal mobilization, or 3) sham Laser. Sixty patients <u>with</u> chronic low back pain will be enrolled into experiment 2 (Spinal Effects) and randomly assigned to the following 3 treatment arms (20 patients per arm): 1) spinal manipulation, 2) spinal mobilization, or 3) sham Laser. Sixty participants <u>without</u> low back pain will be enrolled into a modified experiment 2 (Spinal Effects). Sixty patients with chronic low back pain will be enrolled into experiment 3 (Cortical Effects) and randomly assigned to the following 3 treatment arms (20 patients per arm): 1) spinal manipulation, 2) spinal mobilization, or 3) sham Laser. Treatments will be delivered twice per week for 3-weeks. The clinical outcome data will be pooled across the three physiological experiments resulting in 54 subjects/treatment arm. The table below summarizes the enrollment plan.

.

**Experiment**	**Treatment Arm**			**Healthy Control**
	**Manipulation**	**Mobilization**	**Sham Laser**	
Muscular Effects	14	14	14	
Spinal Effects	20	20	20	60*
Cortical Effects	20	20	20	
**Σ of All Groups**	54	54	54	

*Modified Spinal Effect experiment

<u>Study Location:</u> The primary site for the RELIEF Study will be at the Ohio Musculoskeletal and Neurological Institute's (OMNI) main facility on the 2nd floor West Wing of Irvine Hall. This is a laboratory-based facility designed for specifically for clinical research.

<u>Study Duration:</u> The study duration for an individual participant with low back pain is ~ 8 weeks. Specifically, we expect that the typical amount of time between the event of enrollment ascertainment and randomization will be 3-5 days (maximum 7), and that randomization will occur when study participants begin a study intervention (visit 2). The study intervention will occur over a 3-week period followed by approximately 4-week follow-up. Thus, in total the study duration for an individual participant will be ~ 8 weeks. Specifically, in the first visit we will obtain informed consent and conduct a screening exam to determine if candidates meet the inclusion/exclusion criteria for a given experiment. If subjects meet the inclusion/exclusion criteria for an experiment they will be enrolled into the study and allocated to one of three experimental measurement groups (i.e., Muscular, Spinal, or Cortical). At the second visit, participants will then be randomized to an intervention arm (i.e., mobilization, manipulation, or laser), baseline assessments of both clinical and physiological outcome measures will be obtained, a treatment intervention will be performed, and clinical and physiological outcome measures will be repeated (i.e., follow-up assessment). Participants will then undergo an additional five treatments over the next three weeks (visits 3-7; total treatments: 6; treatment frequency: 2x/week). It should be noted that following treatment 1 we will assess expectancy and credibility expectations for the respective treatment intervention. Three days +/- 1-day after the final treatment, subjects will report back to the laboratory for a follow-up assessment of the clinical and physiological outcome measures (visit 8). Lastly, to determine the long-term effects of the interventions, subjects will report back to the laboratory approximately 4 weeks (28-days +/- 4-days) after their last treatment for the final follow-up assessment of the clinical and physiological outcome measures

(visit 9). The study duration for a participant in the healthy control group is 3-hour including screening, baseline assessments, and lab testing. The anticipated duration for the RELIEF Study is 5-years. We anticipate the total enrollment time to take up to 4 years (48 months). The required monthly enrollment rate for study completion in the proposed time frame is 3.4 subjects/month.

Randomization, Stratification, and Blinding:

- *Randomization & Stratification*: The study statistician will use the R statistical language to block-randomize treatments stratified by sex for each of the three separate experiments. Specifically, within each experiment there will be two strata within which we will create blocks of 3 patients, where treatments are permuted. Based on the randomization table the study coordinator will assign subjects to the treatment arms. In instances where multiple experiments are actively enrolling study participants and a participant meets eligibility requirements for more than one experiment, we will then use an adaptive randomization method to allocate them to one of the experiments. This method ensures that an experiment with fewer participants enrolled will have a higher allocation ratio. Specifically, with two potential experiments, the allocation ratio for Experiment $_i$ (p_i) will be $p_i = 1\text{-}n_i/N$, where n_i is the sample size for Experiment $_i$ and N is the total sample size thus far enrolled. With three active experiments, the allocation ratio will be $p_i = (1\text{-}n_i/N)/2$. Note that this allocation to an experimental measurement group occurs prior to the randomization to a treatment arm.
- *Masking or Blinding:* In the RELIEF Study the principal investigators, the statistician, and members of the data collection team will remain blinded to intervention assignment throughout the duration of the study. They will be given the identifying codes only at the end of the study when it is necessary to interpret the results of the study. The un-blinded study coordinator, who is responsible for scheduling testing and treatment sessions, will serve to receive the study patients and escort them to the various testing and treatments sites to minimize the interaction between patients; however, the study coordinator will not participate in the assessments.

4. SELECTION AND ENROLLMENT OF PARTICIPANTS

4.1 Inclusion Criteria

Participants must meet all of the following inclusion criteria to participate in this study:

- Between 18 to 45 years of age.
- Answer yes to the following questions
  - Have you had low back pain constantly or on most days for the last three months?
  - Has your back pain caused you to seek care or consultation from a health care provider?
- Average pain intensity, assessed using the Numerical Pain Rating (NPR) scale over the past week ≥ 2 on a 0-10 numerical pain scale
- Roland Morris Disability Questionnaire score ≥ 4
- Exhibit 3 of 4 of the following findings reported in the clinical prediction rules for spinal manipulation
  - No symptoms distal to the knee
  - FABQ work subscale score <19
  - At least 1 hypo-mobile lumbar spinal segment
  - At least 1 hip > 35 degrees internal rotation range of motion
- Exception: Participants in the healthy control group must report no low back pain (i.e., 0 rating on NPR, 0 score on Roland Morris Disability Questionnaire, and answer “No” to the following question: Have you had low back pain in the past 1-year that interrupted work/play or caused you to seek care from a healthcare provider?)

4.2 Exclusion Criteria

To be eligible for the study participants must not:

- Have a personal history of the following neurological disorders: Alzheimer’s, Amyotrophic Lateral Sclerosis, Multiple Sclerosis, Parkinson’s, Stroke
- Have a personal history of the following cardiorespiratory disorders: Congestive heart failure, Heart attack in past 24 months
- Have a personal history of the following musculoskeletal disorders: Rheumatoid Arthritis, pathologic fractures of the spine, avascular necrosis or osteonecrosis, severe osteoarthritis. Including a history of spine surgery or a hip arthroplasty
- Have active cancer
- Be Blind
- Have used narcotics or muscle relaxants within 30 days prior to study enrollment.
- Report being pregnant, lactating, or that they anticipate becoming pregnant in the next 3-months
- Have a body mass index greater than 35 kg/m2
- Have clinical depression (i.e., subjects who score 24 or higher on the Center for Epidemiology Depression Scale).
- Report unexplained weight loss over the past month (>10 lbs).

- Report they have pending litigation related to an episode of LBP or are receiving any type of disability services related to low back pain.
- Report having received any manual therapy intervention applied to the spine 30 days prior to first study intervention.
- Current drug or alcohol use or dependence that, in the opinion of the PIs, would interfere with adherence to study requirements.
- Applies to Experiment 3 (Cortical Effects) only: Have a personal or family history of epilepsy or seizures, Migraine Headaches within 6 months (186 days) of V2 physiologic testing, or taking medications in the benzodiazepine class.
- Applies to Experiments 1 and 3 (Muscular Effects and Cortical Effects) only: Have contraindications for exposure to a magnetic field.
- Applies to Experiment 1 (Muscular Effects) only: Have a positive result from pregnancy test administered the day of each MRI.

The supplemental research will enroll an additional 60 healthy participants with no history of LBP and who are matched on sex, age (± 3 years), height (± 2 inches), and weight (± 10 lbs) to the participants recruited into **Experiment 2** of the RELIEF study. The same exclusion criteria are applied to the control group as is used for the RELIEF study LBP participants.

4.3 Study Enrollment Procedures

Recruitment: Study candidates will be: 1) referred from University Medical Associates Clinic, 2) referred by community physicians and other local health care providers, 3) self-referred, or recruited through 4) ResearchMatch.org.

Origin of the referral will be documented. Subjects who do not meet the eligibility criteria will be recorded and the reasons for ineligibility will be noted on the Study Completion form. Subjects who qualify for the study and decline participation will be asked to indicate their reasons for refusal to participate and these will be recorded. The multiple experiments will be permitted to run simultaneously, but this is not required per se. An individual will not be permitted to enroll in more than one experiment.

ResearchMatch.org will be utilized as a recruitment tool for this protocol. ResearchMatch.org is a national electronic, web-based recruitment tool that was created through the Clinical & Translational Science Awards Consortium in 2009 and is maintained at Vanderbilt University as an IRB-approved data repository **(*see IRB #090207***

The Steering Committee will monitor study recruitment on a monthly basis and based on those data will determine whether alternate recruitment strategies should be instituted.

Consent Procedures: An initial screening will be conducted with all participants to describe the research study and determine their interest. For subjects who are interested in further information and potentially volunteering for the study we (the study coordinator and/or investigators) will meet with interested participants. At this time individuals interested in participating can ask questions about the study. If they wish to be considered for the study they will then be asked to read and sign an informed consent document. Subjects must provide written informed consent prior to implementation of any study procedures. This will take place in a quiet, private room. Once the subject has read over the document, a member of the investigative team will review the entire document to be sure that the procedures and time commitments are understood. Comprehension and autonomy will be assessed by asking subjects to explain in their own words what the study is about. Informed consent must be obtained before

participants are screened. Once the study is described in detail to a candidate, if the candidate continues to expresses interest in the study we will consent them prior to beginning the screening process. Participant is notified regarding end of active protocol participation during Visit 9.

Randomization Procedures: The study statistician will use the R statistical language to block-randomize treatments stratified by sex for each of the three separate experiments. Specifically, within each experiment there will be two strata within which we will create blocks of 3 patients, where treatments are permuted. The study coordinator will use this table to assign subjects to the treatment arms.

5. STUDY INTERVENTIONS

5.1 Interventions and Administration

Manipulation Treatment: All patients will receive the same technique. The participant will be placed in a side-lying position facing the clinician with the more painful side facing upward. If the participant can not specify a side, the clinician will select the side to be manipulated. The clinician will passively flex the participant's hips and knees to induce lumbar spine flexion. The degree of flexion will be dependent on the level of the identified hypomobile segment. For example, if the clinician identifies hypomobility at the third lumbar vertebra, they will flex the knees until they feel the spinous process of the fourth lumbar vertebrae begin to move. Next, the clinician will passively rotate the participant's torso opposite to the side they are lying on until they feel rotation in the vertebra above the suspected lesion. Now the participant is positioned for the high velocity low amplitude thrust procedure. The clinician will then deliver a rapid thrust to the shoulder (anterior to posterior force) and pelvis (posterior to anterior force) resulting in a rotation force couple on the hypomobile segment. If a cavitation (that is, a "pop") occurs, the treatment will be considered complete. If no cavitation was produced, the participant will be repositioned and the manipulation will be attempted again. A maximum of 2 attempts per side will be permitted. If no cavitation is produced after the fourth attempt, the treatment will be considered complete.

Mobilization Treatment: All patients will receive the same technique. The participant will be placed in a side-lying position facing the clinician with the more painful side facing upward. If the participant can not specify a side, the clinician will select the side to be mobilized. The clinician will passively flex the participant's hips and knees to induce lumbar spine flexion. The degree of flexion will be dependent on the level of identified hypomobile segment. For example, if the clinician identifies hypomobility at the third lumbar vertebra they will flex the knees until they feel the spinous process of the fourth lumbar vertebrae begin to move. Next, the clinician will passively rotate the participant's torso opposite to the side they are lying on until they feel rotation in the vertebra above the suspected lesion. Now the participant is positioned for the mobilization procedure. The clinician will then place one hand on the anterior shoulder and one hand on the posterior pelvis. The participant will then be asked to try to gently push the shoulder and pelvis into the clinician's hands. Thus they will be performing an isometric rotation of the torso resulting in a rotation force couple on the hypomobile segment. The clinician will have the participant hold the contraction for a minimum of 7 seconds and up to a minute if the patient can comfortably tolerate the stretch. The clinician will reposition the patient as needed and repeat the treatment for up to 10 minutes.

Sham Laser Treatment: Sham Laser will be delivered while the study participant is placed in a side lying position with the hips and knees flexed as described for the manual therapy treatments. The clinician and the participant will don protective eye goggles (standard operating procedure in Laser therapy) and the clinician will apply the laser head over the primary area of pain and proceed to perform the technique with no power to the Laser head (i.e., sham Laser).

Administration of Interventions:

- Controlling Treatment Time: The treatment duration time will be held constant across all treatment groups. Specifically, each treatment group will receive between 10-15 minutes of contact time with the clinicians. Following the first treatment intervention, we will quantify subject expectations of treatment success using the credibility/expectancy questionnaire developed by Devilly and Borkovec.[51]

- The RELIEF Study Treatment Provider Team: Individuals on the RELIEF Study treatment provider team will deliver the respective treatments. Members of the treatment provider team must: 1) hold either a D.C., D.O., or P.T. degree and be licensed in the state of Ohio, 2) have received formal training in manual therapies, 3) have at least 3 years of clinical experience using manual therapies on a regular basis, and 4) complete a workshop organized by the PIs and The RELIEF Study Medical Director (Dr. Timothy Law, D.O.). All members of the RELIEF Study Treatment Provider Team will be required to complete the CITI Training program prior to interacting with human subjects (see Appendix H: Copies of human subjects training certificates).

- Study Coordination: Study participants will be given an appointment time and told to report to the treatment location. Both the study participant and the treatment provider will be sent a pre-appointment reminder (i.e., a phone call, text message, or email reminding them of the appointment). On the day of the appointment study participants will report to the study facility and a research staff member will escort the participant to the examination room where the treatment will be performed. In the event that the participant must wait to receive their treatment care will be taken to ensure that multiple study participants are not waiting in the same room (to minimize interaction among study participants).

5.2 Handling of Study Interventions

Each subject will, of necessity, be aware of group assignment. However, study personnel who collect the outcome measures will be blinded to the subject’s group assignment as will the PIs and the statistician.

5.3 Concomitant Interventions

5.3.1 Allowed Interventions

- Over the counter pain relief medications (e.g., Non-Steroidal Anti-Inflammatory Drugs, Acetaminophen, Aspirin).
- Heat or Ice.

5.3.2 Required Interventions

- None.

5.3.3 Prohibited Interventions

- Narcotics
- Muscle relaxants
- Any treatment specific for low back pain delivered by a health practitioner (e.g., physician, physical therapist, chiropractor, massage therapist, or acupuncturist).

5.4 Adherence Assessment

Adherence to all scheduled intervention contacts is recorded by the study coordinator into a tracking system. Reports on adherence will be reviewed during regular meetings of the Recruitment, Adherence, and Retention Committee (see section 12). If a study participant misses a scheduled treatment every effort will be made to re-schedule that treatment in the proposed weekly time frame. Successful adherence will be defined as study participant who achieve at least an 83% adherence rate (i.e., receive at least 5 out of the 6 treatments).

6. STUDY PROCEDURES

The Schedule of Evaluations in section 6.1 details the temporal occurrence of all study evaluations.

6.1 Schedule of Evaluations.

ASSESSMENT	Screening Visit	OA1	T1	OA2	T2	T3	T4	T5	T6	OA3	OA4
Visit Number	1	2	2	2	3	4	5	6	7	8	9
Week Number	-1	1	1	1	1	2	2	3	3	4	8
Informed Consent	X										
Demographics	X										
Low Back Pain Rating - NPR	X	X		X	X	X	X	X	X	X	X
RMDQ	X	X								X	X
CES-D	X										
FABQ	X										
TSK	X										
Medical History	X										
Medication History	X										
Inclusion/Exclusion Criteria	X										
Physical Exam	X										
Enrollment/ Randomization	X										
Prior & Concomitant Medications		X			X	X	X	X	X	X	X
Change in Medication Use		X			X	X	X	X	X	X	X
PROMIS		X								X	X
Physiological Outcomes		X		X						X	X
Intervention			X		X	X	X	X	X		
Expectancy/Credibility					X						
Intervention Administration Form			X		X	X	X	X	X		

OA: Outcomes assessment; T: Treatment session.

6.2 Description of Evaluations

6.2.1 Screening Evaluation

Consenting Procedure

Subjects must provide written informed consent prior to implementation of any study procedures. This will take place in a quiet, private room. Once the subject has read over the document, a member of the investigative team will review the entire document to be sure that the procedures and time commitments are understood. Comprehension and autonomy will be assessed by asking subjects to explain in their own words what the study is about. Informed consent must be obtained before participants are screened. Once the study is described in detail to a candidate, if the candidate continues to expresses interest in the study we will consent them prior to beginning the screening process. A copy of the signed and dated consent form will be given to participants, and the original document will be placed in subjects' individual study files.

Screening & Physical Exam

The purpose of the screening visit (visit 1) is 1) to describe the study protocol to candidates and obtain informed consent, and 2) to determine if study candidates will qualify for the study. The screening visit will take approximately two hours. During the screening visit the study coordinator will complete, in cooperation with the study candidate, the following:

- Demographic Form: This form documents study candidates gender, date of birth, race, ethnicity, and the date the informed consent document was signed (see appendix).
- Numerical Pain Rating (NPR): This scale will quantify the study candidates rating of their low back pain symptoms (see appendix).
- Roland Morris Disability Questionnaire: This form quantifies the self-report level of disability (see appendix).
- Center for Epidemiology Depression Scale (CES-D) Depression will be assessed using the CES-D survey (see appendix).
- Fear Avoidance Belief Questionnaire (FABQ): This is used to assess candidate's level of pain related fear (see appendix).
- Tampa Scale for Kinesiophobia (TSK). This is used to assess candidate's level of fear of movement (see appendix).
- Medical History Form: This form documents study candidate's medical history (see appendix).
- Medication History: This form documents the study candidate's recent and current medication usage (see appendix).
- Inclusion-Exclusion Form: This form will determine whether the study candidate meets all of the inclusion and exclusion criteria (see appendix).
- Physical Exam Form: This form documents the study candidate's physical exam findings (see appendix).

Re-screening participants may be considered and reviewed by the PIs on a case-by-case basis. Visit 1 screening must occur within 30-days of prescreen completion. Visit 2 (intervention 1) must occur within 7 days of V1 screening. If these deadlines are not met the participants will be assigned a new PID, re-screened, and re-consented.

6.2.2 Enrollment, Randomization and Baseline

Enrollment

A single informed consent form that describes both screening and study procedures will be used. The date of enrollment in The RELIEF Study will be defined as the experiment allocation date. Randomization to treatment arms occurs on the day of the first study intervention (visit 2).

Randomization and Enrollment Form: This form will note the enrollment and randomization dates (see appendix).

Randomization

Randomization will precede baseline testing and intervention administration. Study participants will be randomly allocated to the specific experimental measurement group (i.e., muscular, cortical, spinal) after being deemed eligible for the study and enrolled (i.e., at the conclusion of visit 1). Randomization occurs at visit 2 when participants are randomized to a treatment intervention arm (i.e., mobilization, manipulation, laser) A maximum of 7-days will be permitted between enrollment and randomization or initiation of the study intervention (i.e., a maximum of 7-days will be permitted between visits 1 and 2).

Baseline Assessments

The following baseline clinical outcomes and evaluations will be obtained from all study participants, regardless of which treatment arm they are assigned to:

- Numerical Pain Rating (NPR): This scale will quantify the study candidates rating of their low back pain symptoms (see appendix).
- Roland Morris Disability Questionnaire: This form quantifies the self-reported level of disability (see appendix).
- PROMIS Pain Measures: The NIH PROMIS system will be used to assess outcomes related to pain intensity, pain interference, pain behavior, and physical function.
- Prior & Concomitant Medication Form: This form documents the study participant's recent and current medication usage for their low back pain (see appendix).
- Change in Medication Use: This form documents any changes in medication use not related to low back pain.

The following baseline physiological outcomes will be obtained based the experimental group to which they are randomly assigned to (i.e., muscular, spinal, cortical):

- Group 1 (Muscular Effects):
    - Skeletal muscle proton transverse relaxation time (T2) of the lumbar muscles.
        - Location & Assessment Team: Data will be collected at the Ohio Musculoskeletal and Neurological Institutes Biomedical Imaging Suite located in Irvine Hall at Ohio University. Blinded members of The RELIEF Study Team (i.e., blinded students, staff, and the principal investigators) will be involved with the data collection and analysis.
        - Methods: Female participants will be screened for pregnancy using a urine pregnancy test; positive results will exclude a participant from remaining in the study. Data will be collected and analyzed as we have previously

described [18]. In brief, standard spin-echo magnetic resonance images of the lumbar spine will be obtained. Ten-mm thick transaxial images (2000 milliseconds repetition time; 30 milliseconds and 65 milliseconds echo times; 10-mm slice-to-slice interval) will be obtained from the lumbar region while study participants lie supine position with the legs slightly bent. After scanning, the images will be transferred to a computer for calculation of muscle T2 using the ImageJ software (Research Services Branch, National Institutes of Health). Muscle T2 will be calculated on a pixel-by-pixel basis and averaged over five slices. The T2 values will be calculated from regions of interest within the following muscles: psoas, quadratus lumborum, multifidus, and the iliocostalis lumborum/longissimus thoracis (these two muscles are grouped due to the difficulty in defining distinct fascial borders in some subjects). The dependent variables calculated from the MRI data will be T2 and T2 asymmetry. T2 will be calculated by averaging the left and right side values for each individual muscle, whereas T2 asymmetry is the percent difference in T2 between the sides. Prior to the MRI scan subjects will lie supine for 30-minutes to minimize the effects of fluid shifts on the calculated outcomes.

- **Incidental Findings:** Incidental findings are traditionally defined as results that arise that are outside the original purpose for which the test or procedure was conducted. Due to the nature of the testing being conducted in this research study, it is possible that unanticipated, incidental findings will occur as it relates to MRI testing procedures. If an investigator observes something of concern (i.e., a potential incidental finding) they will be asked to report this concern to the PI (Thomas) and the Medical Director (Law). Individuals with specific expertise may at times be contacted for a consult of the finding using de-identified data (e.g., a radiologist may be consulted). The Medical Director will inform the study participant of the incidental finding if he believes the finding has 1) important health implications for the participant and the associated risks are established and substantial, and 2) the finding is actionable (i.e., there are established therapeutic or preventative interventions or other available actions that have the potential to change the clinical course of the disease), and 3) the test is analytically valid, and 4) the informed consent signed by the participant stated that results may be returned. Dr. Law will also offer to contact the study participant's designated health care provider to discuss the incidental finding if they study participant wishes for him to do this. However, in this event, the study participant must sign an authorization to disclose form. Findings that are not unanticipated for the population being studied will not follow this process; however, we will offer the study participants a copy of their results and answer any questions they may have as it relates to these types of findings.

- Group 2 (Spinal Effects)
  - o Short-latency stretch reflex excitability.
    - Location & Assessment Team: Data will be collected at the Motor Control Laboratory located in Grover Center at Ohio University. Blinded members of The RELIEF Study Team (i.e., blinded students, staff, and the principal investigators) will be involved with the data collection and analysis.

- Methods: Data will be collected and analyzed as we have previously described [43]. In brief, electrical signals will be recorded bilaterally from the erector spinae (ES) muscles using bipolar surface electrodes located longitudinally over the muscle at the vertebral level of L2 and L4 on shaved and cleaned skin with a reference electrode located on the anterior superior iliac spine. The electromyogram (EMG) signals will be amplified (1000×), bandpass filtered (10–500 Hz), and sampled at 5,000 Hz. Erector spinae muscle stretch reflexes will be elicited by mechanically tapping the belly of the left and right erector spinae muscles at the L3 vertebral level between the EMG electrodes. To elicit the stretch reflex the electromechanical prodder will be placed against the skin and the pressure applied to the low back tissue will be gradually increased to a pre-load of 30-N at which time the device will deliver a mechanical tap to the muscle with a net force of 90-Newtons. A total of 10 short-latency stretch reflexes will be elicited from each muscle by tapping the muscle with at least 10 s separating each reflex response. The corresponding EMG responses will be recorded, and the peak-to-peak amplitude of the reflex responses will be averaged to assess stretch reflex excitability. During these measures, subjects were asked to sit with an upright posture while their hands rested in their lap.

o Trunk muscle onset latencies in response to postural perturbations.

- Location & Assessment Team: Data will be collected at the Motor Control Laboratory located in Grover Center at Ohio University. Blinded members of The RELIEF Study Team (i.e., blinded students, staff, and the principal investigators) will be involved with the data collection and analysis.
- Methods: *In this experimental setup, the subject is seated with the lumbar spine in a neutral position. The subject's pelvis is securely fastened using seatbelts that cross the mid-thigh and across the anterior superior iliac spine. A chest harness is snugly attached to the subject at the level of the 3rd thoracic vertebrae. Kevlar cables (3/16" diameter) are attached to load cells affixed to the 4 corners of harness by gimbals and run to the corners of the reference frame through a pulley system to attach to actuators set in force control mode. This will allow us to precisely control the magnitude of the perturbing force as well as the duration of this pulse. Specifically, the participant will sit in the perturbation reference frame with their arms held across their chest. To generate the perturbing force, the actuators will be engaged in pairs (e.g. front, back, left side, right side) to induce a perturbation in one of the following directions: flexion, extension, side-bend left, side-bend right, rotation left, or rotation right. The actuators will provide a 100-ms duration perturbation force equal to 10% of trunk mass calculated from anthropometrics.[52] The forces will be delivered at random time intervals between 2-30 seconds. The onset of the force will result in a small trunk angular displacement (5-10 degrees) and a long latency postural reflex response of the trunk muscles.*
- <u>EMG Data</u>: Surface EMG electrodes will be placed bilaterally over the muscle belly of the following trunk muscles using double sided tape collars: 1) Erector Spinae at the L2 level aligned parallel to the line between the posterior superior iliac spine (PSIS) and the lateral border of the muscle at the 12th rib, 2) Rectus Abdominis (RA) 1 cm above the

umbilicus and 2 cm lateral to the midline, 3) External Oblique (EO) just below the rib cage along a line connecting the most inferior portion of the costal margin and the contralateral pubic tubercle, and 4) Internal Oblique (IO) 1 cm medial to the anterior superior iliac spine (ASIS) and just beneath a line joining both ASIS. EMG signals will be collected using a 16 channel BioPac MP150 System (bandwidth of 10- 500 Hz). The raw EMG data will be amplified (1k), sampled at 5000 Hz, filtered with a 60 Hz notch filter, and then rectified.

- Specifics regarding supplemental research is included in the appendix.

o Lumbar motion during volitional movement tasks

- Location & Assessment Team: Data will be collected at the Motor Control Laboratory located in Grover Center at Ohio University. Blinded members of The RELIEF Study Team (i.e., blinded students, staff, and the principal investigators) will be involved with the data collection and analysis.
- *Methods: Data will be collected and analyzed as previously described* [53-56]. In Brief, participants (while standing) will perform reaching tasks in which they are instructed to touch their index finger to targets located in the mid-sagittal plane. The target locations will be normalized to the subject's hip-to-shoulder length, arm length (humerus + forearm), and hip height. The "high" target will be placed in a position calculated so that the subject (with the elbow extended and the shoulder flexed 90 degrees) could, in theory, reach the target by flexing the pelvis 15 degrees without any flexion of the ankle, knee, and lumbar spine. The "low" target location will be determined such that the subject could, in theory, reach the targets by flexing the hip 60°. Normalization of target location allows for comparison of movement patterns across individuals and is sensitive to changes in low back pain populations.[55, 57-60] The participant will pause at the target for 2 seconds and then return to an upright posture. The participant will be allowed to rest between each trial. After completing the reaches at a comfortable pace, the participant will then be asked to reach for the targets as fast as possible. Again, three fast movement trials at each target location for each hand condition will be collected.Kinematic Data: Movement of light-reflective markers attached to the forearm, humerus, trunk, pelvis, thigh, and shank will be measured and recorded using a Vicon MX-13. The system is an optoelectric-based kinematic system that can track, with a spatial resolution of 0.1 mm, the three-dimensional coordinates of light reflective markers attached to the subject. The time series joint angle data are calculated from the 3-D segment coordinate data using an Euler angle sequence of: 1) flexion-extension, 2) lateral bending, and 3) axial rotation99 using Motion Monitor software. Kinematic data will be sampled at 100Hz.
- Joint Excursions: Joint excursions are defined as the change in joint angle, and are defined as the difference between the joint angles at the beginning of the trial before the go signal and those extracted 100 ms after target contact

- Group 3 (Cortical Effects)

o Motor evoked potential amplitude, intracortical facilitation, and short-interval

intracortical inhibition

- Location & Assessment Team: Data will be collected at the Ohio Musculoskeletal and Neurological Institute in Irvine Hall at Ohio University. Blinded members of The RELIEF Study Team (i.e., blinded students, staff, and the principal investigators) will be involved with the data collection and analysis.
- Methods: Data will be collected and analyzed as we have previously described [43]. In brief, electrical signals will be recorded bilaterally from the erector spinae (ES) muscles using bipolar surface electrodes located longitudinally over the muscle at the vertebral level of L2 and L4 on shaved and cleaned skin with a reference electrode located on the anterior superior iliac spine. The electromyogram (EMG) signals will be amplified (1000×), bandpass filtered (10–500 Hz), and sampled at 5,000 Hz. For the TMS measures, the center of a 110-mm double cone TMS coil will be positioned over the vertex. Single- and paired-pulse magnetic stimuli will be delivered using a Magstim 200^2 magnetic stimulator with the direction of current flowing from an anterior-to-posterior direction. During TMS, subjects were asked to sit with an upright posture while their hands rested in their lap. The TMS protocol will begin by delivering an initial single pulse at 50% of the maximum stimulator output to determine if this stimulus intensity is above or below motor threshold. Based on this finding the motor threshold will be determined by increasing or decreasing the stimulus intensity in 5% increments to determine the stimulation intensity where motor evoked potentials are consistently observed. Once the motor threshold is determined single pulses at intensity equal to 130% of motor threshold will be delivered to quantify motor evoked potential peak-to-peak amplitude. Next, intracortical facilitation will be quantified by delivering two pulses (conditioning and test pulse) with an interstimulus interval of 15-ms. The conditioning pulse intensity will be set to 70% of motor threshold and the test pulse intensity will be set to 130% of motor threshold. Short-interval intracortical inhibition will be assessed by delivering these pulses at the same intensity except with an interstimulus interval of 3-ms. A maximum of 6 trials for each outcome will be assessed in a blocked (a single pulse followed by the paired-pulse trials), with ~15-seconds separating each pulse sequence and ~60-seconds separating each block.

6.2.3 Blinding

In the RELIEF Study the principal investigators, statistician, and members of the data collection team will remain blinded to intervention assignment throughout the duration of the study. They will be given the identifying codes only at the end of the study when it is necessary to interpret the results of the study. To guard against un-blinding, only limited data categorized by intervention assignment will be released to the study investigators (e.g. adherence data), and these data will only be provided in aggregate. Secondly, the RELIEF Study Treatment Provider Team, who cannot be blind to the treatment assignment per se, will be blinded to the specific hypotheses of the study, and they will not be involved in any assessments or analyses. Lastly, the un-blinded study coordinator, who is responsible for scheduling testing and treatment sessions, will serve to receive the study patients and escort them to the various testing and

treatments sites to minimize the interaction between patients; however, the study coordinator will not participate in the assessments.

6.2.4 Follow-up Treatment Interventions and Assessments

Treatment Intervention 1 (visit 2)

- NPR (Assessed following treatment)
- Treatment Administration Form

The following physiological outcomes will be obtained immediately following the first treatment intervention based on the experimental group to which the participant was assigned to (i.e., muscular, spinal, cortical)

- Group 1 (Muscular Effects):
  - Skeletal muscle proton transverse relaxation time (T2) of the lumbar muscles.
- Group 2 (Spinal Effects)
  - Short-latency stretch reflex excitability.
  - Trunk muscle onset latencies in response to postural perturbations.
  - Lumbar motion during volitional movement tasks
  - Dynamic trunk stiffness
- Group 3 (Cortical Effects)
  - Motor evoked potential amplitude
  - Intracortical facilitation
  - Short-interval intracortical inhibition

Treatment Intervention 2 (visit 3)

- NPR
- Expectancy/Credibility Questionnaire
- Prior & Concomitant Medication Form
- Change in Medication Use
- Treatment Administration Form

Treatment Interventions 3-6 (visits 4-7)

- NPR
- Prior & Concomitant Medication Form
- Change in Medication Use
- Treatment Administration Form

Assessments (visits 8 & 9)

The following clinical outcomes and evaluation data will be obtained from all study participants between days 23-25 of the study (post-treatment measures) and between days 57-61 of the study (follow-up measures):

- NPR

- RMDQ
- Prior & Concomitant Medication Form
- Change in Medication Use
- Treatment Administration

The following physiological outcomes will be obtained based the experimental group to which they are randomly assigned (i.e., muscular, spinal, cortical) 3-days +/-2-days (post-treatment measures) and 28-days +/-4-days (follow-up measures) following the final study intervention (visit 7):

- Group 1 (Muscular Effects):
  - Skeletal muscle proton transverse relaxation time (T2) of the lumbar muscles.
- Group 2 (Spinal Effects)
  - Short-latency stretch reflex excitability.
  - Trunk muscle onset latencies in response to postural perturbations.
  - Lumbar motion during volitional movement tasks
  - Dynamic trunk stiffness
- Group 3 (Cortical Effects)
  - Motor evoked potential amplitude
  - Intracortical facilitation
  - Short-interval intracortical inhibition

7. SAFETY ASSESSMENTS

Spinal manual therapies are some of the most common treatments for LBP, and the serious risks of manual therapies are infinitesimally small. However, there is a risk in performing manipulations on individuals with contraindications for this treatment (e.g., osteomyelitis, history of pathological fractures). However, these conditions are associated with other signs and symptoms that would be identified in a medical screening to rule out participants prior to enrollment in the study.

7.1 Specification of Safety Parameters

At visits 2-9 the study staff will use the Health Status Reassessment CRF to ask the study participants if they have had any change in general health since the last visit. If fever or unexplained weight loss is reported, suggesting systemic infection / disease may be causing their low back pain, the clinician will do a basic physical exam and specifically assess for the possibility they have other systemic illness. They will be advised to seek medical care and research participation may be delayed, temporarily discontinued, or permanently suspended at the discretion of the examining clinician and in consultation with the Medical Directors if needed.

Before each treatment the study staff will use the Contraindications for Intervention CRF to inquire about change in back related signs indicative of increased severity of symptoms. Specifically if they have any increases in 1) radiating pain (i.e., burning, numbness, tingling sensations into the legs), 2) have noticed any muscle weakness in the legs, and 3) have any episodes of incontinence of bowel or bladder. If the study participant reports yes on any of these items the clinician will do a basic physical exam and assess neurological function by checking

myotomes, dermatomes, and reflexes to determine whether the study participant now presents with hard neurological signs (e.g., loss of motor function, significant changes in reflexes), or symptoms of other systemic disease. If the participant does present with hard neurological signs or serious systemic disease, they will be encouraged to seek consultation with their personal physician, and they will be excluded from further participation from the study.

Before each experimental measurement lab testing session (Visits 2, 8, and 9) the study staff will use the Contraindications for Lab Testing CRF to inquire specifically about symptoms of health issues contraindicated for the allocated experimental measurement lab test. Migraine headache will be assessed using The ID Migraine™ self-administered screener. If a possible contraindication is identified the PIs are consulted to determine safety before the lab testing begins.

All negative changes in health status will be recorded as Adverse Events, logged, and reported per requirements of the Ohio University IRB and NIH-NCCIH. In the case of dismissal from the protocol for the above stated reasons, the Medical Safety Committee will meet to determine whether the AE was caused by the intervention (Definite, Probable, Possible, Unknown) and an adverse event report will be filed according to the specific requirements of the Ohio University IRB and NCCIH.

7.2 Assessing, Recording, and Analyzing Safety Parameters

All Adverse Events will be reported to the Study Coordinator who will complete an Adverse Event Form and collaborate with study staff involved in the AE. If applicable, the Medical Director will review the AE and determine the Common Terminology Criteria for Adverse Events (CTCAE) scale. The Study Coordinator then compiles masked AE reports for the PIs to review.

Twice monthly, adverse events and serious adverse events will be assessed by the unblinded study coordinator and will be entered directly into REDCap™, an electronic data management system. Potential unanticipated side effects of the manual therapy interventions will be monitored at each study visit and entered by a research assistant. A tracking report will be developed as part of the database to allow the coordinator to assess recruitment, participant status, upcoming visits, and outstanding assessments.

Biannually an AE summary report will be generated and provided to the Independent Monitors for review and forwarded to the IRB and NCCIH.

7.3 Adverse Events and Serious Adverse Events

An adverse event (AE) is any untoward medical occurrence in a subject during participation in the clinical study or with use of the experimental agent being studied. An adverse finding can include a sign, symptom, abnormal assessment (laboratory test value, vital signs, electrocardiogram finding, etc.), or any combination of these.

A serious adverse event (SAE) is any AE that results in one or more of the following outcomes:

- Death
- A life-threatening event
- Inpatient hospitalization or prolongation of existing hospitalization
- A persistent or significant disability/incapacity
- A congenital anomaly or birth defect
- An important medical event based upon appropriate medical judgment

We will utilize the NCI Common Terminology Criteria for adverse Events which are a descriptive terminology that can be utilized for Adverse Event (AE) reporting and provides a grading (severity) scale for each AE. AEs will be labeled according to severity, which is based on their impact on the patient utilizing the following criteria:

Grade refers to the severity of the AE. The CTCAE displays Grades 1 through 5 with unique clinical descriptions of severity for each AE based on this general guideline:

Grade 1	Mild; asymptomatic or mild symptoms; clinical or diagnostic observations only; intervention not indicated.
Grade 2	Moderate; minimal, local or noninvasive intervention indicated; limiting age-appropriate instrumental ADL*.
Grade 3	Severe or medically significant but not immediately life-threatening; hospitalization or prolongation of hospitalization indicated; disabling; limiting self care ADL**.
Grade 4	Life-threatening consequences; urgent intervention indicated.
Grade 5	Death related to AE.

A Semi-colon indicates 'or' within the description of the grade.

A single dash (-) indicates a grade is not available.

A. <u>AE Attribution Scale</u>

Attribution: An assessment of the relationship between the AE and the medical intervention will be performed using the following the NCI guidelines which do not define an AE as necessarily "caused by a therapeutic intervention", after naming and grading the event, the clinical investigator will assign an attribution to the AE using the following attribution categories:

RELATIONSHIP	ATTRIBUTION	DESCRIPTION
Unrelated to investigational agent/intervention[1]	Unrelated	The AE *is clearly **NOT** related* to the intervention
	Unlikely	The AE *is **doubtfully related*** to the intervention
Related to investigational agent/intervention[1]	Possible	The AE ***may be related*** to the intervention
	Probable	The AE ***is likely related*** to the intervention
	Definite	The AE ***is clearly related*** to the intervention

7.4 Reporting Procedures

SAEs that are unanticipated, serious (Grade 3), and possibly related to the study intervention will be reported to the Independent Monitor(s), IRB, and NCCIH in accordance with requirements.

- Unexpected fatal or life-threatening AEs related to the intervention will be reported to the NCCIH Program Officer and Independent Monitoring Committee within 2 days and to the IRB within 1 day. Other serious and unexpected AEs related to the intervention will be reported to the NCCIH Program Official within 7 days.

- Other anticipated or unrelated SAEs will be handled in a less urgent manner but will be reported to the Independent Monitor(s), IRB, NCCIH, and other oversight organizations in accordance with their requirements. In the annual AE summary, the Independent Monitor(s) Report will state that they have reviewed all AE reports.

7.5 Follow-up for Adverse Events

In instances where an adverse event does occur, we will follow-up with participants until the event is resolved or until the IRB deems it unnecessary to continue to follow the participant.

7.6 Safety Monitoring

There is a completed DSMP for THE RELIEF Study that has been established and submitted to NCCIH.

8. INTERVENTION DISCONTINUATION

A subject would be discontinued from the study intervention if a medical condition develops that precludes the continuation of the treatment intervention. For participants who discontinue the RELIEF Study prior to completing all scheduled treatment sessions (regardless of the reason), we will make every attempt to obtain the above-mentioned outcome measurements. If the study participant is unwilling or unable to undergo the laboratory-based tests (e.g., difficulty tolerating the protocol, experienced an adverse event to a lab test) we will still attempt to obtain the clinical outcome measures. In instances where an adverse event does occur, we will follow-up with participants until the event is resolved or until the IRB deems it unnecessary to continue to follow the participant.

9. STATISTICAL CONSIDERATIONS

This study (R01 AT006978-01) was primarily designed to investigate the mechanisms underlying the biology of manual therapies. As such, our statistical power considerations are primarily based on the Mechanistic Component of The RELIEF Study. Below we detail the statistical considerations for the Mechanistic Component first, followed by a discussion of statistical considerations for the exploratory Phase II RCT Component. Importantly, this exploratory Phase II RCT with a nested mechanistic design allows us to maximize the data to be generated to address critical clinical and mechanistic questions necessary to improve our understanding of manual therapy treatments from chronic low back pain.

9.1 General Design Issues

<u>Mechanistic Component</u>
Below we list our primary and secondary hypotheses for the Mechanistic Component. Note that all sample size calculations are based on the primary hypotheses. For more specific information, see *Data Analysis* below.

- Group 1 (Muscular Effects):
  - Primary Hypothesis
    - We hypothesize that there is a significant effect of treatment on skeletal muscle proton transverse relaxation time (T2) asymmetry of the lumbar muscles either immediately following the first treatment intervention (OA2), post-treatment measure (OA3), and/or follow-up measure (OA4), after controlling for the baseline outcome value.
- Group 2 (Spinal Effects)
  - Primary Hypothesis
    - For short-latency stretch reflex excitability, we hypothesize that there is a significant effect of treatment on this outcome at OA2, OA3, and/or OA4, after controlling for the baseline outcome value.
  - Secondary Hypotheses
    - For trunk muscle onset latencies in response to postural perturbations, we hypothesize that there is a significant effect of treatment on the principal component score (i.e., the linear combination of onset latencies of eight muscles) at OA2, OA3, and/or OA4, after controlling for the baseline outcome value.
    - For lumbar motion during volitional movement tasks, we hypothesize that there is a significant effect of treatment on the principal component score at OA2, OA3, and/or OA4, after controlling for the baseline outcome value.
  - See appendix for hypotheses of the supplemental research
- Group 3 (Cortical Effects)
  - Primary Hypothesis
    - We hypothesize that there is a significant effect of treatment on measures indicative of increased intracortical excitability (i.e., increased motor evoked potential amplitude, increased intracortical facilitation, and/or decreased short-interval intracortical inhibition) at either OA2, OA3, and/or OA4, after controlling for the baseline outcome values.

<u>RCT Component</u>
To test the following RCT hypotheses, we will collapse the clinical outcome data across the three physiological outcome groups. Our Co-Primary Outcomes are NPR and RMDQ scores. The NPR and RMDQ are classic measures for the assessment of pain and disability in low back pain. Our secondary outcomes are change in PROMIS measures of Pain Behavior, Pain Interference, Pain Intensity, and Physical Function. Our primary endpoint is OA3 (visit 8, 3-days +/-1-day after completion of course of treatment intervention).

- Primary Hypotheses
  - o We hypothesize that the Manipulation and the Mobilization groups will display a significantly lower NPR level than the Sham Control at the post-treatment measure (OA3, our primary endpoint) after controlling for the baseline value. Because we have a directional hypothesis, we will use a one-tailed test.
  - o We hypothesize that the Manipulation and the Mobilization groups will display a significantly lower RMDQ level than the Sham Control at OA3 after controlling for the baseline value. Because we have a directional hypothesis, we will use a one-tailed test.
- Secondary Hypotheses
  - o We hypothesize that the Manipulation and the Mobilization groups will display significantly lower levels in PROMIS Pain Behavior, Interference, Intensity, and/or Physical Function than the Sham Control at OA3 after controlling for the baseline value.

9.2 Sample Size and Randomization

Sample Size Calculation for Mechanistic Component

For each primary hypothesis, sample size to achieve the power of 0.80 was computed using Monte Carlo simulation studies. For all simulation studies, the following were assumed:

- The Type I error rate (the alpha level) was set at 0.05 and the Type II error rate (the beta level = 1 - power) at 0.20.
- A first-order autoregressive temporal structure was assumed with ρ of -0.50.
- Holm's method was used to adjust the alpha level.
- To produce a plausible attrition rate, 10% of the computer-generated participants dropped out at either 2-days or 4-week post treatment measure completely at random; these participants contributed no scores at subsequent measures. To make the sample-size calculation slightly conservative, missing observations remained missing.
- Linear mixed effects regression (LMER) was used to analyze the data.
- To ensure the acceptable precision, each simulation study was repeated 10,000 times.
- The baseline measure was included as a covariate to improve power and precision of the test.

Mechanistic Component

- Group 1 (Muscular Effects):
  - o The primary dependent variable (DV) for this group is asymmetry in skeletal muscle proton transverse relaxation time (T2) of the lumbar muscles. Our pilot study (n=9) indicates that the primary difference in this outcome is 22.7% asymmetry observed at baseline.[18] Following a manual therapy treatment that included a combination of manipulation and mobilization techniques we observed a reduction in T2 asymmetry to 6.0% immediately following treatment and T2 asymmetry remained reduced (9.4% asymmetry) 2-days post-treatment with a residual standard deviation of 12.8. For this analysis, these values were used as population means. While the mean T2 asymmetry level for 4-weeks post-treatment is unknown, for our power analysis we assume an asymmetry value of 15.0%. For the mobilization treatment arm we assumed T2 asymmetry levels of 22.7%, 7.7%, 10.7%, and 16.7% at baseline, immediately following treatment #1, 2-days following a course of treatment, and 4-weeks post course of treatment, respectively. The rationale for these predicted group differences are based on our recent work suggesting that the cavitation (i.e., audible 'pop') associated with manipulation results in larger physiological changes (i.e., changes in stretch reflex excitability). Given that cavitation is less likely to occur with joint mobilization, we assume

smaller physiological effects from this treatment. For the Sham Control group, we set the baseline mean at the same 22.7% and a placebo effect of ~5%; that is, the population mean T2 asymmetry level for this groups at subsequent measures were set at 21.7%.
- Our simulation studies indicate that 14 participants in each of the 3 Groups were sufficient to achieve power of 0.80 to detect a significant Group effect either immediately following treatment #1, 2-days following a course of treatment, or 4-weeks post course of treatment given an attrition rate of 10% and appropriate alpha adjustments.

- Group 2 (Spinal Effects):
    - The primary dependent variable (DV) for this group is the short-latency stretch reflex amplitude. Our preliminary work indicates that spinal manipulation reduces stretch reflex amplitude in individuals with chronic LBP who exhibit an audible joint sound by 30% from 0.56 to 0.39.[61] In study participants with no audible joint sound, there was a non-significant 10% increase from 0.62 to 0.68. The baseline standard deviation was 0.25 for both groups. We assumed manipulation would result in a 25% reduction 2-days following a course of treatment and a 20% reduction 4-weeks post course of treatment. Given our pilot data indicating the importance of cavitation, we assumed smaller reductions in stretch reflex amplitudes at each time point for the joint mobilization treatment arm. (i.e., 28, 23, and 18% reductions at the three endpoints). Finally, we assumed that Sham Control would have the same mean (0.68) at each testing point.
    - Our simulation studies indicate that 20 participants in each of the 3 Groups were sufficient to achieve power of 0.80 to detect a significant Group effect either immediately following treatment #1, 2-days following a course of treatment, or 4-weeks post course of treatment given an attrition rate of 10% and appropriate alpha adjustments.
    - See appendix for sample size and randomization regarding supplemental research

- Group 3 (Cortical Effects):
    - Changes in cortical excitability following manual therapy treatments are assumed to follow our observed changes in spinal reflex excitability. Accordingly, we assume the same magnitudes of changes for these DV's as detailed above. Specifically, we assume a 30%, 25%, and 20% reduction in measures of intracortical excitability in the manipulation treatment arm immediately following treatment #1, 2-days following a course of treatment, and 4-weeks post course of treatment, respectively. Likewise, we assumed 28%, 23%, and 18% reduction for the mobilization treatment arm, and no change for the sham treatment arm. The residual variance was set at 50% of the initial value, similar to the Group 2 variables.
    - Our simulation studies indicate that 20 participants in each of the 3 Groups were sufficient to achieve power of 0.80 to detect a significant Group effect either immediately following treatment #1, 2-days following a course of treatment, or 4-weeks post course of treatment given an attrition rate of 10% and appropriate alpha adjustments.

Sample Size for RCT Component

Our co-primary outcomes are NPR and RMDQ at the primary endpoint (i.e., 3-days +/-1-day following a course of treatment). As stated previously, we will collapse the clinical outcome data across the three physiological outcome groups for a total sample size of 54/treatment arm. A recent review of a large number of RCTs on the effects of spinal manipulation for the treatment of chronic low back pain indicate that small treatment effect sizes are observed.[62] However, the effect sizes reported for studies that used a clinical prediction rule to identify individuals most likely to respond to manipulation treatment report large effect sizes. (e.g., effect size d > 1.3 for clinically meaningful reductions in disability).[63] The RELIEF Study will use a modified-clinical prediction rule to define a target population of patients with chronic low back pain who are most likely to respond to manual therapy treatment. However, because our population is chronic

rather than acute (a factor in the original clinical prediction rule) we took a more conservative approach and based our power calculations on the assumption of a moderate treatment effect (i.e., effect size d=0.5) to reduce disability.

- Our calculations are based on an assumed moderate effect size (effect size d=0.5; based on data from Maughan and Lewis' work indicating standard deviations for NPR being 2.5 and RMDQ being 6 for patients with chronic low back pain).[64] Our simulation studies indicate that 54 subjects/treatment arm is sufficient to achieve power of 0.80 to detect a significant treatment effect at our primary endpoint (i.e., 2-days following the course of treatment) given an attrition rate of 10% and appropriate alpha adjustments. With this sample size we have sufficient power to detect changes in NPR > 2 and RMDQ > 3, which are the classically defined levels of clinically-meaningful important differences in these outcomes.

Treatment Assignment Procedures

Randomization & Stratification: The study statistician will use the R statistical language to block-randomize treatments stratified by sex for each of the three separate experiments. Specifically, within each experiment there will be two strata within which we will create variable blocks of 3 or 6 patients, where treatments are permuted. Then the study coordinator will assign subjects to the treatment arms. In instances where multiple experiments are actively enrolling study participants and a participant meets eligibility requirements for *more than one experiment,* we will then use an adaptive randomization method to allocate them to one of the experiments. This method ensures that an experiment with fewer participants enrolled will have a higher allocation ratio. Specifically, with two potential experiments, the allocation ratio for Experiment $_i$ (p_i) will be $p_i = 1 - n_i/N$, where n_i is the sample size for Experiment $_i$ and N is the total sample size thus far enrolled. With three active experiments, the allocation ratio will be $p_i = (1 - n_i/N)/2$. Note that this process would occur prior to the randomization to a treatment arm.

- *Masking or Blinding:* In the RELIEF Study the principal investigators, the statistician, and members of the data collection team will remain blinded to intervention assignment throughout the duration of the study. They will be given the identifying codes only at the end of the study when it is necessary to interpret the results of the study. The un-blinded study coordinator, who is responsible for scheduling testing and treatment sessions, will serve to receive the study patients and escort them to the various testing and treatments sites to minimize the interaction between patients; however, the study coordinator will not participate in the assessments.

9.3 Interim Analyses and Stopping Rules

No interim analyses are planned as they relate to either the primary or secondary outcome data. An individual subject would be discontinued from the study intervention if a medical condition develops that precludes the continuation of the treatment intervention. Additionally, the study will be stopped if the IMC, in consultation with NCCIH, decides that the risks associated with the treatment interventions (i.e., excessive SAEs) outweigh the potential benefits of the knowledge gained from continuing the study.

9.4 Outcomes

The primary outcome variables for both components are provided below.

Mechanistic Component

- Group 1 (Muscular Effects):
    - Skeletal muscle proton transverse relaxation time (T2) of the lumbar muscles.
- Group 2 (Spinal Effects):

- o Short-latency stretch reflex excitability.
- o Trunk muscle onset latencies in response to postural perturbations.
- o Lumbar motion during volitional movement tasks.
- o Dynamic trunk stiffness
- Group 3 (Cortical Effects):
    - o Motor evoked potential amplitude.
    - o Intracortical facilitation.
    - o Short-interval intracortical inhibition.

<u>RCT Component</u>

Co-Primary Outcomes:

- Change in Numerical Pain Rating (NPR) score for average over the last 7-days
- Change in Roland Morris Disability Questionnaire (RMDQ)

Secondary Outcomes:

- Change in PROMIS Pain Behavior
- Change in PROMIS Pain Interference
- Change in PROMIS Pain Intensity
- Change in PROMIS Physical Function
- Change in NPR scores for current pain, and worst and best pain in the last 7-days

The primary endpoint will be OA3**.**

9.5 Data Analyses

Here we describe three stages at which both the Mechanistic and RCT data will be analyzed in the same fashion: preliminary analysis, checking assumptions, and detailed analytic approach. Data analysis regarding the supplemental research is included in the appendix.

<u>Preliminary Analysis:</u>

- At the first preliminary-analysis stage, we will examine whether the randomization is successful (i.e., the means for the three treatment arms do not differ in demographic variables or health characteristics) by using χ^2 tests for categorical variables and one-way ANOVAs for continuous variables. If significant differences in the demographic variables or health characteristics emerge, they will be treated as covariates in a model at the second stage. The effect of random factors (i.e., the blocks and treatment providers) will also be examined. If the variances of these factors were not significant at a liberal alpha level of 0.20, they will be excluded from further analysis. However, if a significant block and/or treatment-provider effect is detected, this will be included as a random effect in the model at the second stage. Likewise, we will perform an ANOVA to evaluate whether the means for the three mechanistic experiments differ in the clinical outcome variables. If the experiment factor is significant, this factor will be included as a fixed effect in further analysis. Otherwise, clinical outcome data will be pooled across the experiments.

- We will use means and standard deviations as the primary descriptive statistics for continuous variables and proportions for categorical variables. If variables are skewed, medians and interquartile ranges will be used. We will also compute pairwise correlation between a pair of endpoints as well as autocorrelations through all endpoints for each outcome measure. We will compute confidence intervals around the means as well as effect-size indices (e.g., Cohen's d for two-group comparisons and f for multiple-group comparisons) and confidence intervals around the effect sizes.

Checking Assumptions

- We will check for the following key assumptions: 1) independence of cases (the study design will satisfy this), 2) normal distribution of random intercepts and slopes as well as residuals, and 3) homogeneity of residual variances across the three treatment groups. To ensure that these assumptions are met, we will visually examine random intercepts, slopes, and residual, using boxplots, and q-q plots, and normalized residuals. If the normality or homogeneity assumption is violated, we will transform the data or use Generalized Mixed Linear Models to incorporate the non-normal distribution of the data. Outliers will be detected using Cook's distance and DFFIT. If one or more outliers are detected, we will check whether there has been a type or data mis-entry.

- The pattern of missingness will be examined in the following order. First, missing observations will be set as a dichotomous variable and its correlation with other factors will be examined. If the missing factor is not significantly related with any other factors, the missingness is tentatively deemed Missing Completely At Random (MCAR), and the Last Observation Carried Forward (LOCF) method will be used for an Intention-To-Treat (ITT) analysis (see below). If the missing factor is significantly related with other factors, the missingness it deemed Missing At Random (MAR), and maximum likelihood estimation will be used to estimate missing observations for the ITT analysis. Second, to test whether the missingness is Missing Not At Random (MNAR) or not, sensitivity analysis will be performed by imputing missing observations using a multivariate multiple imputation method under different scenarios. If the influence of missingness is extensive, multiple-imputed data will be used for the ITT analysis.

Detailed Analytic Approach:

Linear mixed-effects regression (LMER) analyses will serve as the main analytic framework, because it offers distinct advantages in statistical power and estimation of population parameters over ANOVA or MANOVA. First, LMER easily incorporates miscellaneous factors (e.g., blocks and treatment providers) as random effects so that the variances due to those factors can be partitioned. As a result, LMER models typically offer less biased parameter estimation and can also offer higher statistical power. It can model multiple dependent variables (e.g., motor evoked potential amplitude, intracortical facilitation, and short-interval intracortical inhibition for Group 3) and test the effect of treatment for each of these dependent variables simultaneously. This modeling technique avoids alpha inflations which typically result from multiple testing. Finally, LMER's maximum-likelihood estimation allows patients with missing observations to be included in a model if the observations are missing completely at random or missing at random. This advantage is important because some degree of attrition in unavoidable given the longitudinal nature of our project. Because we will stratify our samples by sex, sex will be included in each of the models we will describe below. We will test the significant main effect of sex as well as its interaction with the Treatment factor.

- **Intention to Treat Analysis (ITT):** We will include all randomized study participants who have at least baseline endpoint assessments in our ITT analysis. The ITT analysis is our primary method for assessing outcomes, and how it is performed will be determined based on the pattern of

missingness. If the missingness is deemed MCAR, the LOCF method will be used: simply the most recent valid observation will be "carried forward" to replace the subsequent missing observations. If the missingness is deemed MAR, missing observations will remain as missing, and maximum-likelihood estimation, the default estimation algorithm of LMER, will be used to estimate these observations. If the missingness is found to be MNAR, multiple-imputed data will be used to construct LMER models.

- **Per-Protocol Analysis (PPA):** For the PPA we will exclude study participants who: 1) fail to attend 4 out of 6 treatment sessions; and 2) receive prohibited concomitant interventions (section 5.3.3), or 3) develop an exclusionary medical condition while on study protocol. Finally, we will assess how comparable estimated parameters vary between ITT and PPA analyses. Below we describe our analytic plans for specific outcome measures.

Mechanistic Component

- Group 1 (Muscular Effects):
    - o We will test whether three group means will significantly differ from each other at either OA2, OA3, and/or OA4 with the baseline value included in the model as a covariate. Following the significant treatment effect, we will perform Fisher's Least Significant Difference (LSD) to examine which group significantly differs from which without any adjustment. This analytic process will be repeated for the outcomes for the other two Groups.

- Group 2 (Spinal Effects)
    - o For short-latency stretch reflex excitability, we will test whether three group means will significantly differ from each other at either OA2, OA3, and/or OA4 with the baseline value included in the model as a covariate.
    - o For trunk muscle onset latencies in response to postural perturbations and lumbar motion during volitional movement tasks, EMG responses from eight trunk muscles will be recorded each. To reduce the complexity of the data, we will first perform separate principal component analysis (PCA). The scores of the first principal component (i.e., the linear combination of onset latencies of eight muscles) will then be used as the DV for the LMER analysis. We hypothesize that there are significant effects of treatment on PC scores at OA2, OA3, or OA4, after controlling for the baseline outcome value.
    - o For the lumbar motion during volitional movement we will test whether three group means will significantly differ from each other at either OA2, OA3, and/or OA4 with the baseline value included in the model as a covariate.

- Group 3 (Cortical Effects)
    - o For motor evoked potential amplitude, intracortical facilitation and short-interval intracortical inhibition, we will test whether three group means will significantly differ from each other at either OA2, OA3, and/or OA4 with the baseline value included in the model as a covariate.

- Exploratory Analyses
  We will perform three exploratory analyses to examine the relationships between the changes in physiological outcomes and the changes in clinical outcomes as detailed below.
    - o We will calculate Pearson product-moment correlation coefficients for parametric data and Spearman's rank correlation coefficients for non-parametric data.

- We will explore the use of multiple regression techniques to elucidate the predictive relationships between and among the physiologic outcomes on clinical outcomes. To determine the relative contribution of the physiologic outcomes, the predictors will be entered simultaneously into the model (or potentially blocked if logical), and we will focus on the R^2 and the semi-partial r^2 values of the individual predictors.
- Lastly, we will conduct a mediational analysis. Here, the clinical outcome data from our primary endpoint (Y) will first be regressed onto the treatment independent variable (X). The baseline clinical outcome (Y_{Base}) will be included as a covariate. The X→Y path represents the *total effect* of the treatment interventions. Next, we will include a mediator in the model: the physiologic outcome immediately following treatment #1 (M_1). Additionally, baseline (M_{Base}) data will serve as a covariate. Both direct and indirect effects will be examined to assess the degree of mediation. The same mediational analysis will be repeated using the physiologic outcome obtained 3-days +/-1-day following a course of treatment (M_2) as the mediator.

RCT Component
To test the following RCT hypotheses, we will collapse the clinical outcome data across the three physiological outcome groups for a total sample size of 54/treatment arm.

- Primary Hypotheses
  - We will use separate analyses to test whether the manipulation or mobilization treatment intervention result in a significantly lower NPR level compared to the Sham Control at the post-treatment measure (OA3, our primary endpoint) after controlling for the baseline value. A 2 point reduction in NPR score will be considered a clinically-meaningful important difference.
  - We will use separate analyses to test whether the manipulation or mobilization treatment intervention result in a significantly lower RMDQ level compared to the Sham Control at the post-treatment measure (OA3, our primary endpoint) after controlling for the baseline value. A 3 point reduction in RMDQ score will be considered a clinically-meaningful important difference.

- Secondary Hypotheses
  - We will use separate analyses to test whether the manipulation or mobilization treatment intervention result in a significantly lower PROMIS-based outcome measures compared to the Sham Control at the post-treatment measure (OA3, our primary endpoint) after controlling for the baseline value.

- Exploratory analysis
  - To examine the immediate and longitudinal effects of the manipulation and mobilization treatment interventions, we will examine whether the treatment groups display significantly lower levels in NPR and/or RMDQ than the Sham Control at the other endpoints (i.e., OA2 and OA4), after controlling for the baseline value as a covariate.

  - We will use separate analyses to test whether the manipulation treatment intervention results in a significantly different NPR or RMDQ level compared to the mobilization treatment intervention at the post-treatment measure (OA3, our primary endpoint) as well as the other endpoints after controlling for the baseline value.

For all analyses two-tailed comparisons will be made with a pre-set significance level of 0.05. For all analyses, if there is a significant main effect, then we will perform Fisher's Least Significant Difference (LSD) to examine which group significantly differs from which without any adjustment. .

10. DATA COLLECTION AND QUALITY ASSURANCE

10.1 Data Collection Forms

All data collection forms are paper-based or electronic (eCRF) with the exception of the prescreen survey which is always an online form. As source documents, all forms are signed or electronically verified and dated by a study staff member. Forms are identified with Personal Identification Numbers with no personal identifying information. The Study Coordinator or Research Assistant transfers all data from the forms to the database. Every 2-weeks a Research Assistant performs a quality assurance check by comparing paper-based forms to data entry in the database.

All paper data collection forms will be kept in a locked file cabinet in the Study Coordinator's office or the respective offices or laboratories of the PIs. Data will also be entered into an electronic database. The data management system has been developed by the Ohio University Office of Research and Sponsored Programs. Specifically, we will use REDCap™ and Qualtrics™, both web applications for building and managing online surveys and databases. Subjects will be identified by a unique identification number and no personal identifying information will be stored in the study database or used in any of the analyses files.

10.2 Data Management

This is a single site study and as such Ohio University will serve as the Coordinating Center and is responsible for all data management.

10.3 Quality Assurance

10.3.1 Training

Prior to data collection, the following will take place:

1. The Steering Committee will review and approve all protocols.
2. All study personnel will be trained in all study procedures for which they are responsible.
3. The RELIEF Study Treatment provider team will undergo training to ensure successful and consistent delivery of the interventions across team members.

10.3.2 Quality Control Committee

This is a single site study and the Steering Committee will be responsible for overseeing quality control.

10.3.3 Protocol Compliance

We will monitor protocol compliance as follows:

1. The PIs will monitor visit attendance records on a monthly basis to determine whether subjects are adhering to the study protocol as designed.

2. The Steering Committee will review the summary of visit attendance records on a monthly basis to determine any deviation from the protocol.
3. The PIs and Biostatistician will annually review all protocols for compliance.

Protocol compliance:

1. A checklist of potential protocol violations will be created and maintained for tracking by the Steering Committee. Each protocol violation report will include the violation, whether the participant is continued in the treatment and assessment visits, the impact on the study procedures, and next steps for participant continuation. The number and type of violations will be monitored relevant to the total number of subjects recruited. Examples of protocol violations include enrollment of an ineligible subject, randomization of an ineligible subject, failure to collect all screening tests, serious adverse events not reported in a timely fashion, breach of confidentiality, subject lost to follow-up, and visits outside of time window.

2. A tracking report will be developed as part of the database to allow the study coordinator to assess recruitment, consent, participant status, upcoming visits, and outstanding assessments. Reports will be created to monitor compliance with data entry and data errors. Data errors should be limited since fields will be set to exclude implausible entries.

10.3.3 Metrics

The PIs will monitor treatment administration records on a monthly basis to determine whether study participants are receiving appropriate treatments as prescribed.

10.3.4 Protocol Deviations

A checklist of potential protocol deviations will be created and maintained for tracking by the Steering Committee (see appendix). Each protocol violation report will include a description of the violation, the type of deviation that occurred, whether the date the deviation occurred, and whether the IRB was notified. The Steering Committee will review protocol deviations on an as needed basis to determine if subjects should continue in the study, the impact on the procedures, and the next steps for subject continuation. Examples of protocol violations include enrollment of an ineligible subject, randomization of an ineligible subject, failure to collect all screening tests, serious adverse events not reported in a timely fashion, breach of confidentiality, subject lost to follow-up, and visits outside of time window.

10.3.5 Monitoring

The Steering Committee will be responsible for study monitoring. This committee will review and approve the study protocol prior to initiation of enrollment, will review SAEs and AEs at least twice annually and will be alerted to any interim concerns. Should any SAEs occur, they will be reported within 24 hours to the PIs.

11. PARTICIPANT RIGHTS AND CONFIDENTIALITY

11.1 Institutional Review Board (IRB) Review

This protocol and the informed consent document and any subsequent modifications will be reviewed and approved by the Ohio University IRB.

11.2 Informed Consent Forms

An initial screening will be conducted with all participants to describe the research study and determine their interest. For subjects who are interested in potentially volunteering for the study the study coordinator and/or principal investigators will meet with interested candidates. At this time individuals interested in participating can ask questions about the study. If they wish to be considered for the study they will then be asked to read and sign an informed consent document. A signed consent form will be obtained from each participant. The consent form will describe the purpose of the study, the procedures to be followed, and the risks and benefits of participation. A copy will be given to each participant or legal guardian and this fact will be documented in the participant's record. The principal investigators will work with the Ohio University IRB on an as needed basis to make provisions for special populations. For example, if a non-English speaker expresses interest in the study every attempt will be made to convert the informed consent document into their native language and provide a translator during all assessments.

11.3 Participant Confidentiality

All health-related data will be kept confidential by the investigators involved in this study. Specifically, only the investigators in the study will have access to patient data. Published or presented data will not identify patients in any way. Patients will not be audiotaped or videotaped. Data, forms, reports, and other records that leave the site will be identified only by a participant identification number (Participant ID, PID). All records will be kept in a locked file cabinet. All computer entry and networking programs will be done using PIDs only. Once this research has been completed all files will be kept for an additional five years and then destroyed. Additionally, while every effort will be made to keep study-related information confidential, there may be circumstances where this information must be shared with: 1) Federal agencies, for example the Office of Human Research Protections, whose responsibility is to protect human subjects in research; and 2) Representatives of Ohio University (OU), including the Institutional Review Board, a committee that oversees the research at OU.

11.4 Study Discontinuation

This study may be discontinued at any time by the IRB, the NCCIH, the OHRP, the FDA, or other government agencies as part of their duties to ensure that research participants are protected.

12. COMMITTEES

Steering Committee: The RELIEF Study Steering Committee, which is charged with the overall governance of study conduct, consists of James S. Thomas, PhD, PT, Brian C. Clark, PhD, Christopher R. France, PhD, Masato Nakazawa, PhD and Tim Law, D.O. The Steering Committee approves the final protocols and manuals of operations, supervises the overall execution of the trial, generates and approves study policies, considers modifications of the protocol and study operations, and plans and drafts study-related publications. The Steering Committee appoints and charges the subcommittees described below. All major scientific decisions are determined by majority vote of the Steering Committee.

Medical Safety Committee: The RELIEF Study Medical Safety Committee, which consists of James S. Thomas, PhD, PT, Brian C. Clark, PhD, Tim Law, D.O. and Study Coordinator (TBD), reviews masked study data related to the overall safety of study participation, develops safety reports for the Data and

Safety Monitoring Board, addresses IRB issues (related to participant safety) that may arise, reviews clinical practice-related issues and oversees the clinical safety of all study participants.

Data Safety and Monitoring Board: A Data Safety Monitoring Board (DSMB) that consists of an Independent Monitoring Committee IMC (IMC) monitors all aspects of the study, including those that require access to any blinded data.

Recruitment, Adherence, and Retention Committee: The RELIEF Study Recruitment, Adherence and Retention Committee, which consists of the James S. Thomas, PhD, PT, Brian C. Clark, PhD, Tim Law, D.O. and members of the Study Coordination and Data Collection Team (TBD), refines and optimizes protocols and strategies for recruitment, adherence and retention of study participants. The Committee oversees recruitment progress, intervenes in cases of under-recruitment, and reports recruitment progress to the Steering Committee.

13. PUBLICATION OF RESEARCH FINDINGS

This study is governed by a Steering Committee and publication of the results of this trial will be governed by the policies and procedures developed by the Steering Committee. Any presentation, abstract, or manuscript will be made available for review by the sponsor and the NCCIH prior to submission.

14. REFERENCES

1. Andersson, G.B., *Epidemiological features of chronic low-back pain.* Lancet, 1999. **354**(9178): p. 581-5.
2. Carey, T.S., J.M. Garrett, and A.M. Jackman, *Beyond the good prognosis. Examination of an inception cohort of patients with chronic low back pain.* Spine, 2000. **25**(1): p. 115-20.
3. Klenerman, L., et al., *The prediction of chronicity in patients with an acute attack of low back pain in a general practice setting.* Spine, 1995. **20**(4): p. 478-84.
4. Von Korff, M., *Studying the natural history of back pain.* Spine (Phila Pa 1976), 1994. **19**(18 Suppl): p. 2041S-2046S.
5. Nahin, R.L., et al., *Costs of complementary and alternative medicine (CAM) and frequency of visits to CAM practitioners: United States, 2007.* Natl Health Stat Report, 2009(18): p. 1-14.
6. Cleland, J.A., et al., *The audible pop from thoracic spine thrust manipulation and its relation to short-term outcomes in patients with neck pain.* J Man Manip Ther, 2007. **15**(3): p. 143-54.
7. Assendelft, W.J., et al., *Spinal manipulative therapy for low back pain.* Cochrane Database Syst Rev, 2004(1): p. CD000447.
8. Bronfort, G., et al., *Efficacy of spinal manipulation and mobilization for low back pain and neck pain: a systematic review and best evidence synthesis.* Spine J, 2004. **4**(3): p. 335-56.
9. Childs, J.D., et al., *A clinical prediction rule to identify patients with low back pain most likely to benefit from spinal manipulation: a validation study.* Ann Intern Med, 2004. **141**(12): p. 920-8.
10. Licciardone, J.C., A.K. Brimhall, and L.N. King, *Osteopathic manipulative treatment for low back pain: a systematic review and meta-analysis of randomized controlled trials.* BMC Musculoskelet Disord, 2005. **6**: p. 43.
11. Licciardone, J.C., et al., *Osteopathic manipulative treatment for chronic low back pain: a randomized controlled trial.* Spine (Phila Pa 1976), 2003. **28**(13): p. 1355-62.
12. Gatterman, M.I., et al., *Rating specific chiropractic technique procedures for common low back conditions.* J Manipulative Physiol Ther, 2001. **24**(7): p. 449-56.
13. Haas, M., E. Groupp, and D.F. Kraemer, *Dose-response for chiropractic care of chronic low back pain.* Spine J, 2004. **4**(5): p. 574-83.
14. Hoiriis, K.T., et al., *A randomized clinical trial comparing chiropractic adjustments to muscle relaxants for subacute low back pain.* J Manipulative Physiol Ther, 2004. **27**(6): p. 388-98.
15. Hondras, M.A., et al., *A randomized controlled trial comparing 2 types of spinal manipulation and minimal conservative medical care for adults 55 years and older with subacute or chronic low back pain.* J Manipulative Physiol Ther, 2009. **32**(5): p. 330-43.
16. Hurwitz, E.L., et al., *A randomized trial of medical care with and without physical therapy and chiropractic care with and without physical modalities for patients with low back pain: 6-month follow-up outcomes from the UCLA low back pain study.* Spine (Phila Pa 1976), 2002. **27**(20): p. 2193-204.
17. MacDonald, R.S. and C.M. Bell, *An open controlled assessment of osteopathic manipulation in nonspecific low-back pain.* Spine (Phila Pa 1976), 1990. **15**(5): p. 364-70.
18. Clark, B.C., et al., *Muscle functional magnetic resonance imaging and acute low back pain: a pilot study to characterize lumbar muscle activity asymmetries and examine the effects of osteopathic manipulative treatment.* Osteopath Med Prim Care, 2009. **3**: p. 7.
19. Cote, P., S.A. Mior, and H. Vernon, *The short-term effect of a spinal manipulation on pain/pressure threshold in patients with chronic mechanical low back pain.* J Manipulative Physiol Ther, 1994. **17**(6): p. 364-8.
20. Dishman, J.D., D.S. Greco, and J.R. Burke, *Motor-evoked potentials recorded from lumbar erector spinae muscles: a study of corticospinal excitability changes associated with spinal manipulation.* J Manipulative Physiol Ther, 2008. **31**(4): p. 258-70.
21. Howell, J.N., et al., *Stretch reflex and Hoffmann reflex responses to osteopathic manipulative treatment in subjects with Achilles tendinitis.* J Am Osteopath Assoc, 2006. **106**(9): p. 537-45.

22. Johansson, H. and P. Sojka, *Pathophysiological mechanisms involved in genesis and spread of muscular tension in occupational muscle pain and in chronic musculoskeletal pain syndromes: a hypothesis.* Med Hypotheses, 1991. **35**(3): p. 196-203.
23. Knutson, G.A., *The role of the gamma-motor system in increasing muscle tone and muscle pain syndromes: a review of the Johansson/Sojka hypothesis.* J Manipulative Physiol Ther, 2000. **23**(8): p. 564-72.
24. Korr, I.M., *Proprioceptors and somatic dysfunction.* J Am Osteopath Assoc, 1975. **74**(7): p. 638-50.
25. Matre, D.A., et al., *Experimental muscle pain increases the human stretch reflex.* Pain, 1998. **75**(2-3): p. 331-9.
26. Pickar, J.G., *Neurophysiological effects of spinal manipulation.* Spine J, 2002. **2**(5): p. 357-71.
27. Terrett, A.C. and H. Vernon, *Manipulation and pain tolerance. A controlled study of the effect of spinal manipulation on paraspinal cutaneous pain tolerance levels.* Am J Phys Med, 1984. **63**(5): p. 217-25.
28. Vernon, H.T., et al., *Pressure pain threshold evaluation of the effect of spinal manipulation in the treatment of chronic neck pain: a pilot study.* J Manipulative Physiol Ther, 1990. **13**(1): p. 13-6.
29. Pickar, J.G. and Y.M. Kang, *Paraspinal muscle spindle responses to the duration of a spinal manipulation under force control.* J Manipulative Physiol Ther, 2006. **29**(1): p. 22-31.
30. Pickar, J.G. and R.F. McLain, *Responses of mechanosensitive afferents to manipulation of the lumbar facet in the cat.* Spine (Phila Pa 1976), 1995. **20**(22): p. 2379-85.
31. Pickar, J.G., et al., *Response of lumbar paraspinal muscles spindles is greater to spinal manipulative loading compared with slower loading under length control.* Spine J, 2007. **7**(5): p. 583-95.
32. Pickar, J.G. and J.D. Wheeler, *Response of muscle proprioceptors to spinal manipulative-like loads in the anesthetized cat.* J Manipulative Physiol Ther, 2001. **24**(1): p. 2-11.
33. Sung, P.S., Y.M. Kang, and J.G. Pickar, *Effect of spinal manipulation duration on low threshold mechanoreceptors in lumbar paraspinal muscles: a preliminary report.* Spine (Phila Pa 1976), 2005. **30**(1): p. 115-22.
34. Bialosky, J.E., et al., *The mechanisms of manual therapy in the treatment of musculoskeletal pain: a comprehensive model.* Man Ther, 2009. **14**(5): p. 531-8.
35. Lund, J.P., et al., *The pain-adaptation model: a discussion of the relationship between chronic musculoskeletal pain and motor activity.* Can J Physiol Pharmacol, 1991. **69**(5): p. 683-94.
36. Travell, J.G., S. Rinzter, and M. Herman, *Pain and disability of the shoulder and arm.* J Am Med Assoc, 1942. **120**: p. 417-422.
37. van Dieen, J.H., L.P. Selen, and J. Cholewicki, *Trunk muscle activation in low-back pain patients, an analysis of the literature.* J Electromyogr Kinesiol, 2003. **13**(4): p. 333-51.
38. Ellestad, S.M., et al., *Electromyographic and skin resistance responses to osteopathic manipulative treatment for low-back pain.* J Am Osteopath Assoc, 1988. **88**(8): p. 991-7.
39. Lehman, G.J. and S.M. McGill, *Spinal manipulation causes variable spine kinematic and trunk muscle electromyographic responses.* Clin Biomech (Bristol, Avon), 2001. **16**(4): p. 293-9.
40. Krekoukias, G., N.J. Petty, and L. Cheek, *Comparison of surface electromyographic activity of erector spinae before and after the application of central posteroanterior mobilisation on the lumbar spine.* J Electromyogr Kinesiol, 2009. **19**(1): p. 39-45.
41. DeVocht, J.W., J.G. Pickar, and D.G. Wilder, *Spinal manipulation alters electromyographic activity of paraspinal muscles: a descriptive study.* J Manipulative Physiol Ther, 2005. **28**(7): p. 465-71.
42. Herzog, W., *Clinical Biomechanics of Spinal Manipulation*. 2000, New York: Churchill Livingstone.
43. Goss, D.A., J.S. Thomas, and B.C. Clark, *Novel methods for quantifying neurophysiologic properties of the human lumbar paraspinal muscles.* J Neurosci Methods, In Press.
44. Gillette, R.G., *A speculative argument for the coactivation of diverse somatic receptor populations by forceful chiropractic adjustments* Manual Med, 1987. **3**: p. 1-14.
45. Haldeman, S., *Spinal manipulative therapy in sports medicine.* Clin Sports Med, 1986. **5**: p. 277-293.
46. Raftis, K., *Spinal manipulation for back pain.* Hosp Pract, 1989. **15**: p. 95-108.

47. Reinert, O.C., *Fundamentals of chriopractic techniques*. 1983, Chesterfield, MO: Marian Press.
48. Zusman, M., *Spinal manipulative therapy: review of some proposed mechanisms, and a new hypothesis.* Aust J Physiother, 1986. **32**: p. 89-99.
49. Gouveia, L.O., P. Castanho, and J.J. Ferreira, *Safety of chiropractic interventions: a systematic review.* Spine, 2009. **34**(11): p. E405-13.
50. Powell, F.C., W.C. Hanigan, and W.C. Olivero, *A risk/benefit analysis of spinal manipulation therapy for relief of lumbar or cervical pain.* Neurosurgery, 1993. **33**(1): p. 73-8; discussion 78-9.
51. Devilly, G.J. and T.D. Borkovec, *Psychometric properties of the credibility/expectancy questionnaire.* J Behav Ther Exp Psychiatry, 2000. **31**(2): p. 73-86.
52. Plagenhoef, S., F.G. Evans, and T. Abdelnour, *Anatomical data for analyzing human motion.* Research Quarterly For Exercise and Sport, 1983. **54**(2): p. 169-178.
53. Thomas, J.S., D.M. Corcos, and Z. Hasan. *Kinematic rules underlying multi-joint reaching movements*. in *30th Annual Meeting. Society for Neuroscience*. 2000. New Orleans, LA.
54. Thomas, J.S., D.M. Corcos, and Z. Hasan, *Effect of movement speed on limb segment motions for reaching from a standing position.* Experimental Brain Research, 2003. **148**: p. 377-387.
55. Thomas, J.S. and C.R. France, *The relationship between pain-related fear and lumbar flexion during natural recovery from low back pain.* Eur Spine J, 2008. **17**(1): p. 97-103.
56. Trost, Z., et al., *Pain-related fear predicts reduced spinal motion following experimental back injury.* Pain, 2012.
57. Thomas, J.S., et al., *The influence of pain-related fear on peak muscle activity and force generation during maximal isometric trunk exertions.* Spine (Phila Pa 1976), 2008. **33**(11): p. E342-8.
58. Thomas, J.S., et al., *Effects of fear of movement on spine velocity and acceleration after recovery from low back pain.* Spine (Phila Pa 1976), 2008. **33**(5): p. 564-70.
59. Thomas, J.S., et al., *The effect of chronic low back pain on trunk muscle activations in target reaching movements with various loads.* Spine (Phila Pa 1976), 2007. **32**(26): p. E801-8.
60. Thomas, J.S. and C.R. France, *Pain-related fear is associated with avoidance of spinal motion during recovery from low back pain.* Spine (Phila Pa 1976), 2007. **32**(16): p. E460-6.
61. Clark, B.C., et al., *Neurophysiologic effects of spinal manipulation in patients with chronic low back pain.* BMC Musculoskelet Disord, 2011. **12**: p. 170.
62. Rubinstein, S.M., et al., *Spinal manipulative therapy for chronic low-back pain: an update of a Cochrane review.* Spine (Phila Pa 1976), 2011. **36**(13): p. E825-46.
63. Fritz, J.M. and S. George, *The use of a classification approach to identify subgroups of patients with acute low back pain. Interrater reliability and short-term treatment outcomes.* Spine (Phila Pa 1976), 2000. **25**(1): p. 106-14.
64. Maughan, E.F. and J.S. Lewis, *Outcome measures in chronic low back pain.* Eur Spine J, 2010. **19**(9): p. 1484-94.

Appendix: Supplemental

Specific Aims

While there is recent evidence that sagittal plane trunk stiffness is greater in men compared to women[65], the role of sex on dynamic trunk stiffness in LBP populations is unknown. Nor it is known if changes in trunk stiffness following spinal manipulative therapies is different for men and women. This is a substantial problem as conventional clinical wisdom suggests that manipulative therapies are often applied to address a “hypo-mobile” or “stiff” vertebral joint. In fact, Brodeur and colleagues reported differences in lumbar stiffness between male subjects with LBP and matched healthy controls.[66] Additionally, a longitudinal study of patients with sub-acute LBP and matched healthy controls found LBP subjects had an 8% decrease in intervertebral stiffness once they became asymptomatic.[67] However, it is unknown if an increase in stiffness at one or more vertebral segments translates to an increase in stiffness in the lumbar spine as a whole. Yet this would be completely consistent with the clinical perspective that manipulative therapies are aimed at reducing stiffness of individual segments (and possibly the trunk as a whole), with the goal of decreasing pain and improving function. In fact, recent findings indicate that trunk stiffness, assessed using a quasi-static method, is increased in subjects with LBP.[68, 69] Furthermore, Hodges and colleagues have recently suggested that an increase in dynamic trunk stiffness observed in subjects with recurrent LBP may, in part, account for the recurrence of LBP symptoms. While trunk stiffness has also been assessed in numerous movement tasks in healthy individuals,[70-72] no published studies have examined the influence of a course of manipulative therapies on trunk stiffness. Further, sex differences in trunk stiffness in the axial and coronal planes has not been examined in patients with chronic LBP patients who fit the clinical prediction rule for manipulation. Accordingly, we seek to examine the effects of sex on 1) trunk stiffness in response to multi-directional seated perturbations in participants with chronic LBP compared to matched healthy controls and 2) changes in trunk stiffness in response to multi-directional seated perturbations in participants with chronic LBP following an initial spinal manipulative treatment and following a 3-week course of treatment.

Passive trunk stiffness in three cardinal planes during unanticipated seated trunk perturbations will be measured in chronic LBP participants enrolled in the RELIEF study (Experiment 2) and a healthy control group (matched for gender, age, height, and weight)- recruited for the sex/gender differences supplement.

Specific Aim 1. To examine the effects of sex and chronic LBP on passive trunk stiffness. H1.1. Male participants with chronic LBP will present with increased stiffness compared female participants with chronic LBP. H1.2. Participants with chronic LBP will present with increased stiffness compared healthy controls.

Specific Aim 2. To examine sex differences in the effects of spinal manipulation on passive trunk stiffness. H2.1. There are no net sex effects of spinal manipulation on trunk stiffness. H2.2.Both manipulative interventions will decrease passive trunk stiffness as evidenced by the trunk’s initial mechanical response to multi-directional trunk perturbations.

Specific Aim 3. To examine the effects of sex and posture on trunk stiffness.

Specific Aim 4. To examine the effects of sex and trunk displacement from internally generated trunk perturbations.

Research Strategy

SIGNIFICANCE

Low back pain (LBP) is one of the most common reasons for seeking medical care and accounts for over 3.7 million physician visits per year in the United States alone. Ninety percent of adults will experience LBP in their lifetime, 50% will experience recurrent back pain, and 10% will develop chronic pain and related disability.[1, 4, 73, 74] According to the most recent national survey, more than 18 million Americans over the age of 18 years received manipulative therapies in 2007 at a total annual out of pocket cost of 3.9 billion dollars with back pain being the most common clinical complaint of these individuals.[5] While there is growing evidence for the efficacy of manipulative therapies to treat in LBP,[7-11] little is known on the functional biomechanical effects of these treatments. The lack of a mechanistic underpinning hinders acceptance by the wider scientific and health-care communities, and it also limits the development of rational strategies for using manipulative therapies. Further, we could find no published studies that have examined the influence of sex on trunk stiffness in participants with chronic LBP who fit the clinical prediction rule for manipulation or the acute and chronic effects of spinal manipulation on trunk stiffness. Accordingly, this proposed supplement is significant and of high scientific impact because it will provide critical information on the sex and functional biomechanical effects of the most common complementary and alternative therapies (manipulation and mobilization) used to treat LBP. This will provide an ideal expansion of the goals of the RELIEF study described above.

According to the most recent statistics from the U.S. National Health Interview Survey (NHIS), when collapsing across race and ethnicity, approximately 30.0% of women and 24.1% of men reported low back pain in the previous three months. (Summary Health Statistics for U.S. Adults: National Health Interview Survey, 2009, Table 10 http://www.cdc.gov/nchs/data/series/sr_10/sr10_249.pdf). These national statistics indicate that females account for a significantly greater percentage of cases of LBP compared to males. Thus, a further examination of potential sex differences in mechanisms of, and responses to treatment for LBP is needed.

Over the past decade there has been growing scientific evidence supporting the clinical effectiveness of both manipulation and mobilization therapies in treating LBP.[10-17] While considerable clinical evidence supporting the efficacy of these manual therapies has emerged, little scientific evidence has been offered to explain the effects and mechanisms underlying these treatments or the potential for sex specific differences in these mechanisms. Many scientists and clinicians have long-postulated that manipulative therapies exert influences on the functional biomechanics of the spine.[15, 39, 75-78] However, at present there are few empirical investigations that have examined the role of sex in trunk stiffness or response to manual therapies in patients with chronic LBP. Our **central hypothesis** is that spinal manipulative therapies will increase spine motion and reduce trunk stiffness assessed during to multi-directional seated trunk perturbation tasks and males with chronic LBP will present with greater trunk stiffness compared to females with chronic LBP.

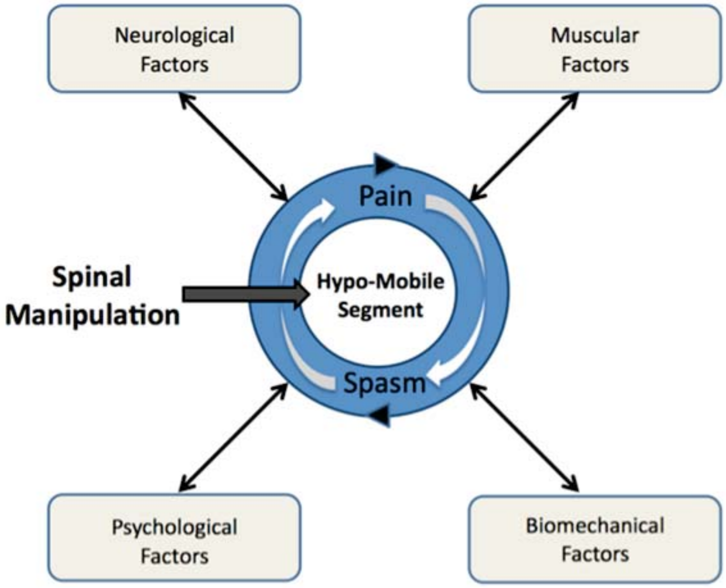

Figure 1. Manipulative therapies are typically used to treat the pain and spasm associated with a hypo-mobile spinal segment. However, the effects of manipulative therapies extend beyond the hypo-mobile segment, and likely interact and function to influence neurological, muscular, biomechanical, and psychological factors. The down stream effects could be a change in trunk stiffness that may influence recovery from an episode of LBP. However, it is unknown if spinal manipulation actually changes trunk stiffness (particularly in the axial and coronal planes). More importantly the role of sex in trunk stiffness or sex effects in the response to spinal manipulation treatments is unknown.

INNOVATION

Spinal manipulative therapies are applied in response to clinical findings -specifically to address "hypo-mobile" or stiff vertebral segments- and purport to exert influence on biomechanical spine function through modulation of muscle activity and joint range of motion [39] (**Figure 1**). It is unknown how the theoretical neurologic and muscular effects of manipulation influence the "downstream" spine biomechanics during suddenly applied perturbations. The role of sex on trunk stiffness in the three-cardinal planes has not been assessed nor, is it known whether the theoretical neuromuscular effects of manipulation actually influence trunk stiffness the same for male and females. It has recently been suggested that increased trunk stiffness may be an underlying mechanism in recurrent LBP.[69] This recent finding would be consistent with underlying clinical rationale for manipulative treatments that frequently focuses on reducing stiffness. Thus, this research implements the following innovative methodologies to examine the biomechanical effects of spinal manipulation in chronic LBP. Passive trunk stiffness in the three cardinal planes will be modeled using linear second order systems using our custom 3-D spine models. This research represents an innovative addition to a robust paradigm to assess the neuromuscular response of spinal manipulation to better understand the role of sex in one of the most common interventions for LBP.

APPROACH

1. Rationale. Conventional clinical wisdom suggests that manipulative therapies (manipulation and mobilization) are applied to address a "hypo-mobile" or "stiff" vertebral joint. In fact, Brodeur and colleagues reported differences in lumbar stiffness between male subjects with LBP and matched healthy controls.[66] Additionally, a longitudinal study of patients with sub-acute LBP and matched healthy

controls found LBP subjects had an 8% decrease in stiffness once they became asymptomatic.[67] However, it is unknown if an increase in stiffness at one or more vertebral segments translates to an increase in stiffness in the lumbar spine as a whole. Yet this would be completely consistent with the clinical perspective that manipulative therapies are aimed at reducing stiffness of individual segments (and possibly the trunk as a whole), with the goal of decreasing pain and improving function. While Gombatto and colleagues found an increase in trunk stiffness in participants with mechanical LBP, it is worth noting that trunk stiffness was determined from the slope of the force-displacement curve in a quasi-static condition. Thus static and not dynamic trunk stiffness was assessed in this cohort.[79] However, Hodges and colleagues have recently suggested that an increase in dynamic trunk stiffness occurs in subjects with recurrent LBP and may, in part, account for the recurrence of LBP symptoms. But this assessment was on asymptomatic participants (i.e., currently pain free, but a history of recurrent LBP) and was restricted to the sagittal plane. While trunk stiffness has also been assessed in numerous movement tasks in healthy individuals,[70-72] no published studies have examined the influence of a course of manipulative therapies on trunk stiffness. While there is recent evidence that sagittal plane trunk stiffness is greater in men compared to women, this has been only assessed in a paradigm that required active trunk co-contraction.[65] This is due, in part, to the use of a system that required a rigid linkage between the actuator and the trunk to assess trunk stiffness. The additional mass of the rigid link system reduces the sensitivity of the system to detect dynamic stiffness in a resting state. A significant advantage of our system to assess trunk stiffness is that light weight non-deformable Kevlar cables are used in place of a rigid linkage (see Figure 2A). This allows the subject to be released following a perturbation so that the actuator system is not part of their response, thereby enabling sex differences in dynamic trunk stiffness to be assessed with greater sensitivity. Further, trunk stiffness in the axial and coronal planes has not been examined in patients with chronic LBP. Interestingly, recent data suggests that males have greater sagittal-plane trunk stiffness compared to females,[65] but females have a higher prevalence of LBP. Thus an underlying mechanism of LBP may be different in males and females, or it could be due to the limitations of the system used to assess trunk stiffness. Accordingly, the proposed aims provide a robust method to determine the effects of sex and spinal manipulative therapies on trunk stiffness in response to multi-directional seated perturbations.

2. Assessing Trunk Stiffness

Assessing Trunk Stiffness Using Unpredictable Multi-directional Loads (Sitting): To quantify trunk stiffness in the three cardinal planes, we will use a linear second order system to model trunk stiffness in response to unpredictable multidirectional trunk loading. This methodology has been recently used by Hodges and colleagues to demonstrate that individuals with increased trunk stiffness are at greater risk for chronic and recurrent low back pain.[69] We will use our custom-built apparatus, illustrated in **Figure 2,** to measure stiffness and trunk muscle control patterns during multi-directional trunk loading. For these experiments, the subject is seated with the lumbar spine in a neutral position. The subject's pelvis is securely fastened using seatbelts that cross the mid thighs. A chest harness is snugly attached to the subject at the level of the 3rd thoracic vertebrae. As shown in Figure 2A, non-deformable Kevlar cables (1/4" diameter) are attached the 4 corners of harness by gimbals and run to the corners of the reference frame through a pulley system to attach to actuators set in force control mode. This will allow us to precisely control the load magnitude and duration of the perturbing force pulse. Participants sit in the reference frame with their arms held across their chest. To generate the loading force, the actuators are engaged in pairs (e.g. front, back, left side, right side) to induce a perturbation in one of the following directions: flexion, extension, side-bend left, side-bend right, rotation left, or rotation right. The actuators provide a rapid load pulse of sufficient magnitude to induce 2 inches of trunk displacement in 100-ms. Thus the applied force is proportional to the subject's anthropometric characteristics. Once the impulse load is delivered to the trunk, all 4 Kevlar cables go slack and trunk stiffness is determined from the kinematic responses. The low mass-design of this system will not only allow a simplified mechanical model in evaluating stiffness, but may also be more sensitive than designs that use a rigid linkage between the actuator and the trunk to manipulate the subjects. In this study, the forces will be delivered at random time intervals between 2 and 30 seconds. The onset of the force will

result in a small trunk angular displacement (5-10 degrees) and a long latency postural reflex response of the trunk muscles. In this paradigm, the participant will be exposed to 3 loads in each of the 6 directions listed above. The sequence of the load direction (e.g. flexion, extension, rotation right etc.) will be randomized for each participant.

Once data are collected, system properties of the trunk will be estimated from the time of onset of the perturbing forces until maximal trunk displacement. Effective trunk mass (M), damping (B), and stiffness (K) will be assumed to be constant after the perturbation and will be described by a second-order linear model (Equation 1).

$$F(t)=M^{*}\,Acceleration(t) + B^{*}Velocity(t) + K^{*}Position(t)$$

F(t) is the resultant force vector on the trunk. The change in trunk position will be measured using the Vicon system. Trunk velocity and acceleration will be determined using a Savitzky-Golay filter.[80] The force applied to the trunk during the perturbation will be measured from the pairs of load cells that are in-series with the actuators. Consistent with the methods proposed by Hodges and colleagues, M, B, and K will be assumed to be constant, and a standard least squares procedure will be used to solve the estimation. The data for the second order linear equation will be integrated numerically twice over time to increase the procedure's robustness.

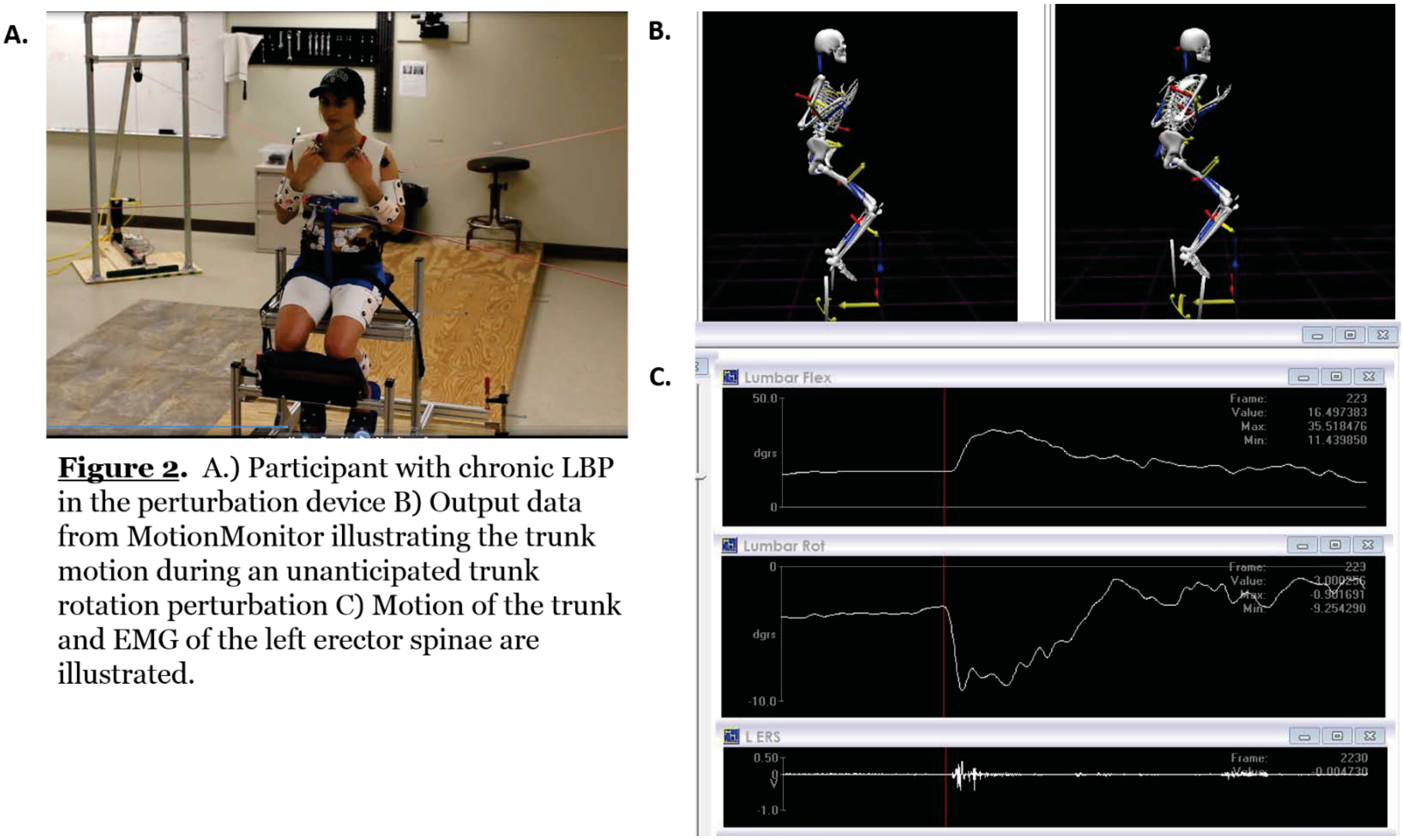

Figure 2. A.) Participant with chronic LBP in the perturbation device B) Output data from MotionMonitor illustrating the trunk motion during an unanticipated trunk rotation perturbation C) Motion of the trunk and EMG of the left erector spinae are illustrated.

<u>Assessing Trunk Stiffness Using Unpredictable Multi-directional Loads (Standing):</u>
The same paradigm described above will be repeated with the healthy participant in a standing posture. This will allow us to examine the effects of posture (i.e., sitting versus standing) and sex (male, female) on trunk stiffness in a healthy cohort.

<u>Assessing Trunk Displacement Using Internally Generated Trunk Perturbations</u>
To assess the effects of sex on trunk displacement with internally generated perturbations, participants will be asked to perform the following 3 rapid (duration of 100 ms) voluntary movements while in a standing posture: 1) flex both shoulders 90° to create a trunk flexion perturbation, 2) abduct the left shoulder 90° to create a trunk side-bending left perturbation, and 3) flex the right shoulder 90° while simultaneously extending the left shoulder 90° to generate a trunk rotation left perturbation. The participants will 6 trials of each of these tasks performed in a random order. To normalize the magnitude of the internally generated force, participants will perform the rapid shoulder movements with weights equal to 5% of body mass. Thus a participant weighing 120 lbs will hold a total of 6 lbs of weight to compete the task. The primary dependent measure is trunk displacement in the three-cardinal planes.

NUMBER OF SUBJECTS AND POWER

The primary dependent variable (DV) for the **Experiment 2** group in the RELIEF study is the short-latency stretch reflex amplitude. Our preliminary work indicates that spinal manipulation reduces stretch reflex amplitude in individuals with chronic LBP who exhibit an audible joint sound by 30% from 0.56 to 0.39.[61] In study participants with no audible joint sound, there was a non-significant 10% increase from 0.62 to 0.68. The baseline standard deviation was 0.25 for both groups. We assumed manipulation would result in a 25% reduction 2-days following a course of treatment and a 20% reduction 4-weeks post course of treatment. Given our pilot data indicating the importance of cavitation, we assumed smaller reductions in stretch reflex amplitudes at each time point for the joint mobilization treatment arm. (i.e., 28, 23, and 18% reductions at the three endpoints). Finally, we assumed that Sham Control would have the same mean (0.68) at each testing point. Based on these data, in order to achieve adequate power, Experiment 2 required a total of 60 participants with chronic LBP that met a modified clinical prediction rule for spinal manipulation. Due to the greater prevalence of LBP in women, our sample for Experiment 2 will consist of 33 females and 27 males.

To address aim 1, we will recruit 60 healthy participants with no history of LBP matched on gender, age (± 3 years), height (± 2 inches), and weight (± 10 lbs) to the LBP group. A recent paper by Vazirian et al reported large effects sizes (η^2=0.425) for sex differences in sagittal plane trunk stiffness in healthy participants.[65] Based on these data our sample of 120 participants (60 participants LBP, 60 matched controls) will provide power of 0.85 to determine an interaction of sex by back pain group for trunk stiffness at $p<0.05$.

Given that our initial power estimates for the parent R01 (i.e., RELIEF study) were designed to detect gender differences as well as treatment effects on trunk onset muscle latencies, and assuming similar effect sizes for our trunk stiffness measures, 60 participants with chronic LBP should provide sufficient power for the stiffness measures in aim 2 of this application.

SUBJECTS AND RECRUITMENT

The characteristics for participants in the RELIEF study are listed below. As noted above, we have already completed testing on 18 men (total n=27) and 31 women (total n=33) in Experiment 2 of the RELIEF study (i.e., parent R01). Once completed, the data collected during the seated trunk perturbations will be used to characterize sex differences in trunk stiffness in chronic LBP participants who fit a modified clinical prediction rule for spinal manipulation. These data will be compared to a sample of matched healthy controls. Finally, the influence of sex on changes in trunk stiffness in response to spinal manipulative therapies will be assessed.

Inclusion criteria

Participants must meet all of the following inclusion criteria to participate in this study:

- Between 18 to 45 years of age.
- Answer no to the following questions:
  - Have you had low back pain constantly or on most days for the last three months?
  - Has your back pain caused you to seek care or consultation from a health care provider?
- Average pain intensity, assessed using the Numerical Pain Rating (NPR) scale over the past week = 0 on a 0-10 numerical pain scale
- Roland Morris Disability Questionnaire score = 0
- Exhibit 3 of 4 of the following findings reported in the clinical prediction rules for spinal manipulation
  - No symptoms distal to the knee
  - FABQ work subscale score <19
  - At least 1 hypo-mobile lumbar spinal segment
  - At least 1 hip > 35 degrees internal rotation range of motion

For this supplemental grant application, we will recruit an additional 60 healthy participants with no history of LBP (i.e. no interruptions in play or work due to back pain, or visits to any healthcare provider secondary to back pain in the past 1 year) and who are matched on sex, age (± 3 years), height (± 2 inches), and weight (± 10 lbs) to the participants recruited into **Experiment 2** of the RELIEF study. We will apply the same exclusion criteria to the control group as is used for the RELIEF study LBP participants. See table below.

Exclusion criteria

Participants must not have any of the following exclusion criteria to participate in this study:

- Have a personal history of the following neurological disorders: Alzheimer's, Amyotrophic Lateral Sclerosis, Multiple Sclerosis, Parkinson's, or Stroke.
- Have a personal history of the following cardiorespiratory disorders: Congestive heart failure, Heart attack in past 24 months.
- Have a personal history of the following musculoskeletal disorders: Rheumatoid Arthritis, pathologic fractures of the spine, avascular necrosis or osteonecrosis, severe osteoarthritis. Including a history of spine surgery or a hip arthroplasty.
- Have active cancer.
- Be Blind.
- Have used narcotics or muscle relaxants within 30 days prior to study enrollment.
- Report being pregnant.
- Have a body mass index greater than 35 kg/m^2.
- Have clinical depression (i.e., subjects who score 24 or higher on the Center for Epidemiology Depression Scale).
- Current drug or alcohol use or dependence that, in the opinion of the PIs, would interfere with adherence to study requirements

Hypothesis Testing: Aim 1

Hypothesis 1.1. Male participants with chronic LBP will present with increased stiffness compared female participants with chronic LBP

Hypothesis 1.2. Participants with chronic LBP will present with increased stiffness compared healthy controls.

Separate 2 Group (Chronic LBP, Matched Healthy Control) x 2 Sex (Male, Female) mixed-model MANOVAs will be conducted on trunk stiffness measures.

Hypothesis Testing: Aim 2

Hypothesis 2.1. There are no net sex effects of spinal manipulation on trunk stiffness.

Hypothesis 2.2. Both manipulative interventions will decrease passive trunk stiffness as evidenced by the trunk's initial mechanical response to multi-directional trunk perturbations.

Separate 3 Group (Manipulation, Mobilization, Sham Laser) x 2 Gender(Male, Female) x 3 Time (Baseline, Immediately following initial treatment, 48 hrs post-3 weeks of treatment) mixed-model MANOVAs will be conducted on trunk stiffness measures.

Specific Aim 3. To examine the effects of sex and posture on trunk stiffness.

Hypothesis 3.1. Healthy Male participants will present with increased trunk stiffness compared to healthy female participants.

Hypothesis 3.2. Trunk stiffness will be greater in sitting compared to standing.

Separate 2 Sex (Male, Female) x 2 Posture (Male, Female) mixed-model MANOVAs will be conducted on trunk stiffness measures.

Specific Aim 4. To examine the effects of sex and trunk displacement from internally generated trunk perturbations.

Hypothesis 4.1. Healthy Male participants will present with less trunk displacement during internally generated trunk perturbations compared to healthy female participants.

Separate 2 Sex (Male, Female) mixed-model MANOVAs will be conducted on trunk displacements.

Timeline

Tasks	Q1	Q2	Q3	Q4
Aims 1, 3,4	Develop stiffness models<br><br>Begin recruiting matched healthy controls	Enroll & test matched healthy controls<br><br>Complete testing for Experiment 2 RELIEF study	Data analyses	Manuscript generation
Aim 2	Complete testing for Experiment 2 RELIEF study		Data analyses	Manuscript generation

Summary

The research represents a critical assessment of sex differences in trunk stiffness assessed in the three cardinal planes. Our low-mass system allows us to assess dynamic trunk stiffness with great sensitivity and this will be the first study to examine sex differences in dynamic stiffness in a cohort of chronic LBP sufferers who meet a clinical prediction for spinal manipulation (i.e., have a clinician identified hypo-mobile segment). We are uniquely positioned to provide the first and most comprehensive study of sex differences in a known factor in recurrent and chronic LBP. This research has a strong potential to positively impact public health by providing data required to develop innovative, individualized treatments to prevent recurrent low back pain and related disability.

References

1. Andersson, G.B., *Epidemiological features of chronic low-back pain*. Lancet, 1999. **354**(9178): p. 581-5.
2. Carey, T.S., J.M. Garrett, and A.M. Jackman, *Beyond the good prognosis. Examination of an inception cohort of patients with chronic low back pain*. Spine, 2000. **25**(1): p. 115-20.
3. Klenerman, L., et al., *The prediction of chronicity in patients with an acute attack of low back pain in a general practice setting*. Spine, 1995. **20**(4): p. 478-84.
4. Von Korff, M., *Studying the natural history of back pain*. Spine (Phila Pa 1976), 1994. **19**(18 Suppl): p. 2041S-2046S.
5. Nahin, R.L., et al., *Costs of complementary and alternative medicine (CAM) and frequency of visits to CAM practitioners: United States, 2007*. Natl Health Stat Report, 2009(18): p. 1-14.
6. Cleland, J.A., et al., *The audible pop from thoracic spine thrust manipulation and its relation to short-term outcomes in patients with neck pain*. J Man Manip Ther, 2007. **15**(3): p. 143-54.
7. Assendelft, W.J., et al., *Spinal manipulative therapy for low back pain*. Cochrane Database Syst Rev, 2004(1): p. CD000447.
8. Bronfort, G., et al., *Efficacy of spinal manipulation and mobilization for low back pain and neck pain: a systematic review and best evidence synthesis*. Spine J, 2004. **4**(3): p. 335-56.
9. Childs, J.D., et al., *A clinical prediction rule to identify patients with low back pain most likely to benefit from spinal manipulation: a validation study*. Ann Intern Med, 2004. **141**(12): p. 920-8.
10. Licciardone, J.C., A.K. Brimhall, and L.N. King, *Osteopathic manipulative treatment for low back pain: a systematic review and meta-analysis of randomized controlled trials*. BMC Musculoskelet Disord, 2005. **6**: p. 43.
11. Licciardone, J.C., et al., *Osteopathic manipulative treatment for chronic low back pain: a randomized controlled trial*. Spine (Phila Pa 1976), 2003. **28**(13): p. 1355-62.
12. Gatterman, M.I., et al., *Rating specific chiropractic technique procedures for common low back conditions*. J Manipulative Physiol Ther, 2001. **24**(7): p. 449-56.
13. Haas, M., E. Groupp, and D.F. Kraemer, *Dose-response for chiropractic care of chronic low back pain*. Spine J, 2004. **4**(5): p. 574-83.
14. Hoiriis, K.T., et al., *A randomized clinical trial comparing chiropractic adjustments to muscle relaxants for subacute low back pain*. J Manipulative Physiol Ther, 2004. **27**(6): p. 388-98.
15. Hondras, M.A., et al., *A randomized controlled trial comparing 2 types of spinal manipulation and minimal conservative medical care for adults 55 years and older with subacute or chronic low back pain*. J Manipulative Physiol Ther, 2009. **32**(5): p. 330-43.
16. Hurwitz, E.L., et al., *A randomized trial of medical care with and without physical therapy and chiropractic care with and without physical modalities for patients with low back pain: 6-month follow-up outcomes from the UCLA low back pain study*. Spine (Phila Pa 1976), 2002. **27**(20): p. 2193-204.
17. MacDonald, R.S. and C.M. Bell, *An open controlled assessment of osteopathic manipulation in nonspecific low-back pain*. Spine (Phila Pa 1976), 1990. **15**(5): p. 364-70.
18. Clark, B.C., et al., *Muscle functional magnetic resonance imaging and acute low back pain: a pilot study to characterize lumbar muscle activity asymmetries and examine the effects of osteopathic manipulative treatment*. Osteopath Med Prim Care, 2009. **3**: p. 7.
19. Cote, P., S.A. Mior, and H. Vernon, *The short-term effect of a spinal manipulation on pain/pressure threshold in patients with chronic mechanical low back pain*. J Manipulative Physiol Ther, 1994. **17**(6): p. 364-8.
20. Dishman, J.D., D.S. Greco, and J.R. Burke, *Motor-evoked potentials recorded from lumbar erector spinae muscles: a study of corticospinal excitability changes associated with spinal manipulation*. J Manipulative Physiol Ther, 2008. **31**(4): p. 258-70.

21. Howell, J.N., et al., *Stretch reflex and Hoffmann reflex responses to osteopathic manipulative treatment in subjects with Achilles tendinitis.* J Am Osteopath Assoc, 2006. **106**(9): p. 537-45.
22. Johansson, H. and P. Sojka, *Pathophysiological mechanisms involved in genesis and spread of muscular tension in occupational muscle pain and in chronic musculoskeletal pain syndromes: a hypothesis.* Med Hypotheses, 1991. **35**(3): p. 196-203.
23. Knutson, G.A., *The role of the gamma-motor system in increasing muscle tone and muscle pain syndromes: a review of the Johansson/Sojka hypothesis.* J Manipulative Physiol Ther, 2000. **23**(8): p. 564-72.
24. Korr, I.M., *Proprioceptors and somatic dysfunction.* J Am Osteopath Assoc, 1975. **74**(7): p. 638-50.
25. Matre, D.A., et al., *Experimental muscle pain increases the human stretch reflex.* Pain, 1998. **75**(2-3): p. 331-9.
26. Pickar, J.G., *Neurophysiological effects of spinal manipulation.* Spine J, 2002. **2**(5): p. 357-71.
27. Terrett, A.C. and H. Vernon, *Manipulation and pain tolerance. A controlled study of the effect of spinal manipulation on paraspinal cutaneous pain tolerance levels.* Am J Phys Med, 1984. **63**(5): p. 217-25.
28. Vernon, H.T., et al., *Pressure pain threshold evaluation of the effect of spinal manipulation in the treatment of chronic neck pain: a pilot study.* J Manipulative Physiol Ther, 1990. **13**(1): p. 13-6.
29. Pickar, J.G. and Y.M. Kang, *Paraspinal muscle spindle responses to the duration of a spinal manipulation under force control.* J Manipulative Physiol Ther, 2006. **29**(1): p. 22-31.
30. Pickar, J.G. and R.F. McLain, *Responses of mechanosensitive afferents to manipulation of the lumbar facet in the cat.* Spine (Phila Pa 1976), 1995. **20**(22): p. 2379-85.
31. Pickar, J.G., et al., *Response of lumbar paraspinal muscles spindles is greater to spinal manipulative loading compared with slower loading under length control.* Spine J, 2007. **7**(5): p. 583-95.
32. Pickar, J.G. and J.D. Wheeler, *Response of muscle proprioceptors to spinal manipulative-like loads in the anesthetized cat.* J Manipulative Physiol Ther, 2001. **24**(1): p. 2-11.
33. Sung, P.S., Y.M. Kang, and J.G. Pickar, *Effect of spinal manipulation duration on low threshold mechanoreceptors in lumbar paraspinal muscles: a preliminary report.* Spine (Phila Pa 1976), 2005. **30**(1): p. 115-22.
34. Bialosky, J.E., et al., *The mechanisms of manual therapy in the treatment of musculoskeletal pain: a comprehensive model.* Man Ther, 2009. **14**(5): p. 531-8.
35. Lund, J.P., et al., *The pain-adaptation model: a discussion of the relationship between chronic musculoskeletal pain and motor activity.* Can J Physiol Pharmacol, 1991. **69**(5): p. 683-94.
36. Travell, J.G., S. Rinzter, and M. Herman, *Pain and disability of the shoulder and arm.* J Am Med Assoc, 1942. **120**: p. 417-422.
37. van Dieen, J.H., L.P. Selen, and J. Cholewicki, *Trunk muscle activation in low-back pain patients, an analysis of the literature.* J Electromyogr Kinesiol, 2003. **13**(4): p. 333-51.
38. Ellestad, S.M., et al., *Electromyographic and skin resistance responses to osteopathic manipulative treatment for low-back pain.* J Am Osteopath Assoc, 1988. **88**(8): p. 991-7.
39. Lehman, G.J. and S.M. McGill, *Spinal manipulation causes variable spine kinematic and trunk muscle electromyographic responses.* Clin Biomech (Bristol, Avon), 2001. **16**(4): p. 293-9.
40. Krekoukias, G., N.J. Petty, and L. Cheek, *Comparison of surface electromyographic activity of erector spinae before and after the application of central posteroanterior mobilisation on the lumbar spine.* J Electromyogr Kinesiol, 2009. **19**(1): p. 39-45.
41. DeVocht, J.W., J.G. Pickar, and D.G. Wilder, *Spinal manipulation alters electromyographic activity of paraspinal muscles: a descriptive study.* J Manipulative Physiol Ther, 2005. **28**(7): p. 465-71.
42. Herzog, W., *Clinical Biomechanics of Spinal Manipulation.* 2000, New York: Churchill Livingstone.
43. Goss, D.A., J.S. Thomas, and B.C. Clark, *Novel methods for quantifying neurophysiologic properties of the human lumbar paraspinal muscles.* J Neurosci Methods, In Press.
44. Gillette, R.G., *A speculative argument for the coactivation of diverse somatic receptor populations by forceful chiropractic adjustments* Manual Med, 1987. **3**: p. 1-14.

45. Haldeman, S., *Spinal manipulative therapy in sports medicine.* Clin Sports Med, 1986. **5**: p. 277-293.
46. Raftis, K., *Spinal manipulation for back pain.* Hosp Pract, 1989. **15**: p. 95-108.
47. Reinert, O.C., *Fundamentals of chriopractic techniques*. 1983, Chesterfield, MO: Marian Press.
48. Zusman, M., *Spinal manipulative therapy: review of some proposed mechanisms, and a new hypothesis.* Aust J Physiother, 1986. **32**: p. 89-99.
49. Gouveia, L.O., P. Castanho, and J.J. Ferreira, *Safety of chiropractic interventions: a systematic review*. Spine, 2009. **34**(11): p. E405-13.
50. Powell, F.C., W.C. Hanigan, and W.C. Olivero, *A risk/benefit analysis of spinal manipulation therapy for relief of lumbar or cervical pain.* Neurosurgery, 1993. **33**(1): p. 73-8; discussion 78-9.
51. Devilly, G.J. and T.D. Borkovec, *Psychometric properties of the credibility/expectancy questionnaire.* J Behav Ther Exp Psychiatry, 2000. **31**(2): p. 73-86.
52. Plagenhoef, S., F.G. Evans, and T. Abdelnour, *Anatomical data for analyzing human motion.* Research Quarterly For Exercise and Sport, 1983. **54**(2): p. 169-178.
53. Thomas, J.S., D.M. Corcos, and Z. Hasan. *Kinematic rules underlying multi-joint reaching movements.* in *30th Annual Meeting. Society for Neuroscience.* 2000. New Orleans, LA.
54. Thomas, J.S., D.M. Corcos, and Z. Hasan, *Effect of movement speed on limb segment motions for reaching from a standing position.* Experimental Brain Research, 2003. **148**: p. 377-387.
55. Thomas, J.S. and C.R. France, *The relationship between pain-related fear and lumbar flexion during natural recovery from low back pain.* Eur Spine J, 2008. **17**(1): p. 97-103.
56. Trost, Z., et al., *Pain-related fear predicts reduced spinal motion following experimental back injury*. Pain, 2012.
57. Thomas, J.S., et al., *The influence of pain-related fear on peak muscle activity and force generation during maximal isometric trunk exertions.* Spine (Phila Pa 1976), 2008. **33**(11): p. E342-8.
58. Thomas, J.S., et al., *Effects of fear of movement on spine velocity and acceleration after recovery from low back pain.* Spine (Phila Pa 1976), 2008. **33**(5): p. 564-70.
59. Thomas, J.S., et al., *The effect of chronic low back pain on trunk muscle activations in target reaching movements with various loads.* Spine (Phila Pa 1976), 2007. **32**(26): p. E801-8.
60. Thomas, J.S. and C.R. France, *Pain-related fear is associated with avoidance of spinal motion during recovery from low back pain.* Spine (Phila Pa 1976), 2007. **32**(16): p. E460-6.
61. Clark, B.C., et al., *Neurophysiologic effects of spinal manipulation in patients with chronic low back pain.* BMC Musculoskelet Disord, 2011. **12**: p. 170.
62. Rubinstein, S.M., et al., *Spinal manipulative therapy for chronic low-back pain: an update of a Cochrane review*. Spine (Phila Pa 1976), 2011. **36**(13): p. E825-46.
63. Fritz, J.M. and S. George, *The use of a classification approach to identify subgroups of patients with acute low back pain. Interrater reliability and short-term treatment outcomes.* Spine (Phila Pa 1976), 2000. **25**(1): p. 106-14.
64. Maughan, E.F. and J.S. Lewis, *Outcome measures in chronic low back pain.* Eur Spine J, 2010. **19**(9): p. 1484-94.
65. Vazirian, M., et al., *Age-related differences in trunk intrinsic stiffness.* J Biomech, 2015.
66. Brodeur, R.R. and L. DelRe, *Stiffness of the thoracolumbar spine for subjects with and without low back pain.* Journal of Neuromusculoskeletal Systems, 1999. **7**(4): p. 123-133.
67. Latimer, J., et al., *An investigation of the relationship between low back pain and lumbar posteroanterior stiffness.* J Manipulative Physiol Ther, 1996. **19**(9): p. 587-91.
68. Gombatto, S.P., et al., *Differences in symmetry of lumbar region passive tissue characteristics between people with and people without low back pain.* Clin Biomech (Bristol, Avon), 2008. **23**(8): p. 986-95.
69. Hodges, P., et al., *Changes in the mechanical properties of the trunk in low back pain may be associated with recurrence.* J Biomech, 2009. **42**(1): p. 61-6.

70. McGill, S., J. Seguin, and G. Bennett, *Passive stiffness of the lumbar torso in flexion, extension, lateral bending, and axial rotation. Effect of belt wearing and breath holding*. Spine (Phila Pa 1976), 1994. **19**(6): p. 696-704.
71. Parkinson, R.J., T.A. Beach, and J.P. Callaghan, *The time-varying response of the in vivo lumbar spine to dynamic repetitive flexion*. Clin Biomech (Bristol, Avon), 2004. **19**(4): p. 330-6.
72. Brown, S.H., M.L. Haumann, and J.R. Potvin, *The responses of leg and trunk muscles to sudden unloading of the hands: implications for balance and spine stability*. Clin Biomech (Bristol, Avon), 2003. **18**(9): p. 812-20.
73. Carey, T.S., J.M. Garrett, and A.M. Jackman, *Beyond the good prognosis. Examination of an inception cohort of patients with chronic low back pain*. Spine (Phila Pa 1976), 2000. **25**(1): p. 115-20.
74. Klenerman, L., et al., *The prediction of chronicity in patients with an acute attack of low back pain in a general practice setting*. Spine (Phila Pa 1976), 1995. **20**(4): p. 478-84.
75. Pope, M.H., et al., *A prospective randomized three-week trial of spinal manipulation, transcutaneous muscle stimulation, massage and corset in the treatment of subacute low back pain*. Spine (Phila Pa 1976), 1994. **19**(22): p. 2571-7.
76. Keller, T.S., et al., *Three-dimensional vertebral motions produced by mechanical force spinal manipulation*. J Manipulative Physiol Ther, 2006. **29**(6): p. 425-36.
77. Kulig, K., R. Landel, and C.M. Powers, *Assessment of lumbar spine kinematics using dynamic MRI: a proposed mechanism of sagittal plane motion induced by manual posterior-to-anterior mobilization*. J Orthop Sports Phys Ther, 2004. **34**(2): p. 57-64.
78. Triano, J.J., *Biomechanics of spinal manipulative therapy*. Spine J, 2001. **1**(2): p. 121-30.
79. Gombatto, S.P., et al., *Validity and reliability of a system to measure passive tissue characteristics of the lumbar region during trunk lateral bending in people with and people without low back pain*. J Rehabil Res Dev, 2008. **45**(9): p. 1415-29.
80. Press, W.H., et al., *Numerical Recipes in FORTRAN. The art of scientific computing*. second ed. 1992, New York: Cambridge University Press.

THE RELIEF STUDY
RESEARCHING THE EFFECTIVENESS OF LUMBAR INTERVENTIONS FOR ENHANCING FUNCTION

HEALTHY CONTROL

Ohio University Consent Form

Title of Research: Researching the Effectiveness of Lumbar Interventions for Enhancing Function Study (The RELIEF Study)
Researchers: James S. Thomas, P.T., Ph.D. and Brian C. Clark, Ph.D.
Study Sponsor: The National Institutes of Health's (NIH) National Center for Complementary and Integrative Medicine (NCCIH)
Grant Number: NIH R01AT006978

You are being asked to participate in a research study. For you to be able to decide whether or not you want to participate in this research, you should understand what the study is about, as well as the possible risks and benefits in order to make an informed decision. This process is known as informed consent. This form describes the purpose, procedures, possible benefits, and risks. It also explains how your personal information will be used and protected. Once you have read this form and your questions about the study are answered, you will be asked to sign it. This will allow your participation in this study. You should receive a copy of this document to take with you.

Explanation of Study

We want to find out if men and women have different responses expected and unexpected movements of the torso, and, as part of a larger study being done, we want to find out what types of interventions work best to treat low back pain.

If you agree to participate in this study, you will undergo screening tests to determine if you are eligible for the study. During this screening visit you will complete a series of surveys and undergo a physical exam where we will examine your health history and your back. Your participation in the study may end if you are found not to meet the entrance criteria that are set forth for all people in this study. If you are eligible to proceed in the study after screening, you will complete more surveys and undergo laboratory testing.

To participate in the study you must be between the ages of 18-45 years, and currently not have back pain or have had any back pain in the last 6 months that required treatment. Additionally, you cannot currently be receiving any medical treatment for your back pain. You should not participate in this study if you: 1) have ever had a surgery on your spine, 2) are using narcotic medications or muscle relaxants for pain, 3) are currently pregnant, could be pregnant, or are planning on becoming pregnant, 4) have a history of weak bones (examples: osteoporosis, spine fractures), 5) are currently running a fever, 6) have any recent unexplained weight loss, or 7) are unwilling to postpone the use of any other medical interventions and/or treatments for your low back pain except those provided in the study (with the exception of Tylenol/ibuprofen or heat/ice taken for pain as needed). If you become pregnant during the study please inform the investigators immediately. If you do become pregnant your participation in the research will be terminated.

Your participation in the study will last approximately 3-hours and require a total of 1 visit to Ohio University.

Laboratory Testing

Your laboratory testing session will occur in the Motor Control Lab at Ohio University located in Grover Center. During this testing you will have the electrical activity of your back muscles examined in response to movement.

Core Muscle Activation Test (CMAT): During this we will measure the electrical activity of your back muscles by placing sticky electrodes on the skin overlying your back muscles. To make sure we are able to record these signals we will

shave your skin and clean your skin using a mild skin abrasive and skin cleansing alcohol pad. Then, special light-weight sensors used to track movement will be attached to your arms, legs, and torso. The markers are attached directly to the skin using a series of elastic cuffs. Next, you will be seated in an upright posture in a custom designed chair. A chest harness will be attached to your torso with Velcro straps and your pelvis will be securely fastened using seatbelts that cross your mid-thigh and pelvis. Cables will be attached from the 4 corners of the torso harness to the puller devices located at the 4 the corners of the lab. These pullers make up the core muscle activation tester. You will be asked to sit with your arms held across your chest. The cables that go to each puller will be tensioned to about 5 lbs. of force and then the lines will be tugged in pairs. This will result in a pulling force that will cause your torso to be tugged 2-3 inches in about 1/10 of a second. The pulling force will result in your torso moving either forwards, backwards, twisting left, or twisting right. Next, the same procedure will be repeated in a standing posture. Finally you will be asked to perform several shoulder movements while holding light weights.

Risks and Discomforts

Risks or discomforts that you might experience from the lab testing procedures are as follows: The cleaning and preparation of the skin for the electrodes used to measure muscle activity may cause a skin irritation. This irritation generally subsides after a few days. The torso responses to the sudden pulling could cause back pain symptoms. However, we have chosen small pulling distances that should not result in painful spine motion. We also minimize risk by allowing you to stop at any time if you find the procedure too uncomfortable. Any irritation would be expected to subside in 24-48 hours. You could also experience some minor muscle soreness from the active shoulder movements.

NOTE: You have the right to stop your participation in this investigation at any time.

What We Will Do In the Event of a Medical Emergency: If you experience a medical emergency during a testing or intervention session that you or we feel requires immediate medical attention (e.g., a seizure) we will alert the Ohio University Campus Police and Safety Personnel.

What to Do and Who to Contact if You Have an Adverse Event: If you experience substantial pain during the study or if symptoms exist following completion of the study please contact James Thomas, P.T., Ph.D. at 740-593-4178 or Brian Clark, Ph.D. at 740-593-2354. In the event of a medical emergency please call 911.

Benefits

This study is important to science and society because low back pain affects 8 in 10 Americans and determining the comparative effectiveness of medical treatments could greatly improve clinical practice.

Confidentiality and Records

Your study information will be kept confidential by the investigators involved in this study. Specifically, only the investigators in the study will have access to your data. Published or presented data will not identify you in any way. You will not be audiotaped or videotaped. All medical history files will be kept in a locked file cabinet located in our office facilities. Once this research has been completed all files will be kept for an additional five years and then destroyed. Additionally, while every effort will be made to keep your study-related information confidential, there may be circumstances where this information must be shared with: 1) Federal agencies, for example the Office of Human

THE RELIEF STUDY
RESEARCHING THE EFFECTIVENESS OF LUMBAR INTERVENTIONS FOR ENHANCING FUNCTION

HEALTHY CONTROL

Research Protections, whose responsibility is to protect human subjects in research or the National Institutes of Health's National Center for Complementary and Integrative Health or its designees; and 2) Representatives of Ohio University (OU), including the Institutional Review Board, a committee that oversees the research at OU and 3) The RELIEF Study Independent Monitoring Committee, a committee that monitors the data and safety of the research.

Compensation You will be paid a total of $40 for your participation in this study. This compensation is to offset the time and inconvenience that study participation imposes. We will have to provide your social security number to OU's Finance department.

Contact Information
If you have any questions regarding this study, please contact James Thomas, P.T., Ph.D. at 740-593-4178, Brian Clark, Ph.D. at 740-593-2354 or Rebecca Batey, Study Coordinator, at 740-566-7246. In the event of a medical emergency please call 911. Additionally, if symptoms exist following completion of the study please contact Drs. Thomas or Clark.

If you have any questions regarding your rights as a research participant, please contact Dr. Chris Hayhow, Director of Research Compliance, at Ohio University, at (740) 593-0664, or email (hayhow@ohio.edu).

By signing below, you are agreeing that:

- you have read this consent form (or it has been read to you) and have been given the opportunity to ask questions and have them answered.
- you have been informed of potential risks and they have been explained to your satisfaction.
- you understand that illness or physical injuries not yet identified could occur as part of this research study.
- you understand Ohio University has no funds set aside for any injuries you might receive as a result of participating in this study.
- you are 18 years of age or older.
- your participation in this research is completely voluntary.
- you may leave the study at any time. If you decide to stop participating in the study, there will be no penalty to you and you will not lose any benefits to which you are otherwise entitled.

Participant Signature_______________________________________ Date________________

Printed Name____

Study Staff Signature_______________________________________ Date________________

Printed Name____

Ohio University Consent Form

Title of Research: Researching the Effectiveness of Lumbar Interventions for Enhancing Function Study (The RELIEF Study)
Researchers: James S. Thomas, P.T., Ph.D. and Brian C. Clark, Ph.D.
Study Sponsor: The National Institutes of Health's (NIH) National Center for Complementary and Integrative Medicine (NCCIH)
Grant Number: NIH R01AT006978

You are being asked to participate in a research study. For you to be able to decide whether or not you want to participate in this research, you should understand what the study is about, as well as the possible risks and benefits in order to make an informed decision. This process is known as informed consent. This form describes the purpose, procedures, possible benefits, and risks. It also explains how your personal information will be used and protected. Once you have read this form and your questions about the study are answered, you will be asked to sign it. This will allow your participation in this study. You should receive a copy of this document to take with you.

Explanation of Study

This study is being done because we want to find out what types of interventions work best to treat low back pain, and we want to determine how these interventions work.

If you agree to participate in this study, you will undergo screening tests to determine if you are eligible for the study. During this screening visit you will complete a series of surveys and undergo a physical exam where we will examine your health history and your back. This visit will take up to two hours. Your participation in the study may end if you are found not to meet the entrance criteria that are set forth for all people in this study. If you are eligible to proceed in the study after screening, you will be randomly assigned to one of three intervention groups. Random assignment means that your assignment is determined by chance, like flipping a coin. In addition to the intervention group that you are assigned to you will be assigned to one of three groups that differ based on the types of laboratory measurements that are assessed before, during and after the intervention. The assignment to the laboratory measurement group is not random, but will rather depend on scheduling availability of different laboratories as well as your medical history. You will not be able to choose one intervention group or one laboratory measurement group over the other.

The three interventions that you could receive, along with a description of the types of laboratory measurements that could be assessed, are described below. You should understand that one of the interventions in the study is a placebo intervention and that you could be randomly assigned to receive that intervention. You have about a 67 percent chance of receiving an experimental intervention and about a 33 percent chance of receiving a placebo intervention. You will not know which of the interventions you have received until all participants in this study have completed their interventions.

To participate in the study you must be between the ages of 18-45 years, and currently have back pain with symptoms lasting greater than 12 weeks. Additionally, you cannot currently be receiving any medical treatment for your back pain. You should not participate in this study if you: 1) have ever had a surgery on your spine, 2) are using narcotic medications or muscle relaxants for pain, 3) are currently pregnant, could be pregnant, or are planning on becoming pregnant, 4) have a history of weak bones (examples: osteoporosis, spine fractures), 5) are currently running a fever, 6) have any recent unexplained weight loss, or 7) are unwilling to postpone the use of any other medical interventions and/or treatments for your low back pain except those provided in the study (with the exception of Tylenol/ibuprofen or heat/ice taken for pain as needed). If you become pregnant during the study please inform the investigators immediately. If you do become pregnant your participation in the research will be terminated.

Your participation in the study will last approximately 2 months and require a total of 9 visits to Ohio University. Some of these visits will involve you undergoing laboratory tests (testing sessions), and during some visits you will receive an intervention for your low back pain (intervention session). The interventions will occur over a 3-week period (two visits/week). Before and after each intervention session, you will be asked to complete some brief surveys about your low back pain. Each laboratory testing session will require approximately 1-2 hours of your time. Each intervention session will require about 15-30 minutes of your time. Here is a diagram showing when you will be required to visit Ohio University:

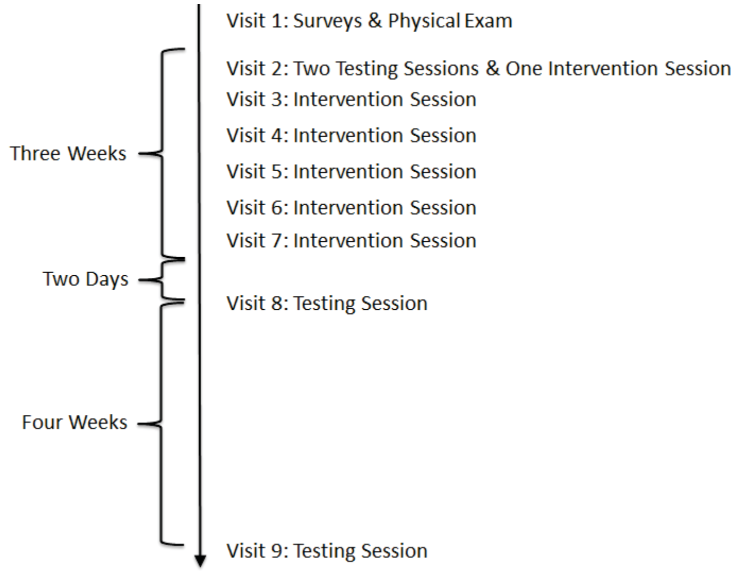

Please note that in visit 2 you will undergo a lab testing session followed by an intervention and then have the lab testing session repeated. The intervention will be delivered in a room located very close to the laboratory (within ~ 100 feet), so, it only requires one visit to the testing site. This visit could take up to 3 hours. You will be notified when your participation has ended.

Interventions You Could Receive

As mentioned previously you will be randomly assigned to receive one of three types of interventions for your low back pain. The different interventions are described below:

Manipulation- The practitioner will carefully position you on a table on your side and then deliver a quick, short, controlled movement of the shoulder, arm, and hand to provide a quick stretch of the lumbar spine that may or may not include a popping noise from your back.

Mobilization- The practitioner will carefully position you on your side on a table and then have you perform a series of gentle contractions of the torso muscles against the resistance provide by the practitioner. You may or may not hear a popping noise from you back during these procedures.

Laser- The practitioner will carefully position you on your side on a table. You will be given a pair of protective eye goggles to wear during the laser intervention. The practitioner will place the laser apparatus on your lower back region for the intervention.

Laboratory Tests That You Could Undergo

As mentioned previously you will be randomly assigned to one of three groups that have different laboratory-based measurements assessed. These different laboratory test groups are described below:

I. Muscular Testing - Lab Test Group I

If you are assigned to this group your laboratory testing sessions will occur in the Ohio Musculoskeletal and Neurological Institute's Medical Imaging Core facility at Ohio University located in the Stuckey Academic & Research Center. During these testing sessions you will complete a series of surveys about your low back pain and undergo an MRI (described below).

Magnetic Resonance Imaging (MRI): You will have an MRI, which provides an image or picture, of your lower back. For the MRI, you will be asked to wear clothing that contains no metal objects and to lie still in the MRI for around 30-40 minutes. You should know that these images are obtained for research purposes only and they will not routinely be reviewed by a physician to determine if pathology (e.g., cancer, slipped disk, etc.) is present. If you are a female, you will be asked to take a urine pregnancy test immediately before your MRI scan. The person performing the MRI will obtain the results of the pregnancy test before administering the scan. If the test is positive, a scan will not be performed. We will inform you that your test is positive, you will be advised to contact your personal physician, and your participation in the research will be terminated.

Incidental findings: Incidental findings are traditionally defined as results that arise that are outside the original purpose for which the test or procedure was conducted. The tests performed in this study are for specific research purposes and are not optimized to find medical abnormalities. The investigators for this project may not be trained to make medical diagnoses. The investigators are

THE RELIEF STUDY
RESEARCHING THE EFFECTIVENESS OF LUMBAR INTERVENTIONS FOR ENHANCING FUNCTION

not responsible for failure to find existing abnormalities. However, on occasion the investigator may notice a finding that seems abnormal. If this occurs, a doctor will be asked to look at the finding (e.g., an image) to see if any medical follow-up is needed. If so, the Medical Director of this project, Dr. Law, will contact you and recommend that you inform your doctor about the findings. If you like (and give written permission) he will also attempt to contact a physician of your choice to discuss the finding with them directly.

II. Spinal Testing - Lab Test Group II

If you are assigned to this group your laboratory testing sessions will occur in the Motor Control Lab at Ohio University located in Grover Center. During these testing sessions you will complete a series of surveys about your low back pain, have the electrical activity of your back muscles examined in response to tapping the back muscles, and participate in a series of tests to see how your body moves (described below).

Core Muscle Reflex Test (CMRT): We will "tap" your back muscles using a small device that is pressed against your back muscles that will periodically push in on your back muscle very quickly. During this we will measure the electrical activity of your back muscles by placing sticky electrodes on the skin overlying your back muscles. To make sure we are able to record these signals we will shave your skin and clean your skin using a mild skin abrasive and skin cleansing alcohol pad. In total we will tap your back muscles about 50 times.

Core Muscle Activation Test (CMAT): First, special light-weight sensors used to track movement will be attached to your arms, legs, and torso. The markers are attached directly to the skin using a series of elastic cuffs. Next, you will be seated in an upright posture in a custom designed chair. A chest harness will be attached to your torso with Velcro straps and your pelvis will be securely fastened using seatbelts that cross your mid-thigh and pelvis. Cables will be attached from the 4 corners of the torso harness to the puller devices located at the 4 the corners of the lab. These pullers make up the core muscle activation tester. You will be asked to sit with your arms held across your chest. The cables that go to each puller will be tensioned to about 5 lbs. of force and then the lines will be tugged in pairs. This will result in a pulling force that will cause your torso to be tugged 2-3 inches in about 1/10 of a second. The pulling force will result in your torso moving either forwards, backwards, twisting left, or twisting right.

Reaching Tasks: You will be asked to reach for two targets placed at different heights in front of you. You will then be asked to point to these same targets located 30 degrees to your left and 30 degree to your right. This sequence is repeated, but we will restrict motion to that of your arms, upper back, and lower back by securing your pelvis to a standing frame using Velcro straps.

III. Cortical Testing - Lab Test Group III

If you are assigned to this group your laboratory testing sessions will occur in the Ohio Musculoskeletal and Neurological Institute at Ohio University located in Irvine Hall. During these testing sessions you will complete a series of surveys about your low back pain, have the electrical activity of your back muscles

examined in response to tapping the back muscles, and participate in a series of tests to see how your brain controls your back muscles (described below).

Core Muscle Reflex Test (CMRT): We will "tap" your back muscles using a small device that is pressed against your back muscles that will periodically push in on your back muscle very quickly. During this we will measure the electrical activity of your back muscles by placing sticky electrodes on the skin overlying your back muscles. To make sure we are able to record these signals we will shave your skin and clean your skin using a mild skin abrasive and skin cleansing alcohol pad. In total we will tap your back muscles about 50 times.

Transcranial Magnetic Stimulation (brain stimulation): A magnetic coil will be placed over the top of your head and the part of your brain that controls the back muscles will be periodically stimulated. This will cause a brief (milliseconds long) contraction of your back muscles as well as some other muscles in your face, torso and limbs. The intensity of these stimulations will vary, but overall each stimulation pulse will be very short (milliseconds long). The brain stimulation will feel like someone is tapping or slapping you on the head.

Risks and Discomforts

Risks or discomforts that you might experience from the interventions are as follows:

- Manipulation Intervention: This is one of the most common interventions for LBP, and the risk of serious injury from lumbar spinal manipulation is less than 1 in 1 million. However, an individual may occasionally have some increased soreness following the manipulation procedure, but this typically resolves in a day.

- Mobilization Intervention: This is one of the most common interventions for LBP, and the risk of serious injury from lumbar spinal mobilization is less than 1 in 1 million. However, an individual may occasionally have some increased soreness following the manipulation procedure, but this typically resolves in a day.

- Cold Laser Intervention: While Cold Laser has no identified side effects, it should not be used over any suspicious cancerous lesions, over the thyroid, on pregnant patients, and there should not be direct irradiation of the eyes, as the laser can cause permanent damage to the eyes.

Risks or discomforts that you might experience from the lab testing procedures are as follows:

- Magnetic Resonance Imaging: Because MRI does not involve the use of radiation, it is generally considered safe for the majority of people. Certain people, however, may be unable to undergo the procedure. These include:
  - Those who have implanted medical devices, including heart pacemakers and inner ear implants
  - Those with metal close to or in an important organ, for example a piece of metal in the eye

possibly from an old injury, or metal clips in the brain following treatment for a brain aneurysm
  - Women who are pregnant

Most MRI exams are painless. However, you may find it uncomfortable to remain still during MR imaging. You may also feel a sense of being closed-in (claustrophobia). Claustrophobia symptoms can include sweating, accelerated heartbeat, nausea, fainting, light-headedness, shaking, and hyperventilation. During the MRI exam, should you feel any of these symptoms, you should make the MRI staff aware. You will be offered earplugs to reduce the noise of the MRI scanner, which produces loud thumping and humming noises during imaging.

- Core Muscle Reflex Test (CMRT): The cleaning and preparation of the skin for the electrodes used to measure muscle activity may cause skin irritation. This irritation generally subsides after a few days.

- Core Muscle Activation Test (CMAT): The cleaning and preparation of the skin for the electrodes used to measure muscle activity may cause a skin irritation. This irritation generally subsides after a few days. The torso responses to the sudden pulling could cause irritation of your current low back pain symptoms. However, we have chosen small pulling distances that should not result in painful spine motion. We also minimize risk by allowing you to stop at any time if you find the procedure too uncomfortable. Any irritation would be expected to subside in 24-48 hours.

- Reaching Tasks: The cleaning and preparation of the skin for the electrodes used to measure muscle activity may cause a skin irritation. This irritation generally subsides after a few days. Bending of the torso could possibly aggravate your current low back symptoms, however these reaching tasks have been chosen because that mimic everyday activities and you should be well within your available range of motion of individuals with back pain. You are free to stop at any time if you find the procedure too uncomfortable.

- Transcranial Magnetic Stimulation (brain stimulation): The cleaning and preparation of the skin for the electrodes used to measure muscle activity may cause a skin irritation. This irritation generally subsides after a few days. The magnetic coil placed over your brain that will induce an electric current and make your muscles contract should not be painful per se, but is frequently described as “weird” or “uncomfortable”. When we do this you will hear a sharp sound that could cause your ears to ring. To minimize this we will give you ear plugs to reduce this noise. More common complaints during or following this procedure are neck pain or headache. It is unknown if these complaints are due to having to sit still or due to the muscles contracting and creating a tension-type headache. These symptoms will probably appear in around 1 out of 4 subjects tested; however, they typically dissipate within a day, and respond well to acetaminophen (Tylenol). You should not participate in this lab testing procedure if you have a history of migraines as the use of the magnetic stimulation technique may induce a migraine. The magnetic coil elicits a magnetic field, so you should not participate in this lab testing procedure if you have any metallic or electrical objects in or on your body (i.e. cardiac pacemakers, metal plates). There is ***no*** radiation exposure associated with this. There is a theoretical chance that the magnetic stimulation could cause a seizure, although this has never been known to occur using the device in the

THE RELIEF STUDY
RESEARCHING THE EFFECTIVENESS OF LUMBAR INTERVENTIONS FOR ENHANCING FUNCTION

manner that we will in healthy people. However, if you have a predisposition to seizures or are taking certain anti-depressant medications you may be susceptible to this. Therefore, if you have a family history of seizures or epilepsy, if you take anti-depressant medications or have experienced a traumatic head injury you should not participate in this study. The magnetic coil makes a 'popping' noise when it is used. You will be asked to insert foam earplugs during this portion of testing to protect your hearing.

NOTE: That you have the right to stop your participation in this investigation at any time.

What We Will Do In the Event of a Medical Emergency: If you experience a medical emergency during a testing or intervention session that you or we feel requires immediate medical attention (e.g., a seizure) we will alert the Ohio University Campus Police and Safety Personnel.

What to Do and Who to Contact if You Have an Adverse Event: If your low back pain substantially worsens during the study or if symptoms exist following completion of the study please contact James Thomas, P.T., Ph.D. at 740-593-4178 or Brian Clark, Ph.D. at 740-593-2354. In the event of a medical emergency please call 911.

Benefits

This study is important to science and society because low back pain affects 8 in 10 Americans and determining the comparative effectiveness of medical treatments could greatly improve clinical practice. You may benefit personally by participating in this study in that your low back pain could be reduced through the application of these interventions.

Confidentiality and Records

Your study information will be kept confidential by the investigators involved in this study. Specifically, only the investigators in the study will have access to your data. Published or presented data will not identify you in any way. You will not be audiotaped or videotaped. All medical history files will be kept in a locked file cabinet located in our office facilities. Once this research has been completed all files will be kept for an additional five years and then destroyed. Additionally, while every effort will be made to keep your study-related information confidential, there may be circumstances where this information must be shared with: 1) Federal agencies, for example the Office of Human Research Protections, whose responsibility is to protect human subjects in research or the National Institutes of Health's National Center for Complementary and Integrative Health or its designees; and 2) Representatives of Ohio University (OU), including the Institutional Review Board, a committee that oversees the research at OU and 3) The RELIEF Study Independent Monitoring Committee, a committee that monitors the data and safety of the research.

Compensation

You will be paid a total of $400 for your participation in this study. Payment is distributed across 8 appointments:

THE RELIEF STUDY
RESEARCHING THE EFFECTIVENESS OF LUMBAR INTERVENTIONS FOR ENHANCING FUNCTION

- Visit 2 - $125
- Visit 3, 4, 5, 6, and 7 - $15 each visit
- Visit 8 - $100
- Visit 9 - $100

This compensation is to offset the time and inconvenience that study participation imposes. We will have to provide your social security number to OU's Finance department because you will earn more than $100 in a calendar year by participating in this study.

Contact Information

If you have any questions regarding this study, please contact James Thomas, P.T., Ph.D. at 740-593-4178, Brian Clark, Ph.D. at 740-593-2354 or Rebecca Batey, Study Coordinator, at 740-566-7246. In the event of a medical emergency please call 911. Additionally, if symptoms exist following completion of the study please contact Drs. Thomas or Clark.

If you have any questions regarding your rights as a research participant, please contact Dr. Chris Hayhow, Director of Research Compliance, at Ohio University, at (740) 593-0664, or email (hayhow@ohio.edu).

By signing below, you are agreeing that:

- you have read this consent form (or it has been read to you) and have been given the opportunity to ask questions and have them answered.
- you have been informed of potential risks and they have been explained to your satisfaction.
- you understand that illness or physical injuries not yet identified could occur as part of this research study.
- you understand Ohio University has no funds set aside for any injuries you might receive as a result of participating in this study.
- you are 18 years of age or older.
- your participation in this research is completely voluntary.
- you may leave the study at any time. If you decide to stop participating in the study, there will be no penalty to you and you will not lose any benefits to which you are otherwise entitled.

Participant Signature____ Date________________

Printed Name____

Study Staff Signature____ Date________________

Printed Name____

THE RELIEF STUDY
RESEARCHING THE EFFECTIVENESS OF LUMBAR INTERVENTIONS FOR ENHANCING FUNCTION
Protocol NIH R01AT006978-01

OHIO UNIVERSITY

DEBRIEFING – Study Treatments Completion

Thank you for participating in The RELIEF Study. Eight out of ten adults will experience an episode of low back pain at some point in their lives. Back pain has a staggeringly negative impact on our society in terms of medical expenses, disability, and individual suffering. It is the second most common reason for a visit to a physician, with costs that exceed $90 billion/year in the U.S. alone. While low back pain is a significant health problem, there is a tremendous lack of mechanistic studies on one of the most popular treatments for this disorder (i.e., manipulation treatment). Spinal manipulative treatments can be broadly classified as non-thrust and thrust-based techniques. Non-thrust-based techniques (e.g., muscle energy) use a low-velocity and low-force approach that generally does not produce audible joint sounds, whereas thrust-based techniques (e.g., translatory thrust) use a high-velocity and low-amplitude approach often accompanied by an audible sound from one or more joints. The overall goal of The RELIEF Study was to examine the mechanisms of non-thrust and thrust-based spinal manipulative techniques used to treat individuals with sub-acute low back pain compared to a placebo group (Laser). We also were interested in a rigorous comparison of the three treatments on pain and disability so we randomly assigned you to the following treatment:

☐ Manipulation ☐ Mobilization ☐ Laser (Placebo)

In the present study we tested the theory that spinal manipulation works at the muscular, spinal, and cortical levels to reduce muscle spasm and thereby breaking the pain-spasm-pain cycle. It is our hope that the present study will lead to a greater understanding of the contributors to low back pain, and will ultimately help us to provide better treatments.

If you have any questions about this study, please do not hesitate to ask the lead scientists James Thomas, P.T., Ph.D. at 740-593-4178 or Brian Clark, Ph.D. at 740-593-2354. Again, thank you for participating in The RELIEF Study.

Regards,

James Thomas, P.T., Ph.D. Brian Clark, Ph.D.

SOP: Protocol Deviations

OBJECTIVE

To describe the policies and procedures for defining, identifying, attributing, dating, signing, and reporting Protocol Deviations.

GENERAL DESCRIPTION

a. Protocol Deviation is defined
b. Protocol Deviation is identified
c. Protocol Deviation is assigned attributions
d. Protocol Deviation is dated
e. Protocol Deviation is signed
f. Protocol Deviation is reported

DEFINITIONS

Protocol Deviation (PD): A variance in a research study between the protocol that has been reviewed and approved by the IRB and the actual performance within the research study, with no substantive effect on the risks to research participants, no substantive effect on the value of the data collected (i.e., the variance does not confound the scientific analysis of the results), and did not result from willful or knowing misconduct on the part of the investigators. (IRB Policy/Procedures Guidelines for the Compliance Office, Revision date 1 July 2016, Section 23.0, Pages 44-45)

Minor Protocol Deviation: a change, divergence, or departure from the study design or procedures that does not affect the scientific soundness of the research plan or the rights, safety, or welfare of human subjects. (Ref: Email from Chris Hayhow, Director of Compliance, dated 17 May 2016 at 9:49 AM)

RESPONSIBILITY

Execution of SOP: Principal Investigator (PI), Co-Investigators, Study Coordinator, Research Assistants, Study Personnel, Independent Monitoring Committee, Sponsor (National Institutes of Health) Program Officer, IRB Chair, IRB, Office of Research Compliance (ORC), ORC Staff

PROCEDURES

a. Protocol Deviation is defined according to IRB Policies & Procedures, the Detailed Study Protocol, Independent Monitoring Committee Data & Safety Monitoring Plan, CFR, ICH, and GCP standards
    1) Definition: A variance in a research study between the protocol that has been reviewed and approved by the IRB and the actual performance within the research study, with no substantive effect on the risks to research participants, no substantive effect on the value of the data collected (i.e., the variance does not confound the scientific analysis of the results), and did not result from willful or knowing misconduct on the part of the investigators. (IRB Policy/Procedures Guidelines for the Compliance Office, Revision date 1 July 2016, Section 23.0, Pages 44-45)
        i. Exemption: A minor protocol deviation is defined as a change, divergence, or departure from the study design or procedures that does not affect the scientific soundness of the research plan or the rights, safety, or welfare of human subjects. Examples of minor or administrative deviations include missed visits, assessments/visits/procedures occurring outside of protocol "windows" or timelines due to scheduling issues or natural events (e.g., weather, University emergency closure), and minor documentation errors (e.g., incorrect date format). (Ref: Email from Chris Hayhow, Director of Compliance, dated 17 May 2016 at 9:49 AM)

b. Protocol Deviation is identified by study staff and documented in the Case Report Form in the Protocol Deviation Form noting description, category & code, date and time of occurrence.

c. Protocol Deviation is assigned attributions by the Study Coordinator under the direct guidance of the Principal Investigator using the following Deviation Category & Code:

CATEGORY A: SAFETY
1. Not reporting an SAE within 24 hours
2. Laboratory tests not done
3. AE/SAE is not reported to IRB
4. Other

CATEGORY B: INFORMED CONSENT
6. Failure to obtain informed consent
7. Consent Form used was not current IRB approved version
8. Consent form does not include updates or information required by IRB
9. Consent form missing
10. Consent form not signed and dated by participant
11. Consent form does not contain all required signatures
12. Other

CATEOGRY C: ELIGIBILITY
1. Participant did not meet eligibility criterion
2. Randomization of an ineligible participant
3. Participant randomized prior to completing baseline assessment, etc.

4. Randomization and/or treatment of participant prior to IRB approval of protocol
5. Other

CATEOGRY D: PROTOCOL IMPLEMENTATION
1. Failure to keep IRB approval up to date
2. Participant receives wrong treatment
3. Participant seen outside visit window intervention & clinical outcomes
4. Participant seen outside visit window physiological outcomes
5. Use of unallowable concomitant treatments
6. Prescribed dosing outside protocol guidelines
7. Missed assessment
8. Missed visit

d. Protocol Deviation is dated
    1) The date of the protocol deviation is assigned for the date the protocol deviation occurred
e. Protocol Deviation is signed
    1) Whenever possible a Principal Investigator signs off on the Protocol Deviation the day the study staff becomes aware of the deviation or at the next weekly investigator meeting.
f. Protocol Deviation is reported
    1) Whenever a researcher anticipates the need to deviate from the procedures previously approved by the IRB, the researcher must obtain the approval of the IRB in advance. (Ref. IRB Policy/Procedures Guidelines for the Compliance Office, Revision date 1 July 2016, Section 23.0, Pages 44-45)
    2) When a deviation occurs without prior approval, the IRB should be notified promptly. The PI must create a Deviation Report and submit it via the LEO electronic IRB system within 72 hours of becoming aware of the deviation. (Ref: IRB Policy/Procedures Guidelines for the Compliance Office, Revision date 1 July 2016, Section 23.0, Pages 44-45)
    3) **Exemption:** Protocol deviations that are only minor or administrative are reported at the time of continuing review. A minor protocol deviation is defined as a change, divergence, or departure from the study design or procedures that does not affect the scientific soundness of the research plan or the rights, safety, or welfare of human subjects. Examples of minor or administrative deviations include missed visits, assessments/visits/procedures occurring outside of protocol "windows" or timelines due to scheduling issues or natural events (e.g., weather, University emergency closure), and minor documentation errors (e.g., incorrect date format). (Ref: Email from Chris Hayhow, Director of Compliance, dated 17 May 2016 at 9:49 AM)
        i. When the Periodic Review (continuing review / renewal) is submitted any deviations that meet the definition above of being "minor or administrative" must be described in both the Periodic Review form and reported in a

single deviation form. The deviation form must list all the deviations that have occurred for ease of review and approval by the Office of Research Compliance and IRB. (Ref: Email from Chris Hayhow, Director of Compliance, dated 17 May 2016 at 9:49 AM)

4) Protocol Deviation Reports are reported to IRB, Independent Monitoring Committee, and the Program Officer.
    i. Protocol Deviations are reported to the IRB submitted through the LEO IRB system.
        1. Within 72-hours of becoming aware of a non-exempt or minor or administrative deviation
        2. At the time of Periodic Review for exempt minor or administrative deviations
            a. Minor or administrative deviations must be described in both the Period Review form and reported in a single deviation form, listing all the deviations have that occurred and have not yet been approved by the IRB (Ref. Phone conversation with Robin Stack 14 October 2016)
    ii. Protocol Deviations are reported to the Independent Monitoring Committee via email on a quarterly basis and reviewed twice annually at meetings.
    iii. Protocol Deviations are reported to the Program Director via email on a quarterly basis in conjunction with the Independent Monitoring Committee reports
    iv. Protocol Deviations are reported with the following attributions: PID, PD Description, Deviation Category & Code, PD Date, and Date Signed by PI

REFERENCES

Email regarding “exemption process” with following header -
From: "Hayhow, Christopher" <hayhow@ohio.edu>
Date: Tuesday, May 17, 2016 at 9:49 AM
To: Brian Clark <clarkb2@ohio.edu>
Cc: France_Christopher <france@ohio.edu>, "Thomas, James" <thomasj5@ohio.edu>
Subject: RE: Protocol Deviation Reporting

IRB Policy/Procedures Guidelines for the Compliance Office, Section 23.0
Detailed Study Protocol, Section 10.3.4
Independent Monitoring Committee Charter
Data and Safety Monitoring Plan
Code of Federal Regulations, Part 312.23(a)(6)(ii) [21 CFR 312.23(a)(6)(ii)]
ICH E6 Good Clinical Practice Consolidated Guidance

ICH E3 Guidance, Section 10.2, Protocol Deviations

SUBMITTER
James Thomas, Principal Investigator
Brian Clark, Co-Investigator
Rebecca Batey, Study Coordinator, Co-Investigator, Corresponding Investigator

Protocol Deviation Category & Code

CATEGORY A: SAFETY
1. Not reporting an SAE within 24 hours
2. Laboratory tests not done
3. AE/SAE is not reported to IRB
4. Other

CATEGORY B: INFORMED CONSENT
6. Failure to obtain informed consent
7. Consent Form used was not current IRB approved version
8. Consent form does not include updates or information required by IRB
9. Consent form missing
10. Consent form not signed and dated by participant
11. Consent form does not contain all required signatures
12. Other

CATEOGRY C: ELIGIBILITY
1. Participant did not meet eligibility criterion
2. Randomization of an ineligible participant
3. Participant randomized prior to completing baseline assessment, etc.
4. Randomization and/or treatment of participant prior to IRB approval of protocol
5. Other

CATEOGRY D: PROTOCOL IMPLEMENTATION
1. Failure to keep IRB approval up to date
2. Participant receives wrong treatment
3. Participant seen outside visit window intervention & clinical outcomes
4. Participant seen outside visit window physiological outcomes
5. Use of unallowable concomitant treatments
6. Prescribed dosing outside protocol guidelines
7. Missed assessment
8. Missed visit

Protocol Deviation Report

PID	PD Description	Deviation Category & Code	PD Date	Date Signed by PI

THE RELIEF STUDY

RESEARCHING THE EFFECTIVENESS OF LUMBAR INTERVENTIONS FOR ENHANCING FUNCTION

Prescreen

THE RELIEF STUDY
RESEARCHING THE EFFECTIVENESS OF LUMBAR INTERVENTIONS FOR ENHANCING FUNCTION
Protocol NIH R01AT006978-01

Place participant ID label here

Participant ID # ___________________

Visit Date _____/_____/______

PRESCREEN ASSESSMENT QUESTIONNAIRE

To be completed in online survey by candidate or by investigator via phone interview
Online survey location – https://ohiochsp.qualtrics.com/jfe/form/SV_9uf1sdySU4DxY9v

CONSENT: You have expressed interest in participating in The RELIEF Study, which is a randomized clinical trial to examine the effects of three treatment interventions for low back pain. To determine if you are eligible for this study we need to ask you some prescreening questions about your medical history, and some of these will be of a personal nature. You do not have to answer any question you do not want to answer and you may choose to stop the survey at any time. Based on your answers to these questions we will determine if we can schedule you for an initial screening assessment, which will include a more complete medical history and physical exam to ensure that you are eligible to participate in this study. However, if based on your answer to these questions we determine that you are not eligible for the study, we will only record data that cannot be linked to you personally, but we will keep record of your answers so that we can obtain a better understanding about how low back pain presents in Southeast Ohio. These questions should take less than 15-minutes. Do you consent to answer these questions?

❍ Yes
❍ No

(NO)
You have answered "No" meaning you decline consent to answer the prescreening questions. You will need to consent to answer the prescreening questions if you wish to complete this screening questionnaire. Are you sure you do NOT wish to consent to answer the prescreening questions?

❍ Yes, I am sure. I do NOT want to consent to answer these questions.
❍ No, it was a mistake. Take me back.

We are sorry you do not consent to answer the prescreening survey questions. Would you prefer to discuss the The RELIEF Study with one of our investigators?

❍ Yes
❍ No

We would be happy to discuss The RELIEF Study with you further. Please indicate below how you would like to be contacted.

❍ Phone (Enter below) _____________________
❍ Email (Enter below) _____________________

Investigator Signature: **Date:**
DAY-MONTH-YEAR

Place participant ID label here

Participant ID # ____________________

Visit Date _____/_____/______

We are sorry you do not wish to answer the prescreening questions for The RELIEF Study. Please recommend our study to others you feel would be interested. Researchers at Ohio University are exploring many different areas of low back pain. May we contact you in the future if we feel other research projects may be of interest to you?

❍ Yes (please enter your email address below) ______________________

❍ No

Are you over 18 years of age?

❍ Yes

❍ No

(NO)

You have indicated you are NOT over 18 years of age. If you are NOT over 18 years of age you may not legally consent to answer the prescreen questions or participate in our study. We welcome you to inquire about The RELIEF Study again when you are 18 years of age or older. Thank you.

We need your date of birth and initials to create your prescreen survey ID #.

Example

*Date of birth: November 18, 1988

*Initials: AMS

Email: alexmsmith@myemail.com

Phone: 740-590-0000

*Required field

*Date of birth:

*Initials:

Email:

Phone:

Investigator Signature: **Date:**

DAY-MONTH-YEAR

THE RELIEF STUDY
RESEARCHING THE EFFECTIVENESS OF LUMBAR INTERVENTIONS FOR ENHANCING FUNCTION
Protocol NIH R01AT006978-01

Place participant ID label here

Participant ID # ____________________

Visit Date _____/_____/______

What is your height and weight?

Example	Height:	5-feet 4-inches
	Weight:	182 pounds
Example	Height:	6 foot 2
	Weight:	123 lbs
Example	Height:	172.5 cm
	Weight:	63.50 kg

Height:
Weight:

Have you had low back pain constantly on most days for the last 3 months?

❍ Yes
❍ No

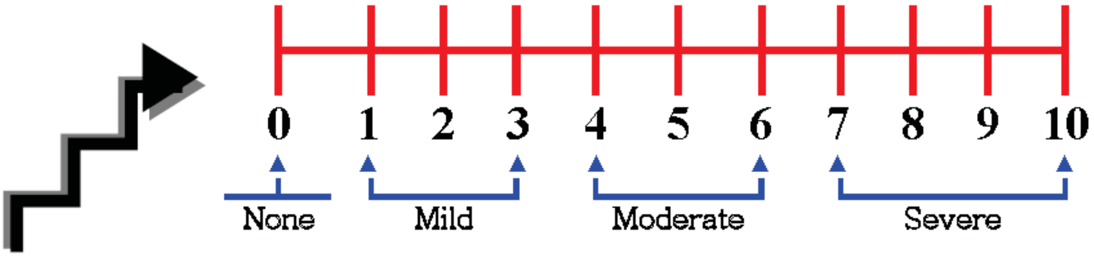

Instructions:
Sometimes it is helpful to think about the Numeric Rating Scale in the following manner:

0 = No Pain
1-3 = Mild Pain (nagging, annoying, interfering little with your activities of daily living)
4–6 = Moderate Pain (interferes significantly with your activities of daily living)
7-10 = Severe Pain (disabling; unable to perform your activities of daily living)

Please indicate:

What number would describe your pain ***over the last 7 days***?

Investigator Signature: **Date:**
DAY-MONTH-YEAR

THE RELIEF STUDY
RESEARCHING THE EFFECTIVENESS OF LUMBAR INTERVENTIONS FOR ENHANCING FUNCTION
Protocol NIH R01AT006978-01

Place participant ID label here

Participant ID # ___________________

Visit Date _____/_____/______

Directions: When your back hurts, you may find it difficult to do some of the things you normally do. Listed below are some sentences that others have used to describe themselves when they have back pain. When you read them, you may find that some stand out because they describe you *today*. As you read the list, think of yourself *today*. When you read a sentence that describes you today, circle YES. If the sentence does not describe you today, circle NO. Remember, only answer YES if you are sure the sentence describes you *today*.

1	I stay at home most of the time because of my back	YES	NO
2	I change positions frequently to try to get my back comfortable	YES	NO
3	I walk more slowly than usual because of my back	YES	NO
4	Because of my back, I am not doing any of the jobs I usually do around the house	YES	NO
5	Because of my back, I use a handrail to get upstairs	YES	NO
6	Because of my back, I lie down to rest more often	YES	NO
7	Because of my back, I have to hold on to something to get out of an easy chair	YES	NO
8	Because of my back, I try to get other people to do things for me	YES	NO
9	I get dressed more slowly than usual because of my back	YES	NO
10	I can only stand up for short periods of time because of my back	YES	NO
11	Because of my back, I try not to bend or kneel down	YES	NO
12	I find it difficult to get out of a chair because of my back	YES	NO
13	My back is painful almost all of the time	YES	NO
14	I find it difficult to turn over in bed because of my back	YES	NO
15	My appetite is not very good because of my back pain	YES	NO
16	I have trouble putting on my socks (or stockings) because of the pain in my back	YES	NO
17	I only walk short distances because of my back pain	YES	NO
18	I sleep less well because of my back	YES	NO
19	Because of my back pain, I get dressed with help from someone else	YES	NO
20	I sit down for most of the day because of my back	YES	NO
21	I avoid heavy jobs around the house because of my back	YES	NO
22	Because of my back pain, I am more irritable and bad tempered with people than usual	YES	NO
23	Because of my back, I go upstairs more slowly than usual	YES	NO
24	I stay in bed most of the time because of my back	YES	NO

Investigator Signature: **Date:**

DAY-MONTH-YEAR

Place participant ID label here

Participant ID # ________________

Visit Date _____/_____/______

Has your back pain caused you to seek care or consultation from a healthcare provider?

❍ Yes
❍ No

In the past month have you received any medical treatments for your back pain? (Check all that apply.)

❍ No
❍ Yes (If yes, date of most recent treatment:____________________ and select treatment below.)
  - ❑ Manual or Manipulative Therapy (e.g., Chiropractic care, osteopathic manipulation, massage therapy, etc.)
  - ❑ Physical therapy
  - ❑ Physician and/or Surgeon care (e.g., care provided by a orthopedic surgeon, family or sports medicine doctor, etc.)
  - ❑ Other (please specify below) ____________________

Have you used narcotic pain medications or muscle relaxants in the past 30 days?

Examples of muscle relaxants:	Examples of narcotic pain medications:
Carisoprodol	Codeine
Soma	Hydrocodone
Flexeril	Oxycodone
Cyclobenzaprine	Lorcet
Diazepam	Lortab
Valium	Norco
Metaxalone	Vicodin
Robaxin	Dilaudid
Skelaxin	Oxycontin
Methocarbamol	Percocet
	Demerol

❍ Yes
❍ No

Investigator Signature: **Date:**

DAY-MONTH-YEAR

Place participant ID label here

Participant ID # ___________________

Visit Date _____/_____/______

Are you currently taking any benzodiazepine medications?

Examples of benzodiazepines:
Alprazolam
Diazepam
Xanax
Klonopin
Valium
Ativan
Halcion
Lorazepam
Diastat
Versed

❍ Yes
❍ No

Are you currently receiving disability services for your low back pain?
❍ Yes
❍ No

Are you involved in pending litigation related to an episode of low back pain?
❍ Yes
❍ No

Investigator Signature: **Date:**

DAY-MONTH-YEAR

Place participant ID label here

Participant ID # ___________________

Visit Date _____/_____/______

Have you had any of the following medical conditions?

- ❑ Congestive heart failure
- ❑ Heart attack
- ❑ Multiple Sclerosis
- ❑ Stroke
- ❑ Osteonecrosis
- ❑ Severe osteoarthritis
- ❑ Alzheimer's Disease
- ❑ Amyotrophic Lateral Sclerosis
- ❑ Parkinson's Disease
- ❑ Rheumatoid arthritis
- ❑ Avascular necrosis
- ❑ Broken spine
- ❑ Spine surgery
- ❑ Hip arthroplasty
- ❑ NONE of these

Do you have a personal or family history of epilepsy or seizures?

- ❍ Yes
- ❍ No

In the past 6-months have you had a migraine headache?

- ❍ Yes

    If yes: Did you have any of the following with your headache?

    You felt nauseated or sick to your stomach when you had a headache?

    Light bothered you (a lot more than when you don't have headaches)?

    Your headaches limited your ability to work, study, or do what you needed to do for at least 1 day?

- ❍ No

Investigator Signature: **Date:**

DAY-MONTH-YEAR

Place participant ID label here

Participant ID # ___________________

Visit Date _____/_____/______

Are you currently...?

- ❑ Blind
- ❑ Pregnant (or anticipate becoming pregnant in the next 3-months)
- ❑ Lactating
- ❑ Diagnosed with active cancer
- ❑ NONE of these

How did you hear about The RELIEF Study?

- ❑ Health Care Provider (please indicate clinic below - UMA, OUTA, Holzer, etc) _______________
- ❑ Email
- ❑ Flyer
- ❑ Billboard / Poster
- ❑ Other; please specify below ___________________
- ❑ Radio
- ❑ Television
- ❑ Friend

What is the race with which you most closely identify?

- ❍ American Indian or Alaska Native
- ❍ Asian
- ❍ Black or African American
- ❍ Native Hawaiian or Other Pacific Islander
- ❍ White
- ❍ More than one race
- ❍ Unknown or not reported

What is the ethnicity with which you most closely identify?

- ❍ Hispanic or Latino
- ❍ Not Hispanic or Latino
- ❍ Unknown or not reported

Thank you for your time in answering these questions. We will review your answers, determine your eligibility to participate in The RELIEF Study, and then contact you soon to discuss the

Investigator Signature: **Date:**

DAY-MONTH-YEAR

Place participant ID label here

Participant ID # ___________________

Visit Date _____/_____/______

study further. If you would like to contact us please email us at RELIEF@ohio.edu or call us at 740-566-PAIN (7246).

Investigator Signature: **Date:**

DAY-MONTH-YEAR

THE RELIEF STUDY

Researching the Effectiveness of Lumbar Interventions for Enhancing Function

CASE REPORT FORMS

Final Version 5.0

PRINCIPAL INVESTIGATORS:

James S. Thomas, P.T., Ph.D.
Professor, School of Rehabilitation and Communication Sciences and the Ohio Musculoskeletal and Neurological Institute, Ohio University
740-593-4178 (W); 740-591-1563 (C); thomasj5@ohio.edu

Brian C. Clark, Ph.D.
Professor, Department of Biomedical Sciences and the Ohio Musculoskeletal and Neurological Institute, Ohio University
740-593-2354 (W); 315-751-8732 (C); clarkb2@ohio.edu

SUPPORTED BY:
The National Center of Complementary and Alternative Medicine
NIH R01AT006978-01

CONFIDENTIAL
The information in The RELIEF Study Case Report Forms document is intended for official use by The RELIEF Study team members and The RELIEF Study funding agencies and monitoring boards. Information in this document is confidential and should not be distributed to unauthorized individuals.

OHIO UNIVERSITY

OHIO UNIVERSITY | OHIO MUSCULOSKELETAL AND NEUROLOGICAL INSTITUTE
Heritage College of Osteopathic Medicine

Revision History

Version Number: DRAFT 1.5
Version Date: 9 January 2013
Summary of Revisions Made:

- Overall changes to align font and formatting to University standards and condense spacing, add consistent branded headers and footers to all documents, add indication of who is to fill out the forms, and minor grammar corrections
- Prescreen Assessment – added more exclusion and inclusion criteria questions; added notice of payment; added scripted dialogues; added questions addressing experiment exclusion criteria; added Participant Information sheet; added General Research Interest sheet
- Screening Visit Checklist – Medical History expanded to two forms and experiment exclusion questions added; Physical Exam expanded to two forms; Standard magnetic field contraindications added to Medical History; Screening Checklist expanded to double-check inclusion, exclusion, and experiment exclusion criteria
- Randomization and Enrollment form expanded to include experiment exclusion and availability, and assignment to experiment and treatment arm
- Treatment Administration Form combined with Treatment Visit Checklist to create a more streamlined document for clinicians.
- Visits 2-7 Checklists expanded to include a checklist for physiological outcomes (experiment 1, 2, or 3)
- Adverse Event forms revised to include NCI Common Terminology Criteria as requested by the IMC.

Version Number: DRAFT 1.7
Version Date: 4 March 2013
Summary of Revisions Made:

- Administrative changes: Minor changes involving grammar, wordsmithing, punctuation, and other editorial changes have been made throughout the document.
- Major revisions to the Prescreen Assessment Questionnaire to be accommodated by both phone screen and internet survey; added several inclusion/exclusion criteria questions; condensed many previous questions
- Demographics form moved to Prescreen Assessment Questionnaire
- Added a new “Health Status Reassessment” and follow-up “Physical Exam – Health Status Reassessment” to routinely rule out a disqualifying change in health status during the course of study participation.
- Added the “Protocol Deviation Form” and “Category and Code” reference sheet
- Added “Delegation of Authority” logs

Version Number: FINAL 1.0
Version Date: 18 March 2013
Summary of Revisions Made:

- Administrative changes: Minor changes involving grammar, wordsmithing, punctuation, and other editorial changes have been made throughout the document.
- Added PARTICIPANT DEBRIEFING letter checkbox to the STUDY COMPLETION form.
- Added CES-D screening failure checkboxes to Visit 1: SCREENING CHECKLIST

- Changed page orientation from landscape to portrait for all INTERVENTION ADMINISTRATION LOGs
- Added “Medical History” and “Medication History” delegations to the treatment providers in the DELEGATION OF AUTHORITY LOG per IRB request
- Added “Adverse Event Inquiry” to Drs. Walkowski and Law’s DELEGATION OF AUTHORITY LOG per IRB request

Version Number: FINAL 2.0
Version Date: 5 June 2014
Summary of Revisions Made:

- Administrative changes: Minor changes involving grammar, wordsmithing, punctuation, and other editorial changes have been made throughout the document.
- Added short latency spinal reflex protocol to CHECKLIST – PHYSIOLOGICAL OUTCOMES Experiment 3. Cortical Effects
- Added an additional Roland Morris Disability Questionnaire and Numerical Pain Rating Scale assessment to the Prescreen Assessment Questionnaire.
- Added additional language to the Prescreen Assessment Questionnaire per IRB request.
- Changed order of forms in Visit 1
- Revised and expanded “Health Status Reassessment” form to better capture Adverse Events
- Added language to “Change in Medication” to better document Adverse Events
- Added “Health Status Reassessment” to Visit 8 and Visit 9
- Moved lab experiment exclusion questions from V1: Medical History – Part 1 to V1: Medical History – Part 2
- Added “Documenting Informed Consent” form to V1
- Added review of prescreen inclusion/exclusion criteria to V1: Medical History – Part 1 and 2
- Added inclusion/exclusion questions to medical history and medication history forms
- Added “end of participation” notification to Appendix: Study Completion form per site monitor request
- Added “Score:_____” fields to every clinical assessment form
- Replaced Visit 1: MEDICAL HISTORY – Magnetic Field Contraindications with form provided by MRI manufacturer
- Added height and weight measurements & BMI calculation to V1 Physical Exam
- Added birth date and age at time of participation to V1
- Removed obsolete random number generator instructions from Enrollment & Randomization
- Added adverse event documenting instructions to Change in Medication form
- Combined the inclusion/exclusion criteria checklists into a single form
-

Version Number: FINAL 5.0
Version Date: 21 April 2016
Summary of Revisions Made:

- Administrative changes: Minor changes involving grammar, wordsmithing, punctuation, and other editorial changes have been made throughout the document.
- Additional questions added to prescreen questionnaire. Some lab experiment groups are closing due to reaching goal capacity. Certain health history factors will exclude some participants from specific lab groups.

Visit 1

Place participant ID label here

Participant ID # ___________________

Visit Date _____/_____/______

Visit 1: Documenting the Informed Consent Process

To be completed by study coordinator

Consent Forms reviewed: The RELIEF Study Informed Consent Version 1.0 Date 18 March 2013
Language of consent form reviewed: English
Study staff member(s) conducting informed consent discussion: Rebecca Batey

Yes	No	Procedure	Note
		Quiet, private room provided?	
		Did the study staff member review the entire document?	
		Purpose of research: To find out what types of treatments work best to treat low back pain and how these treatments work.	
		Duty of participant: 9 visits over 2-months, take only heat/ice/OTC meds for back pain relief	
		Three intervention groups (manipulation, mobilization, laser) with possibility of placebo and random assignment	
		Three laboratory testing groups (MRI, TMS, MCL) and dependent assignment	
		Risks:<br>Interventions: increased soreness is most common, <1/1-million serious injury<br>Lab Testing: skin irritation is most common, 1-MRI claustrophobia, 2-MCL increased pain, 3-TMS headache & neck pain	
		Benefits: important to science, could improve clinical practice, back pain could be reduced	
		Confidentiality: Only investigators have access to participant data, No audio or video taping, files are locked, PID only, retained for 5 years after research completed and destroyed	
		Were comprehension and autonomy assessed by asking the participant to explain what the research is about in his/her own words?	
		Was time allowed to ask/answer questions?	
		Was a copy of the signed consent form provided to the study subject?	
		Was the consent form signed prior to initiation of study procedures?	

Additional Notes:

Investigator Signature: **Date:**

DAY-MONTH-YEAR

Place participant ID label here

Participant ID # ___________________

Visit Date _____/_____/______

Visit 1: MEDICAL HISTORY – Back Pain

To be completed by study coordinator

Date of Birth:__________/______________/__________ Age at time of participation: _________years
(day) (month) (year)

Have you had low back pain constantly OR on most days for the past 3 months? YES NO

Have you ever consulted a health care provider about your low back pain? YES NO

In the past month have you received any medical treatments for your back pain? YES NO
Treatment:
Date of treatment:
*If date of treatment is <30 days prior to V2, exclude participant from study

When did you first notice your low back pain? ______/________/_________
(day) (month) (year)

Is this the first time you've experienced low back pain? ☐ Yes ☐ No
If no: how many episodes of low back pain have you previously had? _____

Would you describe the onset of your low back pain as:
☐ sudden or ☐ gradual

Do you recall a specific incident that caused your low back pain?
☐ Yes ☐ No
If yes, please describe what caused the injur

Have you ever missed work or school due to your low back pain? ☐ Yes (# days?_____) ☐ No

Are you currently receiving disability services for your back pain? ☐ Yes ☐ No

Are you involved in pending litigation related to an episode of low back pain? ☐ Yes ☐ No

Investigator Signature: **Date:**
DAY-MONTH-YEAR

THE RELIEF STUDY
RESEARCHING THE EFFECTIVENESS OF LUMBAR INTERVENTIONS FOR ENHANCING FUNCTION
Protocol NIH R01AT006978-01

Place participant ID label here

Participant ID # ____________________

Visit Date _____/_____/______

Visit 1: NUMERIC PAIN RATING SCALE

To be completed by study coordinator

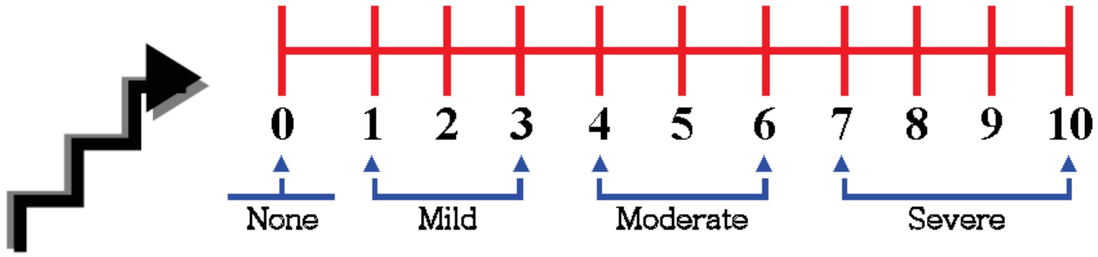

Instructions:
Sometimes it is helpful to think about the Numeric Rating Scale in the following manner:

0 = No Pain
1-3 = Mild Pain (nagging, annoying, interfering little with your activities of daily living)
4–6 = Moderate Pain (interferes significantly with your activities of daily living)
7-10 = Severe Pain (disabling; unable to perform your activities of daily living)

Please indicate:

What number would describe your pain ***over the last 7 days***? ☐

Investigator Signature: **Date:**

DAY-MONTH-YEAR

THE RELIEF STUDY
RESEARCHING THE EFFECTIVENESS OF LUMBAR INTERVENTIONS FOR ENHANCING FUNCTION
Protocol NIH R01AT006978-01

Place participant ID label here

Participant ID # ___________________

Visit Date _____/_____/______

Visit 1: ROLAND-MORRIS DISABILITY QUESTIONNAIRE

To be completed by study coordinator

Directions: When your back hurts, you may find it difficult to do some of the things you normally do. Listed below are some sentences that others have used to describe themselves when they have back pain. When you read them, you may find that some stand out because they describe you *today*. As you read the list, think of yourself *today*. When you read a sentence that describes you today, circle YES. If the sentence does not describe you today, circle NO. Remember, only answer YES if you are sure the sentence describes you *today*.

1	I stay at home most of the time because of my back	YES	NO
2	I change positions frequently to try to get my back comfortable	YES	NO
3	I walk more slowly than usual because of my back	YES	NO
4	Because of my back, I am not doing any of the jobs I usually do around the house	YES	NO
5	Because of my back, I use a handrail to get upstairs	YES	NO
6	Because of my back, I lie down to rest more often	YES	NO
7	Because of my back, I have to hold on to something to get out of an easy chair	YES	NO
8	Because of my back, I try to get other people to do things for me	YES	NO
9	I get dressed more slowly than usual because of my back	YES	NO
10	I can only stand up for short periods of time because of my back	YES	NO
11	Because of my back, I try not to bend or kneel down	YES	NO
12	I find it difficult to get out of a chair because of my back	YES	NO
13	My back is painful almost all of the time	YES	NO
14	I find it difficult to turn over in bed because of my back	YES	NO
15	My appetite is not very good because of my back pain	YES	NO
16	I have trouble putting on my socks (or stockings) because of the pain in my back	YES	NO
17	I only walk short distances because of my back pain	YES	NO
18	I sleep less well because of my back	YES	NO
19	Because of my back pain, I get dressed with help from someone else	YES	NO
20	I sit down for most of the day because of my back	YES	NO
21	I avoid heavy jobs around the house because of my back	YES	NO
22	Because of my back pain, I am more irritable and bad tempered with people than usual	YES	NO
23	Because of my back, I go upstairs more slowly than usual	YES	NO
24	I stay in bed most of the time because of my back	YES	NO

Score:_______

Investigator Signature: **Date:**

DAY-MONTH-YEAR

THE RELIEF STUDY
RESEARCHING THE EFFECTIVENESS OF LUMBAR INTERVENTIONS FOR ENHANCING FUNCTION
Protocol NIH R01AT006978-01

Place participant ID label here

Participant ID # ___________________

Visit Date _____/_____/______

Visit 1: CENTER FOR EPIDEMIOLOGIC STUDIES - DEPRESSION

To be completed by study coordinator

Fill the response circle for each statement which best describes how often you felt this way during the past week according to the given scale. Please fill the response circles completely.		Rarely or None of the Time (less than 1 day)	Some or a Little of the Time (1-2 days)	Occasionally or a Moderate Amount of the Time (3-4 days)	Most or All of the Time (5-7 days)
1.	I was bothered by things that usually don't bother me	O	O	O	O
2.	I did not feel like eating; my appetite was poor	O	O	O	O
3.	I felt that I could not shake off the blues even with help from friends	O	O	O	O
4.	I felt that I was just as good as other people	O	O	O	O
5.	I had trouble keeping my mind on what I was doing	O	O	O	O
6.	I felt depressed	O	O	O	O
7.	I felt that everything I did was an effort	O	O	O	O
8.	I felt hopeful about the future	O	O	O	O
9.	I thought my life had been a failure	O	O	O	O
10.	I felt fearful	O	O	O	O
11.	My sleep was restless	O	O	O	O
12.	I was happy	O	O	O	O
13.	I talked less than usual	O	O	O	O
14.	I felt lonely	O	O	O	O
15.	People were unfriendly	O	O	O	O
16.	I enjoyed life	O	O	O	O
17.	I had crying spells	O	O	O	O
18.	I felt sad	O	O	O	O
19.	I felt that people disliked me	O	O	O	O
20.	I could not get "going"	O	O	O	O

Score:____________

Investigator Signature: **Date:**

DAY-MONTH-YEAR

Place participant ID label here

Participant ID # ___________________

Visit Date _____/_____/______

Visit 1: FEAR AVOIDANCE BELIEFS QUESTIONNAIRE

To be completed by study coordinator

Directions: Here are some of the things that others have said about their pain. For each statement, please circle any number from 0 to 6 to say how much physical activities such as bending, lifting or driving affect or would affect *your* back pain.		Completely disagree			Unsure			Completely agree
1.	My pain was caused by physical activity.	0	1	2	3	4	5	6
2.	Physical activity makes my pain worse.	0	1	2	3	4	5	6
3.	Physical activity might harm my back.	0	1	2	3	4	5	6
4.	I should not do physical activities that (might) make my pain worse.	0	1	2	3	4	5	6
5.	I cannot do physical activities that (might) make my pain worse.	0	1	2	3	4	5	6

The following statements are about how your normal work affects or would affect your back pain.		Completely disagree			Unsure			Completely agree
6.	My pain was caused by my work or by an accident at work.	0	1	2	3	4	5	6
7.	My work aggravated my pain.	0	1	2	3	4	5	6
8.	I have a claim for compensation for my pain.	0	1	2	3	4	5	6
9.	My work is too heavy for me.	0	1	2	3	4	5	6
10.	My work makes or would make my pain worse.	0	1	2	3	4	5	6
11.	My work might harm my back.	0	1	2	3	4	5	6
12.	I should not do my normal work with my present pain.	0	1	2	3	4	5	6
13.	I cannot do my normal work with my present pain.	0	1	2	3	4	5	6
14.	I cannot do my normal work till my pain is treated.	0	1	2	3	4	5	6
15.	I do not think that I will be back to my normal work within 3 months.	0	1	2	3	4	5	6
16.	I do not think that I will ever be able to go back to that work.	0	1	2	3	4	5	6

SCORE Subscale Questions 1-5: Subscale Questions 6-16:

Investigator Signature: **Date:**
DAY-MONTH-YEAR

Place participant ID label here

Participant ID # ___________________

Visit Date _____/_____/______

Visit 1: CONTRAINDICATIONS FOR EXPOSURE TO A MAGNETIC FIELD

To be completed by study coordinator

Do you have any ***non-removable*** metal or foreign objects in your body such as Cardiac Pacemaker, Cardiac Valve Replacement, Brain Aneurysm Clip, Shunt, Aortic Clip, Surgical Clips, Implanted Neurotransmitter, Insulin Pump, Infusion device, or internal hearing aid such as Cochlear implant?

☐ Yes (if yes, indicate on Medical History – Summary, exclude from TMS & MRI lab testing and refer to screening clinician)
☐ No

Do you have any **removable** metal or foreign objects in your body such as piercings, hearing aids, or prosthetic devices?

☐ Yes (refer to screening clinician) ☐ No

Do you have any joint replacements, metal rods, plates, screws, or nails in your body?

☐ Yes (refer to screening clinician and complete below) ☐ No

Surgery:
Date:

Surgery:
Date:

Surgery:
Date:

Do you have any foreign objects in your body such as shrapnel, bullet, an eye injury involving metal or do you work with metal occupationally?

☐ Yes (refer to screening clinician clinician) ☐ No

Investigator Signature: **Date:**

DAY-MONTH-YEAR

THE RELIEF STUDY
RESEARCHING THE EFFECTIVENESS OF LUMBAR INTERVENTIONS FOR ENHANCING FUNCTION
Protocol NIH R01AT006978-01

Place participant ID label here

Participant ID # ___________________

Visit Date _____/_____/______

Visit 1: MEDICAL HISTORY - Summary

To be completed by study coordinator

"Do you have any medical or surgical history, current or resolved, of any of the following?"

MEDICAL HISTORY	Yes / No	Unknown	If Yes, Explain	Current / Resolved
Head, Eye, Ear, Nose, Throat	☐ Yes ☐ No	☐		☐ Current ☐ Resolved
Respiratory	☐ Yes ☐ No	☐		☐ Current ☐ Resolved
Cardiovascular	☐ Yes ☐ No	☐		☐ Current ☐ Resolved
Gastrointestinal	☐ Yes ☐ No	☐		☐ Current ☐ Resolved
Genitourinary	☐ Yes ☐ No	☐		☐ Current ☐ Resolved
Musculoskeletal	☐ Yes ☐ No	☐		☐ Current ☐ Resolved
Neurological	☐ Yes ☐ No	☐		☐ Current ☐ Resolved
Endocrine-Metabolic	☐ Yes ☐ No	☐		☐ Current ☐ Resolved
Blood/Lymphatic	☐ Yes ☐ No	☐		☐ Current ☐ Resolved
Dermatologic	☐ Yes ☐ No	☐		☐ Current ☐ Resolved
Psychiatric	☐ Yes ☐ No	☐		☐ Current ☐ Resolved
Allergy	☐ Yes ☐ No	☐		☐ Current ☐ Resolved
Other, specify: ________	☐ Yes ☐ No	☐		☐ Current ☐ Resolved

Have you ever had spinal surgery? ☐ Yes ☐ No

Do you have any family or personal history of epilepsy or seizures?

☐ Yes or UNKNOWN (assign to experiment group 1 or 2) ☐ No

Do you have any personal history of migraine headaches?

☐ Yes (if yes, when was the most recent migraine?____________________*) ☐ No

*If migraine within 6-months (186 days) of V2 physiologic assessments assign to Experiment 1 or 2

Investigator Signature: **Date:**

DAY-MONTH-YEAR

Place participant ID label here

Participant ID # ____________________

Visit Date _____/_____/______

Visit 1: MEDICATION HISTORY

To be completed by study coordinator

"Have you taken any medications in the past 30 days?"
☐ Yes (If yes, record below.) ☐ No

Medication	Indication	Dose & Frequency	Start Date	Stop Date	Ongoing
1.					☐
2.					☐
3.					☐
4.					☐
5.					☐
6.					
7.					
8.					

Has the participant used narcotic pain medications or muscle relaxants in the past 30 days?
YES (Exclude from study) NO

Is the participant currently taking medications in the benzodiazepine class?
YES (Exclude from Experiment 3 TMS) NO

Investigator Signature: **Date:**
DAY-MONTH-YEAR

Place participant ID label here

Participant ID # ___________________

Visit Date _____/_____/______

Visit 1: PHYSICAL EXAM FORM 1

To be completed by clinician

Category	Normal or Abnormal	If abnormal, describe below
General Appearance	☐ Normal<br>☐ Abnormal<br>☐ Not Examined	
Musculoskeletal	☐ Normal<br>☐ Abnormal<br>☐ Not Examined	
Extremities/ Skin	☐ Normal<br>☐ Abnormal<br>☐ Not Examined	
Neurological (dermatomes, myotomes, reflexes)	☐ Normal<br>☐ Abnormal<br>☐ Not Examined	
Other, specify: _____	☐ Normal<br>☐ Abnormal<br>☐ Not Examined	

Height:______________

Weight: _____________

BMI:________

Investigator Signature: **Date:** ____________________

DAY-MONTH-YEAR

THE RELIEF STUDY
RESEARCHING THE EFFECTIVENESS OF LUMBAR INTERVENTIONS FOR ENHANCING FUNCTION
Protocol NIH R01AT006978-01

Place participant ID label here

Participant ID # __________________

Visit Date _____/_____/______

Visit 1: PHYSICAL EXAM FORM 2

To be completed by clinician

Site(s) of symptoms:

Left Lumbar Spine ☐ Level _____

Right Lumbar Spine ☐ Level _____

Central Lumbar Spine ☐ Level _____

Does the pain radiate to:

Right leg ☐ Below Knee ☐

Left Leg ☐ Below Knee ☐

How does the candidate describe the pain? ____

Does the candidate report that the pain awakens them at night? ☐ Yes ☐ No

Is the pain constant or come in waves/spasm? ☐ Constant ☐ Waves/Spasms

Does the candidate report any recent unexplained change in weight?

☐ Yes (If yes, amount lost _________; time period __________)

☐ No

Does the candidate report any recent infections or fever? ☐ Yes* ☐ No

(*If candidate currently has fever, delay enrollment until fever subsides.)

Clinical Prediction Rules

Does patient have symptoms that radiate below the Knee? ☐ Yes ☐ No

Does patient have at least 1 hypomobile segment? ☐ Yes ☐ No

Does patient have at least 1 hip with >35 degrees of internal rotation range of motion?

☐ Yes ☐ No

Investigator Signature: **Date:**

DAY-MONTH-YEAR

THE RELIEF STUDY
RESEARCHING THE EFFECTIVENESS OF LUMBAR INTERVENTIONS FOR ENHANCING FUNCTION
Protocol NIH R01AT006978-01

Place participant ID label here

Participant ID # ___________________

Visit Date _____/_____/______

Visit 1: TAMPA SCALE for KINESIOPHOBIA

To be completed by study coordinator

Directions: Please read each of the following statements and circle the number that best represents your feelings.	Strongly Disagree	Disagree	Agree	Strongly Agree
I'm afraid that I might injure myself if I exercise.	1	2	3	4
If I were to try to overcome it, my pain would increase.	1	2	3	4
My body is telling me I have something dangerously wrong.	1	2	3	4
My pain would probably be relieved if I were to exercise.	1	2	3	4
People aren't taking my medical condition seriously enough.	1	2	3	4
My accident has put my body at risk for the rest of my life.	1	2	3	4
Pain always means I have injured my body.	1	2	3	4
Just because something aggravates my pain does not mean it is dangerous.	1	2	3	4
I am afraid that I might injure myself accidentally.	1	2	3	4
Simply being careful that I do not make any unnecessary movements is the safest thing I can do to prevent my pain from worsening.	1	2	3	4
I wouldn't have this much pain if there weren't something potentially dangerous going on in my body.	1	2	3	4
Although my condition is painful, I would be better off if I were physically active.	1	2	3	4
Pain lets me know when to stop exercising so that I don't injure myself.	1	2	3	4
It's really not safe for a person with a condition like mine to be physically active.	1	2	3	4
I can't do all the things normal people do because it's too easy for me to get injured.	1	2	3	4
Even though something is causing me a lot of pain, I don't think it's actually dangerous.	1	2	3	4
No one should have to exercise when he/she is in pain.	1	2	3	4

Score: ________

Investigator Signature: **Date:**

DAY-MONTH-YEAR

THE RELIEF STUDY
RESEARCHING THE EFFECTIVENESS OF LUMBAR INTERVENTIONS FOR ENHANCING FUNCTION
Protocol NIH R01AT006978-01

Place participant ID label here

Participant ID # ___________________

Visit Date _____/_____/______

Visit 1: SCREENING CHECKLIST – INCLUSION/EXCLUSION CRITERIA

To be completed by study coordinator

	Yes	No
18-45 years of age		
Answers yes to the following question: “Have you had low back pain constantly or on most days for the last three months?”		
Answer yes to the following question: “Has your back pain caused you to seek care or consultation from a health care provider?”		
Pain intensity rating over the past week ≥ 2 on a 0-10 numerical pain scale		
RMDQ score ≥ 4		
Exhibit 3 of 4 of the following findings reported in the clinical prediction rules for spinal manipulation<br>☐ FABQ work subscale score < 19<br>☐ No symptoms distal to the knee<br>☐ At least 1 hypo-mobile lumbar spinal segment<br>☐ At least 1 hip > 35 degrees internal rotation range of motion		
No personal history of excluded disorders – Alzheimer’s, amyotrophic lateral sclerosis, multiple sclerosis, Parkinson’s, stroke, congestive heart failure, heart attack in the past 24 months, rheumatoid arthritis, spine or pathologic fractures, avascular necrosis, osteonecrosis, severe osteoarthritis, blindness, or active cancer.		
No history of spine surgery or hip arthroplasty		
No use of narcotics or muscle relaxants within 30 days prior to study entry		
Not pregnant, lactating, or anticipate becoming pregnant in the next 3-months		
No BMI > 35 kg/m2		
No clinical depression – CESD score ≥ 24		
No unexplained weight loss > 10lbs over the past month		
No pending litigation related to LBP		
No disability services related to LBP		
No manual therapy treatment of the spine30 days prior to first study intervention		
No drug or alcohol use that would interfere with adherence to study requirements		

Did the participant meet the eligibility requirements for this study?

☐ Yes (If yes, continue to next form)

☐ No (If no, complete “Study Completion Form”)

Investigator Signature: **Date:**

DAY-MONTH-YEAR

Place participant ID label here

Participant ID # ___________________

Visit Date _____/_____/______

Visit 1: SCREENING CHECKLIST - LIMITING CRITERIA

To be completed by study coordinator

The following questions exclude the participant from certain experiments, but do not exclude the participant from enrolling as a study participant:	YES	NO
Have a personal or family history of epilepsy or seizures, migraine headaches within 6-months (186 days) of V2 physiologic testing, or taking medications in the benzodiazepine class (Exclude from Experiment 3 TMS).		
Have contraindications for exposure to a magnetic field (Exclude from Experiment 1 MRI and Experiment 3 TMS).		
Have any other contraindications for participating in any of the 3 study experiments. Explain:		

Does the candidate have any conditions qualifying for exclusion from experiments for this study?

☐ Yes (If yes, complete “Experiment Exclusion Section” of “Randomization and Enrollment Form”)

☐ No

Investigator Signature: **Date:**

DAY-MONTH-YEAR

Place participant ID label here

Participant ID # ___________________

Visit Date _____/_____/______

Visit 1: ENROLLMENT AND RANDOMIZATION

To be completed by study coordinator

Is the participant eligible for the study based on inclusion and exclusion criteria?

☐ Yes (If yes, date enrolled _______________ and randomized if different _____________________)
Day-Month-Year Day-Month-Year
☐ No (If no, leave the rest of the form blank.)

If eligible and not randomized, indicate reason:
☐ Failed to return ☐ Declined participation
☐ Other (specify): ____

Experiment Exclusion Section: The candidate is ***excluded*** from participating in the following experiment(s):
☐ Experiment 1 (Muscular): MRI
☐ Experiment 2 (Spinal): Biomechanics
☐ Experiment 3 (Cortical): TMS

Participant is available* for the following experiments:
☐ 1 Muscular - MRI
☐ 2 Spinal - Biomechanics
☐ 3 Cortical - TMS

*Participant being "available" is based on: 1) meeting inclusion/exclusion criteria for a given experiment; 2) the experimental protocol being open to enrollment; and 3) the study participant and laboratory schedules align.

Participant assigned to Experiment:
☐ 1 Muscular - MRI
☐ 2 Spinal - Biomechanics
☐ 3 Cortical - TMS

Participant randomly assigned to Intervention Arm:
☐ 1 Manipulation
☐ 2 Mobilization
☐ 3 Laser

Parking passes needed?
☐ Yes
☐ No

Investigator Signature: **Date:**
DAY-MONTH-YEAR

Place participant ID label here

Participant ID # ___________________

Visit Date _____/_____/______

VISIT 1: SCREENING CHECKLIST

To be completed by study coordinator

Did the participant attend this visit? ☐ Yes (if yes, continue) ☐ No

Please check all assessments completed at this visit:
Visit Name: Screening

- ☐ Informed Consent
- ☐ Medical History – Part 1
- ☐ NPR
- ☐ RMDQ
- ☐ CES-D
- ☐ FABQ
- ☐ Medical History – Part 2
- ☐ Medication History
- ☐ Physical Exam 1
- ☐ Physical Exam 2
- ☐ TSK
- ☐ Medical History – MRI Contraindications
- ☐ Criteria Checklists (3)
- ☐ Enrollment & Randomization

Is the participant continuing in the study?
☐ Yes (if yes, complete a RANDOMIZATION AND ENROLLMENT form)
☐ No (if no, complete a STUDY COMPLETION form)

Comments:
______

Investigator Signature: **Date:**

DAY-MONTH-YEAR

Visit 2

THE RELIEF STUDY
RESEARCHING THE EFFECTIVENESS OF LUMBAR INTERVENTIONS FOR ENHANCING FUNCTION
Protocol NIH R01AT006978-01

Place participant ID label here

Participant ID # ___________________

Visit Date _____/_____/______

Visit 2: PRIOR AND CONCOMITANT MEDICATIONS

To be completed by study coordinator

Have you taken any medications (including over the counter pain relief medications) or used heat or ice for your back pain in the past 7 days?

☐ No ☐ Yes (If YES, record below.)

Medication/Modality	Frequency and Dose	Indication	Start Date	Stop Date	Ongoing
1.					☐
2.					☐
3.					☐
4.					☐
5.					☐

Investigator Signature: **Date:**

DAY-MONTH-YEAR

THE RELIEF STUDY
RESEARCHING THE EFFECTIVENESS OF LUMBAR INTERVENTIONS FOR ENHANCING FUNCTION
Protocol NIH R01AT006978-01

Place participant ID label here

Participant ID # ___________________

Visit Date _____/_____/______

Visit 2: CHANGE IN MEDICATION USE

To be completed by study coordinator

Have you had any changes in medication use NOT related to your back pain in the past 7 days?
☐ No ☐ Yes (If YES, record below.)

Medication	Indication*	Dose & Frequency	Start Date	Stop Date	Ongoing
1.					☐
2.					☐
3.					☐
4.					☐
5.					☐

*If Indication is related to a negative change in health status, alert study coordinator, complete Health Status Reassessment and enter in Adverse Event Log.

Notes:

Investigator Signature: **Date:**
DAY-MONTH-YEAR

THE RELIEF STUDY
RESEARCHING THE EFFECTIVENESS OF LUMBAR INTERVENTIONS FOR ENHANCING FUNCTION
Protocol NIH R01AT006978-01

Place participant ID label here

Participant ID # ___________________

Visit Date _____/_____/______

Visit 2: HEALTH STATUS REASSESSMENT

To be completed by study staff

Since your last visit, have you had any change in your health?

☐ No ☐ Yes (If YES, record below, alert study coordinator, and complete Adverse Event log.)

**Change & Symptoms**	
**Describe:**<br><br><br><br>**Start Date:**<br>**Stop Date:**<br>**Missed work or school?______days**	**Treatment:**<br><br><br>**Outcome:**

**Change & Symptoms**	
**Describe:**<br><br><br><br>**Start Date:**<br>**Stop Date:**<br>**Missed work or school?______days**	**Treatment:**<br><br><br>**Outcome:**

**Change & Symptoms**	
**Describe:**<br><br><br><br>**Start Date:**<br>**Stop Date:**<br>**Missed work or school?______days**	**Treatment:**<br><br><br>**Outcome:**

Investigator Signature: **Date:**

DAY-MONTH-YEAR

THE RELIEF STUDY
RESEARCHING THE EFFECTIVENESS OF LUMBAR INTERVENTIONS FOR ENHANCING FUNCTION
Protocol NIH R01AT006978-01

Place participant ID label here

Participant ID # ___________________

Visit Date _____/_____/______

Visit 2: CONTRAINDICATIONS FOR INTERVENTION

To be completed by study staff

**Have you had any of the following symptoms?*:**		**If YES, describe here:**
Radiating pain (burning, numbness, or tingling in the legs)	☐ Yes ☐ No	
Muscle weakness in the legs	☐ Yes ☐ No	
Episodes of incontinence of bowel or bladder	☐ Yes ☐ No	

*If YES, describe in right column, refer to clinician for Health Status Reassessment Physical Exam, and complete Adverse Event Log (forms located in Appendix).

Investigator Signature: **Date:**

DAY-MONTH-YEAR

Place participant ID label here

Participant ID # ___________________

Visit Date _____/_____/______

Visit 2: CONTRAINDICATIONS FOR LAB TESTING

To be completed by study staff

Lab Testing Group		Since your last visit have you had any of the following?		If yes, describe and consult PI
☐	1 Muscular MRI	Contraindications for exposure to a magnetic field? (Must complete Magnetic Field Contraindications form)	☐ Yes ☐ No	
☐	2 Spinal MCL	Muscle weakness in the legs?	☐ Yes ☐ No	
		Episodes of incontinence of bowel or bladder?	☐ Yes ☐ No	
		New or previously unreported muscle, bone or joint injury?	☐ Yes ☐ No	Date:__________________
☐	3 Cortical TMS	Contraindications for exposure to a magnetic field?	☐ Yes ☐ No	
		Migraine headache 6-months prior to V2 physiologic assessments?	☐ Yes ☐ No	Administer ID Migraine Screener:<br>Sensitivity to light? ☐Yes ☐No<br>Nausea or vomiting?☐Yes ☐ No<br>Disabling intensity? ☐Yes ☐ No<br>*if at least 2 symptoms, cancel TMS testing<br>Date of last migraine:___________
		Taking medications in the benzodiazepine class?	☐ Yes ☐ No	
		Recent concussion?	☐ Yes ☐ No	

If YES, describe in right column, refer to PIs, and complete Adverse Event Log (forms located in Appendix).

Investigator Signature: **Date:**

DAY-MONTH-YEAR

THE RELIEF STUDY
RESEARCHING THE EFFECTIVENESS OF LUMBAR INTERVENTIONS FOR ENHANCING FUNCTION
Protocol NIH R01AT006978-01

Place participant ID label here

Participant ID # ________________

Visit Date _____/_____/______

Visit 2: CONTRAINDICATIONS FOR EXPOSURE TO A MAGNETIC FIELD

To be completed by study coordinator for MRI & TMS Lab Testing

Do you have any ***non-removable*** metal or foreign objects in your body such as Cardiac Pacemaker, Cardiac Valve Replacement, Brain Aneurysm Clip, Shunt, Aortic Clip, Surgical Clips, Implanted Neurotransmitter, Insulin Pump, Infusion device, or internal hearing aid such as Cochlear implant?

☐ Yes (if yes, indicate on Medical History – Summary, exclude from TMS & MRI lab testing)
☐ No

Do you have any **removable** metal or foreign objects in your body such as piercings, hearing aids, or prosthetic devices?

☐ Yes (alert lab) ☐ No

Do you have any joint replacements, metal rods, plates, screws, or nails in your body?

☐ Yes (alert lab and complete below) ☐ No

Surgery:
Date:

Surgery:
Date:

Surgery:
Date:

Do you have any foreign objects in your body such as shrapnel, bullet, an eye injury involving metal or do you work with metal occupationally?

☐ Yes (alert lab) ☐ No

Investigator Signature: **Date:**

DAY-MONTH-YEAR

THE RELIEF STUDY
RESEARCHING THE EFFECTIVENESS OF LUMBAR INTERVENTIONS FOR ENHANCING FUNCTION
Protocol NIH R01AT006978-01

Place participant ID label here

Participant ID # ___________________

Visit Date _____/_____/______

Visit 2: NUMERIC PAIN RATING SCALE PRE-INTERVENTION

To be completed by assessment team investigator

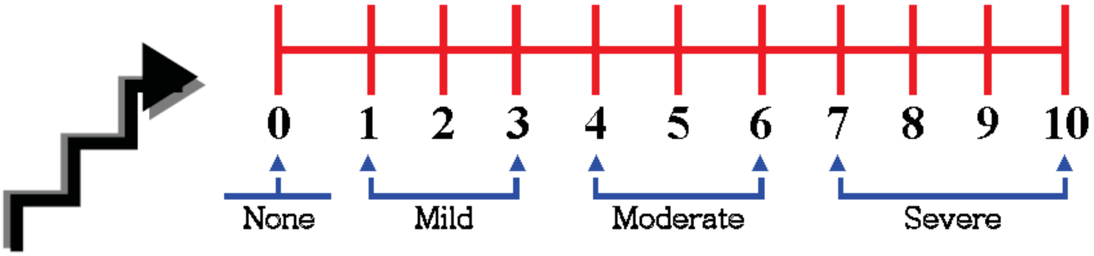

Instructions:
Sometimes it is helpful to think about the Numeric Rating Scale in the following manner:

- 0 = No Pain
- 1-3 = Mild Pain (nagging, annoying, interfering little with your activities of daily living)
- 4–6 = Moderate Pain (interferes significantly with your activities of daily living)
- 7-10 = Severe Pain (disabling; unable to perform your activities of daily living)

Please indicate:

- What number would you give your pain ***right now***? ☐
- What number would describe your pain ***over the last 7 days***? ☐

Since the onset of your low back pain how would you answer the next 3 questions:

- What number on a 0 to 10 scale would you give your pain when it is the worst that it gets? ☐
- What number on a 0 to 10 scale would you give your pain and when it is the best that it gets? ☐
- At what number is the pain at an acceptable level for you? ☐

Investigator Signature: **Date:**
DAY-MONTH-YEAR

Place participant ID label here

Participant ID # ___________________

Visit Date _____/_____/______

Visit 2: ROLAND-MORRIS DISABILITY QUESTIONNAIRE

To be completed by assessment team investigator

Directions: When your back hurts, you may find it difficult to do some of the things you normally do. Listed below are some sentences that others have used to describe themselves when they have back pain. When you read them, you may find that some stand out because they describe you *today*. As you read the list, think of yourself *today*. When you read a sentence that describes you today, circle YES. If the sentence does not describe you today, circle NO. Remember, only answer YES if you are sure the sentence describes you *today*.

1	I stay at home most of the time because of my back	YES	NO
2	I change positions frequently to try to get my back comfortable	YES	NO
3	I walk more slowly than usual because of my back	YES	NO
4	Because of my back, I am not doing any of the jobs I usually do around the house	YES	NO
5	Because of my back, I use a handrail to get upstairs	YES	NO
6	Because of my back, I lie down to rest more often	YES	NO
7	Because of my back, I have to hold on to something to get out of an easy chair	YES	NO
8	Because of my back, I try to get other people to do things for me	YES	NO
9	I get dressed more slowly than usual because of my back	YES	NO
10	I can only stand up for short periods of time because of my back	YES	NO
11	Because of my back, I try not to bend or kneel down	YES	NO
12	I find it difficult to get out of a chair because of my back	YES	NO
13	My back is painful almost all of the time	YES	NO
14	I find it difficult to turn over in bed because of my back	YES	NO
15	My appetite is not very good because of my back pain	YES	NO
16	I have trouble putting on my socks (or stockings) because of the pain in my back	YES	NO
17	I only walk short distances because of my back pain	YES	NO
18	I sleep less well because of my back	YES	NO
19	Because of my back pain, I get dressed with help from someone else	YES	NO
20	I sit down for most of the day because of my back	YES	NO
21	I avoid heavy jobs around the house because of my back	YES	NO
22	Because of my back pain, I am more irritable and bad tempered with people than usual	YES	NO
23	Because of my back, I go upstairs more slowly than usual	YES	NO
24	I stay in bed most of the time because of my back	YES	NO

Score: _________

Investigator Signature: **Date:**

DAY-MONTH-YEAR

THE RELIEF STUDY
RESEARCHING THE EFFECTIVENESS OF LUMBAR INTERVENTIONS FOR ENHANCING FUNCTION
Protocol NIH R01AT006978-01

Place participant ID label here

Participant ID # ___________________

Visit Date _____/_____/______

Visit 2: PROMIS – PAIN INTENSITY

To be completed by assessment team investigator

Instructions: Please respond to each item by marking one box per row.

In the past 7 days…

	Had no pain	Mild	Moderate	Severe	Very Severe
How intense was your pain at its worst?					
How intense was your average pain?					
What is your level of pain right now?					

Score: ________

Investigator Signature: **Date:**
DAY-MONTH-YEAR

THE RELIEF STUDY
RESEARCHING THE EFFECTIVENESS OF LUMBAR INTERVENTIONS FOR ENHANCING FUNCTION
Protocol NIH R01AT006978-01

Place participant ID label here

Participant ID # ________________

Visit Date _____/_____/______

Visit 2: PROMIS – PAIN BEHAVIOR

To be completed by assessment team investigator

Instructions: Please respond to each item by marking one box per row.

In the past 7 days…

	Had no pain	Never	Rarely	Sometimes	Often	Always
When I was in pain I became irritable						
When I was in pain I grimaced						
When I was in pain I moved extremely slowly						
When I was in pain I moved stiffly						
When I was in pain I called out for someone to help me						
When I was in pain I isolated myself from others						
When I was in pain I thrashed						

Score: ________

Investigator Signature: **Date:**

DAY-MONTH-YEAR

THE RELIEF STUDY
RESEARCHING THE EFFECTIVENESS OF LUMBAR INTERVENTIONS FOR ENHANCING FUNCTION
Protocol NIH R01AT006978-01

Place participant ID label here

Participant ID # ___________________

Visit Date _____/_____/______

Visit 2: PROMIS – PAIN INTERFERENCE

To be completed by assessment team investigator

Instructions: Please respond to each item by marking one box per row.

In the past 7 days…

	Not at all	A little bit	Somewhat	Quite a bit	Very much
How much did pain interfere with your enjoyment of life?					
How much did pain interfere with your ability to concentrate?					
How much did pain interfere with your day to day activities?					
How much did pain interfere with your enjoyment of recreational activities?					
How much did pain interfere with doing your tasks away from home (e.g., getting groceries, running errands)?					

	Never	Rarely	Sometimes	Often	Always
How often did pain keep you from socializing with others?					

Score: ________

Investigator Signature: **Date:**

DAY-MONTH-YEAR

Place participant ID label here

Participant ID # ___________________

Visit Date _____/_____/______

Visit 2: PROMIS – PHYSICAL FUNCTION

To be completed by assessment team investigator

Instructions: Please respond to each item by marking one box per row.

	**Not at all**	**Very little**	**Somewhat**	**Quite a bit**	**Cannot do**
Does your health now limit you in doing vigorous activities, such as running, lifting heavy objects, participating in strenuous sports?					
Does your health now limit you in walking more than a mile?					
Does your health now limit you in climbing one flight of stairs?					
Does your health now limit you in lifting or carrying groceries?					
Does your health now limit you in bending, kneeling, or stooping?					

	**Without any difficulty**	**With a little difficulty**	**With some difficulty**	**With much difficulty**	**Unable to do**
Are you able to do chores such as vacuuming or yard work?					
Are you able to dress yourself, including tying shoelaces and doing buttons?					
Are you able to shampoo your hair?					
Are you able to wash and dry your body?					
Are you able to get on and off the toilet?					

Score: ________

Investigator Signature: **Date:**
DAY-MONTH-YEAR

THE RELIEF STUDY
RESEARCHING THE EFFECTIVENESS OF LUMBAR INTERVENTIONS FOR ENHANCING FUNCTION
Protocol NIH R01AT006978-01

Place participant ID label here

Participant ID # ___________________

Visit Date _____/_____/______

Visit 2: CHECKLIST - PHYSIOLOGICAL OUTCOMES – Pre-Intervention

To be completed by assessment team investigator

Experiment 1. Muscular Effects

- ☐ Ensure study participant is safe to enter a magnetic field.
- ☐ Have study participant lie supine for 30-minutes.
- ☐ Obtain and review scout images.
- ☐ Perform The RELIEF Study imaging protocol.
- ☐ Review images and ensure images are high quality.
- ☐ If necessary, repeat the imaging protocol and delete prior file.
- ☐ Save files to MRI terminal hard drive and back-up to cloud.

Experiment 2. Spinal Effects

- ☐ Instrument study participant for EMG.
- ☐ Instrument study participant for motion capture.
- ☐ Perform The RELIEF Study short latency spinal reflex protocol
- ☐ Perform The RELIEF Study standardized reaching protocol.
- ☐ Perform The RELIEF Study trunk perturbation protocol.
- ☐ If necessary repeat a trial and delete prior file.
- ☐ Save files to lab secure network hard drive and back-up to cloud.

Experiment 3. Cortical Effects

- ☐ Instrument study participant for EMG.
- ☐ Perform The RELIEF Study short latency spinal reflex protocol
- ☐ Map and mark the vertex of the skull.
- ☐ Find motor threshold.
- ☐ Perform The RELIEF Study paired pulse TMS protocol.
- ☐ Review signals throughout and ensure they are high quality.
- ☐ If necessary repeat a trial and delete prior file.
- ☐ Save files to lab terminal hard drive and back-up to cloud.

Did the participant complete the experiment?

☐ No

☐ Yes

Comments:

Investigator Signature: **Date:**

DAY-MONTH-YEAR

THE RELIEF STUDY
RESEARCHING THE EFFECTIVENESS OF LUMBAR INTERVENTIONS FOR ENHANCING FUNCTION
Protocol NIH R01AT006978-01

Place participant ID label here

Participant ID # ___________________

Visit Date _____/_____/______

Visit 2 (Intervention 1): INTERVENTION ADMINISTRATION LOG

To be completed by clinician

All fields must be completed	**Symmetry of Active Side Bending**		**L1**	**L2**	**L3**	**L4**	**L5**
**Pre-Intervention Assessment**	SBL cm<br>SBR cm	Transverse Process	FRS-L<br>FRS-R<br>ERS-L<br>ERS-R<br>NORM	FRS-L<br>FRS-R<br>ERS-L<br>ERS-R<br>NORM	FRS-L<br>FRS-R<br>ERS-L<br>ERS-R<br>NORM	FRS-L<br>FRS-R<br>ERS-L<br>ERS-R<br>NORM	FRS-L<br>FRS-R<br>ERS-L<br>ERS-R<br>NORM
		P-A Translation	Hypermobile<br>Hypomobile<br>Normal<br>NA	Hypermobile<br>Hypomobile<br>Normal<br>NA	Hypermobile<br>Hypomobile<br>Normal<br>NA	Hypermobile<br>Hypomobile<br>Normal<br>NA	Hypermobile<br>Hypomobile<br>Normal<br>NA
**Intervention**		# of Repetitions Applied	1 2 3 4<br>NA	1 2 3 4<br>NA	1 2 3 4<br>NA	1 2 3 4<br>NA	1 2 3 4<br>NA
		Audible Response	Yes No<br>NA	Yes No<br>NA	Yes No<br>NA	Yes No<br>NA	Yes No<br>NA
**Post-Intervention Assessment**	SBL cm<br>SBR cm	P-A Translation	Hypermobile<br>Hypomobile<br>Normal<br>NA	Hypermobile<br>Hypomobile<br>Normal<br>NA	Hypermobile<br>Hypomobile<br>Normal<br>NA	Hypermobile<br>Hypomobile<br>Normal<br>NA	Hypermobile<br>Hypomobile<br>Normal<br>NA
		Transverse Process	FRS-L<br>FRS-R<br>ERS-L<br>ERS-R<br>NORM	FRS-L<br>FRS-R<br>ERS-L<br>ERS-R<br>NORM	FRS-L<br>FRS-R<br>ERS-L<br>ERS-R<br>NORM	FRS-L<br>FRS-R<br>ERS-L<br>ERS-R<br>NORM	FRS-L<br>FRS-R<br>ERS-L<br>ERS-R<br>NORM

Notes:

Investigator Signature: **Date:**

DAY-MONTH-YEAR

Place participant ID label here

Participant ID # ___________________

Visit Date _____/_____/______

Visit 2: NUMERIC PAIN RATING SCALE POST-INTERVENTION

To be completed by assessment team investigator

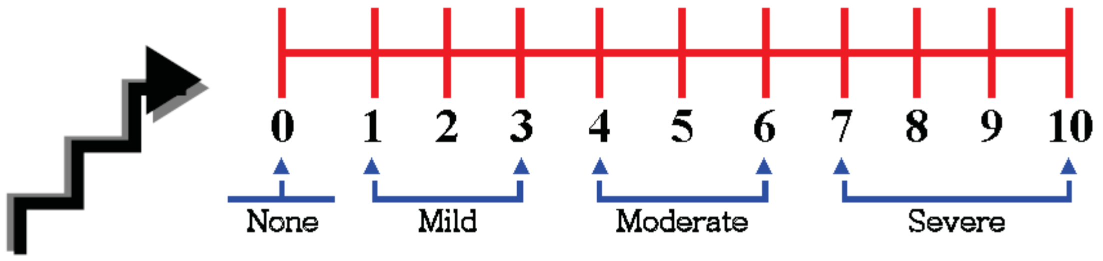

Instructions:
Sometimes it is helpful to think about the Numeric Rating Scale in the following manner:

- 0 = No Pain
- 1-3 = Mild Pain (nagging, annoying, interfering little with your activities of daily living)
- 4–6 = Moderate Pain (interferes significantly with your activities of daily living)
- 7-10 = Severe Pain (disabling; unable to perform your activities of daily living)

Please indicate:
- What number would you give your pain ***right now***? ☐

Investigator Signature: **Date:**

DAY-MONTH-YEAR

Place participant ID label here

Participant ID # ___________________

Visit Date _____/_____/______

Visit 2: CHECKLIST - PHYSIOLOGICAL OUTCOMES – Post-Intervention

To be completed by assessment team investigator

Experiment 1. Muscular Effects

- ☐ Ensure study participant is safe to enter a magnetic field.
- ☐ Have study participant lie supine for 30-minutes.
- ☐ Obtain and review scout images.
- ☐ Perform The RELIEF Study imaging protocol.
- ☐ Review images and ensure images are high quality.
- ☐ If necessary, repeat the imaging protocol and delete prior file.
- ☐ Save files to MRI terminal hard drive and back-up to cloud.

Experiment 2. Spinal Effects

- ☐ Instrument study participant for EMG.
- ☐ Instrument study participant for motion capture.
- ☐ Perform The RELIEF Study short latency spinal reflex protocol
- ☐ Perform The RELIEF Study standardized reaching protocol.
- ☐ Perform The RELIEF Study trunk perturbation protocol.
- ☐ If necessary repeat a trial and delete prior file.
- ☐ Save files to lab secure network hard drive and back-up to cloud.

Experiment 3. Cortical Effects

- ☐ Instrument study participant for EMG.
- ☐ Perform The RELIEF Study short latency spinal reflex protocol
- ☐ Map and mark the vertex of the skull.
- ☐ Find motor threshold.
- ☐ Perform The RELIEF Study paired pulse TMS protocol.
- ☐ Review signals throughout and ensure they are high quality.
- ☐ If necessary repeat a trial and delete prior file.
- ☐ Save files to lab terminal hard drive and back-up to cloud.

Did the participant complete the experiment?

☐ No

☐ Yes

Comments:

Investigator Signature: **Date:**

DAY-MONTH-YEAR

THE RELIEF STUDY
RESEARCHING THE EFFECTIVENESS OF LUMBAR INTERVENTIONS FOR ENHANCING FUNCTION
Protocol NIH R01AT006978-01

Place participant ID label here

Participant ID # ________________

Visit Date _____/_____/______

VISIT 2: CHECKLIST

To be completed by study coordinator

1. Did the participant attend this visit? ☐ Yes (if yes, continue) ☐ No

2. Please check all forms completed at this visit:

   - ☐ Prior and Concomitant Medications
   - ☐ Change in Medication Use
   - ☐ Health Status Reassessment
   - ☐ Contraindications for Lab Testing
   - ☐ Contraindications for Exposure to a Magnetic Field (if applicable)
   - ☐ Contraindications for Intervention
   - ☐ NPR – Numeric Pain Rating - Pre-Intervention
   - ☐ RMDQ - Roland Morris Disability Questionnaire
   - ☐ PROMIS – Pain Intensity
   - ☐ PROMIS – Pain Behavior
   - ☐ PROMIS – Pain Interference
   - ☐ PROMIS – Physical Function
   - ☐ Checklist - Physiological Outcomes (Experiment 1, 2, or 3)
   - ☐ Intervention Administration Log
   - ☐ NPR – Numeric Pain Rating – Post-Intervention
   - ☐ Checklist - Physiological Outcomes (Experiment 1, 2, or 3)

3. Is the participant continuing in the study?

   ☐ No (If no, remember to complete a STUDY COMPLETION form.)
   ☐ Yes (If yes, schedule next visit.)

Comments:

____

Investigator Signature: **Date:**

DAY-MONTH-YEAR

Visit 3

Place participant ID label here

Participant ID # ________________

Visit Date _____/_____/______

Visit 3: PRIOR AND CONCOMITANT MEDICATIONS

To be completed by study coordinator

Have you taken any medications (including over the counter pain relief medications) or used heat or ice for your back pain since your last visit?

☐ No ☐ Yes (If so, record below.)

Number Days since last Visit: ________

Medication/Modality	Frequency and Dose	Indication	Start Date	Stop Date	Ongoing
1.					☐
2.					☐
3.					☐
4.					☐
5.					☐

Investigator Signature: **Date:**

DAY-MONTH-YEAR

THE RELIEF STUDY
RESEARCHING THE EFFECTIVENESS OF LUMBAR INTERVENTIONS FOR ENHANCING FUNCTION
Protocol NIH R01AT006978-01

Place participant ID label here

Participant ID # ___________________

Visit Date _____/_____/______

Visit 3: CHANGE IN MEDICATION USE

To be completed by study coordinator

Have you had any changes in medication use NOT related to your back pain in the past 7 days?

☐ No ☐ Yes (If YES, record below.)

Medication	Indication*	Dose & Frequency	Start Date	Stop Date	Ongoing
1.					☐
2.					☐
3.					☐
4.					☐
5.					☐

*If Indication is related to a negative change in health status, alert study coordinator, complete Health Status Reassessment and enter in Adverse Event Log.

Notes:

Investigator Signature: **Date:**

DAY-MONTH-YEAR

Place participant ID label here

Participant ID # ___________________

Visit Date _____/_____/______

Visit 3: HEALTH STATUS REASSESSMENT

To be completed by study staff

Since your last visit, have you had any change in your health?

☐ No ☐ Yes (If YES, record below, alert study coordinator, and complete Adverse Event log.)

**Change & Symptoms**	
**Describe:**<br><br>**Start Date:**<br>**Stop Date:**<br>**Missed work or school?______days**	**Treatment:**<br><br>**Outcome:**

**Change & Symptoms**	
**Describe:**<br><br>**Start Date:**<br>**Stop Date:**<br>**Missed work or school?______days**	**Treatment:**<br><br>**Outcome:**

**Change & Symptoms**	
**Describe:**<br><br>**Start Date:**<br>**Stop Date:**<br>**Missed work or school?______days**	**Treatment:**<br><br>**Outcome:**

Investigator Signature: **Date:**

DAY-MONTH-YEAR

THE RELIEF STUDY
RESEARCHING THE EFFECTIVENESS OF LUMBAR INTERVENTIONS FOR ENHANCING FUNCTION
Protocol NIH R01AT006978-01

Place participant ID label here

Participant ID # ___________________

Visit Date _____/_____/______

Visit 3: CONTRAINDICATIONS FOR INTERVENTION

To be completed by study staff

**Have you had any of the following symptoms?*:**		**If YES, describe here:**
Radiating pain (burning, numbness, or tingling in the legs)	☐ Yes ☐ No	
Muscle weakness in the legs	☐ Yes ☐ No	
Episodes of incontinence of bowel or bladder	☐ Yes ☐ No	

*If YES, describe in right column, refer to clinician for Health Status Reassessment Physical Exam, and complete Adverse Event Log (forms located in Appendix).

Investigator Signature: **Date:**
DAY-MONTH-YEAR

THE RELIEF STUDY
RESEARCHING THE EFFECTIVENESS OF LUMBAR INTERVENTIONS FOR ENHANCING FUNCTION
Protocol NIH R01AT006978-01

Place participant ID label here

Participant ID # ____________________

Visit Date _____/_____/______

Visit 3: NUMERIC PAIN RATING SCALE

To be completed by assessment team investigator

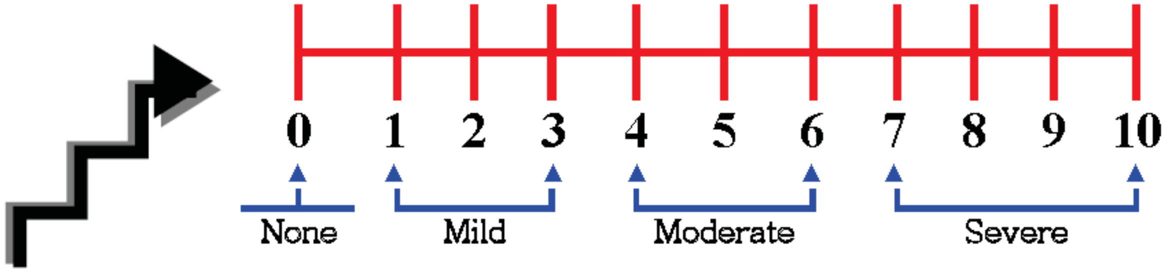

Instructions:
Sometimes it is helpful to think about the Numeric Rating Scale in the following manner:

- 0 = No Pain
- 1-3 = Mild Pain (nagging, annoying, interfering little with your activities of daily living)
- 4–6 = Moderate Pain (interferes significantly with your activities of daily living)
- 7-10 = Severe Pain (disabling; unable to perform your activities of daily living)

Please indicate:

- What number would you give your pain ***right now***? ☐
- What number would describe your pain ***since your last visit***? ☐

Since your last visit how would you answer the next 3 questions:

What number on a 0 to 10 scale would you give your pain when it is the worst that it gets? ☐

- What number on a 0 to 10 scale would you give your pain and when it is the best that it gets? ☐
- At what number is the pain at an acceptable level for you? ☐

Investigator Signature: **Date:**
DAY-MONTH-YEAR

THE RELIEF STUDY
RESEARCHING THE EFFECTIVENESS OF LUMBAR INTERVENTIONS FOR ENHANCING FUNCTION
Protocol NIH R01AT006978-01

Place participant ID label here

Participant ID # __________________

Visit Date _____/_____/______

Visit 3: EXPECTANCY QUESTIONNAIRE

To be completed by assessment team investigator

We would like you to indicate below how much you believe, right now, that the therapy you are receiving will help to reduce your back pain. Belief usually has two aspects to it: (1) what one ***thinks*** will happen and (2) what one ***feels*** will happen. Sometimes these are similar; sometimes they are different. Please answer the questions below.

Set I – Answer in terms of what you *think*.

1. **At this point, how logical does the therapy ordered to you seem?**

   1 2 3 4 5 6 7 8 9
   not at all logical — somewhat logical — very logical

2. **At this point, how successfully do you think this treatment will be in reducing your back pain symptoms?**

   1 2 3 4 5 6 7 8 9
   not at all useful — somewhat useful — very useful

3. **How confident would you be in recommending this treatment to a friend who experiences similar problems?**

   1 2 3 4 5 6 7 8 9
   not at all confident — somewhat confident — very confident

4. **By the end of the therapy period, how much improvement in your pain symptoms do you think will occur?**

   0% 10% 20% 30% 40% 50% 60% 70% 80% 90% 100%

Set II - For this set, close your eyes for a few moments, and try to identify what you really *feel* about the treatment and its likely success. Then answer the following questions.

1. **At this point, how much do you really feel that treatment will help you to reduce your pain symptoms?**

   1 2 3 4 5 6 7 8 9
   not at all — somewhat — Very much

2. **By the end of the therapy period, how much improvement in your pain symptoms do you really feel will occur?**

   0% 10% 20% 30% 40% 50% 60% 70% 80% 90% 100%

Investigator Signature: **Date:**
DAY-MONTH-YEAR

THE RELIEF STUDY
RESEARCHING THE EFFECTIVENESS OF LUMBAR INTERVENTIONS FOR ENHANCING FUNCTION
Protocol NIH R01AT006978-01

Place participant ID label here

Participant ID # ___________________

Visit Date _____/_____/______

Visit 3 (Intervention 2): INTERVENTION ADMINISTRATION LOG

To be completed by clinician

All fields must be completed	**Symmetry of Active Side Bending**		**L1**	**L2**	**L3**	**L4**	**L5**
**Pre-Intervention Assessment**	SBL cm<br>SBR cm	Transverse Process	FRS-L<br>FRS-R<br>ERS-L<br>ERS-R<br>NORM	FRS-L<br>FRS-R<br>ERS-L<br>ERS-R<br>NORM	FRS-L<br>FRS-R<br>ERS-L<br>ERS-R<br>NORM	FRS-L<br>FRS-R<br>ERS-L<br>ERS-R<br>NORM	FRS-L<br>FRS-R<br>ERS-L<br>ERS-R<br>NORM
		P-A Translation	Hypermobile<br>Hypomobile<br>Normal<br>NA	Hypermobile<br>Hypomobile<br>Normal<br>NA	Hypermobile<br>Hypomobile<br>Normal<br>NA	Hypermobile<br>Hypomobile<br>Normal<br>NA	Hypermobile<br>Hypomobile<br>Normal<br>NA
**Intervention**		# of Thrusts Applied	1 2 3 4<br>NA	1 2 3 4<br>NA	1 2 3 4<br>NA	1 2 3 4<br>NA	1 2 3 4<br>NA
		Audible Response	Yes No<br>NA	Yes No<br>NA	Yes No<br>NA	Yes No<br>NA	Yes No<br>NA
**Post-Intervention Assessment**	SBL cm<br>SBR cm	P-A Translation	Hypermobile<br>Hypomobile<br>Normal<br>NA	Hypermobile<br>Hypomobile<br>Normal<br>NA	Hypermobile<br>Hypomobile<br>Normal<br>NA	Hypermobile<br>Hypomobile<br>Normal<br>NA	Hypermobile<br>Hypomobile<br>Normal<br>NA
		Transverse Process	FRS-L<br>FRS-R<br>ERS-L<br>ERS-R<br>NORM	FRS-L<br>FRS-R<br>ERS-L<br>ERS-R<br>NORM	FRS-L<br>FRS-R<br>ERS-L<br>ERS-R<br>NORM	FRS-L<br>FRS-R<br>ERS-L<br>ERS-R<br>NORM	FRS-L<br>FRS-R<br>ERS-L<br>ERS-R<br>NORM

Notes:

Investigator Signature: **Date:**

DAY-MONTH-YEAR

Place participant ID label here

Participant ID # ___________________

Visit Date _____/_____/______

VISIT 3: CHECKLIST

To be completed by study coordinator

1. Did the participant attend this visit? ☐ Yes (if yes, continue) ☐ No
2. Please check all assessments completed at this visit:

Visit Name: Intervention Checklist

☐ Prior and Concomitant Medications

☐ Change in Medications

☐ Health Status Reassessment

☐ Contraindications for Intervention

☐ NPR – Numeric Pain Rating

☐ Expectancy Questionnaire

☐ Intervention Administration Log

3. Is the participant continuing in the study?

Investigator Signature: **Date:**

DAY-MONTH-YEAR

Place participant ID label here

Participant ID # ________________

Visit Date _____/_____/______

☐ No (If no, remember to complete a STUDY COMPLETION form.)

☐ Yes (If yes, schedule next visit.)

Investigator Signature: **Date:**

DAY-MONTH-YEAR

Visit 4

THE RELIEF STUDY
RESEARCHING THE EFFECTIVENESS OF LUMBAR INTERVENTIONS FOR ENHANCING FUNCTION
Protocol NIH R01AT006978-01

Place participant ID label here

Participant ID # ________________

Visit Date _____/_____/______

Visit 4: PRIOR AND CONCOMITANT MEDICATIONS

To be completed by study coordinator

Have you taken any medications (including over the counter pain relief medications) or used heat or ice for your back pain since your last visit?

☐ No ☐ Yes (If so, record below.)

Number Days since last Visit: ________

Medication/Modality	Frequency and Dose	Indication	Start Date	Stop Date	Ongoing
1.					☐
2.					☐
3.					☐
4.					☐
5.					☐

Investigator Signature: **Date:**

DAY-MONTH-YEAR

THE RELIEF STUDY
RESEARCHING THE EFFECTIVENESS OF LUMBAR INTERVENTIONS FOR ENHANCING FUNCTION
Protocol NIH R01AT006978-01

Place participant ID label here

Participant ID # ________________

Visit Date _____/_____/______

Visit 4: CHANGE IN MEDICATION USE

To be completed by study coordinator

Have you had any changes in medication use NOT related to your back pain in the past 7 days?

☐ No ☐ Yes (If YES, record below.)

Medication	Indication*	Dose & Frequency	Start Date	Stop Date	Ongoing
1.					☐
2.					☐
3.					☐
4.					☐
5.					☐

*If Indication is related to a negative change in health status, alert study coordinator, complete Health Status Reassessment and enter in Adverse Event Log.

Notes:

Investigator Signature: **Date:**

DAY-MONTH-YEAR

THE RELIEF STUDY
RESEARCHING THE EFFECTIVENESS OF LUMBAR INTERVENTIONS FOR ENHANCING FUNCTION
Protocol NIH R01AT006978-01

Place participant ID label here

Participant ID # ___________________

Visit Date _____/_____/______

Visit 4: HEALTH STATUS REASSESSMENT

To be completed by study staff

Since your last visit, have you had any change in your health?

☐ No ☐ Yes (If YES, record below, alert study coordinator, and complete Adverse Event log.)

**Change & Symptoms**	
**Describe:**<br><br>**Start Date:**<br>**Stop Date:**<br>**Missed work or school?______days**	**Treatment:**<br><br>**Outcome:**

**Change & Symptoms**	
**Describe:**<br><br>**Start Date:**<br>**Stop Date:**<br>**Missed work or school?______days**	**Treatment:**<br><br>**Outcome:**

**Change & Symptoms**	
**Describe:**<br><br>**Start Date:**<br>**Stop Date:**<br>**Missed work or school?______days**	**Treatment:**<br><br>**Outcome:**

Investigator Signature: **Date:**

DAY-MONTH-YEAR

THE RELIEF STUDY
RESEARCHING THE EFFECTIVENESS OF LUMBAR INTERVENTIONS FOR ENHANCING FUNCTION
Protocol NIH R01AT006978-01

Place participant ID label here

Participant ID # ___________________

Visit Date _____/_____/______

Visit 4: CONTRAINDICATIONS FOR INTERVENTION

To be completed by study staff

**Have you had any of the following symptoms?*:**		**If YES, describe here:**
Radiating pain (burning, numbness, or tingling in the legs)	☐ Yes ☐ No	
Muscle weakness in the legs	☐ Yes ☐ No	
Episodes of incontinence of bowel or bladder	☐ Yes ☐ No	

*If YES, describe in right column, refer to clinician for Health Status Reassessment Physical Exam, and complete Adverse Event Log (forms located in Appendix).

Investigator Signature: **Date:**
DAY-MONTH-YEAR

THE RELIEF STUDY
RESEARCHING THE EFFECTIVENESS OF LUMBAR INTERVENTIONS FOR ENHANCING FUNCTION
Protocol NIH R01AT006978-01

Place participant ID label here

Participant ID # ___________________

Visit Date _____/_____/______

Visit 4: NUMERIC PAIN RATING SCALE (NPR)

To be completed by assessment team investigator

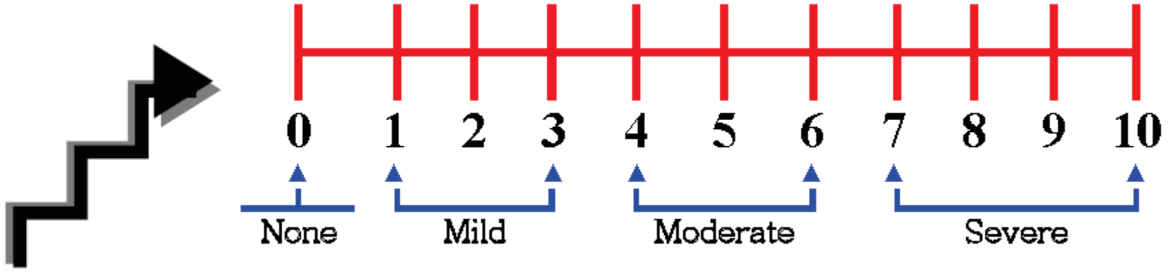

Instructions:
Sometimes it is helpful to think about the Numeric Rating Scale in the following manner:

- 0 = No Pain
- 1-3 = Mild Pain (nagging, annoying, interfering little with your activities of daily living)
- 4–6 = Moderate Pain (interferes significantly with your activities of daily living)
- 7-10 = Severe Pain (disabling; unable to perform your activities of daily living)

Please indicate:

- What number would you give your pain ***right now***? ☐
- What number would describe your pain ***since your last visit***? ☐

Since your last visit how would you answer the next 3 questions:

What number on a 0 to 10 scale would you give your pain when it is the worst that it gets? ☐

- What number on a 0 to 10 scale would you give your pain and when it is the best that it gets? ☐
- At what number is the pain at an acceptable level for you? ☐

Investigator Signature: **Date:**

DAY-MONTH-YEAR

THE RELIEF STUDY
RESEARCHING THE EFFECTIVENESS OF LUMBAR INTERVENTIONS FOR ENHANCING FUNCTION
Protocol NIH R01AT006978-01

Place participant ID label here

Participant ID # __________________

Visit Date _____/_____/______

Visit 4 (Intervention 3): INTERVENTION ADMINISTRATION LOG

To be completed by clinician

All fields must be completed	**Symmetry of Active Side Bending**		**L1**	**L2**	**L3**	**L4**	**L5**
**Pre-Intervention Assessment**	SBL cm<br>SBR cm	Transverse Process	FRS-L<br>FRS-R<br>ERS-L<br>ERS-R<br>NORM	FRS-L<br>FRS-R<br>ERS-L<br>ERS-R<br>NORM	FRS-L<br>FRS-R<br>ERS-L<br>ERS-R<br>NORM	FRS-L<br>FRS-R<br>ERS-L<br>ERS-R<br>NORM	FRS-L<br>FRS-R<br>ERS-L<br>ERS-R<br>NORM
		P-A Translation	Hypermobile<br>Hypomobile<br>Normal<br>NA	Hypermobile<br>Hypomobile<br>Normal<br>NA	Hypermobile<br>Hypomobile<br>Normal<br>NA	Hypermobile<br>Hypomobile<br>Normal<br>NA	Hypermobile<br>Hypomobile<br>Normal<br>NA
**Intervention**		# of Thrusts Applied	1 2 3 4<br>NA	1 2 3 4<br>NA	1 2 3 4<br>NA	1 2 3 4<br>NA	1 2 3 4<br>NA
		Audible Response	Yes No<br>NA	Yes No<br>NA	Yes No<br>NA	Yes No<br>NA	Yes No<br>NA
**Post-Intervention Assessment**	SBL cm<br>SBR cm	P-A Translation	Hypermobile<br>Hypomobile<br>Normal<br>NA	Hypermobile<br>Hypomobile<br>Normal<br>NA	Hypermobile<br>Hypomobile<br>Normal<br>NA	Hypermobile<br>Hypomobile<br>Normal<br>NA	Hypermobile<br>Hypomobile<br>Normal<br>NA
		Transverse Process	FRS-L<br>FRS-R<br>ERS-L<br>ERS-R<br>NORM	FRS-L<br>FRS-R<br>ERS-L<br>ERS-R<br>NORM	FRS-L<br>FRS-R<br>ERS-L<br>ERS-R<br>NORM	FRS-L<br>FRS-R<br>ERS-L<br>ERS-R<br>NORM	FRS-L<br>FRS-R<br>ERS-L<br>ERS-R<br>NORM

Notes:

Investigator Signature: **Date:**

DAY-MONTH-YEAR

THE RELIEF STUDY
RESEARCHING THE EFFECTIVENESS OF LUMBAR INTERVENTIONS FOR ENHANCING FUNCTION
Protocol NIH R01AT006978-01

Place participant ID label here

Participant ID # ___________________

Visit Date _____/_____/______

VISIT 4: CHECKLIST

To be completed by study coordinator

4. Did the participant attend this visit? ☐ Yes (if yes, continue) ☐ No
5. Please check all assessments completed at this visit:

Visit Name: Intervention Checklist

☐ Prior and Concomitant Medications

☐ Change in Medications

☐ Health Status Reassessment

☐ Contraindications for Intervention

☐ NPR – Numeric Pain Rating

☐ Intervention Administration Log

6. Is the participant continuing in the study?

☐ No (If no, remember to complete a STUDY COMPLETION form.)

☐ Yes (If yes, schedule next visit.)

Investigator Signature: **Date:**

DAY-MONTH-YEAR

Place participant ID label here

Participant ID # ________________

Visit Date _____/_____/______

Comments:

____

Investigator Signature: **Date:** ______________________
DAY-MONTH-YEAR

Visit 5

Investigator Signature: Date:
DAY-MONTH-YEAR

Place participant ID label here

Participant ID # ________________

Visit Date _____/_____/______

Visit 5: PRIOR AND CONCOMITANT MEDICATIONS

To be completed by study coordinator

Have you taken any medications (including over the counter pain relief medications) or used heat or ice for your back pain since your last visit?

☐ No ☐ Yes (If so, record below.)

Number Days since last Visit: ________

Medication/Modality	Frequency and Dose	Indication	Start Date	Stop Date	Ongoing
1.					☐
2.					☐
3.					☐
4.					☐
5.					☐

Investigator Signature: **Date:**

DAY-MONTH-YEAR

THE RELIEF STUDY
RESEARCHING THE EFFECTIVENESS OF LUMBAR INTERVENTIONS FOR ENHANCING FUNCTION
Protocol NIH R01AT006978-01

Place participant ID label here

Participant ID # ________________

Visit Date _____/_____/______

Visit 5: CHANGE IN MEDICATION USE

To be completed by study coordinator

Have you had any changes in medication use NOT related to your back pain in the past 7 days?
☐ No ☐ Yes (If YES, record below.)

Medication	Indication*	Dose & Frequency	Start Date	Stop Date	Ongoing
1.					☐
2.					☐
3.					☐
4.					☐
5.					☐

*If Indication is related to a negative change in health status, alert study coordinator, complete Health Status Reassessment and enter in Adverse Event Log.

Notes:

Investigator Signature: **Date:**
DAY-MONTH-YEAR

Place participant ID label here

Participant ID # ___________________

Visit Date _____/_____/______

Visit 5: HEALTH STATUS REASSESSMENT

To be completed by study staff

Since your last visit, have you had any change in your health?

☐ No ☐ Yes (If YES, record below, alert study coordinator, and complete Adverse Event log.)

**Change & Symptoms**	
**Describe:**<br><br>**Start Date:**<br>**Stop Date:**<br>**Missed work or school?______days**	**Treatment:**<br><br>**Outcome:**

**Change & Symptoms**	
**Describe:**<br><br>**Start Date:**<br>**Stop Date:**<br>**Missed work or school?______days**	**Treatment:**<br><br>**Outcome:**

**Change & Symptoms**	
**Describe:**<br><br>**Start Date:**<br>**Stop Date:**<br>**Missed work or school?______days**	**Treatment:**<br><br>**Outcome:**

Investigator Signature: **Date:**

DAY-MONTH-YEAR

THE RELIEF STUDY
RESEARCHING THE EFFECTIVENESS OF LUMBAR INTERVENTIONS FOR ENHANCING FUNCTION
Protocol NIH R01AT006978-01

Place participant ID label here

Participant ID # ________________

Visit Date _____/_____/______

Visit 5: CONTRAINDICATIONS FOR INTERVENTION

To be completed by study staff

**Have you had any of the following symptoms?*:**		**If YES, describe here:**
Radiating pain (burning, numbness, or tingling in the legs)	☐ Yes ☐ No	
Muscle weakness in the legs	☐ Yes ☐ No	
Episodes of incontinence of bowel or bladder	☐ Yes ☐ No	

*If YES, describe in right column, refer to clinician for Health Status Reassessment Physical Exam, and complete Adverse Event Log (forms located in Appendix).

Investigator Signature: **Date:**
DAY-MONTH-YEAR

THE RELIEF STUDY
RESEARCHING THE EFFECTIVENESS OF LUMBAR INTERVENTIONS FOR ENHANCING FUNCTION
Protocol NIH R01AT006978-01

Place participant ID label here

Participant ID # ___________________

Visit Date _____/_____/______

Visit 5: NUMERIC PAIN RATING SCALE (NPR)

To be completed by assessment team member

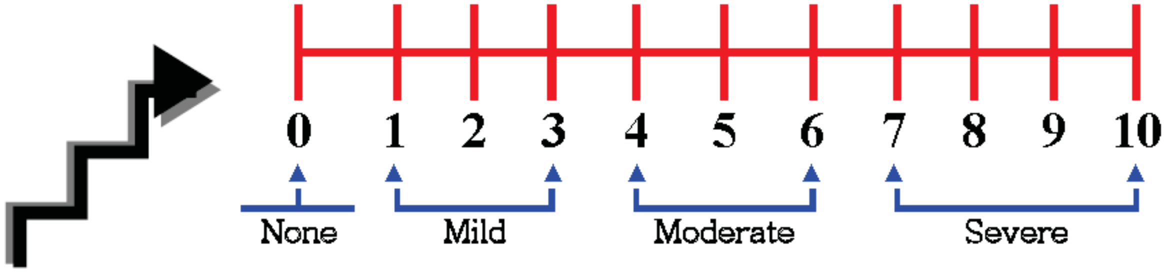

Instructions:
Sometimes it is helpful to think about the Numeric Rating Scale in the following manner:

- 0 = No Pain
- 1-3 = Mild Pain (nagging, annoying, interfering little with your activities of daily living)
- 4–6 = Moderate Pain (interferes significantly with your activities of daily living)
- 7-10 = Severe Pain (disabling; unable to perform your activities of daily living)

Please indicate:

- What number would you give your pain ***right now***? ☐
- What number would describe your pain ***since your last visit***? ☐

Since your last visit how would you answer the next 3 questions:

What number on a 0 to 10 scale would you give your pain when it is the worst that it gets? ☐

- What number on a 0 to 10 scale would you give your pain and when it is the best that it gets? ☐
- At what number is the pain at an acceptable level for you? ☐

Investigator Signature: **Date:**

DAY-MONTH-YEAR

THE RELIEF STUDY
RESEARCHING THE EFFECTIVENESS OF LUMBAR INTERVENTIONS FOR ENHANCING FUNCTION
Protocol NIH R01AT006978-01

Place participant ID label here

Participant ID # ________________

Visit Date _____/_____/______

Visit 5 (Intervention 4): INTERVENTION ADMINISTRATION LOG

To be completed by clinician

All fields must be completed	**Symmetry of Active Side Bending**		**L1**	**L2**	**L3**	**L4**	**L5**
**Pre-Intervention Assessment**	SBL cm<br>SBR cm	Transverse Process	FRS-L<br>FRS-R<br>ERS-L<br>ERS-R<br>NORM	FRS-L<br>FRS-R<br>ERS-L<br>ERS-R<br>NORM	FRS-L<br>FRS-R<br>ERS-L<br>ERS-R<br>NORM	FRS-L<br>FRS-R<br>ERS-L<br>ERS-R<br>NORM	FRS-L<br>FRS-R<br>ERS-L<br>ERS-R<br>NORM
		P-A Translation	Hypermobile<br>Hypomobile<br>Normal<br>NA	Hypermobile<br>Hypomobile<br>Normal<br>NA	Hypermobile<br>Hypomobile<br>Normal<br>NA	Hypermobile<br>Hypomobile<br>Normal<br>NA	Hypermobile<br>Hypomobile<br>Normal<br>NA
**Intervention**		# of Thrusts Applied	1 2 3 4<br>NA	1 2 3 4<br>NA	1 2 3 4<br>NA	1 2 3 4<br>NA	1 2 3 4<br>NA
		Audible Response	Yes No<br>NA	Yes No<br>NA	Yes No<br>NA	Yes No<br>NA	Yes No<br>NA
**Post-Intervention Assessment**	SBL cm<br>SBR cm	P-A Translation	Hypermobile<br>Hypomobile<br>Normal<br>NA	Hypermobile<br>Hypomobile<br>Normal<br>NA	Hypermobile<br>Hypomobile<br>Normal<br>NA	Hypermobile<br>Hypomobile<br>Normal<br>NA	Hypermobile<br>Hypomobile<br>Normal<br>NA
		Transverse Process	FRS-L<br>FRS-R<br>ERS-L<br>ERS-R<br>NORM	FRS-L<br>FRS-R<br>ERS-L<br>ERS-R<br>NORM	FRS-L<br>FRS-R<br>ERS-L<br>ERS-R<br>NORM	FRS-L<br>FRS-R<br>ERS-L<br>ERS-R<br>NORM	FRS-L<br>FRS-R<br>ERS-L<br>ERS-R<br>NORM

Notes:

Investigator Signature: **Date:**

DAY-MONTH-YEAR

Place participant ID label here

Participant ID # ___________________

Visit Date _____/_____/______

VISIT 5: CHECKLIST

To be completed by study coordinator

7. Did the participant attend this visit? ☐ Yes (if yes, continue) ☐ No
8. Please check all assessments completed at this visit:

Visit Name: Intervention Checklist

☐ Prior and Concomitant Medications

☐ Change in Medications

☐ Health Status Reassessment

☐ Contraindications for Intervention

☐ NPR – Numeric Pain Rating

☐ Intervention Administration Log

9. Is the participant continuing in the study?

☐ No (If no, remember to complete a STUDY COMPLETION form.)

☐ Yes (If yes, schedule next visit.)

Comments:

____

Investigator Signature: **Date:**

DAY-MONTH-YEAR

Visit 6

THE RELIEF STUDY
RESEARCHING THE EFFECTIVENESS OF LUMBAR INTERVENTIONS FOR ENHANCING FUNCTION
Protocol NIH R01AT006978-01

Place participant ID label here

Participant ID # ________________

Visit Date _____/_____/______

Visit 6: PRIOR AND CONCOMITANT MEDICATIONS

To be completed by study coordinator

Have you taken any medications (including over the counter pain relief medications) or used heat or ice for your back pain since your last visit?

☐ No ☐ Yes (If so, record below.)

Number Days since last Visit: ________

Medication/Modality	Frequency and Dose	Indication	Start Date	Stop Date	Ongoing
1.					☐
2.					☐
3.					☐
4.					☐
5.					☐

Investigator Signature: **Date:**

DAY-MONTH-YEAR

THE RELIEF STUDY
RESEARCHING THE EFFECTIVENESS OF LUMBAR INTERVENTIONS FOR ENHANCING FUNCTION
Protocol NIH R01AT006978-01

Place participant ID label here

Participant ID # ________________

Visit Date _____/_____/______

Visit 6: CHANGE IN MEDICATION USE

To be completed by study coordinator

Have you had any changes in medication use NOT related to your back pain in the past 7 days?

☐ No ☐ Yes (If YES, record below.)

Medication	Indication*	Dose & Frequency	Start Date	Stop Date	Ongoing
1.					☐
2.					☐
3.					☐
4.					☐
5.					☐

*If Indication is related to a negative change in health status, alert study coordinator, complete Health Status Reassessment and enter in Adverse Event Log.

Notes:

Investigator Signature: **Date:**

DAY-MONTH-YEAR

THE RELIEF STUDY
RESEARCHING THE EFFECTIVENESS OF LUMBAR INTERVENTIONS FOR ENHANCING FUNCTION
Protocol NIH R01AT006978-01

Place participant ID label here

Participant ID # ___________________

Visit Date _____/_____/______

Visit 6: HEALTH STATUS REASSESSMENT

To be completed by study staff

Since your last visit, have you had any change in your health?

☐ No ☐ Yes (If YES, record below, alert study coordinator, and complete Adverse Event log.)

**Change & Symptoms**	
**Describe:**<br><br>**Start Date:**<br>**Stop Date:**<br>**Missed work or school?______days**	**Treatment:**<br><br>**Outcome:**

**Change & Symptoms**	
**Describe:**<br><br>**Start Date:**<br>**Stop Date:**<br>**Missed work or school?______days**	**Treatment:**<br><br>**Outcome:**

**Change & Symptoms**	
**Describe:**<br><br>**Start Date:**<br>**Stop Date:**<br>**Missed work or school?______days**	**Treatment:**<br><br>**Outcome:**

Investigator Signature: **Date:**

DAY-MONTH-YEAR

THE RELIEF STUDY
RESEARCHING THE EFFECTIVENESS OF LUMBAR INTERVENTIONS FOR ENHANCING FUNCTION
Protocol NIH R01AT006978-01

Place participant ID label here

Participant ID # ________________

Visit Date _____/_____/______

Visit 6: CONTRAINDICATIONS FOR INTERVENTION

To be completed by study staff

**Have you had any of the following symptoms?*:**		**If YES, describe here:**
Radiating pain (burning, numbness, or tingling in the legs)	☐ Yes ☐ No	
Muscle weakness in the legs	☐ Yes ☐ No	
Episodes of incontinence of bowel or bladder	☐ Yes ☐ No	

*If YES, describe in right column, refer to clinician for Health Status Reassessment Physical Exam, and complete Adverse Event Log (forms located in Appendix).

Investigator Signature: **Date:**
DAY-MONTH-YEAR

THE RELIEF STUDY
RESEARCHING THE EFFECTIVENESS OF LUMBAR INTERVENTIONS FOR ENHANCING FUNCTION
Protocol NIH R01AT006978-01

Place participant ID label here

Participant ID # ___________________

Visit Date _____/_____/______

Visit 6: NUMERIC PAIN RATING SCALE (NPR)

To be completed by assessment team member

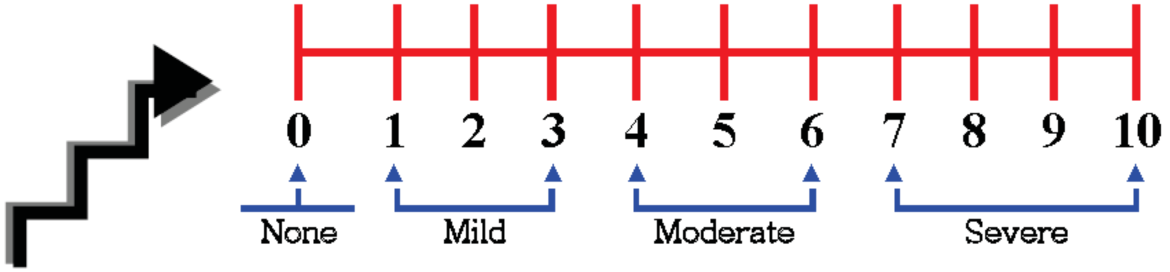

Instructions:
Sometimes it is helpful to think about the Numeric Rating Scale in the following manner:

- 0 = No Pain
- 1-3 = Mild Pain (nagging, annoying, interfering little with your activities of daily living)
- 4–6 = Moderate Pain (interferes significantly with your activities of daily living)
- 7-10 = Severe Pain (disabling; unable to perform your activities of daily living)

Please indicate:

- What number would you give your pain ***right now***? ☐
- What number would describe your pain ***since your last visit***? ☐

Since your last visit how would you answer the next 3 questions:

What number on a 0 to 10 scale would you give your pain when it is the worst that it gets? ☐

- What number on a 0 to 10 scale would you give your pain and when it is the best that it gets? ☐
- At what number is the pain at an acceptable level for you? ☐

Investigator Signature: **Date:**

DAY-MONTH-YEAR

OHIO UNIVERSITY   OHIO UNIVERSITY | OHIO MUSCULOSKELETAL AND NEUROLOGICAL INSTITUTE Heritage College of Osteopathic Medicine

THE RELIEF STUDY
RESEARCHING THE EFFECTIVENESS OF LUMBAR INTERVENTIONS FOR ENHANCING FUNCTION
Protocol NIH R01AT006978-01

Place participant ID label here

Participant ID # ___________________

Visit Date _____/_____/______

Visit 6 (Intervention 5): INTERVENTION ADMINISTRATION LOG

To be completed by clinician

All fields must be completed	**Symmetry of Active Side Bending**		**L1**	**L2**	**L3**	**L4**	**L5**
**Pre-Intervention Assessment**	SBL<br>cm<br><br>SBR<br>cm	Transverse Process	FRS-L<br>FRS-R<br>ERS-L<br>ERS-R<br>NORM	FRS-L<br>FRS-R<br>ERS-L<br>ERS-R<br>NORM	FRS-L<br>FRS-R<br>ERS-L<br>ERS-R<br>NORM	FRS-L<br>FRS-R<br>ERS-L<br>ERS-R<br>NORM	FRS-L<br>FRS-R<br>ERS-L<br>ERS-R<br>NORM
		P-A Translation	Hypermobile<br>Hypomobile<br>Normal<br>NA	Hypermobile<br>Hypomobile<br>Normal<br>NA	Hypermobile<br>Hypomobile<br>Normal<br>NA	Hypermobile<br>Hypomobile<br>Normal<br>NA	Hypermobile<br>Hypomobile<br>Normal<br>NA
**Intervention**		# of Thrusts Applied	1 2 3 4<br>NA	1 2 3 4<br>NA	1 2 3 4<br>NA	1 2 3 4<br>NA	1 2 3 4<br>NA
		Audible Response	Yes No<br>NA	Yes No<br>NA	Yes No<br>NA	Yes No<br>NA	Yes No<br>NA
**Post-Intervention Assessment**	SBL<br>cm<br><br>SBR<br>cm	P-A Translation	Hypermobile<br>Hypomobile<br>Normal<br>NA	Hypermobile<br>Hypomobile<br>Normal<br>NA	Hypermobile<br>Hypomobile<br>Normal<br>NA	Hypermobile<br>Hypomobile<br>Normal<br>NA	Hypermobile<br>Hypomobile<br>Normal<br>NA
		Transverse Process	FRS-L<br>FRS-R<br>ERS-L<br>ERS-R<br>NORM	FRS-L<br>FRS-R<br>ERS-L<br>ERS-R<br>NORM	FRS-L<br>FRS-R<br>ERS-L<br>ERS-R<br>NORM	FRS-L<br>FRS-R<br>ERS-L<br>ERS-R<br>NORM	FRS-L<br>FRS-R<br>ERS-L<br>ERS-R<br>NORM

Notes:

Investigator Signature: **Date:**
DAY-MONTH-YEAR

THE RELIEF STUDY
RESEARCHING THE EFFECTIVENESS OF LUMBAR INTERVENTIONS FOR ENHANCING FUNCTION
Protocol NIH R01AT006978-01

Place participant ID label here

Participant ID # ___________________

Visit Date _____/_____/______

VISIT 6: CHECKLIST

To be completed by study coordinator

10. Did the participant attend this visit? ☐ Yes (if yes, continue) ☐ No
11. Please check all assessments completed at this visit:

Visit Name: Intervention Checklist

☐ Prior and Concomitant Medications

☐ Change in Medications

☐ Health Status Reassessment

☐ Contraindications for Intervention

☐ NPR – Numeric Pain Rating

☐ Intervention Administration Log

12. Is the participant continuing in the study?

☐ No (If no, remember to complete a STUDY COMPLETION form.)

☐ Yes (If yes, schedule next visit.)

Comments:

____

Investigator Signature: **Date:**

DAY-MONTH-YEAR

Visit 7

THE RELIEF STUDY
RESEARCHING THE EFFECTIVENESS OF LUMBAR INTERVENTIONS FOR ENHANCING FUNCTION
Protocol NIH R01AT006978-01

Place participant ID label here

Participant ID # ________________

Visit Date _____/_____/______

Visit 7: PRIOR AND CONCOMITANT MEDICATIONS

To be completed by study coordinator

Have you taken any medications (including over the counter pain relief medications) or used heat or ice for your back pain since your last visit?

☐ No ☐ Yes (If so, record below.)

Number Days since last Visit:________

Medication/Modality	Frequency and Dose	Indication	Start Date	Stop Date	Ongoing
1.					☐
2.					☐
3.					☐
4.					☐
5.					☐

Investigator Signature: **Date:**

DAY-MONTH-YEAR

THE RELIEF STUDY
RESEARCHING THE EFFECTIVENESS OF LUMBAR INTERVENTIONS FOR ENHANCING FUNCTION
Protocol NIH R01AT006978-01

Place participant ID label here

Participant ID # ________________

Visit Date _____/_____/______

Visit 7: CHANGE IN MEDICATION USE

To be completed by study coordinator

Have you had any changes in medication use NOT related to your back pain in the past 7 days?

☐ No ☐ Yes (If YES, record below.)

Medication	Indication*	Dose & Frequency	Start Date	Stop Date	Ongoing
1.					☐
2.					☐
3.					☐
4.					☐
5.					☐

*If Indication is related to a negative change in health status, alert study coordinator, complete Health Status Reassessment and enter in Adverse Event Log.

Notes:

Investigator Signature: **Date:**

DAY-MONTH-YEAR

THE RELIEF STUDY
RESEARCHING THE EFFECTIVENESS OF LUMBAR INTERVENTIONS FOR ENHANCING FUNCTION
Protocol NIH R01AT006978-01

Place participant ID label here

Participant ID # ___________________

Visit Date _____/_____/______

Visit 7: HEALTH STATUS REASSESSMENT

To be completed by study staff

Since your last visit, have you had any change in your health?

☐ No ☐ Yes (If YES, record below, alert study coordinator, and complete Adverse Event log.)

**Change & Symptoms**	
**Describe:**<br><br>**Start Date:**<br>**Stop Date:**<br>**Missed work or school?______days**	**Treatment:**<br><br>**Outcome:**

**Change & Symptoms**	
**Describe:**<br><br>**Start Date:**<br>**Stop Date:**<br>**Missed work or school?______days**	**Treatment:**<br><br>**Outcome:**

**Change & Symptoms**	
**Describe:**<br><br>**Start Date:**<br>**Stop Date:**<br>**Missed work or school?______days**	**Treatment:**<br><br>**Outcome:**

Investigator Signature: **Date:**

DAY-MONTH-YEAR

Place participant ID label here

Participant ID # ___________________

Visit Date _____/_____/______

Visit 7: CONTRAINDICATIONS FOR INTERVENTION

To be completed by study staff

**Have you had any of the following symptoms?*:**		**If YES, describe here:**
Radiating pain (burning, numbness, or tingling in the legs)	☐ Yes ☐ No	
Muscle weakness in the legs	☐ Yes ☐ No	
Episodes of incontinence of bowel or bladder	☐ Yes ☐ No	

*If YES, describe in right column, refer to clinician for Health Status Reassessment Physical Exam, and complete Adverse Event Log (forms located in Appendix).

Investigator Signature: **Date:**

DAY-MONTH-YEAR

THE RELIEF STUDY
RESEARCHING THE EFFECTIVENESS OF LUMBAR INTERVENTIONS FOR ENHANCING FUNCTION
Protocol NIH R01AT006978-01

Place participant ID label here

Participant ID # ___________________

Visit Date _____/_____/______

Visit 7: NUMERIC PAIN RATING SCALE (NPR)

To be completed by assessment team member

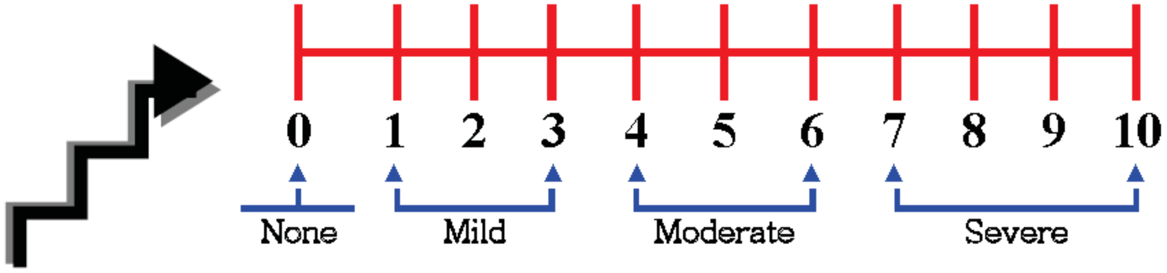

Instructions:
Sometimes it is helpful to think about the Numeric Rating Scale in the following manner:

- 0 = No Pain
- 1-3 = Mild Pain (nagging, annoying, interfering little with your activities of daily living)
- 4–6 = Moderate Pain (interferes significantly with your activities of daily living)
- 7-10 = Severe Pain (disabling; unable to perform your activities of daily living)

Please indicate:

- What number would you give your pain ***right now***? ☐
- What number would describe your pain ***since your last visit***? ☐

Since your last visit how would you answer the next 3 questions:

What number on a 0 to 10 scale would you give your pain when it is the worst that it gets? ☐

- What number on a 0 to 10 scale would you give your pain and when it is the best that it gets? ☐
- At what number is the pain at an acceptable level for you? ☐

Investigator Signature: **Date:**

DAY-MONTH-YEAR

THE RELIEF STUDY
RESEARCHING THE EFFECTIVENESS OF LUMBAR INTERVENTIONS FOR ENHANCING FUNCTION
Protocol NIH R01AT006978-01

Place participant ID label here

Participant ID # ___________________

Visit Date _____/_____/______

Visit 7 (Intervention 6): INTERVENTION ADMINISTRATION LOG

To be completed by clinician

<table>
<tr><th>All fields must be completed</th><th>Symmetry of Active Side Bending</th><th></th><th>L1</th><th>L2</th><th>L3</th><th>L4</th><th>L5</th></tr>
<tr><td rowspan="2">Pre-Intervention Assessment</td><td rowspan="2">SBL cm
SBR cm</td><td>Transverse Process</td><td>FRS-L
FRS-R
ERS-L
ERS-R
NORM</td><td>FRS-L
FRS-R
ERS-L
ERS-R
NORM</td><td>FRS-L
FRS-R
ERS-L
ERS-R
NORM</td><td>FRS-L
FRS-R
ERS-L
ERS-R
NORM</td><td>FRS-L
FRS-R
ERS-L
ERS-R
NORM</td></tr>
<tr><td>P-A Translation</td><td>Hypermobile
Hypomobile
Normal
NA</td><td>Hypermobile
Hypomobile
Normal
NA</td><td>Hypermobile
Hypomobile
Normal
NA</td><td>Hypermobile
Hypomobile
Normal
NA</td><td>Hypermobile
Hypomobile
Normal
NA</td></tr>
<tr><td colspan="2" rowspan="2">Intervention</td><td># of Thrusts Applied</td><td>1 2 3 4
NA</td><td>1 2 3 4
NA</td><td>1 2 3 4
NA</td><td>1 2 3 4
NA</td><td>1 2 3 4
NA</td></tr>
<tr><td>Audible Response</td><td>Yes No
NA</td><td>Yes No
NA</td><td>Yes No
NA</td><td>Yes No
NA</td><td>Yes No
NA</td></tr>
<tr><td rowspan="2">Post-Intervention Assessment</td><td rowspan="2">SBL cm
SBR cm</td><td>P-A Translation</td><td>Hypermobile
Hypomobile
Normal
NA</td><td>Hypermobile
Hypomobile
Normal
NA</td><td>Hypermobile
Hypomobile
Normal
NA</td><td>Hypermobile
Hypomobile
Normal
NA</td><td>Hypermobile
Hypomobile
Normal
NA</td></tr>
<tr><td>Transverse Process</td><td>FRS-L
FRS-R
ERS-L
ERS-R
NORM</td><td>FRS-L
FRS-R
ERS-L
ERS-R
NORM</td><td>FRS-L
FRS-R
ERS-L
ERS-R
NORM</td><td>FRS-L
FRS-R
ERS-L
ERS-R
NORM</td><td>FRS-L
FRS-R
ERS-L
ERS-R
NORM</td></tr>
</table>

Notes:

Investigator Signature: **Date:**

DAY-MONTH-YEAR

Place participant ID label here

Participant ID # ___________________

Visit Date _____/_____/______

VISIT 7: CHECKLIST

To be completed by study coordinator

13. Did the participant attend this visit? ☐ Yes (if yes, continue) ☐ No
14. Please check all assessments completed at this visit:

Visit Name: Intervention Checklist

☐ Prior and Concomitant Medications

☐ Change in Medications

☐ Health Status Reassessment

☐ Contraindications for Intervention

☐ NPR – Numeric Pain Rating

☐ Intervention Administration Log

15. Is the participant continuing in the study?

☐ No (If no, remember to complete a STUDY COMPLETION form.)

☐ Yes (If yes, schedule next visit.)

Comments:

____

Investigator Signature: **Date:**

DAY-MONTH-YEAR

Visit 8

Investigator Signature: **Date:**

DAY-MONTH-YEAR

THE RELIEF STUDY
RESEARCHING THE EFFECTIVENESS OF LUMBAR INTERVENTIONS FOR ENHANCING FUNCTION
Protocol NIH R01AT006978-01

Place participant ID label here

Participant ID # ________________

Visit Date _____/_____/______

Visit 8: PRIOR AND CONCOMITANT MEDICATIONS

To be completed by study coordinator

Have you taken any medications (including over the counter pain relief medications) or used heat or ice for your back pain since your last visit?

☐ No ☐ Yes (If so, record below.)

Number Days since last Visit: ________

Medication/Modality	Frequency and Dose	Indication	Start Date	Stop Date	Ongoing
1.					☐
2.					☐
3.					☐
4.					☐
5.					☐

Investigator Signature: **Date:**

DAY-MONTH-YEAR

THE RELIEF STUDY
RESEARCHING THE EFFECTIVENESS OF LUMBAR INTERVENTIONS FOR ENHANCING FUNCTION
Protocol NIH R01AT006978-01

Place participant ID label here

Participant ID # ___________________

Visit Date _____/_____/______

Visit 8: CHANGE IN MEDICATION USE

To be completed by study coordinator

Have you had any changes in medication use NOT related to your back pain in the past 7 days?

☐ No ☐ Yes (If YES, record below.)

Medication	Indication*	Dose & Frequency	Start Date	Stop Date	Ongoing
1.					☐
2.					☐
3.					☐
4.					☐
5.					☐

*If Indication is related to a negative change in health status, alert study coordinator, complete Health Status Reassessment and enter in Adverse Event Log.

Notes:

Investigator Signature: **Date:**

DAY-MONTH-YEAR

THE RELIEF STUDY
RESEARCHING THE EFFECTIVENESS OF LUMBAR INTERVENTIONS FOR ENHANCING FUNCTION
Protocol NIH R01AT006978-01

Place participant ID label here

Participant ID # __________________

Visit Date _____/_____/______

Visit 8: HEALTH STATUS REASSESSMENT

To be completed by study staff

Since your last visit, have you had any change in your health?

☐ No ☐ Yes (If YES, record below, alert study coordinator, and complete Adverse Event log.)

**Change & Symptoms**	
**Describe:**<br><br>**Start Date:**<br>**Stop Date:**<br>**Missed work or school?______days**	**Treatment:**<br><br>**Outcome:**

**Change & Symptoms**	
**Describe:**<br><br>**Start Date:**<br>**Stop Date:**<br>**Missed work or school?______days**	**Treatment:**<br><br>**Outcome:**

**Change & Symptoms**	
**Describe:**<br><br>**Start Date:**<br>**Stop Date:**<br>**Missed work or school?______days**	**Treatment:**<br><br>**Outcome:**

Investigator Signature: **Date:**

DAY-MONTH-YEAR

THE RELIEF STUDY
RESEARCHING THE EFFECTIVENESS OF LUMBAR INTERVENTIONS FOR ENHANCING FUNCTION
Protocol NIH R01AT006978-01

Place participant ID label here

Participant ID # ___________________

Visit Date _____/_____/______

Visit 8: CONTRAINDICATIONS FOR LAB TESTING

To be completed by study staff

Lab Testing Group		Since your last visit have you had any of the following?		If yes, describe and consult PI
☐	1 Muscular MRI	Contraindications for exposure to a magnetic field? (Must complete Magnetic Field Contraindications form)	☐ Yes ☐ No	
☐	2 Spinal MCL	Muscle weakness in the legs?	☐ Yes ☐ No	
		Episodes of incontinence of bowel or bladder?	☐ Yes ☐ No	
		New or previously unreported muscle, bone or joint injury?	☐ Yes ☐ No	Date:__________________
☐	3 Cortical TMS	Contraindications for exposure to a magnetic field?	☐ Yes ☐ No	
		Migraine headache 6-months prior to V2 physiologic assessments?	☐ Yes ☐ No	Administer ID Migraine Screener:<br>Sensitivity to light? ☐Yes ☐No<br>Nausea or vomiting?☐Yes ☐ No<br>Disabling intensity? ☐Yes ☐ No<br>*if at least 2 symptoms, cancel TMS testing<br>Date of last migraine:___________
		Taking medications in the benzodiazepine class?	☐ Yes ☐ No	
		Recent concussion?	☐ Yes ☐ No	

If YES, describe in right column, refer to PIs, and complete Adverse Event Log (forms located in Appendix).

Investigator Signature: **Date:**
DAY-MONTH-YEAR

Place participant ID label here

Participant ID # __________________

Visit Date _____/_____/______

Visit 8: CONTRAINDICATIONS FOR EXPOSURE TO A MAGNETIC FIELD

To be completed by study coordinator for MRI & TMS Lab Testing

Do you have any ***non-removable*** metal or foreign objects in your body such as Cardiac Pacemaker, Cardiac Valve Replacement, Brain Aneurysm Clip, Shunt, Aortic Clip, Surgical Clips, Implanted Neurotransmitter, Insulin Pump, Infusion device, or internal hearing aid such as Cochlear implant?

☐ Yes (if yes, indicate on Medical History – Summary, exclude from TMS & MRI lab testing)
☐ No

Do you have any **removable** metal or foreign objects in your body such as piercings, hearing aids, or prosthetic devices?

☐ Yes (alert lab) ☐ No

Do you have any joint replacements, metal rods, plates, screws, or nails in your body?

☐ Yes (alert lab and complete below) ☐ No

Surgery:
Date:

Surgery:
Date:

Surgery:
Date:

Do you have any foreign objects in your body such as shrapnel, bullet, an eye injury involving metal or do you work with metal occupationally?

☐ Yes (alert lab) ☐ No

Investigator Signature: **Date:**

DAY-MONTH-YEAR

THE RELIEF STUDY
RESEARCHING THE EFFECTIVENESS OF LUMBAR INTERVENTIONS FOR ENHANCING FUNCTION
Protocol NIH R01AT006978-01

Place participant ID label here

Participant ID # ____________________

Visit Date _____/_____/______

Visit 8: NUMERIC PAIN RATING SCALE

To be completed assessment team investigator

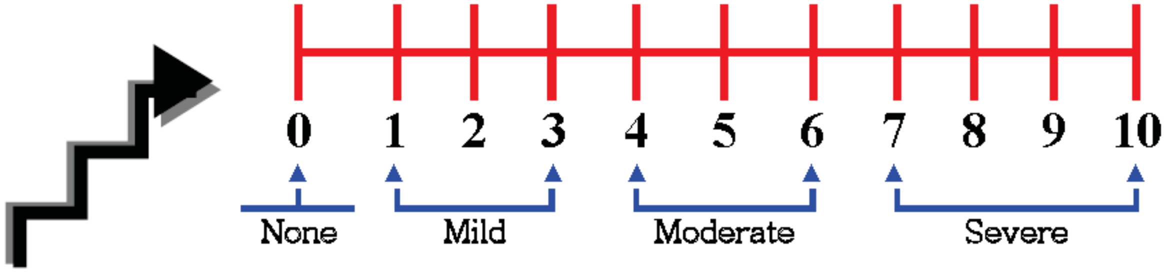

Instructions:
Sometimes it is helpful to think about the Numeric Rating Scale in the following manner:

- 0 = No Pain
- 1-3 = Mild Pain (nagging, annoying, interfering little with your activities of daily living)
- 4–6 = Moderate Pain (interferes significantly with your activities of daily living)
- 7-10 = Severe Pain (disabling; unable to perform your activities of daily living)

Please indicate:

- What number would you give your pain ***right now***? ☐
- What number would describe your pain ***since your last visit***? ☐

Since your last visit how would you answer the next 3 questions:

What number on a 0 to 10 scale would you give your pain when it is the worst that it gets? ☐

- What number on a 0 to 10 scale would you give your pain and when it is the best that it gets? ☐
- At what number is the pain at an acceptable level for you? ☐

Investigator Signature: **Date:**
DAY-MONTH-YEAR

THE RELIEF STUDY
RESEARCHING THE EFFECTIVENESS OF LUMBAR INTERVENTIONS FOR ENHANCING FUNCTION
Protocol NIH R01AT006978-01

Place participant ID label here

Participant ID # ___________________

Visit Date _____/_____/______

Visit 8: ROLAND-MORRIS DISABILITY QUESTIONNAIRE

To be completed by assessment team investigator

Directions: When your back hurts, you may find it difficult to do some of the things you normally do. Listed below are some sentences that others have used to describe themselves when they have back pain. When you read them, you may find that some stand out because they describe you *today*. As you read the list, think of yourself *today*. When you read a sentence that describes you today, circle YES. If the sentence does not describe you today, circle NO. Remember, only answer YES if you are sure the sentence describes you *today*.

1	I stay at home most of the time because of my back	YES	NO
2	I change positions frequently to try to get my back comfortable	YES	NO
3	I walk more slowly than usual because of my back	YES	NO
4	Because of my back, I am not doing any of the jobs I usually do around the house	YES	NO
5	Because of my back, I use a handrail to get upstairs	YES	NO
6	Because of my back, I lie down to rest more often	YES	NO
7	Because of my back, I have to hold on to something to get out of an easy chair	YES	NO
8	Because of my back, I try to get other people to do things for me	YES	NO
9	I get dressed more slowly than usual because of my back	YES	NO
10	I can only stand up for short periods of time because of my back	YES	NO
11	Because of my back, I try not to bend or kneel down	YES	NO
12	I find it difficult to get out of a chair because of my back	YES	NO
13	My back is painful almost all of the time	YES	NO
14	I find it difficult to turn over in bed because of my back	YES	NO
15	My appetite is not very good because of my back pain	YES	NO
16	I have trouble putting on my socks (or stockings) because of the pain in my back	YES	NO
17	I only walk short distances because of my back pain	YES	NO
18	I sleep less well because of my back	YES	NO
19	Because of my back pain, I get dressed with help from someone else	YES	NO
20	I sit down for most of the day because of my back	YES	NO
21	I avoid heavy jobs around the house because of my back	YES	NO
22	Because of my back pain, I am more irritable and bad tempered with people than usual	YES	NO
23	Because of my back, I go upstairs more slowly than usual	YES	NO
24	I stay in bed most of the time because of my back	YES	NO

Score: _________

Investigator Signature: **Date:**

DAY-MONTH-YEAR

THE RELIEF STUDY
RESEARCHING THE EFFECTIVENESS OF LUMBAR INTERVENTIONS FOR ENHANCING FUNCTION
Protocol NIH R01AT006978-01

Place participant ID label here

Participant ID # ___________________

Visit Date _____/_____/______

Visit 8: PROMIS – PAIN INTENSITY

To be completed by assessment team investigator

Instructions: Please respond to each item by marking one box per row.

In the past 7 days…

	Had no pain	Mild	Moderate	Severe	Very Severe
How intense was your pain at its worst?					
How intense was your average pain?					
What is your level of pain right now?					

Score: ________

Investigator Signature: **Date:**

DAY-MONTH-YEAR

Place participant ID label here

Participant ID # ___________________

Visit Date _____/_____/______

Visit 8: PROMIS – PAIN BEHAVIOR

To be completed by assessment team investigator

Instructions: Please respond to each item by marking one box per row.

In the past 7 days…

	**Had no pain**	**Never**	**Rarely**	**Sometimes**	**Often**	**Always**
When I was in pain I became irritable						
When I was in pain I grimaced						
When I was in pain I moved extremely slowly						
When I was in pain I moved stiffly						
When I was in pain I called out for someone to help me						
When I was in pain I isolated myself from others						
When I was in pain I thrashed						

Score: ________

Investigator Signature: **Date:**

DAY-MONTH-YEAR

THE RELIEF STUDY
RESEARCHING THE EFFECTIVENESS OF LUMBAR INTERVENTIONS FOR ENHANCING FUNCTION
Protocol NIH R01AT006978-01

Place participant ID label here

Participant ID # ___________________

Visit Date _____/_____/______

Visit 8: PROMIS – PAIN INTERFERENCE

To be completed by assessment team investigator

Instructions: Please respond to each item by marking one box per row.

In the past 7 days…

	Not at all	A little bit	Somewhat	Quite a bit	Very much
How much did pain interfere with your enjoyment of life?					
How much did pain interfere with your ability to concentrate?					
How much did pain interfere with your day to day activities?					
How much did pain interfere with your enjoyment of recreational activities?					
How much did pain interfere with doing your tasks away from home (e.g., getting groceries, running errands)?					

	Never	Rarely	Sometimes	Often	Always
How often did pain keep you from socializing with others?					

Score: ________

Investigator Signature: **Date:**

DAY-MONTH-YEAR

OHIO UNIVERSITY
OHIO UNIVERSITY OHIO MUSCULOSKELETAL AND NEUROLOGICAL INSTITUTE Heritage College of Osteopathic Medicine

Place participant ID label here

Participant ID # ___________________

Visit Date _____/_____/______

Visit 8: PROMIS – PHYSICAL FUNCTION

To be completed by assessment team investigator

Instructions: Please respond to each item by marking one box per row.

	**Not at all**	**Very little**	**Somewhat**	**Quite a bit**	**Cannot do**
Does your health now limit you in doing vigorous activities, such as running, lifting heavy objects, participating in strenuous sports?					
Does your health now limit you in walking more than a mile?					
Does your health now limit you in climbing one flight of stairs?					
Does your health now limit you in lifting or carrying groceries?					
Does your health now limit you in bending, kneeling, or stooping?					

	**Without any difficulty**	**With a little difficulty**	**With some difficulty**	**With much difficulty**	**Unable to do**
Are you able to do chores such as vacuuming or yard work?					
Are you able to dress yourself, including tying shoelaces and doing buttons?					
Are you able to shampoo your hair?					
Are you able to wash and dry your body?					
Are you able to get on and off the toilet?					

Score: ________

Investigator Signature: **Date:**

DAY-MONTH-YEAR

THE RELIEF STUDY
RESEARCHING THE EFFECTIVENESS OF LUMBAR INTERVENTIONS FOR ENHANCING FUNCTION
Protocol NIH R01AT006978-01

Place participant ID label here

Participant ID # ___________________

Visit Date _____/_____/______

Visit 8: CHECKLIST - PHYSIOLOGICAL OUTCOMES

To be completed by assessment team investigator

Experiment 1. Muscular Effects

- ☐ Ensure study participant is safe to enter a magnetic field.
- ☐ Have study participant lie supine for 30-minutes.
- ☐ Obtain and review scout images.
- ☐ Perform The RELIEF Study imaging protocol.
- ☐ Review images and ensure images are high quality.
- ☐ If necessary, repeat the imaging protocol and delete prior file.
- ☐ Save files to MRI terminal hard drive and back-up to cloud.

Experiment 2. Spinal Effects

- ☐ Instrument study participant for EMG.
- ☐ Instrument study participant for motion capture.
- ☐ Perform The RELIEF Study short latency spinal reflex protocol
- ☐ Perform The RELIEF Study standardized reaching protocol.
- ☐ Perform The RELIEF Study trunk perturbation protocol.
- ☐ If necessary repeat a trial and delete prior file.
- ☐ Save files to lab secure network hard drive and back-up to cloud.

Experiment 3. Cortical Effects

- ☐ Instrument study participant for EMG.
- ☐ Perform The RELIEF Study short latency spinal reflex protocol
- ☐ Map and mark the vertex of the skull.
- ☐ Find motor threshold.
- ☐ Perform The RELIEF Study paired pulse TMS protocol.
- ☐ Review signals throughout and ensure they are high quality.
- ☐ If necessary repeat a trial and delete prior file.
- ☐ Save files to lab terminal hard drive and back-up to cloud.

Did the participant complete the experiment?

☐ No

☐ Yes

Comments:

Investigator Signature: **Date:**

DAY-MONTH-YEAR

THE RELIEF STUDY
RESEARCHING THE EFFECTIVENESS OF LUMBAR INTERVENTIONS FOR ENHANCING FUNCTION
Protocol NIH R01AT006978-01

Place participant ID label here

Participant ID # __________________

Visit Date _____/_____/______

VISIT 8: CHECKLIST

To be completed by study coordinator

1. Did the participant attend this visit? ☐ Yes (if yes, continue) ☐ No

2. Please check all assessments completed at this visit:
   - ☐ Prior and Concomitant Medications
   - ☐ Change in Medications
   - ☐ Health Status Reassessment
   - ☐ Contraindications for Lab Testing
   - ☐ Contraindications for Exposure to a Magnetic Field (if applicable)
   - ☐ NPR – Numeric Pain Rating
   - ☐ RMDQ - Roland Morris Disability Questionnaire
   - ☐ PROMIS – Pain Intensity
   - ☐ PROMIS – Pain Behavior
   - ☐ PROMIS – Pain Interference
   - ☐ PROMIS – Physical Function
   - ☐ Physiological Outcomes

3. Is the participant continuing in the study?

   ☐ No (If no, remember to complete a STUDY COMPLETION form.)

   ☐ Yes (If yes, schedule next visit.)

Comments:

____

Investigator Signature: **Date:**

DAY-MONTH-YEAR

Visit 9

THE RELIEF STUDY
RESEARCHING THE EFFECTIVENESS OF LUMBAR INTERVENTIONS FOR ENHANCING FUNCTION
Protocol NIH R01AT006978-01

Place participant ID label here

Participant ID # ___________________

Visit Date _____/_____/______

Visit 9: PRIOR AND CONCOMITANT MEDICATIONS

To be completed by study coordinator

Have you taken any medications (including over the counter pain relief medications) or used heat or ice for your back pain in the past 7 days?

☐ No ☐ Yes (If YES, record below.)

Medication/Modality	Frequency and Dose	Indication	Start Date	Stop Date	Ongoing
1.					☐
2.					☐
3.					☐
4.					☐
5.					☐

Investigator Signature: **Date:**

DAY-MONTH-YEAR

THE RELIEF STUDY
RESEARCHING THE EFFECTIVENESS OF LUMBAR INTERVENTIONS FOR ENHANCING FUNCTION
Protocol NIH R01AT006978-01

Place participant ID label here

Participant ID # ________________

Visit Date _____/_____/______

Visit 9: CHANGE IN MEDICATION USE

To be completed by study coordinator

Have you had any changes in medication use NOT related to your back pain in the past 7 days?

☐ No ☐ Yes (If YES, record below.)

Medication	Indication*	Dose & Frequency	Start Date	Stop Date	Ongoing
1.					☐
2.					☐
3.					☐
4.					☐
5.					☐

*If Indication is related to a negative change in health status, alert study coordinator, complete Health Status Reassessment and enter in Adverse Event Log.

Notes:

Investigator Signature: **Date:**

DAY-MONTH-YEAR

Place participant ID label here

Participant ID # ___________________

Visit Date _____/_____/______

Visit 9: HEALTH STATUS REASSESSMENT

To be completed by study staff

Since your last visit, have you had any change in your health?

☐ No ☐ Yes (If YES, record below, alert study coordinator, and complete Adverse Event log.)

**Change & Symptoms**	
**Describe:**<br><br>**Start Date:**<br>**Stop Date:**<br>**Missed work or school?______days**	**Treatment:**<br><br>**Outcome:**

**Change & Symptoms**	
**Describe:**<br><br>**Start Date:**<br>**Stop Date:**<br>**Missed work or school?______days**	**Treatment:**<br><br>**Outcome:**

**Change & Symptoms**	
**Describe:**<br><br>**Start Date:**<br>**Stop Date:**<br>**Missed work or school?______days**	**Treatment:**<br><br>**Outcome:**

Investigator Signature: **Date:**

DAY-MONTH-YEAR

THE RELIEF STUDY
RESEARCHING THE EFFECTIVENESS OF LUMBAR INTERVENTIONS FOR ENHANCING FUNCTION
Protocol NIH R01AT006978-01

Place participant ID label here

Participant ID # ___________________

Visit Date _____/_____/______

Visit 9: CONTRAINDICATIONS FOR LAB TESTING

To be completed by study staff

Lab Testing Group		Since your last visit have you had any of the following?		If yes, describe and consult PI
☐	1 Muscular MRI	Contraindications for exposure to a magnetic field? (Must complete Magnetic Field Contraindications form)	☐ Yes ☐ No	
☐	2 Spinal MCL	Muscle weakness in the legs?	☐ Yes ☐ No	
		Episodes of incontinence of bowel or bladder?	☐ Yes ☐ No	
		New or previously unreported muscle, bone or joint injury?	☐ Yes ☐ No	Date:__________________
☐	3 Cortical TMS	Contraindications for exposure to a magnetic field?	☐ Yes ☐ No	
		Migraine headache 6-months prior to V2 physiologic assessments?	☐ Yes ☐ No	Administer ID Migraine Screener:<br>Sensitivity to light? ☐Yes ☐No<br>Nausea or vomiting?☐Yes ☐ No<br>Disabling intensity? ☐Yes ☐ No<br>*if at least 2 symptoms, cancel TMS testing<br>Date of last migraine:___________
		Taking medications in the benzodiazepine class?	☐ Yes ☐ No	
		Recent concussion?	☐ Yes ☐ No	

If YES, describe in right column, refer to PIs, and complete Adverse Event Log (forms located in Appendix).

Investigator Signature: **Date:**
DAY-MONTH-YEAR

Place participant ID label here

Participant ID # ________________

Visit Date _____/_____/______

Visit 9: CONTRAINDICATIONS FOR EXPOSURE TO A MAGNETIC FIELD

To be completed by study coordinator for MRI & TMS Lab Testing

Do you have any ***non-removable*** metal or foreign objects in your body such as Cardiac Pacemaker, Cardiac Valve Replacement, Brain Aneurysm Clip, Shunt, Aortic Clip, Surgical Clips, Implanted Neurotransmitter, Insulin Pump, Infusion device, or internal hearing aid such as Cochlear implant?

☐ Yes (if yes, indicate on Medical History – Summary, exclude from TMS & MRI lab testing and refer to screening clinician)
☐ No

Do you have any **removable** metal or foreign objects in your body such as piercings, hearing aids, or prosthetic devices?
☐ Yes (alert lab) ☐ No

Do you have any joint replacements, metal rods, plates, screws, or nails in your body?
☐ Yes (alert lab and complete below) ☐ No

Surgery:
Date:

Surgery:
Date:

Surgery:
Date:

Do you have any foreign objects in your body such as shrapnel, bullet, an eye injury involving metal or do you work with metal occupationally?
☐ Yes (alert lab) ☐ No

Investigator Signature: **Date:**
DAY-MONTH-YEAR

THE RELIEF STUDY
RESEARCHING THE EFFECTIVENESS OF LUMBAR INTERVENTIONS FOR ENHANCING FUNCTION
Protocol NIH R01AT006978-01

Place participant ID label here

Participant ID # ___________________

Visit Date _____/_____/______

Visit 9: NUMERIC PAIN RATING SCALE PRE-INTERVENTION

To be completed by assessment team investigator

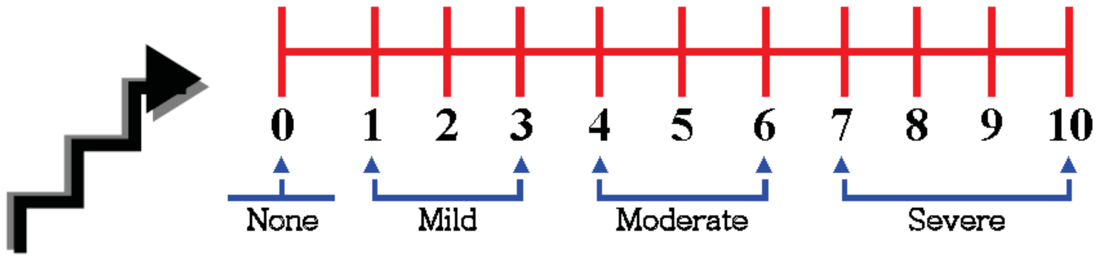

Instructions:
Sometimes it is helpful to think about the Numeric Rating Scale in the following manner:

- 0 = No Pain
- 1-3 = Mild Pain (nagging, annoying, interfering little with your activities of daily living)
- 4–6 = Moderate Pain (interferes significantly with your activities of daily living)
- 7-10 = Severe Pain (disabling; unable to perform your activities of daily living)

Please indicate:

- What number would you give your pain ***right now***? ☐
- What number would describe your pain ***over the last 7 days***? ☐

Since the onset of your low back pain how would you answer the next 3 questions:

- What number on a 0 to 10 scale would you give your pain when it is the worst that it gets? ☐
- What number on a 0 to 10 scale would you give your pain and when it is the best that it gets? ☐
- At what number is the pain at an acceptable level for you? ☐

Investigator Signature: **Date:**

DAY-MONTH-YEAR

THE RELIEF STUDY
RESEARCHING THE EFFECTIVENESS OF LUMBAR INTERVENTIONS FOR ENHANCING FUNCTION
Protocol NIH R01AT006978-01

Place participant ID label here

Participant ID # ___________________

Visit Date _____/_____/______

Visit 9: ROLAND-MORRIS DISABILITY QUESTIONNAIRE

To be completed by assessment team investigator

Directions: When your back hurts, you may find it difficult to do some of the things you normally do. Listed below are some sentences that others have used to describe themselves when they have back pain. When you read them, you may find that some stand out because they describe you *today*. As you read the list, think of yourself *today*. When you read a sentence that describes you today, circle YES. If the sentence does not describe you today, circle NO. Remember, only answer YES if you are sure the sentence describes you *today*.

1	I stay at home most of the time because of my back	YES	NO
2	I change positions frequently to try to get my back comfortable	YES	NO
3	I walk more slowly than usual because of my back	YES	NO
4	Because of my back, I am not doing any of the jobs I usually do around the house	YES	NO
5	Because of my back, I use a handrail to get upstairs	YES	NO
6	Because of my back, I lie down to rest more often	YES	NO
7	Because of my back, I have to hold on to something to get out of an easy chair	YES	NO
8	Because of my back, I try to get other people to do things for me	YES	NO
9	I get dressed more slowly than usual because of my back	YES	NO
10	I can only stand up for short periods of time because of my back	YES	NO
11	Because of my back, I try not to bend or kneel down	YES	NO
12	I find it difficult to get out of a chair because of my back	YES	NO
13	My back is painful almost all of the time	YES	NO
14	I find it difficult to turn over in bed because of my back	YES	NO
15	My appetite is not very good because of my back pain	YES	NO
16	I have trouble putting on my socks (or stockings) because of the pain in my back	YES	NO
17	I only walk short distances because of my back pain	YES	NO
18	I sleep less well because of my back	YES	NO
19	Because of my back pain, I get dressed with help from someone else	YES	NO
20	I sit down for most of the day because of my back	YES	NO
21	I avoid heavy jobs around the house because of my back	YES	NO
22	Because of my back pain, I am more irritable and bad tempered with people than usual	YES	NO
23	Because of my back, I go upstairs more slowly than usual	YES	NO
24	I stay in bed most of the time because of my back	YES	NO

Score: ________

Investigator Signature: **Date:**

DAY-MONTH-YEAR

THE RELIEF STUDY
RESEARCHING THE EFFECTIVENESS OF LUMBAR INTERVENTIONS FOR ENHANCING FUNCTION
Protocol NIH R01AT006978-01

Place participant ID label here

Participant ID # ___________________

Visit Date _____/_____/______

Visit 9: PROMIS – PAIN INTENSITY

To be completed by assessment team investigator

Instructions: Please respond to each item by marking one box per row.

In the past 7 days…

	Had no pain	Mild	Moderate	Severe	Very Severe
How intense was your pain at its worst?					
How intense was your average pain?					
What is your level of pain right now?					

Score: ________

Investigator Signature: **Date:**
DAY-MONTH-YEAR

THE RELIEF STUDY
RESEARCHING THE EFFECTIVENESS OF LUMBAR INTERVENTIONS FOR ENHANCING FUNCTION
Protocol NIH R01AT006978-01

Place participant ID label here

Participant ID # ___________________

Visit Date _____/_____/______

Visit 9: PROMIS – PAIN BEHAVIOR

To be completed by assessment team investigator

Instructions: Please respond to each item by marking one box per row.

In the past 7 days…

	**Had no pain**	**Never**	**Rarely**	**Sometimes**	**Often**	**Always**
When I was in pain I became irritable						
When I was in pain I grimaced						
When I was in pain I moved extremely slowly						
What I was in pain I moved stiffly						
When I was in pain I called out for someone to help me						
When I was in pain I isolated myself from others						
When I was in pain I thrashed						

Score: ________

Investigator Signature: **Date:**

DAY-MONTH-YEAR

Place participant ID label here

Participant ID # ________________

Visit Date _____/_____/______

Visit 9: PROMIS – PAIN INTERFERENCE

To be completed by assessment team investigator

Instructions: Please respond to each item by marking one box per row.

In the past 7 days…

	Not at all	A little bit	Somewhat	Quite a bit	Very much
How much did pain interfere with your enjoyment of life?					
How much did pain interfere with your ability to concentrate?					
How much did pain interfere with your day to day activities?					
How much did pain interfere with your enjoyment of recreational activities?					
How much did pain interfere with doing your tasks away from home (e.g., getting groceries, running errands)?					

	Never	Rarely	Sometimes	Often	Always
How often did pain keep you from socializing with others?					

Score: ________

Investigator Signature: **Date:**

DAY-MONTH-YEAR

THE RELIEF STUDY
RESEARCHING THE EFFECTIVENESS OF LUMBAR INTERVENTIONS FOR ENHANCING FUNCTION
Protocol NIH R01AT006978-01

Place participant ID label here

Participant ID # ___________________

Visit Date _____/_____/______

Visit 9: PROMIS – PHYSICAL FUNCTION

To be completed by assessment team investigator

Instructions: Please respond to each item by marking one box per row.

	**Not at all**	**Very little**	**Somewhat**	**Quite a bit**	**Cannot do**
Does your health now limit you in doing vigorous activities, such as running, lifting heavy objects, participating in strenuous sports?					
Does your health now limit you in walking more than a mile?					
Does your health now limit you in climbing one flight of stairs?					
Does your health now limit you in lifting or carrying groceries?					
Does your health now limit you in bending, kneeling, or stooping?					

	**Without any difficulty**	**With a little difficulty**	**With some difficulty**	**With much difficulty**	**Unable to do**
Are you able to do chores such as vacuuming or yard work?					
Are you able to dress yourself, including tying shoelaces and doing buttons?					
Are you able to shampoo your hair?					
Are you able to wash and dry your body?					
Are you able to get on and off the toilet?					

Score: ________

Investigator Signature: **Date:**
DAY-MONTH-YEAR

OHIO UNIVERSITY
OHIO UNIVERSITY OHIO MUSCULOSKELETAL AND NEUROLOGICAL INSTITUTE Heritage College of Osteopathic Medicine

THE RELIEF STUDY
RESEARCHING THE EFFECTIVENESS OF LUMBAR INTERVENTIONS FOR ENHANCING FUNCTION
Protocol NIH R01AT006978-01

Place participant ID label here

Participant ID # ________________

Visit Date _____/_____/______

Visit 9: CHECKLIST - PHYSIOLOGICAL OUTCOMES

To be completed by assessment team investigator

Experiment 1. Muscular Effects

- ☐ Ensure study participant is safe to enter a magnetic field.
- ☐ Have study participant lie supine for 30-minutes.
- ☐ Obtain and review scout images.
- ☐ Perform The RELIEF Study imaging protocol.
- ☐ Review images and ensure images are high quality.
- ☐ If necessary, repeat the imaging protocol and delete prior file.
- ☐ Save files to MRI terminal hard drive and back-up to cloud.

Experiment 2. Spinal Effects

- ☐ Instrument study participant for EMG.
- ☐ Instrument study participant for motion capture.
- ☐ Perform The RELIEF Study short latency spinal reflex protocol
- ☐ Perform The RELIEF Study standardized reaching protocol.
- ☐ Perform The RELIEF Study trunk perturbation protocol.
- ☐ If necessary repeat a trial and delete prior file.
- ☐ Save files to lab secure network hard drive and back-up to cloud.

Experiment 3. Cortical Effects

- ☐ Instrument study participant for EMG.
- ☐ Perform The RELIEF Study short latency spinal reflex protocol
- ☐ Map and mark the vertex of the skull.
- ☐ Find motor threshold.
- ☐ Perform The RELIEF Study paired pulse TMS protocol.
- ☐ Review signals throughout and ensure they are high quality.
- ☐ If necessary repeat a trial and delete prior file.
- ☐ Save files to lab terminal hard drive and back-up to cloud.

Did the participant complete the experiment?

☐ No

☐ Yes

Comments:

Investigator Signature: **Date:**

DAY-MONTH-YEAR

Place participant ID label here

Participant ID # ________________

Visit Date _____/_____/______

VISIT 9: CHECKLIST

To be completed by study coordinator

1. Did the participant attend this visit? ☐ Yes (if yes, continue) ☐ No

2. Please check all assessments completed at this visit:

☐ NPR – Numeric Pain Rating
☐ RMDQ - Roland Morris Disability Questionnaire
☐ PROMIS – Pain Intensity
☐ PROMIS – Pain Behavior
☐ PROMIS – Pain Interference
☐ PROMIS – Physical Function
☐ Prior and Concomitant Medications
☐ Change in Medication Use
☐ Health Status Reassessment
☐ Contraindications for Lab Testing
☐ Contraindications for Exposure to a Magnetic Field (if applicable)
☐ Physiological Outcomes (Experiment 1, 2, or 3)

3. Is the participant continuing in the study?

☐ No (If no, complete a STUDY COMPLETION form.)
☐ Yes (If yes, schedule next visit.)

Comments:

____

Investigator Signature: **Date:**
DAY-MONTH-YEAR

APPENDIX

THE RELIEF STUDY
RESEARCHING THE EFFECTIVENESS OF LUMBAR INTERVENTIONS FOR ENHANCING FUNCTION
Protocol NIH R01AT006978-01

Place participant ID label here

Participant ID # ________________

Visit Date _____/_____/______

Appendix: STUDY COMPLETION

To be completed by study coordinator

1. Date of final study visit: _________ / ___________ / ___________
   Day Month Year

2. Participant notified regarding end of active protocol study participation?
   ☐ YES ☐ NO

3. Date of last-known study intervention: _________ / ___________ / ___________
   Day Month Year

4. Primary reason for terminating participation in the study:
   - ☐ Completed study
   - ☐ Not eligible*
   - ☐ Participant was determined after enrollment to be ineligible (provide comments) ______*
   - ☐ Participant withdrew consent
   - ☐ In the principal investigator's opinion, it was not in the participant's best interest to continue (provide comments) ________________________________
   - ☐ Adverse event (If checked, complete the AE form)
   - ☐ Death
   - ☐ Lost to follow-up
   - ☐ Other (specify): ________________________________
   - ☐ Unknown

*If participant determined to be ineligible due to CES-D score, give participant the DEBRIEFING – DEPRESSION letter.

Comments:

Investigator Signature: **Date:**

DAY-MONTH-YEAR

Place participant ID label here

Participant ID # ________________

Visit Date _____/_____/______

ID Migraine Screener

To be completed by participant if migraine headache reported

During the last 3 months, did you have any of the following with your headaches?*

1. You felt nauseated or sick to your stomach when you had a headache?

   ☐ Yes ☐ No

2. Light bothered you (a lot more than when you don't have headaches)?

   ☐ Yes ☐ No

3. Your headaches limited your ability to work, study, or do what you needed to do for at least 1 day?

   ☐ Yes ☐ No

Investigator Signature: **Date:**

DAY-MONTH-YEAR

THE RELIEF STUDY
RESEARCHING THE EFFECTIVENESS OF LUMBAR INTERVENTIONS FOR ENHANCING FUNCTION
Protocol NIH R01AT006978-01

Place participant ID label here

Participant ID # ___________________

Visit Date _____/_____/______

PHYSICAL EXAM - HEALTH STATUS REASSESSMENT

To be completed by clinician if indicated in Health Status Reassessment

This participant has self-reported a change in his or her health status and needs to be reassessed for adverse events and ability to participate in the study. Refer to HEALTH STATUS REASSESSMENT form for details and complete the form below based on findings.

Category	Normal or Abnormal	If abnormal, describe below
General Appearance	☐ Normal<br>☐ Abnormal<br>☐ Not Examined	
Musculoskeletal	☐ Normal<br>☐ Abnormal<br>☐ Not Examined	
Extremities/ Skin	☐ Normal<br>☐ Abnormal<br>☐ Not Examined	
Neurological (dermatomes, myotomes, reflexes)	☐ Normal<br>☐ Abnormal<br>☐ Not Examined	
Other, specify: _____	☐ Normal<br>☐ Abnormal<br>☐ Not Examined	

I certify this patient is:

- ☐ Able to continue study
- ☐ Temporarily unable to continue in the study at this time due to a self-limited medical condition
  - o Reassess health status in ____________ days / weeks. (complete Protocol Deviation Form and Adverse Event Form)
- ☐ Unable to continue in the study due to a serious change in medical status (complete Adverse Event Form, Protocol Deviation Form and Study Completion Form)

☐ I have recommended the participant seek medical care from his or her personal physician.

Investigator Signature: **Date:**

DAY-MONTH-YEAR

THE RELIEF STUDY
RESEARCHING THE EFFECTIVENESS OF LUMBAR INTERVENTIONS FOR ENHANCING FUNCTION
Protocol NIH R01AT006978-01

Place participant ID label here

Participant ID # ___________________

Visit Date _____/_____/______

Appendix: SERIOUS ADVERSE EVENT

To be completed by study coordinator

1. SAE onset date: ______/_______/________
   Day Month Year

2. SAE stop date: _____/_________/________
   Day Month Year

3. Location of serious adverse event: ____

4. Was this an unexpected adverse event? ☐ Yes ☐ No

5. Brief description of participants with no personal identifiers:
   Sex: ☐ F ☐ M Age: _____
   Diagnosis for study participation: ____

6. Brief description of the nature of the serious adverse event (attach description if more space is needed):
   ____

7. Category of the serious adverse event:
   - ☐ Death: Date of death ____________ (dd/mm/yyyy)
   - ☐ Life threatening event
   - ☐ Hospitalization – inpatient or prolonged
   - ☐ Disability/incapacity
   - ☐ Congenital anomaly/birth defect
   - ☐ Required intervention to prevent permanent impairment – important medical event based upon appropriate medical judgment
   - ☐ Other: ____________________________

8. Intervention type:
   - ☐ Medication or nutritional supplement (specify): ______________________________
   - ☐ Device (specify): ______________________________
   - ☐ Surgery (specify): ______________________________
   - ☐ Behavioral/lifestyle (specify): ______________________________

Investigator Signature: **Date:**
DAY-MONTH-YEAR

THE RELIEF STUDY
RESEARCHING THE EFFECTIVENESS OF LUMBAR INTERVENTIONS FOR ENHANCING FUNCTION
Protocol NIH R01AT006978-01

Place participant ID label here

Participant ID # ___________________

Visit Date _____/_____/______

Appendix: ADVERSE EVENTS

To be completed by study coordinator

Has the participant had any adverse events (AE) during this study?

☐ Yes *(if yes, please list all adverse events below.)*

☐ No

**Adverse Event** (Refer to CTCAE Grading and Attribution Scales)	**Start Date**	**Stop Date**	**Severity Grade***	**Attribution****	**Action Taken**	**Outcome of AE**	**Expected**	**Serious Adverse Event**	**Reportable to IRB? Y/N**	**Initials**
**1.**										
**2.**										
**3.**										
**4.**										
**5.**										
**6.**										
**7.**										
**8.**										
**9.**										
**10.**										
**11.**										
**12.**										

Investigator Signature: **Date:**

Appendix: ADVERSE EVENTS - ATTRIBUTES

**ATTRIBUTION

Severity Grade*	Attribution**	Action Taken	Outcome of AE	Expected	Serious Adverse Event
1 Mild<br>2 Moderate<br>3 Severe<br>4 Life-threatening<br>5 Death	1 Clearly Unrelated<br>2 Doubtfully Related<br>3 May be Related<br>4 Likely Related<br>5 Clearly Related	1 None<br>2 Discontinued permanently<br>3 Discontinued temporarily<br>4 Reduced dose<br>5 Increased dose<br>6 Delayed dose	1 Resolved, no sequela<br>2 AE still present, no treatment<br>3 AE still present, being treated<br>4 Residual effects present, not treated<br>5 Residual effects present, treated<br>6 Death<br>7 Unknown	1 Yes<br><br>2 No	1 Yes (If YES, complete SAE form)<br><br>2 No

RELATIONSHIP	ATTRIBUTION	DESCRIPTION
Unrelated to investigational agent/intervention[1]	Unrelated	The AE *is clearly **NOT** related* to the intervention
	Unlikely	The AE *is **doubtfully related*** to the intervention
Related to investigational agent/intervention[1]	Possible	The AE ***may be related*** to the intervention
	Probable	The AE ***is likely related*** to the intervention
	Definite	The AE ***is clearly related*** to the intervention

Grade refers to the severity of the AE. The CTCAE displays Grades 1 through 5 with unique clinical descriptions of severity for each AE based on this general guideline:

Grade 1	Mild; asymptomatic or mild symptoms; clinical or diagnostic observations only; intervention not indicated.
Grade 2	Moderate; minimal, local or noninvasive intervention indicated; limiting age-appropriate instrumental ADL*.
Grade 3	Severe or medically significant but not immediately life-threatening; hospitalization or prolongation of hospitalization indicated; disabling; limiting self care ADL**.
Grade 4	Life-threatening consequences; urgent intervention indicated.
Grade 5	Death related to AE.

A Semi-colon indicates 'or' within the description of the grade.

A single dash (-) indicates a grade is not available.

Investigator Signature: **Date:**

Place participant ID label here

Participant ID # ___________________

Visit Date _____/_____/______

Investigator Signature: **Date:**

THE RELIEF STUDY
RESEARCHING THE EFFECTIVENESS OF LUMBAR INTERVENTIONS FOR ENHANCING FUNCTION
Protocol NIH R01AT006978-01

Place participant ID label here

Participant ID # ___________________

Visit Date _____/_____/______

PROTOCOL DEVIATION FORM

Did this participant have any protocol deviations? ☐ Yes ☐ No

Description of Protocol Deviation	Deviation Category*	Deviation Code**	Date Deviation Occurred	Date IRB Notified (if applicable)	Principal Investigator's Signature	Date Signed

Investigator Signature: **Date:**

THE RELIEF STUDY
RESEARCHING THE EFFECTIVENESS OF LUMBAR INTERVENTIONS FOR ENHANCING FUNCTION
Protocol NIH R01AT006978-01

PROTOCOL DEVIATION CATEGORY AND CODE

*DEVIATION CATEGORY

A. Safety	B. Informed Consent	C. Eligibility	D. Protocol Implementation	E. Other, specify in log

**DEVIATION CODES: Numbers listed by the sample protocol deviations

**Safety (Category A)**<br>1. Not reporting an SAE within 24 hours<br>2. Laboratory tests not done<br>3. AE/SAE is not reported to IRB<br>4. Other, specify in log	**Eligibility (Category C)**<br>1. Participant did not meet eligibility criterion<br>2. Randomization of an ineligible participant<br>3. Participant randomized prior to completing baseline assessment, etc.<br>4. Randomization and/or treatment of participant prior to IRB approval of protocol<br>5. Other, specify in log
**Informed consent (Category B)**<br>6. Failure to obtain informed consent<br>7. Consent form used was not current IRB-approved version<br>8. Consent form does not include updates or information required by IRB<br>9. Consent form missing<br>10. Consent form not signed and dated by participant<br>11. Consent form does not contain all required signatures<br>12. Other, specify in log	**Protocol implementation (Category D)**<br>1. Failure to keep IRB approval up to date<br>2. Participant receives wrong treatment<br>3. Participant seen outside visit window – intervention & clinical outcomes<br>4. Participant seen outside visit window – physiological outcomes<br>5. Use of unallowable concomitant treatments<br>6. Prescribed dosing outside protocol guidelines<br>7. Missed assessment<br>8. Missed visit<br>9. Other, specify in log

Investigator Signature: **Date:**

THE RELIEF STUDY
RESEARCHING THE EFFECTIVENESS OF LUMBAR INTERVENTIONS FOR ENHANCING FUNCTION
Protocol NIH R01AT006978-01

DELEGATION OF AUTHORITY LOG

Name	Study Role	Study Delegations*	Study Involvement Start Date	Study Involvement Stop Date	Initials	Signature

We certify that the above individuals are appropriately trained, have read the protocol and pertinent sections of 21 Code of Federal Regulations Parts 50 and 56 (21 CFR Parts 50, 56) and the International Conference on Harmonisation and Good Clinical Practice (ICH GCP) Guidance, and are authorized to perform the above study-related tasks and procedures. Although we have delegated significant trial-related duties, as the principal investigators, we still maintain full responsibility for this trial.

***Study Delegations**
1 Obtain Informed Consent
2 Secure Document Completion
3 Complete Case Report Forms (CRFs)
4 Assess Inclusion and Exclusion Criteria
5 Physical Examination
6 Medical History
7 Medication History and Concomitant Medications
8 Assess Clinical & Physiological Outcomes
9 Adverse Event Inquiry and Reporting
10 Deliver Interventions
11 Maintain Regulatory Documents

Principal Investigator's Signature:

Name:________________________________Date: __________________

Name:________________________________Date: __________________

THE RELIEF STUDY
RESEARCHING THE EFFECTIVENESS OF LUMBAR INTERVENTIONS FOR ENHANCING FUNCTION
Protocol NIH R01AT006978-01

OHIO UNIVERSITY

DEBRIEFING – Change in health status

Dear participant,

We very much appreciate the time and effort it took for you to be part of The RELIEF Study. Our screening indicates you may have a medical problem, and as such we recommend that you follow-up with your physician regarding your health status. At this time we feel it is not safe or appropriate for you to continue participating in The RELIEF Study. If you or your physician have questions for us please feel free to contact The RELIEF Study Principal Investigators (James Thomas, P.T., Ph.D. at 740.593.4178 or Brian Clark, Ph.D. at 740.593.2354).

Regards,

James Thomas, P.T., Ph.D. Brian Clark, Ph.D

THE

RELIEF

STUDY

Researching the **E**ffectiveness of **L**umbar **I**nterventions for **E**nhancing **F**unction

Core Muscle Test

The RELIEF Study is seeking **healthy** men and women between the ages of 18-45 years old to participate in a research study investigating treatment for low back pain. Participation requires completing several surveys and attending a testing session. Financial compensation is provided for participating in this study. Please contact us at RELIEF@ohio.edu or 740.566.PAIN for more information.

NIH National Center for Complementary and Alternative Medicine (NCCAM)

OHIO UNIVERSITY | OHIO MUSCULOSKELETAL AND NEUROLOGICAL INSTITUTE
Heritage College of Osteopathic Medicine

Back Pain?

(Pain on most days for several months?)

Learn.

You can help researchers discover new ways to treat back pain!

Volunteer.

Several studies, that may involve:

- MRI
- Virtual Reality Gaming
- Motion Capture
- Physical Therapy
- Osteopathic Therapies
- Cold Laser Therapy

Participate.

- Time commitments range from a few days to several months, Financial compensation provided, possibly receive treatments

www.ohio.edu/backpain

MCL THE MOTOR CONTROL LAB OHIO UNIVERSITY

1804 OHIO UNIVERSITY

THE RELIEF STUDY
RESEARCHING THE EFFECTIVENESS OF LUMBAR INTERVENTIONS FOR ENHANCING FUNCTION
Protocol NIH R01AT006978-01

OHIO UNIVERSITY

DEBRIEFING – Depression

Dear participant,

Thank you so much for your interest in our research project.

As recommended by Ohio University Institutional Review Board, we have an obligation to inform you that your performance on a test during the survey process indicates that you may be clinically depressed. Please note that a simple survey test cannot definitely make a diagnosis of depression. Only your doctor can determine whether you are depressed and should need a treatment or not.

We highly recommend you do the following:

1). Visit your primary care doctor and discuss the possibility of being depressed as soon as possible.

2) If you do not have a doctor available you can visit one of two centers located in Athens which have clinical expertise in diagnosis and treatment of depression:

Ohio University Psychology and Social Work Clinic
(740) 593-0902

Hopewell Health Services
(740) 594-5045

Once again, we appreciate your participation in our research project. We wish you the best and with excellent health.

Regards,

James Thomas, P.T., Ph.D.　　　Brian Clark, Ph.D

A research team with Ohio University, Athens, OH, believes you might be good match for the following study:

Are you...?

- *currently experiencing low back pain*
- *between the ages of 18-45 years old*
- *able to attend several appointments at Ohio University in Athens, OH*
- *interested in 3-weeks of non-surgical treatment*

Research may be right for you. Please consider The RELIEF Study. We want to find out what works best to treat low back pain and how certain treatments for low back pain work. The RELIEF Study includes 9 visits lasting from 15-minutes to 3-hours over an 8-week period. Visits include completing surveys, attending several testing sessions, and receiving treatments. For your time, you will receive $400 disbursed across the appointments.

If you are interested in this study and having the research team contact you directly, please select the "Yes, I'm interested" link below. By clicking the "Yes, I'm interested" link, your contact information will be released to the research team. If you select the "No, thanks." link or do not respond to this study message, your contact information will not be released to the research team.

QUICK LINK OPTION: YES QUICK LINK OPTION: NO

Thank you for your interest in ResearchMatch.

THE RELIEF STUDY

Researching the **E**ffectiveness of **L**umbar **I**nterventions for **E**nhancing **F**unction

Back Pain?

The RELIEF Study is seeking men and women between the ages of 18-45 years old to participate in a research study investigating treatment for low back pain. Participation requires completing several testing sessions and attending several treatment sessions. Financial compensation is provided for participating in this study.

Funded by
NIH National Center for Complementary and Alternative Medicine (NCCAM)

Contact The RELIEF Study: 640.566.PAIN or www.ohio.edu/RELIEF

The RELIEF Study
640.566.PAIN or
www.ohio.edu/RELIEF

The RELIEF Study
640.566.PAIN or
www.ohio.edu/RELIEF

The RELIEF Study
640.566.PAIN or
www.ohio.edu/RELIEF

The RELIEF Study
640.566.PAIN or
www.ohio.edu/RELIEF

The RELIEF Study
640.566.PAIN or
www.ohio.edu/RELIEF

The RELIEF Study
640.566.PAIN or
www.ohio.edu/RELIEF

The RELIEF Study
640.566.PAIN or
www.ohio.edu/RELIEF

The RELIEF Study
640.566.PAIN or
www.ohio.edu/RELIEF

The RELIEF Study
640.566.PAIN or
www.ohio.edu/RELIEF

The RELIEF Study
640.566.PAIN or
www.ohio.edu/RELIEF

THE RELIEF STUDY
Researching the Effectiveness of Lumbar Interventions for Enhancing Function

Dr. James Thomas & Brian Clark
Ohio University
Irvine Hall 233
Athens, Ohio 45701

February 18, 2013

Dr. Name Name
Business
Address
Athens, Ohio 45701

Dear Dr. Name:

We are pleased to announce The RELIEF Study - a randomized, masked, placebo controlled, clinical trial designed to explore the effects of manual therapies used to treat low back pain. This research is funded by the National Center for Complementary and Alternative Medicine, a division of the National Institutes of Health. Despite the high prevalence of low back pain and the popularity of manual therapy treatments, relatively little formal research exists. The RELIEF Study is a multidisciplinary, collaborative research project designed to determine the biological mechanisms of commonly used manual therapies and to determine their effectiveness at reducing pain and disability in patients with chronic low back pain.

We are recruiting eligible patients with symptomatic low back pain between the ages of 18 to 45 years. Participants will be cared for by licensed and trained physical therapists and osteopathic physicians. Outcomes are measured through standardized surveys and questionnaires as well as through laboratory tests including transcranial magnetic stimulation, magnetic resonance imaging, and biomechanical instrumentation. There will be 3 weeks of active treatment for each subject and an additional 4 weeks of follow-up assessments. There will be no fee for any of the treatment visits and participants will be financially compensated.

We are trying to establish a select number of local doctors who will refer patients for this study. We invite you to consider referring potential candidates to us. This would be of great value in helping us recruit subjects for this study. It would also be a valuable service to your patients because they would be eligible to receive treatment for low back pain at no cost. Thank you for your consideration. If you have any questions, would like further information, or are interested in referring patients for The RELIEF Study, please call us at (740)59#-####.

Sincerely,

James Thomas Brian Clark

OHIO UNIVERSITY 1804

OHIO UNIVERSITY | OHIO MUSCULOSKELETAL AND NEUROLOGICAL INSTITUTE
Heritage College of Osteopathic Medicine

THE RELIEF STUDY

Researching the Effectiveness of Lumbar Interventions for Enhancing Function

Back Pain?

The RELIEF Study is seeking men and women between the ages of 18-45 years old to participate in a research study investigating treatment for low back pain. Participation requires completing several testing sessions and attending several treatment sessions. Financial compensation is provided for participating in this study. Please contact us at RELIEF@ohio.edu or 740.566.PAIN for more information.

NIH National Center for Complementary and Alternative Medicine (NCCAM)

OHIO UNIVERSITY

Heritage College of Osteopathic Medicine

Services

News

Events

Media Relations

Multimedia

Publications

Ohio University Medicine

Viewbook

Rounds

Design Services

Project Initiation Form

Research Posters

Exhibits/Displays

OU-HCOM Mark

Staff/Contacts

Policies

Communication Home

Ohio researchers receive NIH grant to study treatments for lower back pain

(ATHENS, Ohio – Oct. 1, 2012) Approximately 60 to 90 percent of U.S. residents will experience lower back pain over the course of their lives. Recent reports indicate that annual costs related to low back pain exceed $90 billion in medical expenses, missed work and lost productivity.

In a step towards finding more effective treatments for lower back pain, two Ohio University researchers, **James S. Thomas, P.T., Ph.D.**, and **Brian C. Clark, Ph.D.,** recently received a five year, $2.1 million grant from the National Institutes of Health (NIH) to fund "The RELIEF Study," which stands for Researching the Effectiveness of Lumbar Interventions for Enhancing Function Study. Thomas is a professor of physical therapy and director of research in the Division of Physical Therapy, in the School of Rehabilitation and Communication Sciences at the Ohio University College of Health Sciences and Professions (CHSP), and Clark is a professor of physiology in the Department of Biomedical Sciences and director of the Ohio Musculoskeletal and Neurological Institute (OMNI) at the Ohio University Heritage College of Osteopathic Medicine (OU-HCOM).

"The study will look at the mechanisms and effectiveness of three different non-surgical interventions used to treat chronic low back pain. It will determine both the physiological effects and clinical effectiveness of the treatments at reducing pain and disability," said Clark.

"The idea behind the study, in part, grew out of my earlier NIH-funded study that investigated the neuromuscular mechanisms underlying motor behavior in people with low-back pain. This builds on these previous results by examining several therapeutic interventions commonly used to treat low back pain and to look as some of the neurophysiological mechanisms that underlie these treatments. Understanding these basic mechanisms will help to enhance the decision-making process of when to use one intervention or another," said Thomas.

The RELIEF Study is OMNI's first foray into large, randomized clinical trials, said Clark. It will include 162 people with lower back pain randomly assigned to one of three of the treatment groups. To prevent bias in measuring the effects of these treatment interventions, the research scientists will not know the treatment any study participant is receiving. "This information will only be known by the treating clinicians and the research coordinator. This level of 'blinding' is a critical component in a randomized clinical trial and minimizes the potential for experimenter bias," Thomas said.

The researchers will use a number of techniques to assess the effects of the treatments on a variety of physiological outcomes. For instance, they will use transcranial magnetic stimulation to assess the excitability of the portion of the brain that controls the low back muscles. They will use magnetic resonance imaging to determine if the treatments are able to reduce muscle hyperactivity in the low back region, and finally, they will use sophisticated biomechanical instrumentation to examine how the low back muscles are activated, and how the spine moves, during various tasks. Each of the study participants will receive one of the three non-surgical interventions over a period of three weeks.

"This is a synergistic effort that has been building for several years," said Clark. The study not only builds on Thomas' previous study but also the work of researchers involved in OMNI's Low Back and Chronic Pain Disorders Research Program. Recent work performed in Clark's Neuromuscular Physiology Laboratory and in Thomas' Motor Control Laboratory has focused on pioneering and developing a number of techniques to measure physiological properties of the low back muscles. "We have published several articles recently on these techniques, and now [with this grant] have the ability to do extend this work into a robust clinical trial," Thomas said.

Importantly, This project also brings together a multi-disciplinary team of researchers from Ohio University, the Kessler Foundation Research Center in New Jersey and the University of Illinois at Chicago. In addition to Thomas and Clark, key personnel for The RELIEF Study include:

- **David Russ, P.T., Ph.D.,** assistant professor the Division of Physical Therapy in the School of Rehabilitation and Communication Sciences, a physical therapist with expertise in exercise and skeletal muscle physiology;
- **Christopher France Ph.D.,** professor in the Department of Psychology, a psychophysiologist with expertise in chronic pain and pain-related fear;
- **Masato Nakazawa Ph.D.,** a biostatistician in the OU-HCOM Office of Research and Grants;
- **Stevan Walkowski, D.O.,** assistant professor in the Department of Osteopathic Manipulative Medicine at OU-HCOM, a physician with expertise in the treatment of low back pain;
- **Daniel Corcos, Ph.D.,** professor in the Department of Kinesiology and Nutrition at the University of Illinois at Chicago, a neuroscientist with expertise in randomized clinical trials; and
- **Guang Yue, Ph.D.,** director of the Human Performance and Engineering Laboratory at the Kessler Foundation Research Center, a physiologist with expertise in neurophysiology and magnetic resonance imaging.

  In addition to these individuals, a number of osteopathic physicians and physical therapists will serve on The RELIEF Study Treatment Provider Team. These clinicians will supervise and provide the treatment interventions when the trial begins in the spring of 2013.

  "This grant will allow for collaboration between physicians in OU-HCOM and the physical therapists in CHSP who comprise our treatment team. This is particularly important because the interventions being studied are used extensively by both osteopathic physicians and physical therapists in the treatment of low back pain, " said Clark.

"This is a very substantial grant in a time when less than 10% of applications are being funded at the NIH. Through a very rigorous process of peer review we've been able to get a sophisticated study funded that should provide important answers and help direct future interventions in the treatment in low back pain,"

Thomas said. "Low back pain is a clinically significant problem and we are uniquely positioned to take a multidisciplinary approach to address this problem."

Office of Communication
Ohio University Heritage College of Osteopathic Medicine
210 Irvine Hall, Athens, Ohio 45701
Tel: 740-593-2346 FAX: 740-593-0343

Last updated: 11/30/2012

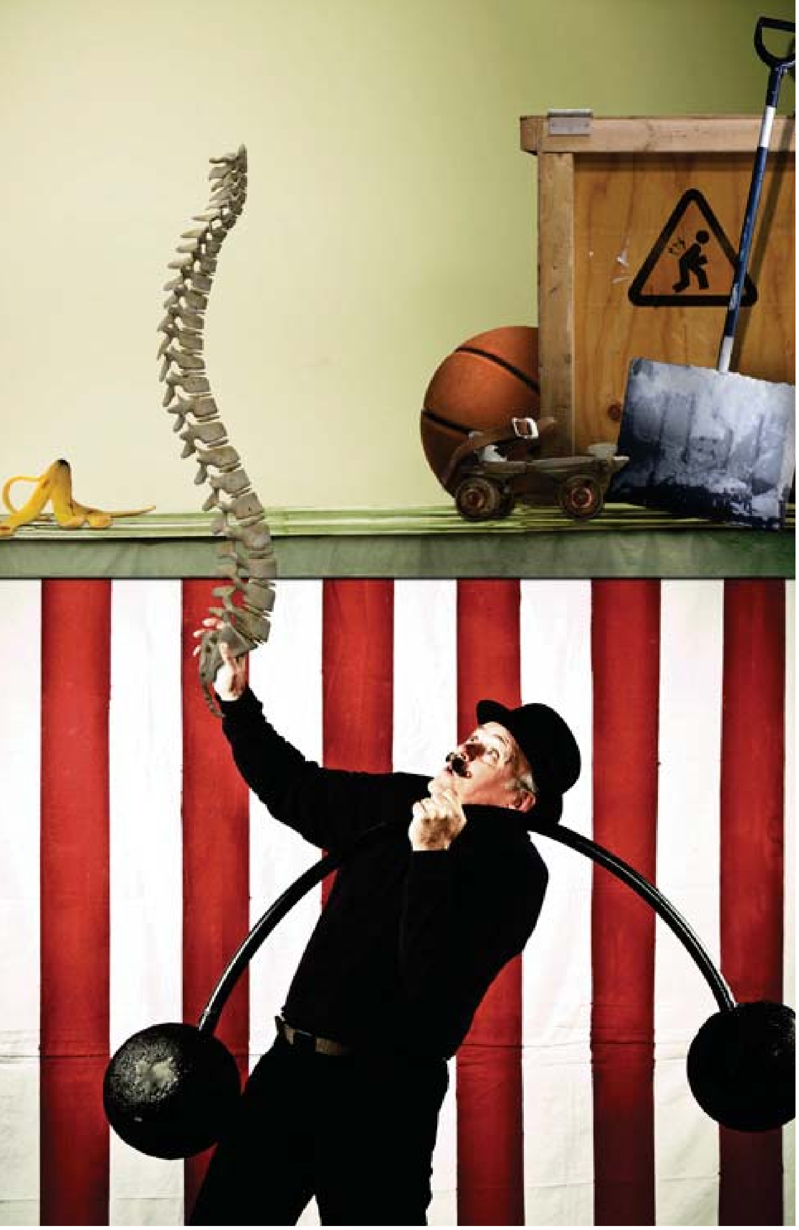

"Low back pain is a clinically significant problem and we are uniquely positioned to take a multidisciplinary approach to address this problem."

740.566.PAIN (7246)
www.ohio.edu/RELIEF
RELIEF@ohio.edu

A randomized clinical trial supported through a grant from the National Institutes of Health National Center for Complimentary and Alternative Medicine..

Low back pain is one of the most common reasons for seeking medical care and accounts for over 3.7 million physician visits/year in the U.S. alone. Ninety percent of adults will experience low back pain in their lifetime, 50% will experience recurrent LBP, and 10% will develop chronic pain and related disability. According to a recent national survey, more than 18 million Americans over the age of 18 years received manipulative therapies in 2007 at a total annual out of pocket cost of $3.9 billion.

While there is growing evidence for the clinical effectiveness of alternative and complementary therapies to treat low back pain, little is known on the neurophysiologic consequences and effects of these treatments. Further, additional data is needed to understand how these different treatment techniques effect clinical changes in pain and disability.

The lack of empirical data hinders acceptance by the wider scientific and health-care communities, and it also limits the development of rational strategies for using alternative and complementary therapies.

The RELIEF Study is seeking men and women between the ages of 18-45 years to participate in a research study investigating treatment for low back pain.

THE RELIEF STUDY

RESEARCHING THE EFFECTIVENESS OF LUMBAR INTERVENTIONS FOR ENHANCING FUNCTION

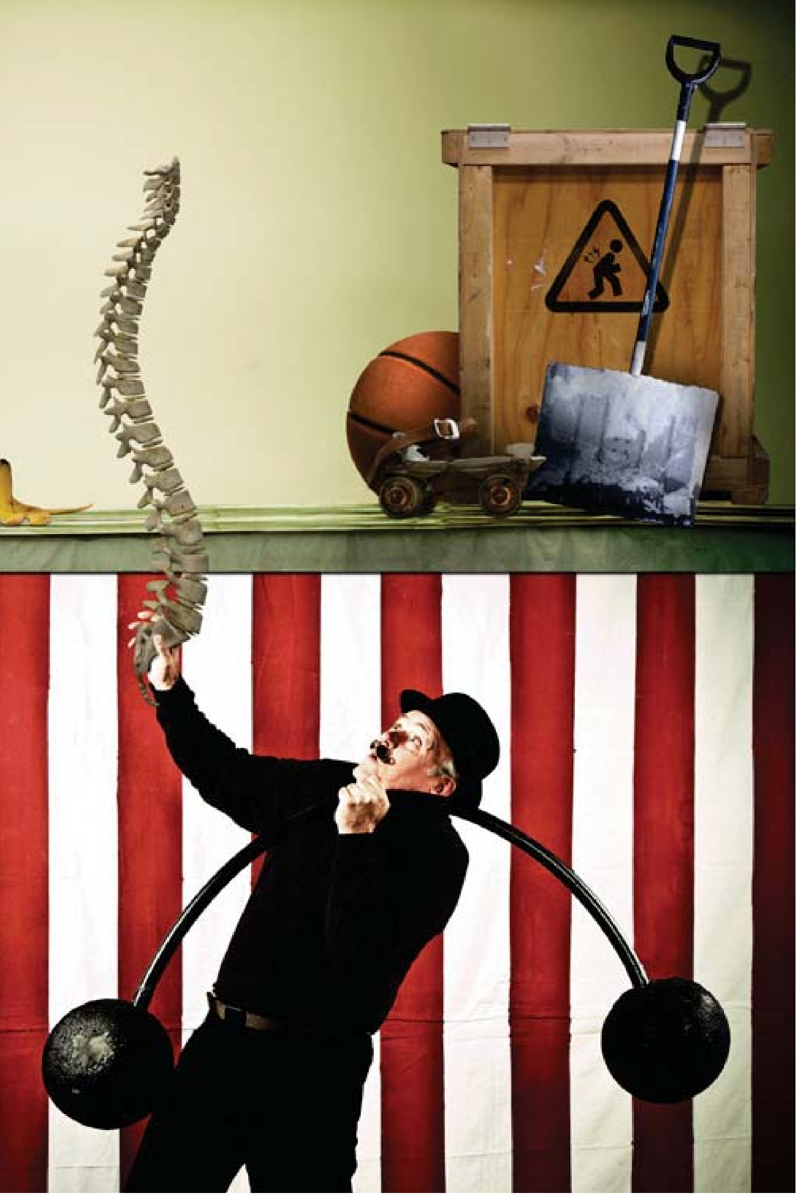

"a sophisticated study…that should provide **important answers** and help direct future interventions in the **treatment** in **low back pain.**"

740.566.PAIN
RELIEF@ohio.edu
www.ohio.edu/RELIEF

There are thousands of clinical trials. Now there is finally one concerned with low back pain. You can help researchers uncover better ways to treat low back pain.

Low back pain is one of the most common reasons for seeking medical care and accounts for over 3.7 million physician visits/year in the U.S. alone. Ninety percent of adults will experience low back pain in their lifetime, 50% will experience recurrent LBP, and 10% will develop chronic pain and related disability. According to a recent national survey, more than 18 million Americans over the age of 18 years received manipulative therapies in 2007 at a total annual out of pocket cost of $3.9 billion.

While there is growing evidence for the clinical effectiveness of alternative and complementary therapies to treat low back pain, little is known on the neurophysiologic consequences and effects of these treatments. Further, additional data is needed to understand how these different treatment techniques effect clinical changes in pain and disability. The lack of empirical data hinders acceptance by the wider scientific and health-care communities, and it also limits the development of rational strategies for using alternative and complementary therapies.

The RELIEF Study is seeking men and women between the ages of 18-45 years to participate in a research study investigating treatment for low back pain. Participation requires completing several testing sessions and attending several treatment sessions.

THE RELIEF STUDY

RESEARCHING THE EFFECTIVENESS OF LUMBAR INTERVENTIONS FOR ENHANCING FUNCTION

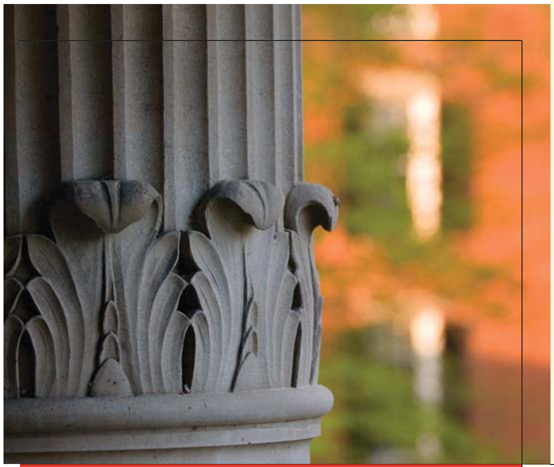

Using cutting edge science to find better treatments for low back pain. There are thousands of clinical trials. Now there is finally one concerned with low back pain.

Get involved. Talk to your doctor. Consider The RELIEF Study. Your involvement helps scientists better understand low back pain. Now is the time for people with low back pain, doctors, and scientists to find relief. Clinical trials are critical in the quest for a better life for people with low back pain. Could you be part of the answer?

Are you...?

- currently experiencing low back pain
- between the ages of 18-45 years old
- able to attend several laboratory testing sessions
- interested in 3-weeks of non-surgical treatment

Research may be right for you. We want to find out what types of interventions work best to treat low back pain and we want to determine how these interventions work. Research may be right for you.

Where?
All appointments, testing, and treatments are conducted at Ohio University in Athens, Ohio.

When?
Participation in the study involves 9 appointments scheduled over an 8-week period.

Who?
The RELIEF Study team consists of professionals in the field who have been selectively chosen and extensively trained. All treatment providers are licensed, experienced clinicians.

Interested?
Contact us. You may be compensated for your time.

Program Contact Information:
Call 740-566-PAIN for more information
RELIEF@ohio.edu

www.ohio.edu/RELIEF

Mission Statement
The Ohio Musculoskeletal Institute and Ohio University are dedicated to learning more about low back pain and finding more effective treatments.

THE RELIEF STUDY
RESEARCHING THE EFFECTIVENESS OF LUMBAR INTERVENTIONS FOR ENHANCING FUNCTION

Back Pain?

Help researchers find answers.

THE RELIEF STUDY
RESEARCHING THE EFFECTIVENESS OF LUMBAR INTERVENTIONS FOR ENHANCING FUNCTION

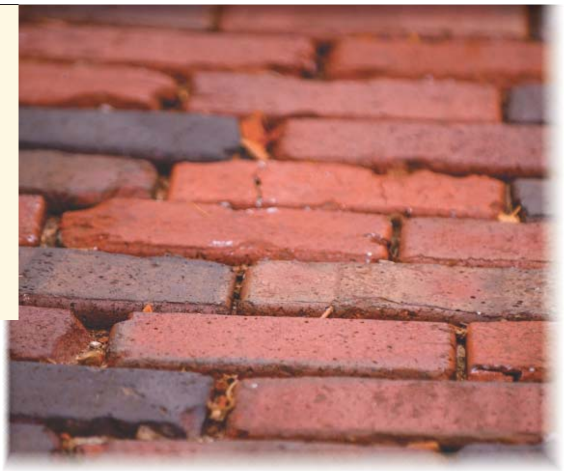

OHIO UNIVERSITY

OHIO UNIVERSITY OHIO MUSCULOSKELETAL AND NEUROLOGICAL INSTITUTE
Heritage College of Osteopathic Medicine

Why research? Why The RELIEF Study?

Local research. Global Impact.

THE RELIEF STUDY

RESEARCHING THE EFFECTIVENESS OF LUMBAR INTERVENTIONS FOR ENHANCING FUNCTION

Research - Clinical Trials

Clinical trials are the best way to determine exactly which treatments work best for people with low back pain. Clinical trials are the final step in medical research on the path between discoveries and treatments improving the lives of people with low back pain.

Participate. Collaborate. Discover.

The health of millions has been improved because of those who have volunteered their time to take part in research studies. There are many ways to become one of those very important volunteers. Study volunteers make a profound gift to science. Remember that healthy volunteers and those with certain medical conditions are important to research, so don't hesitate to get involved.

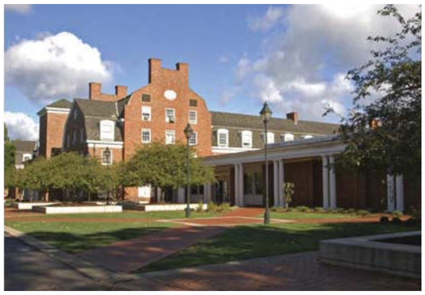

OMNI

The Ohio Musculoskeletal and Neurological Institute (OMNI) OMNI is composed of more than 25 affiliated scientists at Ohio University spanning 8 departments schools (Biomedical Sciences, Family Medicine, Geriatric Medicine, Mechanical Engineering, Psychology, School of Rehabilitation and Communication Sciences, Applied Health Sciences and Wellness) and 4 colleges (Medicine, Health Sciences and Professions, Engineering, Arts and Sciences). Seven of these scientists hold principal investigator status within OMNI.

OMNI's overarching goal is to bring together physicians, engineers, neuroscientists, physiologists, psychologists, and allied health researchers to study a range of problems and disorders of the musculoskeletal and nervous systems.

OHIO UNIVERSITY
OHIO MUSCULOSKELETAL AND NEUROLOGICAL INSTITUTE
Heritage College of Osteopathic Medicine

OMNI was recently awarded a $28 million development and infrastructure enhancement grant from the Osteopathic Heritages Foundations to support OMNI's strategic initiatives. These include building a new 40,000 square foot facility to house OMNI, hiring numerous new faculty and staff members, as well as investing in and supporting OMNI's highest-priority research programs, which are: 1) low back and chronic pain disorders research program, 2) sarcopenia and dynapenia research program, 3) exercise physiology and rehabilitation medicine research program, 4) the biology of manual therapies research program, and 5) bone, connective tissue, and cutaneous biology research program. Ohio University has demonstrated a strong commitment to OMNI, as evidenced by an internal match of more than $5 million to supplement the aforementioned extramural award.

DISCOVERY > Clinical Trial > Improving outcomes for people with low back pain

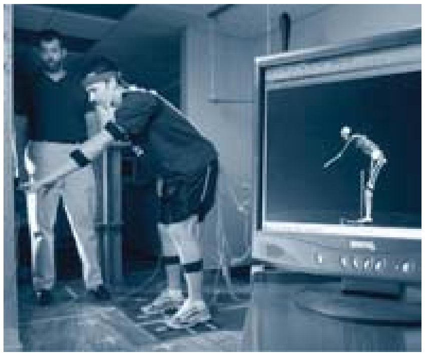

Investigating and Learning

The RELIEF Study is "a sophisticated study…that should provide important answers and help direct future interventions in the treatment in low back pain." - - Dr. James Thomas, P.T., Ph.D., The RELIEF Study co-Principal Investigator

A research study for people with low back pain.

The RELIEF Study isn't about drugs or labs. It is a real life study involving people with pain.

Are you one of the 90%?

Up to 90 percent of us will experience low back pain at some point during our lives. Some recent reports indicate annual costs related to low back pain exceed *$90 billion*. The costs include medical expenses, missed work and lost productivity.

Pain research brings relief

"Low back pain is a clinically significant problem and we are uniquely positioned to take a multidisciplinary approach to address this problem." - Dr. James Thomas, P.T., Ph.D., The RELIEF Study co-Principal Investigator

National Center for Complementary and Alternative Medicine (NCCAM)

OHIO UNIVERSITY

Back Pain?

Looking for more effective treatment?

So are we.

740.566.PAIN (7246)
www.ohio.edu/RELIEF
RELIEF@ohio.edu

THE RELIEF STUDY

RESEARCHING THE EFFECTIVENESS OF LUMBAR INTERVENTIONS FOR ENHANCING FUNCTION

THE RELIEF STUDY

RESEARCHING THE EFFECTIVENESS OF LUMBAR INTERVENTIONS FOR ENHANCING FUNCTION

A randomized clinical trial supported through a grant from the National Institutes of Health National Center for Complimentary and Alternative Medicine.

You can help researchers uncover better ways to treat low back pain.

Low back pain is one of the most common reasons for seeking medical care and accounts for over 3.7 million physician visits/year in the U.S. alone. Ninety percent of adults will experience low back pain in their lifetime, 50% will experience recurrent LBP, and 10% will develop chronic pain and related disability.

While there is growing evidence for the clinical effectiveness of alternative and complementary therapies to treat low back pain, little is known on the neurophysiologic consequences and effects of these treatments. Further, additional data is needed to understand how these different treatment techniques effect clinical changes in pain and disability. The lack of empirical data hinders acceptance by the wider scientific and health-care communities, and it also limits the development of rational strategies for using alternative and complementary therapies.

The RELIEF Study is seeking men and women between the ages of 18-45 years to participate in a research study investigating treatment for low back pain. Participation requires completing several testing sessions and attending several treatment sessions. Financial compensation is provided for participating in this study.

740.566.PAIN (7246)
www.ohio.edu/RELIEF
RELIEF@ohio.edu
