# Supplementary material for: Effect of Spinal Manipulative and Mobilization Therapies in Young Adults With Mild to Moderate Chronic Low Back Pain: A Randomized Clinical Trial
Source: JAMA Netw Open. 2020 Aug 5;3(8):e2012589. doi: 10.1001/jamanetworkopen.2020.12589 (PMC7407093; doi:10.1001/jamanetworkopen.2020.12589)
Supplement: Supplement 2. — Data Sharing Statement [file jamanetwopen-3-e2012589-s002.pdf]

Data Sharing Statement

Thomas. Effect of Spinal Manipulative and Mobilization Therapies in Young Adults With Mild to Moderate Chronic Low Back Pain. *JAMA Netw Open*. Published August 05, 2020.
10.1001/jamanetworkopen.2020.12589

Data
Data available: Yes
Data types: Deidentified participant data
How to access data: jthomas32@vcu.edu
When available: With publication

Supporting Documents
Document types: None

Additional Information
Who can access the data: researchers whose proposed use of the data has been approved
Types of analyses: any purpose
Mechanisms of data availability: with a signed data access agreement
